# Ruthenium-Catalyzed Cascade C—H Functionalization of Phenylacetophenones**

**DOI:** 10.1002/anie.201309114

**Published:** 2014-01-22

**Authors:** Vaibhav P Mehta, José-Antonio García-López, Michael F Greaney

**Affiliations:** School of Chemistry, The University of Manchester, Manchester, M13 9PL (UK)

**Keywords:** cascade chemistry, C–H activation, homogeneous catalysis, oxidative coupling, ruthenium

## Abstract

Three orthogonal cascade C—H functionalization processes are described, based on ruthenium-catalyzed C—H alkenylation. 1-Indanones, indeno indenes, and indeno furanones were accessed through cascade pathways by using arylacetophenones as substrates under conditions of catalytic [{Ru(*p*-cymene)Cl_2_}_2_] and stoichiometric Cu(OAc)_2_. Each transformation uses C—H functionalization methods to form C—C bonds sequentially, with the indeno furanone synthesis featuring a C—O bond formation as the terminating step. This work demonstrates the power of ruthenium-catalyzed alkenylation as a platform reaction to develop more complex transformations, with multiple C—H functionalization steps taking place in a single operation to access novel carbocyclic structures.

Transition-metal-catalyzed C—H functionalization removes the need to prefunctionalize C—H positions for C—C and C—X bond formation, thus enhancing both the scope and efficiency of synthetic route design.^1^ The concept has great potential in the context of cascade synthesis, where an initial C—H functionalization leads to bond formation, with the new motif being primed for a second metal-catalyzed C—H functionalization (Scheme 1). Further iterations are then possible, according to substrate design, resulting in the rapid construction of complex structures with little or no requirement for prefunctionalization.^2^, ^3^ We report our own studies in this area, which have uncovered novel cascade syntheses of polycyclic architectures in a single step by using ruthenium catalysis.

We based our cascade studies on the ruthenium-catalyzed alkenylation reaction, now established as a superbly versatile method for styrene synthesis.^4^–^6^ Arenes containing suitable directing groups (**1**) can undergo *ortho* ruthenation, commonly by using a Ru^II^ complex such as [{Ru(*p*-cymene)Cl_2_}_2_] as the precatalyst, and subsequent reaction with alkenes (**2**) affords the styrene derivatives (**3**). A stoichiometric oxidant is then required to regenerate the Ru^II^ catalyst. The lower cost of ruthenium relative to other noble metals such as palladium^7^ and rhodium^8^ makes ruthenium-catalyzed alkenylation an attractive reaction for developing cascade C—H functionalization. We reasoned that a directing group containing C—H bonds, such as an alkyl ketone, could offer the opportunity for subsequent C—H functionalizations following the initial ruthenium-catalyzed alkenylation step. We chose the readily available α*-*phenyl acetophenone (**4 a**) as our starting substrate, and studied its reaction with methyl acrylate (**5 a**) under ruthenium catalysis with the aim of uncovering cascade C—H functionalization processes (Table 1).

**Scheme 1:**
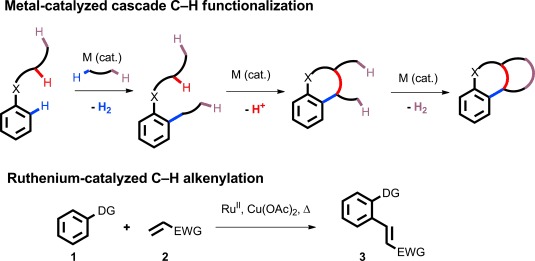
Cascade C—H functionalization. DG=directing group, EWG=electron-withdrawing group.

After an initial coarse screen of solvents and catalysts, (see the Supporting Information) we were delighted to observe the formation of two cascade C—H functionalization products, **6 a** and **7 a**, when using [{Ru(*p*-cymene)Cl_2_}_2_] (5 mol %), AgBF_4_ (20 mol %), Cu(OAc)_2_⋅H_2_O (2 equiv) in DCE at 100 °C (Table 1, entry 1). The 1-indanone **6 a** is the major product and was isolated as a 10:1 mixture of *trans*/*cis* isomers. The intriguing minor compound **7 a** appears to arise from oxidative C—H coupling of *cis-***6 a**, thus resulting in the formation of a tetracyclic 6-5-5-6 structure. This connectivity was initially established by ^1^H NMR and confirmed through X-ray analysis of an analogue subsequently prepared (see below). We elected to optimize the synthesis of the 1-indanone **6 a** in the first instance.

Lowering the catalyst loading below 5 % was unhelpful (Table 1, entry 2), but it did prove effective to lower the amount of silver cocatalyst to 10 % (entry 3). Variation of the silver counterion was tolerated, with little difference between the BF_4_^−^ and SbF_6_^−^ anions (entries 4 and 5). A slight reduction in the amount of Cu(OAc)_2_⋅H_2_O to 1.5 equiv gave a cleaner reaction profile and provided the highest yield of **6 a** with AgSbF_6_ (entry 6; 64 %). Control experiments established the requirement for catalytic Ru and Ag (entries 7 and 8) and stoichiometric Cu(OAc)_2_⋅H_2_O (entry 9). Silver-mediated halide sequestration (to generate cationic Ru centers) is known to assist weakly coordinating directing groups such as ketones in the initial ruthenation step.^6^ We were able, however, to replace the silver cocatalyst activator with a Brønsted acid, with 50 mol % of aqueous HBF_4_ affording a 48 % yield of **6 a** (entry 10). The slightly lower yield was accompanied by several by-products (see below), so we maintained silver cocatalysis to access the indanone products. The reaction proved quite specific for [{Ru(*p*-cymene)Cl_2_}_2_], with other ruthenium- and palladium-based catalysts failing completely (see the Supporting Information). However, the transformation could be mediated by rhodium catalysis, with [{Rh(Cp*)Cl_2_}_2_] (5 mol %) delivering 56 % of **6 a** (entry 11). Lower catalyst loadings of Rh^III^ were not effective, so we elected to continue with the cheaper [{Ru(*p*-cymene)Cl_2_}_2_] catalyst in our reaction development. With optimized conditions in hand for the indanone synthesis, we moved on to investigate the substrate scope of the reaction (Scheme 2a).

**Table 1:** Reaction development. 
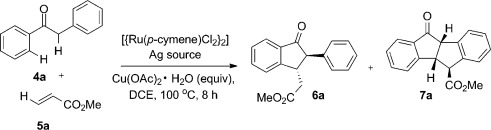

Entry[a]	Ru dimer (mol %)	Ag source (mol %)	Cu(OAc)_2_·H_2_O (equiv)	6 a[%]	7 a[%]
1	5	AgBF_4_ (20)	2	43	11
2	2.5	AgBF_4_ (10)	2	32	4
3	5	AgBF_4_ (10)	2	53	8
4	5	AgOTf (10)	2	45	10
5	5	AgSbF_6_ (10)	2	55	6
6	5	AgSbF_6_ (10)	1.5	64	6
7	–	AgBF_4_ (10)	1.5	–	–
8	5	–	1.5	–	–
9	5	AgBF_4_ (10)	–	–	–
10	5	HBF_4_ (50)	2	48	8
11	^[b]^	AgSbF_6_ (10)	1.5	56	8

[a] Reaction conditions: 0.5 mmol of **4 a**, 1 mmol of **5 a**, Ru dimer, Ag source, Cu(OAc)_2_⋅H_2_O, 2 mL of DCE, *T*=100°C, 8 h. Yields of isolated products are given. [b] [{Rh(Cp^*^)Cl_2_}_2_] (5 mol % of dimer) used. DCE=1,2-dichloroethane, Cp^*^=pentamethylcyclopentadienyl.

The reaction proved versatile with respect to the Michael acceptor, with a range of acrylates cyclizing in 60–64 % yields (**6 a**, **6 b**, **6 c**, and **6 e**). Phenyl vinyl sulfone was also productive, delivering the sulfone **6 d** in 62 % yield, and X-ray analysis of the major diastereoisomer confirmed *trans* stereochemistry.^9^ The reaction was amenable to a five-fold scale-up, with 2.5 mmol of arene **4 a** reacting under the standard conditions to afford a 54 % yield of isolated monocyclized product **6 a**. Substitution on the arene undergoing ruthenation was well tolerated in the position *para* to the ketone, with OMe- and Cl-containing substrates reacting smoothly with a variety of acrylates (**6 f**–**6 i**). *meta* Substitution enabled the steric and electronic character of the C—H functionalization to be probed; Me-containing substrate **4 d** reacted under steric control to direct C—H functionalization to the least hindered position, thus leading to **6 j** in 69 % yield (X-ray analysis). Fluoroarene **4 e**, by contrast, activated the more acidic C—H position next to the fluorine atom and gave **6 k** as the only indanone product (characterized by NOESY NMR). A methoxy group in the *meta* position proved to be deactivating, leading to a mixture of the regioisomers **6 l** and **6′ l** in poor overall yield. This suggests that simple S_E_Ar metallation is not a feature of the mechanism, an observation in line with literature reports on ruthenium-catalyzed alkenylation.^10^ Substitution at the position *ortho* to the ketone proved sensitive to steric factors. Both OMe- and F-containing substrates underwent clean reaction (**6 m**–**6 q**), but Me substitution was unfavorable (30 % and 20 % of **6 r** and **6 s**, respectively), and an *ortho*-CF_3_ group shut down the reaction completely (see the Supporting Information).

**Scheme 2:**
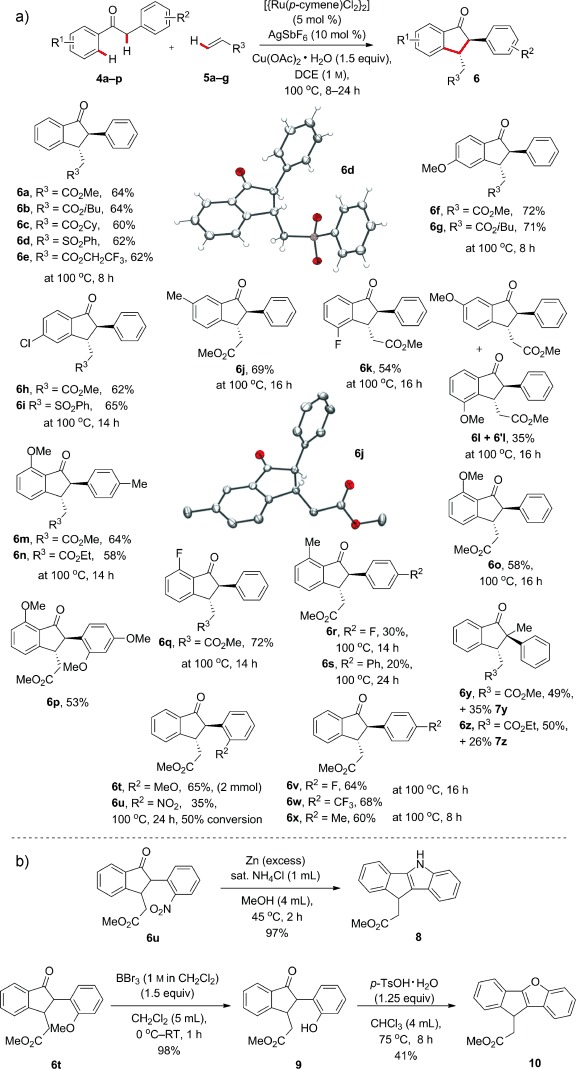
a) 1-Indanone substrate scope. Indanones **6 a**–**6 x** were isolated as the *trans* isomers, d.r.≥10:1 in all cases. b) Further reaction of compounds **6u** and **6t**. TsOH=*p*-toluenesulfonic acid.

Substitution at the arene ring adjacent to the second C—H functionalization was more broadly tolerated, with OMe, F, CF_3_, and Me groups affording indanones in 60–68 % yields (**6 t**, **6 v**–**6 x**). The strongly electron-withdrawing *ortho*-NO_2_ group was an exception, slowing the reaction down to give a 35 % yield of **6 u** after 50 % conversion over 24 h. Finally, we were able to construct the quaternary-center-containing indanones **6 y** and **6 z** starting from α-methyl,α-phenyl acetophenone. The additional methyl substituent promoted the formation of tetracycles **7 y** and **7 z**, possibly by increasing the ratio of *cis* diastereoisomer present after the first double C—H functionalization.

1-Indanones are versatile building blocks in medicinal and materials chemistry. To demonstrate the applicability of the ruthenium-catalyzed tandem process, we conducted further transformations on compounds **6 t** and **6 u** (Scheme 2b). Reduction of the nitro group in **6 u** with zinc gave an aniline, which cyclized in situ to give the dihydroindenoindole **8** in high yield. The dihydroindeno benzofuran **10** was prepared in an analogous fashion from **6 t** through demethylation and acid-mediated condensation. Fused dihydroindeno heterocycles have been widely studied for their biological activity,^11^ as well as applications in organic electronics^12^ and organometallic chemistry (ligands for polymerization catalysts).^13^

We then turned our attention to the tetracycle compound series **7**. Compound **7 a** appears to arise from an oxidative intramolecular arylation reaction of the minor *cis* stereoisomer of **6 a**, a process that would formally involve four separate C—H functionalizations starting from acetophenone **4 a** and methyl acrylate **5 a**. We resubjected **6 a** (*trans/cis*=10:1) to the reaction conditions and observed only a trace of **7 a**, thus suggesting that epimerization under the reaction conditions is not capable of providing sufficient *cis* isomer to undergo further reaction. To encourage formation of the tetracycle, we prepared diarylated acetophenone substrates **11 a**–**11 d** to eliminate the *cis/trans* stereorelationship. Using the same optimized reaction conditions as before, we were pleased to observe sequential C—H functionalization with a range of Michael acceptors to form the pentacycles **12 a**–**12 k** in generally excellent yields (Scheme 3).

X-ray analysis of **12 a** confirmed the indeno indene structure and the *exo* stereochemistry of the ester group. None of the analogous monocyclized 1-indanone products were isolated from the reaction, thus indicating that the oxidative arylation step is highly efficient for the doubly arylated acetophenone substrates **11**. The overall efficiency of the process is notable, with carbocycles **12** being produced in high yields, as single diastereoisomers, through four successive C—H functionalization events.

We uncovered a third, orthogonal mode of cascade C—H functionalization from observations made in the optimization of the initial monocyclization reaction. On using aqueous HBF_4_ in lieu of silver catalysis (Table 1, entry 10), we had observed small amounts of the lactone **13 a** being formed as a side product, the production of which could be increased to a 26 % yield of isolated product on increasing the amount of HBF_4_ to one equivalent. The initial cyclized indanone product **6** undergoes oxidative oxyacylation at the enolic C—H position, a transformation that has been reported on simpler substrates by using hypervalent iodine oxidants,^14^ peroxide oxidation of enamine-type intermediates,^15^ and heavy-metal oxidants such as Tl(OAc)_3_ and Pb(OAc)_4_.^16^ Oxidative oxyacylation by using simple Cu^II^ salts and a Brønsted acid has not been reported, so we were pleased to find that yields could be considerably enhanced by using a silver catalyst as additive in the initial cyclization as before, and then adding a second charge of Cu(OAc)_2_⋅H_2_O/HBF_4_ (aq) to the reaction vessel and heating for a further 16 h. This one-pot, two-step procedure gave a 61 % yield of **13 a** (structure established by X-ray analysis) from phenyl acetophenone **4 a** (Scheme 4). Substrate-scope exploration established the oxidative lactonization step to be efficient, with yields of isolated **13** only slightly lower than those obtained for the corresponding indanones **6** in most cases. Exceptions were noted for strong electron-withdrawing groups in the neighboring arene ring, with substrates containing *ortho*-nitro or *para-*CF_3_ groups failing in the reaction. The efficiency of the oxidative oxyacylation reaction was confirmed by reacting purified 1-indanone **6 a** with HBF_4_ (aq) and Cu(OAc)_2_⋅H_2_O (1.5 equiv), which resulted in the production of lactone **13 a** in 72 % yield. Control experiments established that neither Cu(OAc)_2_·H_2_O or HBF_4_ (aq) alone were effective for oxyacylation from **6 a** and the combination of the two of them was necessary to access the lactone structure.

**Scheme 3:**
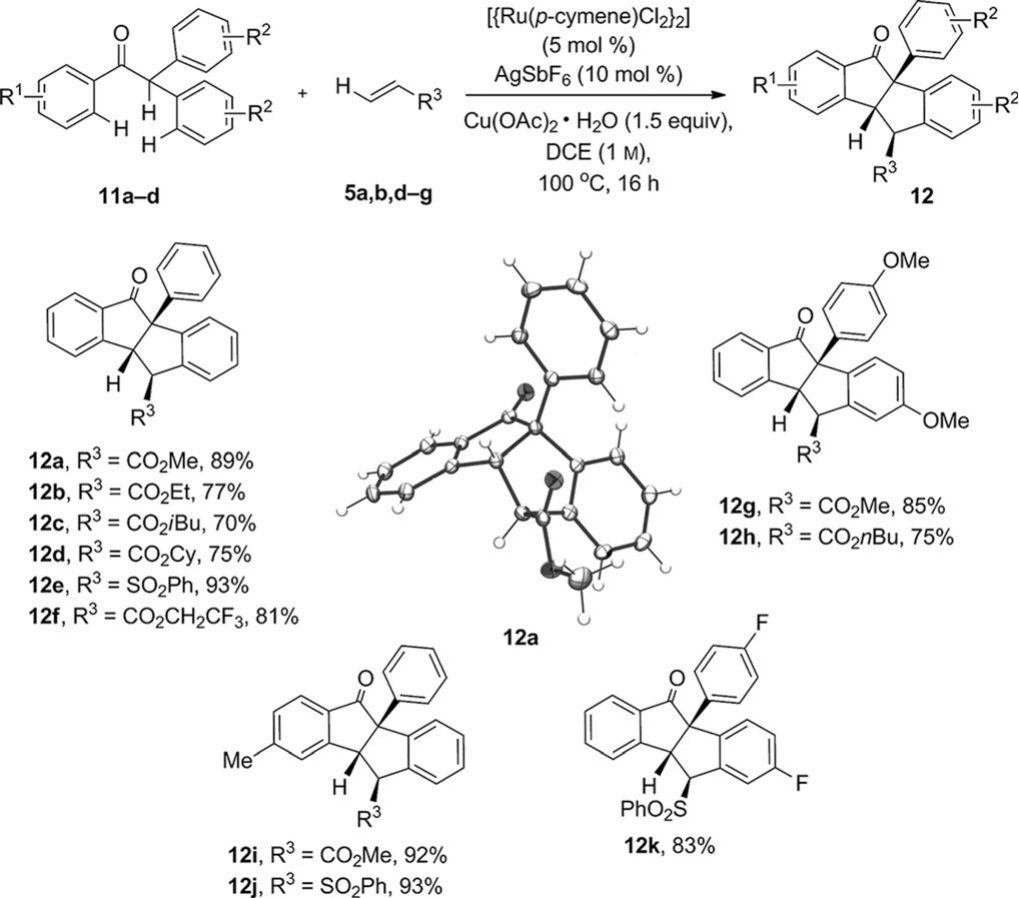
Indeno indene synthesis

Preliminary mechanistic experiments in the 1-indanone and indeno indene pathways led to the outline mechanism shown in Scheme 5. We found that simple acetophenones stopped at the alkenylation stage, in line with observations from Jeganmohan and Padala indicating that the phenyl group is essential to activate the α-C_sp3_—H bond for further C—H functionalization processes.^[6k]^ We then examined the effect of Cu^II^ alone on indanone synthesis, starting from alkenylated substrate **14** [Scheme 5, Eq. (1)]. Somewhat surprisingly, the reaction proceeded to give low yields of the indan-2-one **6 a** and the tetracycle **7 a**. The fact that we had previously observed only trace amounts of **7 a** on treatment of indanone **6 a** with Cu^II^ [Eq. (2)] (similar results with Cu^II^/cat. Ru^II^, and Cu^II^/cat. Ru^II^/Ag^I^, see the Supporting Information) suggests that tetracycle **7 a** is formed from a reactive intermediate in the process, rather than from the product **6 a**. A possibility is shown in [Eq. (3)], whereby radical **16** is generated through deprotonation of the acidic α-hydrogen atom, followed by 1e^−^ oxidation of the stabilized enolate. Radical 5-*exo*-trig addition affords the indanone radical **17**; if R=Ar, then a facile S_Ar_H reaction takes place to yield **12**. If R=H, then the tetracyclization is a minor component owing to the predominately *trans* relationship between the radical and the acceptor arene ring. Although we cannot rule out a 2e^−^ process for the transformation through Michael addition of an initial copper enolate, we note that Kündig et al. have postulated a similar 1e^−^ oxidation and S_Ar_H process for the direct intramolecular arylation of diphenyl propanamides with Cu(OAc)_2_⋅H_2_O,^17^ an analogue of the second C—C bond-forming event of the current reaction. Quenching intermediate **17** in the reaction to give indanone product **6** prevents subsequent oxidative arylation, because the reaction conditions are insufficiently basic/oxidizing to regenerate productive quantities of **17** from the unactivated ester group.

**Scheme 4:**
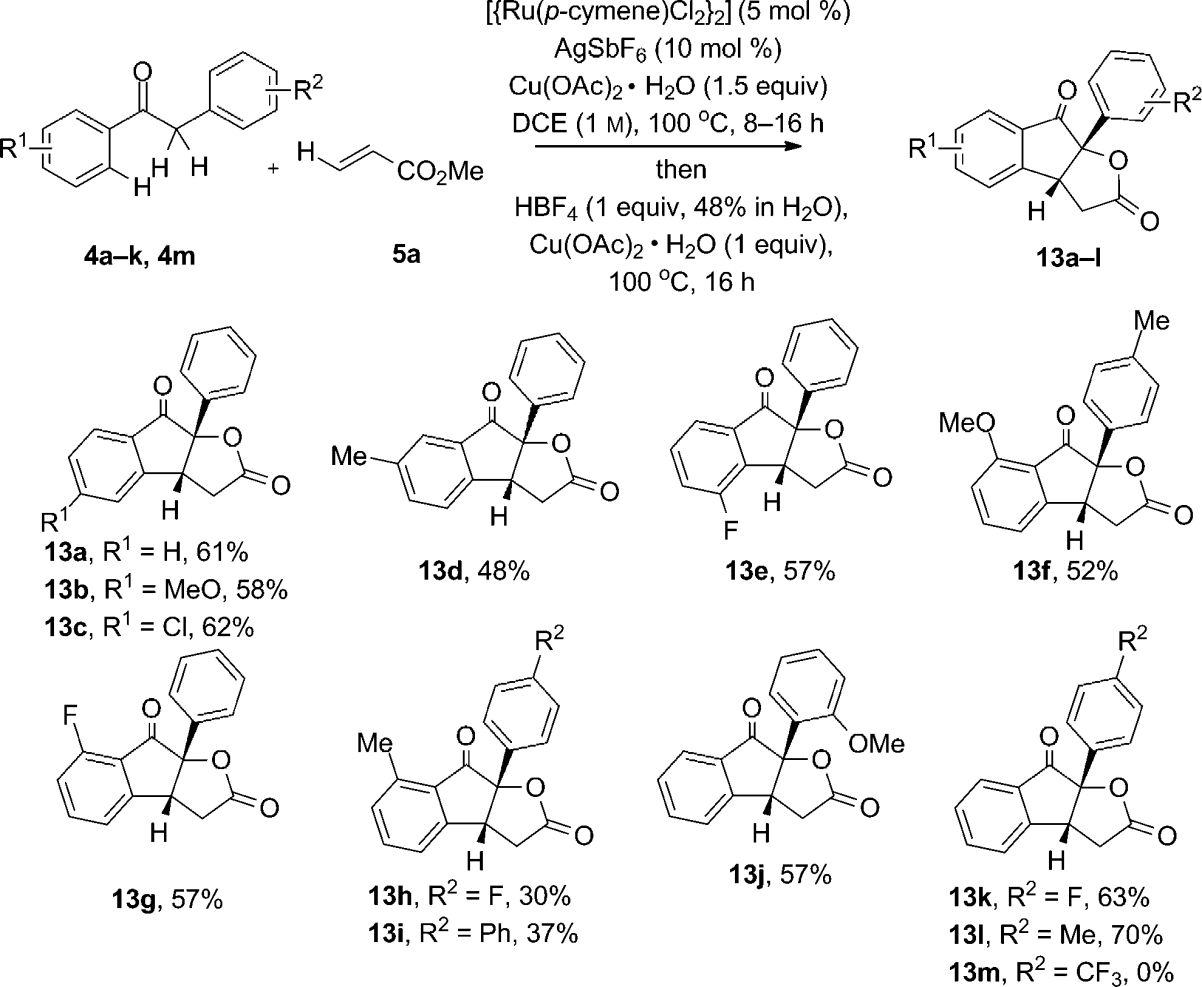
Substrate scope for alkenylation–cyclization–lactonization.

**Scheme 5:**
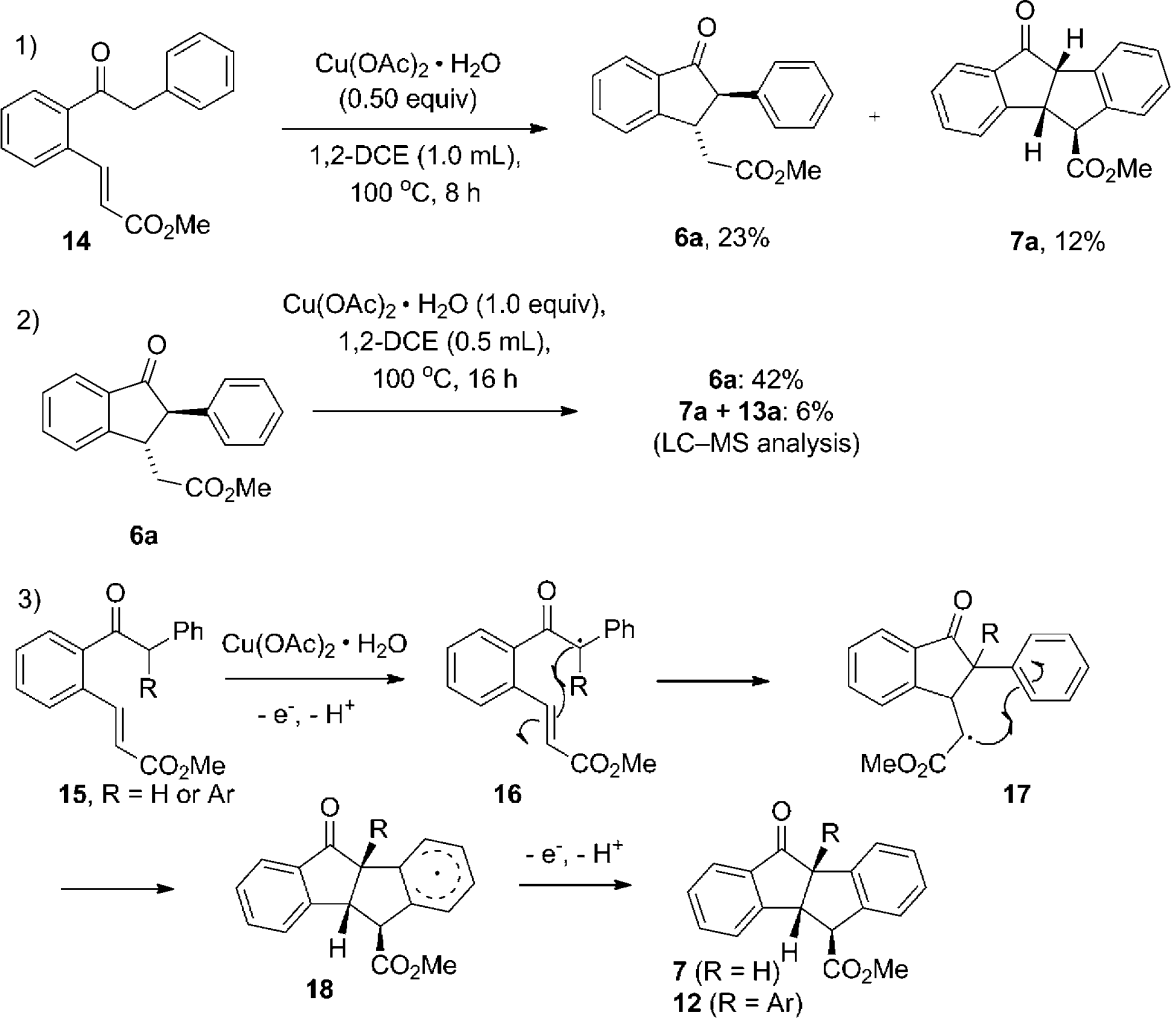
Mechanistic investigations and possible reaction pathway.

In conclusion, we have developed a ruthenium(II)-catalyzed cascade C—H functionalization system that can be directed three different ways according to the choice of substrate and reaction conditions. Simple arylacetophenones react with a variety of Michael acceptors to afford 1-indanones though triple C—H functionalization.

Recharging the reaction vessel with Cu(OAc)_2_⋅H_2_O/aqueous HBF_4_ creates a fourth C—H pathway to the novel lactone structures **13**. Finally, diarylacetophenones undergo four successive C—H functionalizations when treated with Michael acceptors under ruthenium catalysis, thereby giving pentacycles **12** in excellent yield. Further applications of these cascade syntheses are underway.

## Supporting Information

As a service to our authors and readers, this journal provides supporting information supplied by the authors. Such materials are peer reviewed and may be re-organized for online delivery, but are not copy-edited or typeset. Technical support issues arising from supporting information (other than missing files) should be addressed to the authors.

miscellaneous_information
