# Supplementary material for: Ruthenium-Catalyzed Cascade C—H Functionalization of Phenylacetophenones
Source: Angew Chem Int Ed Engl. 2014 Jan 22;53(6):1529–33. doi: 10.1002/anie.201309114 (PMC4265981; doi:10.1002/anie.201309114)

Angewandte Chemie
Eine Zeitschrift der Gesellschaft Deutscher Chemiker

Supporting Information

© Wiley-VCH 2014

69451 Weinheim, Germany

Ruthenium-Catalyzed Cascade C–H Functionalization of Phenylacetophenones**

*Vaibhav P. Mehta, José-Antonio García-López, and Michael F. Greaney**

anie_201309114_sm_miscellaneous_information.pdf

Supporting Information

General Remarks	S2
Experimental procedure and characterization of compounds	S3 – S42
References	S42
^{1}H, ^{13}C and ^{19}F NMR spectra	S43 – S138

General Remarks

Nuclear Magnetic Resonance (NMR) spectra were recorded on a 500, 400 or 300 MHz Bruker NMR spectrometers in $CDCl_3$ at 298 K (unless stated otherwise). All chemical shift values are reported in parts per million (ppm) relative to the solvent signal and were determined in $CDCl_3$, with coupling constant (*J*) values reported in Hz. All spectra were referenced to $CDCl_3$ the residual solvent peak $CHCl_3$ (δ = 7.26 ppm) for ^1H NMR and the $CDCl_3$ solvent peak (δ = 77.16 ppm) for $^{13}C\{^1H\}$ NMR. The notation of signals is: Proton: δ chemical shift in ppm (number of protons, multiplicity, *J* value(s), proton assignment). Carbon: δ chemical shift in ppm (carbon assignment). Fluorine: δ chemical shift in ppm (Fluorine assignment). Splitting patterns are assigned s = singlet, bd = broad doublet, d = doublet, td = triplet of doublet, dt = doublet of triplet, t = triplet, q = quartet, brs = broad singlet. Catalytic reactions were carried out on a 0.50 mmol scale under N_2 using pre-dried glassware. All reactions were carried out in 5 mL glass microwave vials equipped with aluminium crimp caps or a 100 mL round bottomed flask and sealed with a glass stopper and heated in oil baths with a thermocouple temperature control. Toluene, THF and dichloromethane were freshly distilled over sodium or calcium hydride and stored under N_2. 1,2-dichloroethane used for the catalytic reactions was analytical grade (99.5%) and was purged with N_2 for 30 min and stored over dried MS 4 Å. Other solvents, unless otherwise stated, were purchased in reagent grade or anhydrous quality and used as received. Reagents were either purchased directly from commercial suppliers or prepared according to literature procedures. Yields refer to isolated compounds. TLC: Macherey-Nagel, TLC plates Alugram® Sil G/UV254. Detection under UV light at 254 nm. Chromatography: Separations were carried out on Silica gel (Sigma Aldrich, 40-63 μ, 60 Å). Infrared spectra were recorded on a Perkin-Elmer Spectrum One FT-IR spectrometer as a thin film and are reported in cm^{-1}. High Resolution Mass Spectrometry (HRMS) were recorded on Waters QTOF (ES, HRMS). Melting points were determined using a Buchi M565 melting point apparatus.

Synthesis of starting materials

Ketones **4a** and **4c** were purchased from Sigma Aldrich and used as received. The synthesis of α-monoarylated acetophenones **4**, except **4n**[(1)] and **4r**, [(2)] was carried out according to a modified procedure previously reported in the literature for α-arylation of ketones [(3)] (see yields in Table S1 below). α-Diarylated acetophenones **11a-d**[(4)] were synthesized according to literature procedures. All the acetophenones **4** and **11** used as starting materials, excluding the compounds **4i**, **4j**, **4l**, **4s** and **4t**, have been previously been reported in the literature: **4b**;[5,6] **4d**;[7,8] **4e**;[7,9] **4f**;[7,8] **4g**;[10,11] **4h**;[12] **4k**;[11] **4m**;[13,14] **4o**;[8,15] **4p**;[14] **4q**[8,15,16]; **11a**,**c**;[17] **11b**;[18] **11d**[19].

Representative procedure for the synthesis of α-monoarylated acetophenones **4**: Synthesis of compound **4b**. An oven-dried Schlenck tube containing a stir bar was charged with $Pd_2(dba)_3$ (70 mg, 0.076 mmol, 1.5 mol %), DPE-Phos (100 mg, 0.186 mmol, 3.6 mol %), and NaO^tBu (625 mg, 6.5 mmol). The Schlenck tube was evacuated and filled with N_2. THF (5 ml) was added followed by bromobenzene (527 μL, 5 mmol) and *p*-methoxy-acetophenone (825 mg, 5.6 mmol). The resulting mixture was heated under N_2 at 70 ^oC for 3 h. The mixture was cooled to room temperature, water (20 mL) was added and the mixture was extracted with diethyl ether (2 x 50 mL). The organic layers were combined and dried over $MgSO_4$. The filtrate was concentrated and purified by column chromatography on silica gel using a mixture 15/1 of petroleum ether/ethyl acetate to afford compound **4b**. Yield: 800 mg, 3.53 mmol, 71 %.

List of compounds

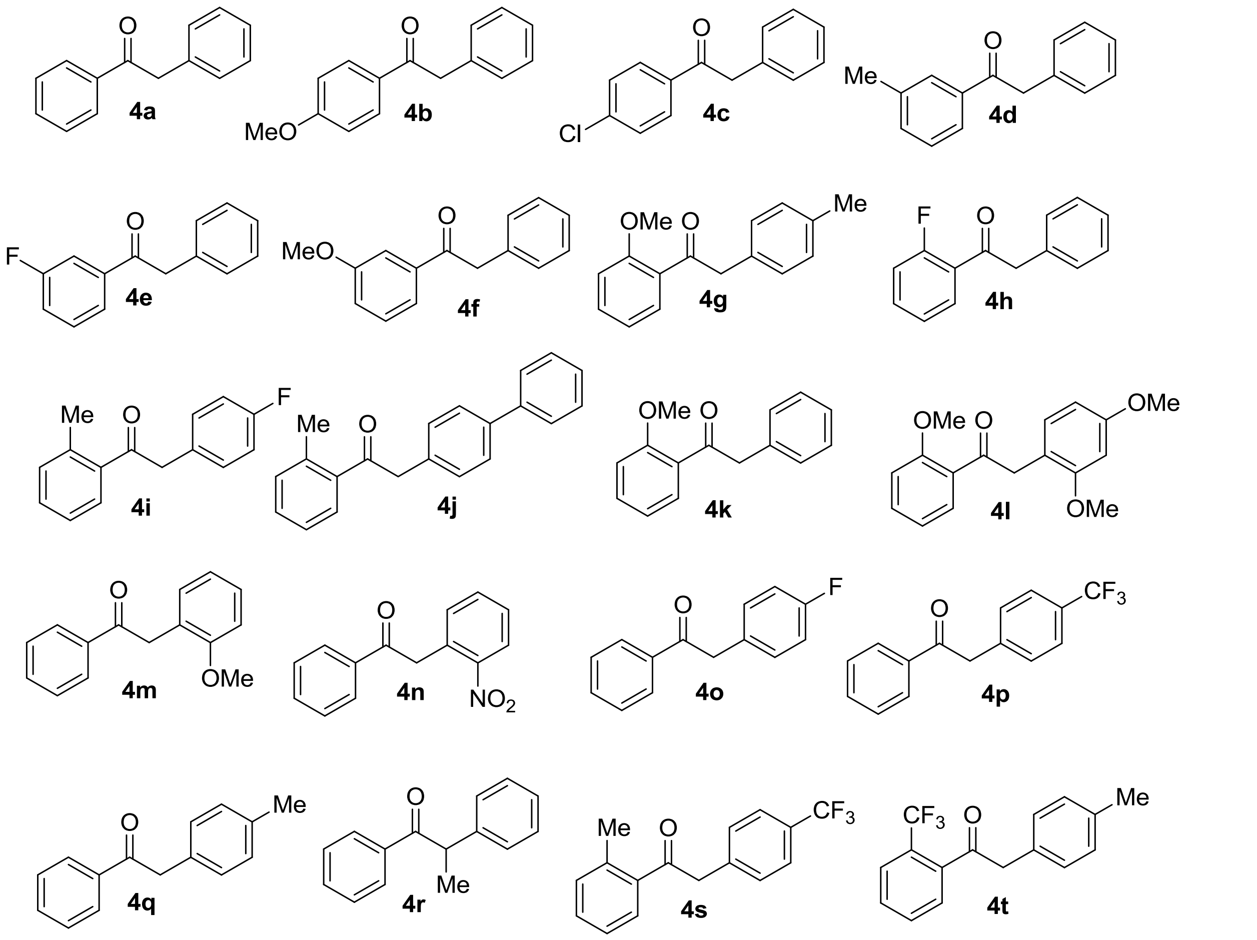

Table S1: Scale of synthesised starting material along with isolated yield.

Compound	Scale (mmol)	yield (%)	Compound	Scale (mmol)	yield (%)
**4b**	5	71	**4l**	5	57
**4d**	2	81	**4m**	5	66
**4e**	2	56	**4n**	5	50
**4f**	3	82	**4o**	3	77
**4g**	5	75	**4p**	1.5	83
**4h**	6	80	**4q**	5	57
**4i**	5	85	**4r**	3	55
**4j**	5	54	**4s**	5	62
**4k**	3	92	**4t**	5	75

Spectroscopic data for novel starting materials.

2-(4-Fluorophenyl)-1-(*o*-tolyl)ethanone (4i). Dense liquid. **^{1}H-NMR** (300 MHz): δ 2.47 (s, 3 H, Me), 4.21 (s, 2 H, CH_2), 7.00-7.08 (m, 2 H), 7.18-7.33 (m, 4 H), 7.40 (td, 1 H, $^{3}J_{HH}$ = 7.5, $^{4}J_{HH}$ = 1.5 Hz), 7.74 (dd, 1 H, $^{3}J_{HH}$ = 7.5, $^{4}J_{HH}$ = 1.5 Hz). **^{13}C{^{1}H}-NMR** (75 MHz): δ 21.3 (s, CH_3), 47.4 (s, CH_2), 115.5 (d, CH, J_{CF} = 21.0 Hz), 125.7 (s, CH), 128.6 (s, CH), 130.1 (d, C, J_{CF} = 3.8 Hz), 131.1 (d, CH, J_{CF} = 7.5 Hz), 131.5 (s, CH), 132.1 (s, CH), 137.4 (s, C), 138.6 (s, C), 161.6 (d, C, J_{CF} = 243.8 Hz), 201.0 (s, CO).**^{19}F{^{1}H}NMR**(376MHz): –116.0 ppm (s). MS (ESI) *m/z* (relative intensity): 229 (100) $[M+H]^{+}$.

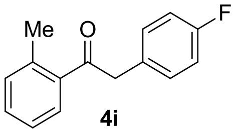

4i

CAS: 1184455-92-2

2-([1,1'-Biphenyl]-4-yl)-1-(*o*-tolyl)ethanone (4j). M.p.: 86-88 ºC. **^{1}H-NMR** (400 MHz): δ 2.39 (s, 3 H, Me), 4.17 (s, 2 H, CH_2), 7.15-7.36 (m, 8 H), 7.48 (m, 4 H), 7.67 (d, 1 H, $^{3}J_{HH}$ = 9.0 Hz). **^{13}C{^{1}H}-NMR** (100 MHz): δ 21.4 (s, Me), 48.0 (s, CH_2), 125.7 (s, CH), 127.1 (s, CH), 127.3 (s, CH), 127.4 (s, CH), 128.75 (s, CH), 128.8 (s, CH), 130.0 (s, CH), 131.5 (s, CH), 132.0 (s, CH), 133.6 (s, C), 137.6 (s, C), 138.7 (s, C), 139.8 (s, C), 140.9 (s, C), 201.3 (s, CO). MS (ESI) *m/z* (relative intensity): 287.3 (100) $[M+H]^{+}$. HR-MS (ESI) *m/z* calcd for $C_{21}H_{19}O_1$ $[M+H]^{+}$ 287.1430, found 287.1430.

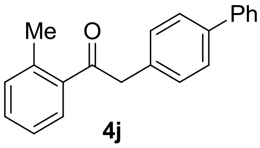

4j

2-(2,4-Dimethoxyphenyl)-1-(2-methoxyphenyl)ethanone 4l. Dense liquid. **^{1}H-NMR** (300 MHz): δ 3.74 (s, 3 H, OMe), 3.81 (s, 3 H, OMe), 3.92 (s, 3 H, OMe), 4.20 (s, 2 H, CH_2), 6.45-6.48 (m, 2 H), 6.97 (t, 1 H, $^{3}J_{HH}$ = 9.0 Hz), 7.01 (d, 1 H, $^{3}J_{HH}$ = 9.0 Hz), 7.07 (br d, 1 H, $^{3}J_{HH}$ = 9.0 Hz), 7.44 (td, 1H, $^{3}J_{HH}$ = 9.0, $^{4}J_{HH}$ = 2.5 Hz), 7.68 (dd, 1 H, $^{3}J_{HH}$ = 9.0, $^{4}J_{HH}$ = 2.5 Hz). **^{13}C{^{1}H}-NMR** (75 MHz): δ 44.7 (s, CH_2), 55.32 (s, OMe), 55.35 (s, OMe), 55.48 (s, OMe), 98.5 (s, CH), 104.2 (s, CH), 111.3 (s, CH), 117.0 (s, C), 120.5 (s, CH), 128.9 (s, C), 130.3 (s, CH), 131.4 (s, CH), 133.0 (s, CH), 158.2 (s, C), 158.4 (s, C), 159.9 (s, C), 200.9 (s, CO). MS (ESI) *m/z* (relative intensity): 287.3 (100) $[M+H]^{+}$. HR-MS (ESI) *m/z* calcd for $C_{17}H_{19}O_4$ $[M+H]^{+}$ 287.1278, found 287.1279.

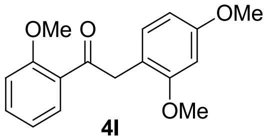

4l

1-(*o*-Tolyl)-2-(4-(trifluoromethyl)phenyl)ethanone 4s. M.p.: 60-62 ºC. **^{1}H-NMR** (300 MHz): δ 2.39 (s, 3 H, Me), 4.22 (s, 2 H, CH_2), 7.18-7.29 (m, 4 H, Ar), 7.33 (td, 1 H, $^{3}J_{HH}$ = 7.0, $^{4}J_{HH}$ = 3.0 Hz), 7.51 (d, 2 H, $^{3}J_{HH}$ = 9.0 Hz), 7.67 (br d, 1 H, $^{3}J_{HH}$ = 9.0 Hz). **^{13}C{^{1}H}-NMR** (75.45MHz): δ 21.4 (s, Me), 47.9 (s, CH_2), 124.2 (q, CF_3, $^{1}J_{CF}$ = 271.6 Hz), 125.5 (q, CH, $^{3}J_{CF}$ = 4.0 Hz), 125.8 (s, CH), 128.7 (s, CH), 129.0 (d, C, $^{2}J_{CF}$ = 32.4 Hz), 130 (s, CH), 131.8 (s, CH), 132.2 (s, CH), 137.1 (s, C), 138.5 (br s, C), 138.9 (s, C), 200.2 (s, CO).**^{19}F{^{1}H}-NMR**(376 MHz): –62.5 ppm (s). MS (ESI) *m/z* (relative intensity): 279.1 (100) $[M+H]^{+}$. HR-MS (EI) *m/z* calcd for $C_{16}H_{13}O_1F_3$ $[M+H]^{+}$ 278.0913, found 278.0901.

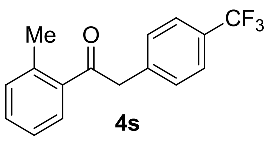

4s

2-(*p*-Tolyl)-1-(2-(trifluoromethyl)phenyl)ethanone 4t. M.p.: 58-60 ºC. **^{1}H-NMR** (300 MHz): δ 2.25 (s, 3 H, Me), 4.03 (s, 2 H, CH_2), 7.01-7.08 (m, 4 H), 7.24-7.27 (m, 1 H), 7.43-7.48 (m, 2 H), 7.61-7.64 (m, 1 H). **^{13}C{^{1}H}-NMR** (75 MHz): δ 21.1 (s, CH_3), 49.7 (br q, CH_2, J_{CF} = 1.5 Hz), 123.7 (q, CF_3, J_{CF} = 273.0 Hz), 126.5 (q, CH, J_{CF} = 4.5 Hz), 126.9 (q, C, J_{CF} = 32.0 Hz), 127.0 127.8 (s, CH), 129.4 (s, CH), 129.6 (s, CH), 130.0 (s, CH), 131.7 (br m, CH), 136.9 (s, C), 140.0 (q, C, J_{CF} = 2.7 Hz), 201.9 (s, CO).**^{19}F{^{1}H}-NMR**(376 MHz): –58.9 ppm (s). MS (ESI) *m/z* (relative intensity): 279.2 (100) $[M+H]^+$. HR-MS (ESI) *m/z* calcd for $C_{16}H_{17}O_1N_1F_3$ $[M+NH_4]^+$ 296.1257, found 296.1261.

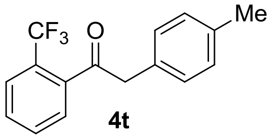

4t

CAS: 1179689-82-7

Optimization study: Representative procedure for synthesis of monocyclized compound 6a.

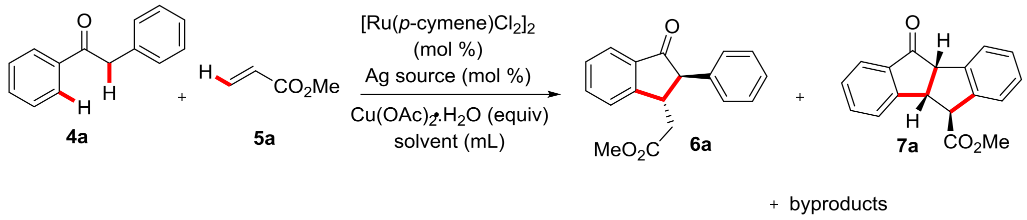

To an oven dried 5 mL microwave vial containing a stir bar was added ketone **4a** (98 mg, 0.50 mmol, 1.00 equiv), Ru[(*p*-cymene)Cl_2]$_2$ (mol%) and $Cu(OAc)_2$•H_2O (equiv). The vial was sealed with a rubber septum and purged with N_2. A freshly prepared stock solution of additive (mol%) in dry solvent (mL) was added to this vial and the mixture was stirred for 10 min at room temperature. Then methyl acrylate **5a** (90 µL, 1.00 mmol, 2.00 equiv) was added via syringe and the rubber septum was replaced with aluminium crimp cap [the reaction was open to air during the swapping of seals]. The vial was tightly sealed and the mixture was heated with stirring in a preheated oil bath at different temperature for stipulated time. After allotted time the mixture was cooled to room temperature, diluted with EtOAc (5 mL) and filtered through celite pad. The pad was further washed with EtOAc (25 mL) and the combined organic solvent was evaporated *in vacuo* to afford a crude mixture. This purified by column chromatography (silica, pet.ether/EtOAc: 9/1) to yield a mixture of **6a** and **7a**. **6a** was isolated as the *trans* isomer predominately, dr = 10:1). Unless otherwise stated the yields reported in the tables are isolated and the ratio of mono- and dicyclized products were determined via ^{1}H-NMR analysis.

Screening of solvents

Table S2

SOLVENT	Total yield **6a + 7a** (%)
1,4-dioxane	Traces
**1,2- DCE**	**54**
DME	Traces
**HFIP**	**55**
H_2O	n.d.
TOLUENE	n.d.
**DCM**	**50**
ACETONITRILE	n.d.
DODECANE	n.d
t-amylOH	traces
DMF	traces
AcOH	traces
THF	traces

Reactions carried out with 0.5 mmol of **4a**, 1.5 equiv of acrylate **5a**, Ru cat (10 mol %), $AgBF_4$ (20 mol%) and $Cu(OAc)_2.H_2O$ (2 equiv) in 2 mL of solvent in a sealed vial for 8 h at 100 ºC. N.d. = not determined, only unreacted starting material.

Comments: 1,2-DCE, DCM and HFIP showed similar conversion on LCMS, but DCM built some overpressure at 100 ºC and HFIP is much more expensive than 1,2-DCE.

Screening of temperature[a]

Table S3

**T (ºC)**	**Ru dimer (mol%)**	**silver (mol%)**	**acrylate (equiv)**	**$Cu(OAc)_2·H_2O$ (equiv)**	**total yield 6a + 7a (%)**
80	5	$AgBF_4$ (10)	3	2	45
**100**	**5**	**$AgBF_4$ (10)**	**3**	**2**	**57**

110	5	$AgBF_4$ (10)	3	2	56
80	4	$AgBF_4$ (20)	2	2	33
80	4	$AgSbF_6$ (20)	2	2	33
60	5	$AgSbF_6$ (10)	2	2	-[b]

[a] Reactions carried out with 0.5 mmol of **4a**, in 2 mL of 1,2-DCE in a sealed vial for 8 h. [b] Only uncyclised alkenylated product isolated.

Comments: Higher temperature leads to more byproduct formation, while lower reaction temperature shows incomplete reaction.

Screening of reaction time

Table S4

**Ru dimer (mol%)**	**silver (mol%)**	**$Cu(OAc)_2{\cdot}H_2O$ (equiv)**	**acrylate (equiv)**	**time (h)**	**total yield 6a + 7a (%)**
5	$AgBF_4$ (10)	1.5	2	1	42
5	$AgBF_4$ (10)	1.5	2	2	44
5	$AgBF_4$ (10)	1.5	2	4	49
**5**	**$AgBF_4$ (10)**	**1.5**	**2**	**8**	**60**
5	$AgBF_4$ (10)	1.5	2	16	60

Reactions carried out with 0.5 mmol of **4a**, in 2 mL of 1,2-DCE at 100 °C in a sealed vial.

Comment: Prolonged reaction time does not improve in reaction yields.

Screening of catalyst loading

Table S5

**Ru dimer (mol%)**	**silver (mol%)**	**$Cu(OAc)_2·H_2O$ (equiv)**	**acrylate (equiv)**	**total yield 6a + 7a (%)**
**5**	**$AgBF_4$ (10)**	**1.5**	**2**	**61**
2.5	$AgBF_4$ (10)	1.5	2	48
5	$AgSbF_6$ (10)	2	3	56
2.5	$AgSbF_6$ (10)	2	3	52
5	$AgBF_4$ (10)	1.1	2	57
2.5	$AgBF_4$ (10)	1.1	2	53

Reactions carried out with 0.5 mmol of **4a**, in 2 mL of 1,2-DCE at 100 °C for 8 h in a sealed vial.

Comment: Lower amount of Ru-catalyst is less effective, leading to formation of uncyclised alkenylated byproduct.

Screening of silver source and stoichiometry

Table S6

**silver source**	**silver (mol%)**	**Ru dimer (mol%)**	**$Cu(OAc)_2·H_2O$ (equiv)**	**acrylate (equiv)**	**total yield 6a + 7a (%)**
-	-	5	2	2	0
$AgBF_4$	20	5	2	2	54
$AgSbF_6$	20	5	2	2	37

$AgBF_4$	20	2.5	2	2	36
$AgBF_4$	10	5	2	2	55
AgOTf	10	5	2	2	55
**$AgSbF_6$**	**10**	**5**	**2**	**2**	**56**
$AgSbF_6$	10	2.5	2	3	52
AgOAc	20	5	2	2	traces

Reactions carried out with 0.5 mmol of **4a**, in 2 mL of 1,2-DCE at 100 ºC for 8 h in a sealed vial.

Comment: Best yields are obtained having half equiv of the Ag source relative to Cl content of the Ru-complex.

Screening of concentration

Table S7

**Solvent (mL)**	**silver (mol %)**	**Ru dimer (mol %)**	**$Cu(OAc)_2 \cdot H_2O$ (equiv)**	**acrylate (equiv)**	**total yield 6a + 7a (%)**
5	$AgBF_4$(10)	5	1.1	2	43
2	$AgBF_4$(10)	5	1.1	2	57
2	$AgBF_4$(10)	5	1.5	2	60
**0.5**	**$AgBF_4$(10)**	**5**	**1.5**	**2**	**62**
0.5	$AgBF_4$(10)	5	2	2	61

Reactions carried out with 0.5 mmol of **4a**, in 1,2-DCE at 100 ºC in a sealed vial for 8 h.

Comment: A 1M concentration of substrate proved optimal.

Screening of $Cu(OAc)_2·H_2O$ stoichiometry

Table S8

**$Cu(OAc)_2·H_2O$ (equiv)**	**Ru dimer (%)**	**silver (mol %)**	**acrylate (equiv)**	**solvent (mL)**	**total yield 6a + 7a (%)**
2	5	$AgBF_4$ (10)	2	2	55
1.5	5	$AgBF_4$ (10)	2	2	60
1.1	5	$AgBF_4$ (10)	2	2	56
1.5	5	$AgBF_4$ (10)	2	0.5	61
**1.5**	**5**	**$AgSbF_6$ (10)**	**2**	**0.5**	**68**
1.5	5	AgOTf (10)	2	0.5	54

Reactions carried out with 0.5 mmol of **4a** in 1,2-DCE at 100 ºC in a sealed vial for 8 h. Other oxidants such as oxone and $K_2S_2O_8$ failed to give the desired products.

Screening of additives[a]

Table S9

**ADDITIVE (mol %)**	**Ru dimer (mol%)**	**$AgBF_4$ (mol%)**	**$Cu(OAc)_2·H_2O$ (equiv)**	**solvent (mL)**	**total yield 6a + 7a (%)**
-	5	10	1.5	2	60
CsOPiv (30)	2.5	10	1.5	2	traces
NaOAc (100)	2.5	10	1.5	2	traces
Cs_2CO_3 (200)	2.5	10	1.5	2	traces

**Pivalic acid (30)**	**5**	**10**	**1.5**	**0.5**	**61**
KPF_6 (20)	5	-	1.5	2	0
HBF_4[b] (30)	5	-	1.5	0.5	45
HBF_4[b] (50)	5	-	2	0.5	38
**HBF_4[b, c] (50)**	**5**	**-**	**2**	**2**	**54**

[a] Reactions carried out with 0.5 mmol of **4a**, 2.0 equiv of **5a**, in 1,2-DCE, 100 ºC, for 8 h. [b] HBF_4 (48% soln. in H_2O) used; [c] Reaction carried out using 3.0 equiv of **5a** and run for 24 h.

Comment: Pivalic acid was tolerated as an additive, but did not improve the reaction yield. The silver promoter could be replaced by the cheaper aq HBF_4 reagent to generate active, cationic Ru-species, resulting in a slight drop in yield (54%). Use of excess of HBF_4 reagent leads to generation of lactone byproducts.

Screening of other metal catalysts

Table S10

**catalyst (metal mol %)**	**$AgBF_4$ (mol%)**	**total yield of 6a + 7a (%)**
$[Ru(Cp^*)Cl(PPh_3)_2]$ (10)	10	-
$[Ru(Cp^*)Cl_2]_2$ (10)	10	-
$RuCl_3$ (10)+ PPh_3 (20)	10	-
$[PdCl_2(MeCN)_2]$ (10)	20	-
$Pd(OAc)_2$ (10)	-	-
**$[Rh(Cp^*)Cl_2]_2$ (10)**	**10**	**64**

Reactions carried out with 0.5 mmol of **4a**, 2 equiv of **5a**, 2 equiv of $Cu(OAc)_2$•H_2O in 2 mL of 1,2-DCE at 100 ºC in a sealed vial for 8 h.

Comment: Use of an analagous Rh-catalyst afforded the same isolated yield of **6a** and **7a** in comparison with the Ru-catalyst. Lowering the loading of the expensive Rh-catalyst proved ineffective and unreacted starting material was observed after reaction.

Table S11. Optimization table for lactone product 13a.[a]

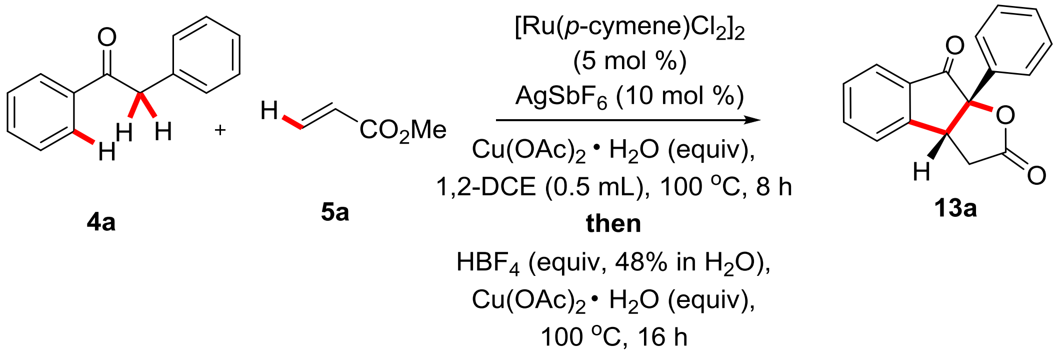

4a 5a 13a

entry	additive (%)	$Cu(OAc)_2{\cdot}H_2O$(equiv)		HBF_4 (48% soln. in H_2O) (equiv)	yield **13a** (%)
		first step	second step		
1	-	1.5	-	1	26
2	-	2	-	1	38
3	-	2	-	0.5	traces[b]
4	$AgSbF_6$ (10)/ K_2CO_3 (100)	1.5	-	-	-
5	$AgBF_4$ (10)	1.5	0.3	1	58[c]
6	$AgBF_4$ (10)	1	1	-	35[c d]
**7**	**$AgBF_4$ (10)**	**1.5**	**1**	**1**	**61**[c]
8	$AgBF_4$ (10)	1.5	0.5	0.3	45[c]

[a] Reactions were carried out using 0.5 mmol of **4a** in 0.5 mL of 1,2-DCE at 100 ºC for 8 h. Yields reported are isolated. [b] 54% of product **6a** was isolated. [c] Reactions were carried out in one pot and two steps, adding extra amount of $Cu(OAc)_2{\cdot}H_2O$ and then HBF_4 and further heating for another 16 h. [d] 25% of product **6a** was isolated.

Representative procedure A for the synthesis of monocyclized compounds 6a – 6z, 6ab-6ag.

To an oven dried 5 mL microwave vial containing stir bar was added ketone **4a** (98 mg, 0.50 mmol, 1.00 equiv), Ru[(*p*-cymene)Cl_2]$_2$ (15.2 mg, 5.0 mol%) and $Cu(OAc)_2$•H_2O (150 mg, 0.75 mmol, 1.50 equiv). The vial was sealed with rubber septum and purged with N_2. A freshly prepared stock solution of $AgSbF_6$ (17.5 mg, 10 mol%) in dry 1,2-dichloroethane (0.50 mL) was added to this vial and the mixture was stirred for 10 min at room temperature. Then methyl acrylate **5a** (90 μL, 1.00 mmol, 2.00 equiv) was added via syringe and the rubber septum was replaced with aluminium crimp cap [the reaction was open to air during the process of swapping seals]. The vial was tightly sealed and the mixture was heated with stirring in a preheated oil bath at 100 ^oC for 8 h. After allotted time the mixture was cooled to room temperature, diluted with EtOAc (5 mL) and filtered through a celite pad. The pad was further washed with EtOAc (25 mL) and the combined organic solvent was evaporated *in vacuo* to afford a crude mixture. Purification by column chromatography (silica, pet.ether/EtOAc: 9/1) gave **6a** (90 mg, 0.32 mmol, 64%) as a yellow solid, inseparable mixture of *trans* and *cis* isomers, dr = 10:1 (^{1}H-NMR analysis. The relative peak area for –CH_2-group in the range δ = 2.94 – 2.90 ppm and δ = 2.70 – 2.64 ppm was considered for determining the diastereomeric ratios). NMR data for the major *trans* isomer is reported throughout.

Reaction on 2.5 mmol scale

To an oven dried 50 mL schlenk flask containing stir bar was added ketone **4a** (490 mg, 2.50 mmol, 1.00 equiv), Ru[(*p*-cymene)Cl_2]$_2$ (76 mg, 5.0 mol%) and $Cu(OAc)_2$•H_2O (750 mg, 3.75 mmol, 1.50 equiv). The flask was sealed with rubber septum and purged with N_2. A freshly prepared stock solution of $AgSbF_6$ (87.5 mg, 10 mol%) in dry 1,2-dichloroethane (3.00 mL) was added to the flask and the mixture was stirred for 10 min at room temperature. Then methyl acrylate **5a** (450 μL, 5.00 mmol, 2.00 equiv) was added dropwise via syringe and the rubber septum was replaced with glass stopper [the reaction was open to air during the process of swapping seals]. The flask was tightly sealed and the mixture was heated with stirring in a preheated oil bath at 100 ^oC for 8 h. (Note: the flask was shaken occasionally to ensure all $Cu(OAc)_2$•H_2O remains in the mixture). After allotted time the mixture was cooled to room temperature, diluted with EtOAc (25 mL) and filtered through a celite pad. The pad was further washed with EtOAc (100 mL) and the combined organic solvent was evaporated *in vacuo* to afford a crude mixture. This was purified by column chromatography (silica, pet.ether/EtOAc: 9/1) to yield **6a** (378 mg, 1.35 mmol, 54%) as a yellow solid (dr = 10:1 *trans* : *cis*). In addition, dicyclized product **7a** (42 mg, 0.15 mmol, 6%) was isolated as a yellow solid and as a single diastereomer as determined via ^{1}H-NMR analysis.

Methyl 2-(3-oxo-2-phenyl-2,3-dihydro-1*H*-inden-1-yl)acetate (6a): The representative procedure A is followed. R_f = 0.17 (pet.ether/EtOAc: 9/1). ^{1}H-NMR (300 MHz, $CDCl_3$): δ = 7.83 (d, *J* = 7.9 Hz, 1H), 7.68 (dt, *J* = 8.4, 1.0 Hz, 1H), 7.54 (d, *J* = 7.6 Hz, 1H), 7.47 (t, *J* = 7.4 Hz, 1H), 7.35 – 7.27 (m, 3H), 7.15 (dd, *J* = 8.6, 1.9 Hz, 2H), 3.90 (m, 1H), 3.65 (d, *J* = 4.5 Hz, 1H), 3.58 (s, 3H), 2.95 (dd, *J* = 15.5, 6.2 Hz, 1H), 2.79 (dd, *J* = 15.5,

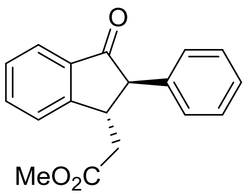

7.6 Hz, 1H). ^{13}C-NMR (75 MHz, $CDCl_3$): δ = 204.5 (C_q), 172.0 (C_q), 155.2 (C_q), 138.6 (C_q), 136.1 (C_q), 135.4 (CH), 129.0 (CH), 128.6 (CH), 128.4 (CH), 127.4 (CH), 125.3 (CH), 124.5 (CH), 60.7 (CH), 51.9 (OCH_3), 44.6 (CH), 39.2 (CH_2). MS (ESI) *m/z* (relative intensity): 303.4 (60) $[M+Na]^+$. HR-MS (ESI) *m/z* calcd for $C_{18}H_{17}O_3$ $[M+H]^+$ 281.1172, found 281.1162.

Methyl 10-oxo-4b,5,9b,10-tetrahydroindeno[2,1-*a*]indene-5-carboxylate (7a): M. p.= 68 – 70 °C. (single diastereomer) R_f = 0.21 (pet.ether/EtOAc: 9/1). ^{1}H-NMR (300 MHz, $CDCl_3$): δ = 7.72 (d, *J* = 7.6 Hz, 1H), 7.67 – 7.60 (m, 3H), 7.41 – 7.35 (m, 2H), 7.30 – 7.22 (m, 2H), 4.63 (dd, *J* = 6.8, 2.5 Hz, 1H), 4.42 (d, *J* = 6.8 Hz, 1H), 4.25 (brs, 1H), 3.82 (s, 3H). ^{13}C-NMR (75 MHz, $CDCl_3$): δ = 204.1 (C_q), 173.5 (C_q), 156.3 (C_q), 139.5 (C_q), 139.3 (C_q), 135.9 (C_q), 135.6 (CH), 128.8 (CH), 128.5 (CH), 128.4 (CH), 126.1 (CH), 125.9 (CH), 124.4 (CH), 124.6 (CH), 58.5 (CH), 56.5 (CH), 52.8 (OCH_3), 46.1 (CH). MS (ESI) *m/z* (relative intensity): 301.3 (50) $[M+Na]^+$. HR-MS (ESI) *m/z* calcd for $C_{18}H_{15}O_3$ $[M+H]^+$ 279.1016, found 279.1015.

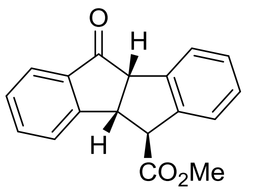

Isobutyl 2-(3-oxo-2-phenyl-2,3-dihydro-1*H*-inden-1-yl)acetate (6b): Representative procedure A was followed using ketone **4a** (98 mg, 0.50 mmol, 1.00 equiv) and isobutyl acrylate **5d** (108 µL, 0.75 mmol, 1.50 equiv) for 8 h. Purification by column chromatography (pet.ether/EtOAc: 9/1) gave **6b** (103 mg, 0.32 mmol, 64%) as a yellow oil (dr = 10:1). R_f = 0.23 (pet.ether/EtOAc: 9/1). ^{1}H-NMR (400 MHz, $CDCl_3$): δ = 7.76 (d, *J* = 7.6 Hz, 1H), 7.62 (dt, *J* = 7.4, 1.0 Hz, 1H), 7.51 (dd, *J* = 7.9, 0.8 Hz, 1H), 7.41 (t, *J* = 7.4 Hz, 1H), 7.28 – 7.18 (m, 3H), 7.09 (dd, *J* = 8.3, 1.6 Hz, 2H), 3.84 (m, 1H), 3.70 (m, 2H), 3.61 (d, *J* = 4.6 Hz, 1H), 2.88 (dd, *J* = 15.4, 6.1 Hz, 1H), 2.74 (dd, *J* = 15.4, 7.6 Hz, 1H), 1.77 – 1.66 (septet, 1H), 0.77 (d, *J* = 3.7 Hz, 3H), 0.76 (d, *J* = 3.7 Hz, 3H). ^{13}C-NMR (101 MHz, $CDCl_3$): δ = 204.6 (C_q), 171.6 (C_q), 155.3 (C_q), 138.6 (C_q), 136.0 (C_q), 135.4 (CH), 128.9 (CH), 128.5 (CH), 128.4 (CH), 127.4 (CH), 125.3 (CH), 124.5 (CH), 71.1 (CH_2), 60.6 (CH), 44.6 (CH), 39.4 (CH_2), 27.6 (CH), 19.2 (CH_3), 19.1 (CH_3). MS (ESI) *m/z* (relative intensity): 345.2 (100) $[M+Na]^+$. HR-MS (ESI) *m/z* calcd for $C_{21}H_{22}O_3Na_1$ $[M+Na]^+$ 345.1461, found 345.1467.

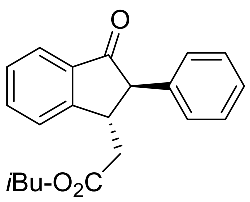

Cyclohexyl 2-(3-oxo-2-phenyl-2,3-dihydro-1*H*-inden-1-yl)acetate (6c): Representative procedure A was followed using ketone **4a** (98 mg, 0.50 mmol, 1.00 equiv) and cyclohexyl acrylate **5e** (157 µL, 1.00 mmol, 2.00 equiv) for 8 h. Purification by column chromatography (pet.ether/EtOAc: 9/1) gave **6c** (105 mg, 0.30 mmol, 60%) as a yellow oil (dr = 10:1). R_f = 0.45 (pet.ether/EtOAc: 9/1). ^{1}H-NMR (500 MHz, $CDCl_3$): δ = 7.76 (d, *J* = 7.5 Hz, 1H), 7.60 (dt, *J* = 7.4, 1.0 Hz, 1H), 7.50 (dd, *J* = 7.9, 0.8 Hz, 1H), 7.40 (tt, *J* = 7.4, 0.9 Hz, 1H), 7.24 (t, *J* = 7.0 Hz, 2H), 7.18 – 7.17 (m, 1H), 7.08 (dd, *J* = 8.4, 1.6 Hz, 2H), 4.59 (m, 1H), 3.80 (m, 1H), 3.61 (d, *J* = 4.6 Hz, 1H), 2.83 (dd, *J* = 15.5, 5.9 Hz, 1H), 2.70 (dd, *J* = 15.5, 7.5 Hz, 1H), 1.70 – 1.40 (m, 6H), 1.24 – 1.18 (m, 2H), 1.14 – 1.07 (m, 2H). ^{13}C-NMR (125 MHz, $CDCl_3$): δ = 204.7 (C_q),

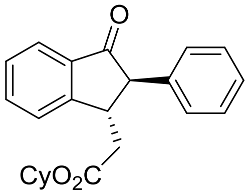

171.0 (C_q), 155.4 (C_q), 138.7 (C_q), 136.1 (C_q), 135.3 (CH), 128.9 (CH), 128.5 (CH), 128.4 (CH), 127.3 (CH), 125.4 (CH), 124.5 (CH), 73.4 (CH), 60.5 (CH), 44.6 (CH), 39.7 (CH_2), 31.6 (CH_2), 31.4 (CH_2), 25.2 (CH_2), 23.8 (CH_2), 23.7 (CH_2). MS (ESI) *m/z* (relative intensity): 371.4 (100) $[M+Na]^+$. HR-MS (ESI) *m/z* calcd for $C_{23}H_{24}O_3Na_1$ $[M+Na]^+$ 371.1618, found 371.1615.

2-Phenyl-3-((phenylsulfonyl)methyl)-2,3-dihydro-1*H*-inden-1-one (6d): Representative procedure A was followed using ketone **4a** (98 mg, 0.50 mmol, 1.00 equiv) and phenyl vinyl sulfone **5f** (126 mg, 0.75 mmol, 1.50 equiv) for 8 h. Purification by column chromatography (pet.ether/EtOAc: 4/1) gave **6d** (112 mg, 0.31 mmol, 62%) as a yellow solid (dr > 20:1). R_f = 0.28 (pet.ether/EtOAc: 3/1). The major *trans* diastereomer crystallized from $CDCl_3$ via vapour diffusion giving white crystals suitable for X-ray crystallography. M.p. = 158 – 160 °C. ^{1}H-NMR (500 MHz, $CDCl_3$): δ = 7.64 (d, *J* = 7.7 Hz, 1H), 7.54 (dd, *J* = 8.4, 1.3 Hz, 2H), 7.52 (dd, *J* = 6.6, 1.2 Hz, 1H), 7.40 (d, *J* = 7.4 Hz, 1H), 7.43 (tt, *J* = 7.3, 1.3 Hz, 1H), 7.34 (t, *J* = 7.3 Hz, 1H), 7.26 (t, *J* = 7.4 Hz, 2H), 7.12 – 7.09 (m, 3H), 6.90 (dd, *J* = 7.7, 2.3 Hz, 2H), 3.76 (d, *J* = 3.4 Hz, 1H), 3.72 (m, 1H), 3.52 (dd, *J* = 14.6, 4.1 Hz, 1H), 3.30 (dd, *J* = 14.6, 8.9 Hz, 1H). ^{13}C-NMR (125 MHz, $CDCl_3$): δ = 203.7 (C_q), 153.7 (C_q), 138.6 (C_q), 138.1 (C_q), 135.8 (C_q), 135.7 (CH), 134.1 (CH), 129.5 (CH), 129.1 (CH), 128.9 (CH), 128.1 (CH), 127.5 (CH), 125.9 (CH), 124.8 (CH), 61.3 (CH_2), 59.5 (CH), 42.7 (CH). MS (ESI) *m/z* (relative intensity): 385.3 (100) $[M+Na]^+$. HR-MS (ESI) *m/z* calcd for $C_{22}H_{18}O_3Na_1S_1$ $[M+Na]^+$ 385.0874, found 385.0870.

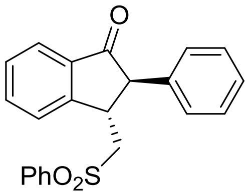

2,2,2-Trifluoroethyl 2-(3-oxo-2-phenyl-2,3-dihydro-1H-inden-1-yl)acetate (6e): Representative procedure A was followed using ketone **4a** (98 mg, 0.50 mmol, 1.00 equiv) and 2,2,2-trifluoroethyl acrylate **5g** (127 µl, 1.00 mmol, 2.00 equiv) for 8 h. Purification by column chromatography (pet.ether/EtOAc: 9/1) gave **6e** (108 mg, 0.31 mmol, 62%) as a yellow oil (dr = 16:1), along with the dicyclized product **(7e)** as a yellow solid (10.4 mg, 0.03 mmol, 6%) (single diastereoisomer). **6e**: R_f = 0.31 (pet.ether/EtOAc: 9/1). ^{1}H-NMR (400 MHz, $CDCl_3$): δ = 7.76 (d, *J* = 7.6 Hz, 1H), 7.62 (dt, *J* = 8.0, 1.2 Hz, 1H), 7.49 (dd, *J* = 7.8, 0.8 Hz, 1H), 7.41 (tt, *J* = 7.4, 0.9 Hz, 1H), 7.28 – 7.18 (m, 3H), 7.07 (dd, *J* = 8.6, 1.6 Hz, 2H), 4.35 – 4.13 (m, 2H), 3.72 (m, 1H), 3.58 (d, *J* = 4.6 Hz, 1H), 2.99 (dd, *J* = 15.9, 6.0 Hz, 1H), 2.83 (dd, *J* = 15.9, 7.1 Hz, 1H). ^{13}C-NMR (75 MHz, $CDCl_3$): δ = 204.0 (C_q), 170.0 (C_q), 154.4 (C_q), 138.3 (C_q), 136.0 (C_q), 135.5 (CH), 129.1 (CH), 128.8 (CH), 128.4 (CH), 127.6 (CH), 125.1 (CH), 124.7 (CH), 121.0 (q, J_{CF} = 269 Hz, C_q), 60.6 (CH), 60.5 (q, J_{CF} = 37.0 Hz, CH_2), 44.3 (CH), 38.6 (CH_2). ^{19}F-NMR (376 MHz, $CDCl_3$): δ = -73.7 (t, *J* = 8.3 Hz, CF_3). MS (ESI) *m/z* (relative intensity): 371.3 (100) $[M+Na]^+$. HR-MS (ESI) *m/z* calcd for $C_{19}H_{15}O_3F_3Na_1$ $[M+Na]^+$ 371.0866, found 371.0876.

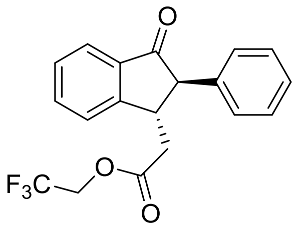

2,2,2-Trifluoroethyl 10-oxo-4b,5,9b,10-tetrahydroindeno[2,1-*a*]indene-5-carboxylate (7e). M.p. = 78-80 °C. R_f = 0.35 (pet.ether/EtOAc: 9/1). ^{1}H-NMR (400

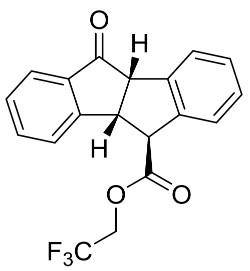

MHz, $CDCl_3$): δ = 7.73 (d, J = 7.4 Hz, 1H), 7.69 – 7.61 (m, 3H), 7.42 (tt, J = 7.3, 1.1 Hz, 1H), 7.37 – 7.27 (m, 3H), 4.63 (dd, J = 6.6, 2.5 Hz, 1H), 4.59 (q, J = 8.2 Hz, 2H), 4.43 (d, J = 6.8 Hz, 1H), 4.36 (bs, 1H). ^{13}C-NMR (75 MHz, $CDCl_3$): δ = 203.7 (C_q), 171.5 (C_q), 155.7 (C_q), 139.5 (C_q), 138.2 (C_q), 135.9 (C_q), 135.7 (CH), 129.2 (CH), 128.8 (CH), 128.7 (CH), 126.1 (CH), 125.9 (CH), 125.5 (CH), 124.7 (CH), 121.2 (q, J_{CF} = 277 Hz, C_q), 61.3 (q, J = 36.9 Hz, CH_2), 58.4 (CH), 56.0 (CH), 45.9 (CH). ^{19}F-NMR (376 MHz, $CDCl_3$): δ = -73.6 (t, J = 8.3 Hz, CF_3). MS (ESI) *m/z* (relative intensity): 369.3 (20) $[M+Na]^+$. HR-MS (ESI) *m/z* calcd for $C_{19}H_{17}O_3F_3N_1$ $[M+NH_4]^+$ 364.1155, found 364.1157.

Methyl 2-(6-methoxy-3-oxo-2-phenyl-2,3-dihydro-1*H*-inden-1-yl)acetate (6f): Representative procedure A was followed using ketone **4b** (113 mg, 0.50 mmol, 1.00 equiv) and methyl acrylate **5a** (90 µL, 1.00 mmol, 2.00 equiv) for 8 h. Purification by column chromatography (pet.ether/EtOAc: 9/1 to 5/1) gave **6f** (111 mg, 0.36 mmol, 72%) as yellow solid (dr = 10:1). R_f = 0.3 (pet.ether/EtOAc: 5/1). ^{1}H-NMR (400 MHz, $CDCl_3$): δ = 7.72 (d, J = 8.4 Hz, 1H), 7.28 – 7.18 (m, 3H), 7.09 (dd, J = 8.4, 1.4 Hz, 2H), 6.95 (dd, J = 2.3, 0.7, 1H), 6.93 (bs, 1H), 3.85 (s, 3H), 3.76 (m, 1H), 3.59 (d, J = 4.2 Hz, 1H), 3.54 (s, 3H), 2.85 (dd, J = 15.6, 6.4 Hz, 1H), 2.71 (dd, J = 15.6, 7.8 Hz, 1H). ^{13}C-NMR (101 MHz, $CDCl_3$): δ = 202.8 (C_q), 172.0 (C_q), 165.9 (C_q), 158.2 (C_q), 139.1 (C_q), 130.3 (C_q), 128.9 (CH), 128.3 (CH), 127.3 (CH), 126.3 (CH), 116.1 (CH), 108.9 (CH), 60.9 (CH), 55.9 (OCH_3), 51.9 (OCH_3), 44.6 (CH), 39.4 (CH_2). MS (ESI) *m/z* (relative intensity): 333.3 (100) $[M+Na]^+$. HR-MS (ESI) *m/z* calcd for $C_{19}H_{19}O_4$ $[M+H]^+$ 311.1283, found 311.1280.

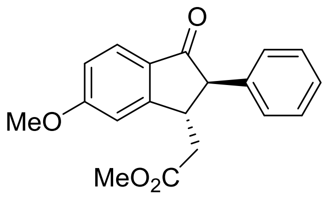

Isobutyl 2-(6-methoxy-3-oxo-2-phenyl-2,3-dihydro-1*H*-inden-1-yl)acetate (6g): Representative procedure A was followed using ketone **4b** (113 mg, 0.50 mmol, 1.00 equiv) and isobutyl acrylate **5d** (108 µL, 0.75 mmol, 1.50 equiv) for 8 h. Purification by column chromatography (pet.ether/EtOAc: 9/1) gave **6g** (125 mg, 0.36 mmol, 71%) as a yellow oil (dr = 15:1). R_f = 0.31 (pet.ether/EtOAc: 9/1). ^{1}H-NMR (500 MHz, $CDCl_3$): δ = 7.76 (d, J = 9.1 Hz, 1H), 7.32 – 7.24 (m, 3H), 7.14 (dd, J = 8.4, 1.6 Hz, 2H), 6.99 – 6.97 (m, 2H), 3.91 (s, 3H), 3.84 – 3.74 (m, 3H), 3.63 (d, J = 4.5 Hz, 1H), 2.91 (dd, J = 15.5, 6.2 Hz, 1H), 2.77 (dd, J = 15.5, 7.6 Hz, 1H), 1.82 – 1.73 (septet, 1H), 0.84 (d, J = 3.6 Hz, 3H), 0.82 (d, J = 3.6 Hz, 3H). ^{13}C-NMR (125 MHz, $CDCl_3$): δ = 202.8 (C_q), 171.7 (C_q), 165.9 (C_q), 158.4 (C_q), 139.2 (C_q), 129.3 (C_q), 129.0 (CH), 128.3 (CH), 127.3 (CH), 126.3 (CH), 116.2 (CH), 108.9 (CH), 71.1 (CH_2), 60.8 (CH), 55.9 (OCH_3), 44.6 (CH), 39.7 (CH_2), 27.7 (CH), 19.2 (CH_3), 19.1 (CH_3). MS (ESI) *m/z* (relative intensity): 375.4 (100) $[M+Na]^+$. HR-MS (ESI) *m/z* calcd for $C_{22}H_{24}O_4Na_1$ $[M+Na]^+$ 375.1567, found 375.1573.

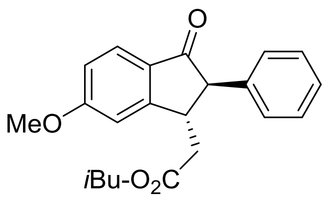

Methyl 2-(6-chloro-3-oxo-2-phenyl-2,3-dihydro-1*H*-inden-1-yl)acetate (6h): Representative procedure A was followed using ketone **4c** (115 mg, 0.50 mmol, 1.00 equiv) and methyl

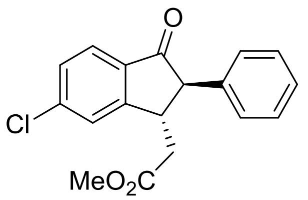

acrylate **5a** (90 µL, 1.00 mmol, 2.00 equiv) for 14 h. Purification by column chromatography (pet.ether/EtOAc: 9/1) gave **6h** (97 mg, 0.31 mmol, 62%) as a yellow oil (dr =10:1). R_f = 0.32 (pet.ether/EtOAc: 9/1). ^{1}H-NMR (500 MHz, $CDCl_3$): *δ* = 7.67 (d, *J* = 8.0 Hz, 1H), 7.47 (bs, 1H), 7.36 (ddd, *J* = 8.2, 1.4, 0.6 Hz, 1H), 7.25 – 7.18 (m, 3H), 7.05 (dd, *J* = 8.5, 1.4, 2H), 3.76 (m, 1H), 3.58 (d, *J* = 4.3 Hz, 1H), 3.52 (s, 3H), 2.82 (dd, *J* = 15.8, 6.4 Hz, 1H), 2.73 (dd, *J* = 15.8, 7.2 Hz, 1H). ^{13}C-NMR (125 MHz, $CDCl_3$): *δ* = 203.1 (C_q), 171.7 (C_q), 156.7 (C_q), 142.0 (C_q), 138.1 (C_q), 134.5 (C_q), 129.3 (CH), 129.0 (CH), 128.3 (CH), 127.6 (CH), 125.7 (CH), 125.6 (CH), 60.7 (CH), 52.2 (OCH_3), 44.2 (CH), 38.7 (CH_2). MS (ESI) *m/z* (relative intensity): 337.3 (100) $[M+Na]^+$. HR-MS (ESI) *m/z* calcd for $C_{18}H_{15}O_3Cl_1Na_1$ $[M+Na]^+$ 337.0607, found 337.0610.

5-Chloro-2-phenyl-3-((phenylsulfonyl)methyl)-2,3-dihydro-1*H*-inden-1-one (6i): Representative procedure A was followed using ketone **4c** (115 mg, 0.50 mmol, 1.00 equiv) and phenyl vinyl sulfone **5f** (126 mg, 0.75 mmol, 1.50 equiv) for 14 h. Purification by column chromatography (pet.ether/EtOAc: 9/1 to 3/1 to 2/1) gave **6i** (129 mg, 0.33 mmol, 65%) as a yellow solid (dr = 10:1). R_f = 0.2 (pet.ether/EtOAc: 9/1). ^{1}H-NMR (400 MHz, $CDCl_3$): *δ* = 7.74 (d, *J* = 8.2 Hz, 1H), 7.71 (dd, *J* = 8.4, 1.2 Hz, 2H), 7.64 – 7.59 (m, 3H), 7.48 – 7.43 (m, 3H), 7.28 – 7.26 (m, 3H), 7.08 – 7.04 (m, 2H), 3.98 (d, *J* = 3.6 Hz, 1H), 3.87 (m, 1H), 3.67 (dd, *J* = 14.7, 4.4 Hz, 1H), 3.48 (dd, *J* = 14.7, 8.4 Hz, 1H). ^{13}C-NMR (101 MHz, $CDCl_3$): *δ* = 202.2 (C_q), 155.1 (C_q), 142.3 (C_q), 137.7 (C_q), 134.2 (C_q), 129.9 (C_q), 129.5 (CH), 129.1 (CH), 128.2 (CH), 128.1 (CH), 127.7 (CH), 126.3 (CH), 125.9 (CH), 60.8 (CH_2), 59.5 (CH), 42.5 (CH). MS (ESI) *m/z* (relative intensity): 419.3 (100) $[M+Na]^+$. HR-MS (ESI) *m/z* calcd for $C_{22}H_{17}O_3Cl_1Na_1S_1$ $[M+Na]^+$ 419.0479, found 419.0492.

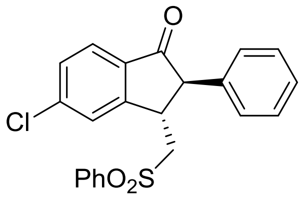

Methyl 2-(5-methyl-3-oxo-2-phenyl-2,3-dihydro-1*H*-inden-1-yl)acetate (6j): Representative procedure A was followed using ketone **4d** (105 mg, 0.50 mmol, 1.00 equiv) and methyl acrylate **5a** (90 µL, 1.00 mmol, 2.00 equiv) for 16 h. Purification by column chromatography (pet.ether/EtOAc: 9/1) gave **6j** (101 mg, 0.35 mmol, 69%) as a yellow solid and as a as a mixture of diastereomers (dr > 20:1). R_f = 0.27 (pet.ether/EtOAc: 9/1). ^{1}H-NMR (400 MHz, $CDCl_3$): *δ* = 7.62 (s, 1H), 7.49 (dd, *J* = 7.9, 1.5 Hz, 1H), 7.42 (d, *J* = 7.9 Hz, 1H), 7.33 – 7.30 (m, 2H), 7.27 (dt, *J* = 7.2, 1.4 Hz, 1H), 7.13 (dd, *J* = 8.4, 1.6 Hz, 2H), 3.84 (m, 1H), 3.64 (d, *J* = 4.4 Hz, 1H), 3.58 (s, 3H), 2.90 (dd, *J* = 15.5, 6.5 Hz, 1H), 2.76 (dd, *J* = 15.5, 7.6 Hz, 1H), 2.44 (s, 3H). ^{13}C-NMR (101 MHz, $CDCl_3$): *δ* = 204.7 (C_q), 172.1 (C_q), 152.7 (C_q), 138.8 (C_q), 138.7 (C_q), 136.7 (C_q), 136.2 (CH), 129.0 (CH), 128.4 (CH), 127.4 (CH), 125.0 (CH), 124.5 (CH), 61.0 (CH), 51.9 (OCH_3), 44.3 (CH), 39.4 (CH_2), 21.3 (CH_3). MS (ESI) *m/z* (relative intensity): 317.2 (100) $[M+Na]^+$. HR-MS (ESI) *m/z* calcd for $C_{19}H_{18}O_3Na_1$ $[M+Na]^+$ 317.1148, found 317.1144.

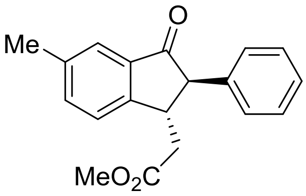

Methyl 2-(7-fluoro-3-oxo-2-phenyl-2,3-dihydro-1*H*-inden-1-yl)acetate (6k): Representative procedure A was followed using ketone **4e** (107 mg, 0.50 mmol, 1.00 equiv) and methyl acrylate **5a** (90 μL, 1.00 mmol, 2.00 equiv) for 16 h. Purification by column chromatography (pet.ether/EtOAc: 9/1) gave **6k** (80 mg, 0.27 mmol, 54%) as a yellow oil (dr = 16:1). R_f = 0.27 (pet.ether/EtOAc: 9/1). ^{1}H-NMR (400 MHz, $CDCl_3$): δ = 7.57 (d, J = 7.6 Hz, 1H), 7.41 (dt, J = 8.0, 4.5 Hz, 1H), 7.30 – 7. 20 (m, 4H), 7.07 (dd, J =8.6, 1.7, 2H), 3.92 (m, 1H), 3.68 (d, J = 3.9 Hz, 1H), 3.49 (s, 3H), 3.08 (dd, J = 15.7, 4.4 Hz, 1H), 2.73 (dd, J = 15.7, 8.5 Hz, 1H). ^{13}C-NMR (101 MHz, $CDCl_3$): δ = 203.6 (d, J_{CF} = 2.1 Hz, C_q), 171.6 (C_q), 160.2 (d, J_{CF} = 251.3 Hz, C_q), 140.5 (d, J_{CF} = 17.2 Hz, C_q), 139.2 (d, J_{CF} = 4.5 Hz, C_q), 138.4 (C_q), 130.7 (d, J_{CF} = 6.2 Hz, CH), 129.0 (CH), 128.1 (CH), 127.5 (CH), 121.8 (d, J_{CF} = 20.8 Hz, CH), 120.4 (d, J_{CF} = 3.9 Hz, CH), 60.4 (CH), 51.8 (s, OCH_3), 42.2 (d, J_{CF} = 1.5 Hz, CH), 37.7 (d, J_{CF} = 2.2 Hz, CH_2). ^{19}F-NMR (376 MHz, $CDCl_3$) δ = (-117.8 – -117.9) (m). MS (ESI) *m/z* (relative intensity): 321.2 (40) $[M+Na]^+$. HR-MS (ESI) *m/z* calcd for $C_{18}H_{15}O_3F_1Na_1$ $[M+Na]^+$ 321.0897, found 321.0908.

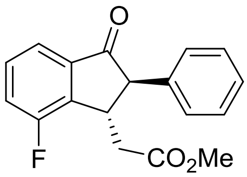

Methyl 2-(7-methoxy-3-oxo-2-phenyl-2,3-dihydro-1*H*-inden-1-yl)acetate (6l): Representative procedure A was followed using ketone **4f** (113 mg, 0.50 mmol, 1.00 equiv) and methyl acrylate **5a** (90 μL, 1.00 mmol, 2.00 equiv) for 14 h. Careful purification by column chromatography (pet.ether/EtOAc: 12/1) gave **6l** (33 mg, 0.106 mmol, 21%), dr = 10:1, and **6'l** (22 mg, 0.071 mmol, 14%, containing some **6l**) as yellow oils. **6l**: R_f = 0.20 (pet.ether/EtOAc: 9/1). ^{1}H-NMR (500 MHz, $CDCl_3$) of compound **6l** : δ = 7.43 (dd, J = 8.2, 0.9 Hz, 1H), 7.33 – 7.30 (m, 2H), 7.28 – 7.24 (m, 3H), 7.14 – 7.12 (m, 2H), 3.86 (s, 3H), 3.82 – 3.81 (m, 1H), 3.64 (d, J = 4.2 Hz, 1H), 3.58 (s, 3H), 2.88 (dd, J = 15.3, 6.3 Hz, 1H), 2.75 (dd, J = 15.3, 7.5 Hz, 1H). ^{13}C-NMR (125 MHz, $CDCl_3$): δ = 204.6 (C_q), 172.1 (C_q), 160.2 (C_q), 148.1 (C_q), 138.7 (C_q), 137.3 (C_q), 129.0 (CH), 128.3 (CH), 127.4 (CH), 126.1 (CH), 124.7 (CH), 105.6 (CH), 61.3 (CH), 55.8 (OCH_3), 51.9 (OCH_3), 44.1 (CH), 39.4 (CH_2).

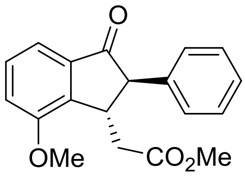

Methyl 2-(5-methoxy-3-oxo-2-phenyl-2,3-dihydro-1H-inden-1-yl)acetate (6'l): Due to overlapping aromatic signals from **6l**, only characteristic ^{1}H NMR data is reported: ^{1}H-NMR (500 MHz, $CDCl_3$): δ = 3.92 (m, 1H), 3.92 (s, 3H), 3.68 (d, J = 3.2 Hz, 1H), 3.55 (s, 3H), 3.26 (dd, J = 15.6, 4.1 Hz, 1H), 2.63 (dd, J = 15.6, 9.3 Hz, 1H). MS (ESI) *m/z* (relative intensity): 311.2 (100) $[M+H]^+$. HR-MS (EI) *m/z* calcd for $C_{19}H_{18}O_4$ $[M^+]$ 310.1200, found 310.1186.

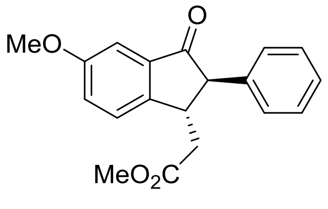

Methyl 2-(4-methoxy-3-oxo-2-(*p*-tolyl)-2,3-dihydro-1*H*-inden-1-yl)acetate (6m): Representative procedure A was followed using ketone **4g** (120 mg, 0.50 mmol, 1.00 equiv) and methyl acrylate **5a** (90 μL, 1.00 mmol, 2.00 equiv) for 14 h. Purification by column chromatography (pet.ether/EtOAc: 9/1 to 3/1 to 2/1) gave **6m** (104 mg,

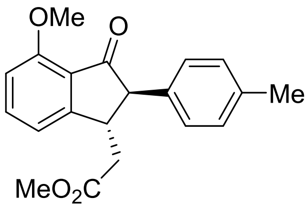

0.32 mmol, 64%) as a yellow solid (dr = 10:1). R_f = 0.22 (pet.ether/EtOAc: 9/1). ^{1}H-NMR (400 MHz, $CDCl_3$): δ = 7.59 (t, J = 7.9 Hz, 1H), 7.10 (d, J = 8.2 Hz, 2H), 7.04 (d, J = 7.8 Hz, 1H), 7.02 (d, J = 8.2 Hz, 2H), 6.87 (d, J = 8.4 Hz, 1H), 3.94 (s, 3H), 3.77 (m, 1H), 3.59 (s, 3H), 3.58 (d, J = 4.3 Hz, 1H), 2.87 (dd, J = 15.6, 6.1 Hz, 1H), 2.72 (dd, J = 15.6, 7.6 Hz, 1H), 2.3 (s, 3H). ^{13}C-NMR (101 MHz, $CDCl_3$): δ = 202.3 (C_q), 172.1 (C_q), 158.3 (C_q), 157.7 (C_q), 137.0 (C_q), 136.7 (C_q), 135.9 (C_q), 129.5 (CH), 128.2 (CH), 124.0 (CH), 116.8 (CH), 110.0 (CH), 60.5 (CH), 55.9 (OCH_3), 51.9 (OCH_3), 44.1 (CH), 39.3 (CH_2), 21.2 (CH_3). MS (ESI) *m/z* (relative intensity): 347.4 (100) $[M+Na]^+$. HR-MS (ESI) *m/z* calcd for $C_{20}H_{21}O_4$ $[M+H]^+$ 325.1440, found 325.1430.

Ethyl 2-(4-methoxy-3-oxo-2-(*p*-tolyl)-2,3-dihydro-1*H*-inden-1-yl)acetate (6n): Representative procedure

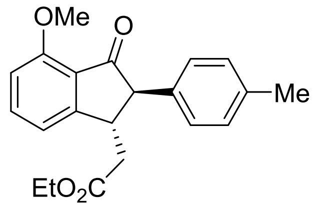

A was followed using ketone **4g** (120 mg, 0.50 mmol, 1.00 equiv) and ethyl acrylate **5b** (109 µL, 1.00 mmol, 2.00 equiv) for 14 h. Purification by column chromatography (pet.ether/EtOAc: 9/1 to 3/1) gave **6n** (98 mg, 0.29 mmol, 58%) as a yellow oil (dr = 10:1). R_f = 0.24 (pet.ether/EtOAc: 9/1). ^{1}H-NMR (400 MHz, $CDCl_3$): δ = 7.59 (t, J = 8.2 Hz, 1H), 7.10 (d, J = 8.2 Hz, 2H), 7.05 (d, J = 7.7 Hz, 1H), 7.03 (d, J = 8.2 Hz, 2H), 6.86 (d, J = 8.2 Hz, 1H), 4.04 (m, 2H), 3.94 (s, 3H), 3.75 (m, 1H), 3.59 (d, J = 4.2 Hz, 1H), 2.86 (dd, J = 15.5, 6.0 Hz, 1H), 2.71 (dd, J = 15.5, 7.8 Hz, 1H), 2.30 (s, 3H), 1.13 (t, J = 7.1 Hz, 3H).^{13}C-NMR (101 MHz, $CDCl_3$): δ = 202.4 (C_q), 171.6 (C_q), 158.3 (C_q), 157.9 (C_q), 137.0 (C_q), 136.7 (C_q), 136.0 (C_q), 129.5 (CH), 128.2 (CH), 116.9 (CH), 109.9 (CH), 60.9 (CH_2), 60.5 (CH), 55.9 (OCH_3), 44.2 (CH), 39.7 (CH_2), 21.2 (CH_3), 14.1 (CH_3). MS (ESI) *m/z* (relative intensity): 339.3 (100) $[M+H]^+$. HR-MS (ESI) *m/z* calcd for $C_{21}H_{23}O_4$ $[M+H]^+$ 339.1591, found 339.1594.

Methyl 2-(4-methoxy-3-oxo-2-phenyl-2,3-dihydro-1*H*-inden-1-yl)acetate (6o): Representative procedure

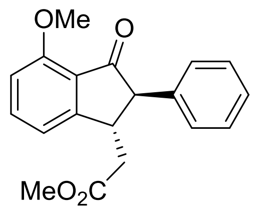

A was followed using ketone **4k** (113 mg, 0.50 mmol, 1.00 equiv) and methyl acrylate **5a** (90 µL, 1.00 mmol, 2.00 equiv) for 16 h. Purification by column chromatography (pet.ether/EtOAc: 9/1 to 4/1) gave **6o** (90 mg, 0.29 mmol, 58%) as a yellow oil (single diastereomer), and **7o** as a yellow oil (14 mg, 0.03 mmol, 9%) (single diastereomer). **6o**: R_f = 0.30 (pet.ether/EtOAc: 4/1). ^{1}H-NMR (300 MHz, $CDCl_3$): δ = 7.57 (t, J = 8.0 Hz, 1H), 7.29 – 7.20 (m, 3H), 7.13 – 7.10 (m, 2H), 7.04 (d, J = 7.5 Hz, 1H), 6.85 (d, J = 8.5 Hz, 1H), 3.91 (s, 3H), 3.77 (m, 1H), 3.59 (d, J = 4.4 Hz, 1H), 3.55 (s, 3H), 2.86 (dd, J = 15.4, 6.0 Hz, 1H), 2.70 (dd, J = 15.4, 7.9 Hz, 1H). ^{13}C-NMR (75 MHz, $CDCl_3$): δ = 202.0 (C_q), 172.0 (C_q), 158.4 (C_q), 157.8 (C_q), 139.0 (C_q), 137.1 (CH), 128.8 (CH), 128.3 (CH), 127.1 (CH), 124.0 (C_q), 116.9 (CH), 110.1 (CH), 60.8 (CH), 55.9 (OCH_3), 51.8 (OCH_3), 44.1 (CH), 39.5 (CH_2). MS (ESI) *m/z* (relative intensity): 311.2 (100) $[M+H]^+$, 333.2 $[M+Na]^+$. HR-MS (EI) *m/z* calcd for $C_{19}H_{18}O_4$ $[M^+]$ 310.1200, found 310.1188.

Methyl 1-methoxy-10-oxo-4b,5,9b,10-tetrahydroindeno[2,1-*a*]indene-5-carboxylate (7o). R_f = 0.32 (pet.ether/EtOAc: 4/1). ^{1}H-NMR (300 MHz, $CDCl_3$): δ = 7.68 (d, J = 7.6 Hz, 1H),

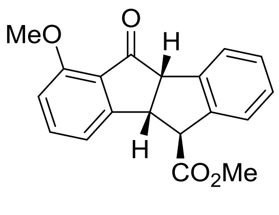

7.57 (t, J = 7.8 Hz, 1H), 7.35 (dd, J = 6.9, 1.2 Hz, 1H), 7.27 – 7.22 (m, 2H), 7.14 (d, J = 7.6 Hz, 1H), 6.79 (d, J = 8.4 Hz, 1H), 4.54 (dd, J = 7.0, 2.4 Hz, 1H), 4.36 (d, J = 6.9 Hz, 1H), 4.24 (brs, 1H), 3.90 (s, 3H), 3.79 (s, 3H). ^{13}C-NMR (75 MHz, $CDCl_3$): δ = 202.0 (C_q), 173.5 (C_q), 159.0 (C_q), 158.7 (C_q), 139.9 (C_q), 139.2 (C_q), 137.3 (CH), 128.7 (CH), 128.4 (CH), 125.8 (CH), 125.5 (CH), 123.8 (C_q), 117.8 (CH), 110.0 (CH), 58.7 (CH), 56.7 (OCH_3), 55.9 (OCH_3), 52.8 (CH), 45.6 (CH). MS (ESI) *m/z* (relative intensity): 309.2 (100) $[M+H]^+$, 331.3 (80) $[M+Na]^+$. HR-MS (EI) *m/z* calcd for $C_{19}H_{16}O_4$ $[M^+]$ 308.1049, found 308.1052.

Methyl 2-(2-(2,4-dimethoxyphenyl)-4-methoxy-3-oxo-2,3-dihydro-1*H*-inden-1-yl)acetate (6p): Representative procedure A was followed using ketone **4l** (143 mg, 0.50 mmol, 1.00 equiv) and methyl acrylate **5a** (90 µL, 1.00 mmol, 2.00 equiv) for 8 h. Purification by column chromatography (pet.ether/EtOAc: 9/1 to 3/1 to 1/1) gave **6p** (98 mg, 0.27 mmol, 53%) as an orange oil (single diastereomer). R_f = 0.22 (pet.ether/EtOAc: 5/1). ^{1}H-NMR (500 MHz, $CDCl_3$): δ = 7.55 (t, J = 7.9 Hz, 1H), 7.00 (t, J = 7.7 Hz, 2H), 6.85 (d, J = 8.1 Hz, 1H), 6.43 (dd, J = 9.1, 2.4 Hz, 1H), 6.41 (s, 1H), 3.94 (s, 3H), 3.77 (s, 3H), 3.75 (m, 1H), 3.60 (s, 3H), 3.55 (s, 3H), 3.54 (d, J = 5.5 Hz, 1H), 2.85 (dd, J = 15.4, 6.4 Hz, 1H), 2.74 (dd, J = 15.4, 7.2 Hz, 1H). ^{13}C-NMR (125 MHz, $CDCl_3$): δ = 202.7 (C_q), 172.4 (C_q), 160.4 (C_q), 158.2 (C_q), 157.9 (C_q), 157.2 (C_q), 136.4 (C_q), 131.7 (C_q), 124.4 (CH), 119.8 (CH), 116.5 (CH), 109.6 (CH), 104.2 (CH), 99.1 (CH), 57.5 (CH), 55.9 (OCH_3), 55.6 (OCH_3), 55.5 (OCH_3), 51.7 (OCH_3), 42.5 (CH), 38.5 (CH_2). MS (ESI) *m/z* (relative intensity): 393.3 (100) $[M+Na]^+$. HR-MS (ESI) *m/z* calcd for $C_{21}H_{23}O_6$ $[M+H]^+$ 371.1489, found 371.1488.

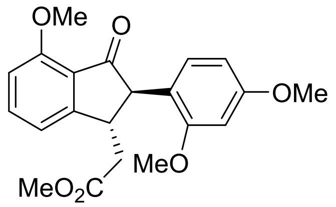

Methyl 2-(4-fluoro-3-oxo-2-phenyl-2,3-dihydro-1*H*-inden-1-yl)acetate (6q): Representative procedure A was followed using ketone **4h** (107 mg, 0.50 mmol, 1.00 equiv) and methyl acrylate **5a** (90 µL, 1.00 mmol, 2.00 equiv) for 14 h. Purification by column chromatography (pet.ether/EtOAc: 9/1 to 3/1) gave **6q** (107 mg, 0.36 mmol, 72%) as a yellow oil (dr = 15:1). R_f = 0.37 (pet.ether/EtOAc: 3/1). ^{1}H-NMR (400 MHz, $CDCl_3$): δ = 7.57 (dt, J = 8.0, 5.2 Hz, 1H), 7.27 – 7. 18 (m, 4H), 7.07 (dd, J =8.4, 1.6, 2H), 6.99 (t, J = 8.6 Hz, 1H), 3.80 (m, 1H), 3.61 (d, J = 4.7 Hz, 1H), 3.53 (s, 3H), 2.84 (dd, J = 15.7, 6.0 Hz, 1H), 2.72 (dd, J = 15.7, 7.3 Hz, 1H). ^{13}C-NMR (101 MHz, $CDCl_3$): δ = 200.6 (d, J_{CF} = 1.8 Hz, C_q), 171.7 (C_q), 159.0 (d, J_{CF} = 265.9 Hz, C_q), 157.1 (d, J_{CF} = 2.1 Hz, C_q), 138.0 (C_q), 137.3 (d, J_{CF} = 8.3 Hz, C_q), 129.0 (CH), 128.4 (CH), 127.5 (CH), 126.6 (d, J_{CF} = 54.5, CH), 123.8 (d, J_{CF} = 12.9 Hz, CH), 121.0 (d, J_{CF} = 4.2 Hz, CH), 115.4 (d, J_{CF} = 19.1 Hz, CH), 60.9 (CH), 51.9 (s, OCH_3), 44.3 (d, J_{CF} = 1.4 Hz, CH), 38.8 (CH_2). ^{19}F-NMR (376 MHz, $CDCl_3$) δ = (-113.3 – -113.4) (m). MS (ESI) *m/z* (relative intensity): 321.3 (100) $[M+Na]^+$. HR-MS (ESI) *m/z* calcd for $C_{18}H_{15}O_3F_1Na_1$ $[M+Na]^+$ 321.0897, found 321.0899.

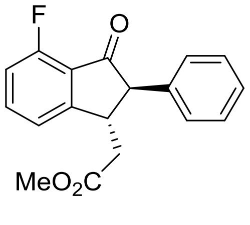

Methyl 2-(2-(4-fluorophenyl)-4-methyl-3-oxo-2,3-dihydro-1*H*-inden-1-yl)acetate (6r): Representative procedure A was followed using ketone **4i** (114 mg, 0.50 mmol, 1.00 equiv) and

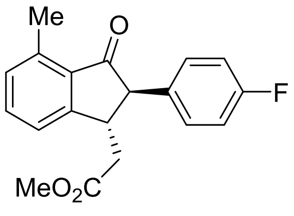

methyl acrylate **5a** (90 μL, 1.00 mmol, 2.00 equiv) for 14 h. Careful purification by column chromatography (pet.ether/EtOAc: 9/1) gave **6r** (47 mg, 0.15 mmol, 30%) as a light yellow solid (dr =10:1). R_f = 0.45 (pet.ether/EtOAc: 9/1). ^{1}H-NMR (500 MHz, $CDCl_3$): δ = 7.52 (t, J = 7.7 Hz, 1H), 7.34 (d, J = 7.7 Hz, 1H), 7.21 (dd, J = 7.5, 0.8 Hz, 1H), 7.13 – 7.10 (m, 2H), 7.03 – 6.99 (m, 2H), 3.81 – 3.79 (m, 1H), 3.59 (d, J = 4.8 Hz, 1H), 3.57 (s, 3H), 2.93 (dd, J = 15.3, 5.8 Hz, 1H), 2.71 (dd, J = 15.3, 8.0 Hz, 1H), 2.64 (s, 3H). ^{13}C-NMR (125 MHz, $CDCl_3$): δ = 205.1 (C_q), 172.1 (C_q), 162.1 (d, J_{CF} = 245.7 Hz, C_q), 155.7 (C_q), 139.8 (C_q), 134.7 (C_q), 134.6 (d, J_{CF} = 3.0 Hz, C_q), 133.1 (CH), 130.5 (CH), 130.0 (d, J_{CF} = 8.0 Hz, CH), 122.4 (CH), 115.7 (d, J_{CF} = 21.5 Hz, CH), 60.2 (CH), 51.9 (OCH_3), 43.8 (CH), 39.4 (CH_2), 18.6 (CH_3). ^{19}F-NMR (376 MHz, $CDCl_3$) δ = (-115.5 – -115.6) (m). MS (ESI) *m/z* (relative intensity): 313.3 (30) $[M+H]^+$, 335.3 (50) $[M+Na]^+$. HR-MS (ESI) *m/z* calcd for $C_{19}H_{18}O_3F_1$ $[M+H]^+$ 313.1240, found 313.1240.

Methyl 2-(2-([1,1'-biphenyl]-4-yl)-4-methyl-3-oxo-2,3-dihydro-1H-inden-1-yl)acetate (6s):

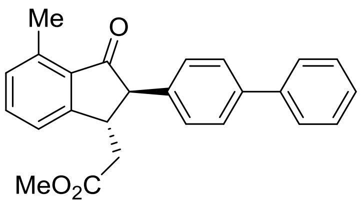

Representative procedure A was followed using ketone **4j** (144 mg, 0.50 mmol, 1.00 equiv) and methyl acrylate **5a** (90 μL, 1.00 mmol, 2.00 equiv) for 24 h. Purification by column chromatography (pet.ether/EtOAc: 9/1) gave **6s** (37 mg, 0.1 mmol, 20%) as a yellow oil (dr =14:1). R_f = 0.18 (pet.ether/EtOAc: 9/1). ~15% unreacted starting material also recovered. ^{1}H-NMR (300 MHz, $CDCl_3$): δ = 7.50 – 7.45 (m, 5H), 7.37 – 7.25 (m, 4H), 7.18 – 7.12 (m, 3H), 3.82 (m, 1H), 3.59 (d, J = 4.5 Hz, 1H), 3.51 (s, 3H), 2.87 (dd, J = 15.5, 5.9 Hz, 1H), 2.69 (dd, J = 15.5, 7.7 Hz, 1H), 2.58 (s, 3H). ^{13}C-NMR (75 MHz, $CDCl_3$): δ = 205.3 (C_q), 172.1 (C_q), 155.9 (C_q), 141.0 (C_q), 140.2 (C_q), 139.8 (C_q), 137.9 (C_q), 133.4 (C_q), 130.4 (CH), 128.8 (CH), 127.7 (CH), 127.3 (CH), 127.2 (CH), 127.1 (CH), 122.5 (CH), 60.7 (CH), 51.9 (OCH_3), 43.7 (CH), 39.5 (CH_2), 18.6 (CH_3). MS (ESI) *m/z* (relative intensity): 393.4 (100) $[M+Na]^+$. HR-MS (ESI) *m/z* calcd for $C_{25}H_{22}O_3Na_1$ $[M+Na]^+$ 393.1461, found 393.1462.

Methyl 2-(2-(2-methoxyphenyl)-3-oxo-2,3-dihydro-1*H*-inden-1-yl)acetate (6t): Representative procedure

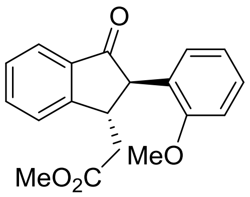

A was followed using ketone **4m** (452 mg, 2.00 mmol, 1.00 equiv) and methyl acrylate **5a** (360 μL, 4.00 mmol, 2.00 equiv) for 8 h. Purification by column chromatography (pet.ether/EtOAc: 9/1 to 5/1) gave **6t** (378 mg, 1.22 mmol, 61%) as a yellow solid (dr >20:1). R_f = 0.18 (pet.ether/EtOAc: 9/1). ^{1}H-NMR (400 MHz, $CDCl_3$): δ = 7.82 (d, J = 7.5 Hz, 1H), 7.63 (dt, J = 7.6, 1.2 Hz, 1H), 7.50 (dd, J = 7.8, 0.7 Hz, 1H), 7.44 (tt, J = 7.4, 0.7 Hz, 1H), 7.26 (dt, J = 8.6, 1.7 Hz, 1H), 7.12 (dd, J = 7.4, 1.7 Hz, 1H), 6.92 (dt, J = 7.4, 1.0 Hz, 1H), 6.85 (d, J = 8.3 Hz, 1H), 3.75 (m, 1H), 3.62 (d, J = 5.4 Hz, 1H), 3.58 (s, 3H), 3.55 (s, 3H), 2.91 (dd, J = 15.5, 6.4 Hz, 1H), 2.79 (dd, J = 15.5, 7.2 Hz, 1H). ^{13}C-NMR (101 MHz, $CDCl_3$): δ = 204.8 (C_q), 172.3 (C_q), 157.1 (C_q), 154.5 (C_q), 136.3 (C_q), 134.8 (C_q), 131.3 (CH), 128.8 (CH), 127.9 (CH), 127.2 (CH), 124.8 (CH), 123.8 (CH), 120.7 (CH), 111.1 (CH), 57.8 (CH), 55.3 (OCH_3), 51.7 (OCH_3), 42.9 (CH), 38.4 (CH_2). MS (ESI) *m/z* (relative intensity): 332.8 (100) $[M+Na]^+$. HR-MS (ESI) *m/z* calcd for $C_{19}H_{18}O_4Na_1$ $[M+Na]^+$ 333.1097, found 333.1102.

Methyl 2-(2-(2-nitrophenyl)-3-oxo-2,3-dihydro-1*H*-inden-1-yl)acetate (6u): Representative procedure A was followed using ketone **4n** (120 mg, 0.50 mmol, 1.00 equiv) and methyl acrylate **5a** (90 μL, 1.00 mmol, 2.00 equiv) for 24 h. ~50% conversion to desired product was observed via LC-MS analysis. Purification by column chromatography (pet.ether/EtOAc: 9/1 to 7/1) gave **6u** (57 mg, 0.18 mmol, 35%) as a yellow solid (dr >20:1). R_f = 0.19 (pet.ether/EtOAc: 9/1). ^{1}H-NMR (400 MHz, $CDCl_3$): δ = 8.10 (dd, J = 8.2, 1.2 Hz, 1H), 7.85 (d, J = 7.6 Hz, 1H), 7.69 (dt, J = 7.7, 1.2 Hz, 1H), 7.59 (dt, J = 7.4, 1.3 Hz, 1H), 7.54 – 7.46 (m, 3H), 7.27 (dd, J = 7.7, 1.1 Hz, 1H), 4.11 (d, J = 5.2 Hz, 1H), 3.99 (m, 1H), 3.54 (s, 3H), 3.02 (dd, J = 16.0, 5.9 Hz, 1H), 2.89 (dd, J = 16.0, 7.4 Hz, 1H). ^{13}C-NMR (101 MHz, $CDCl_3$): δ = 201.9 (C_q), 172.0 (C_q), 153.8 (C_q), 148.5 (C_q), 136.1 (C_q), 135.4 (C_q), 133.8 (CH), 133.5 (CH), 128.8 (CH), 128.5 (CH), 125.9 (CH), 124.9 (CH), 124.4 (CH), 58.8 (CH), 51.9 (OCH_3), 43.6 (CH), 38.0 (CH_2). MS (ESI) *m/z* (relative intensity): 348.3 (100) $[M+Na]^+$. HR-MS (ESI) *m/z* calcd for $C_{18}H_{15}O_5N_1Na_1$ $[M+Na]^+$ 348.0842, found 348.0838.

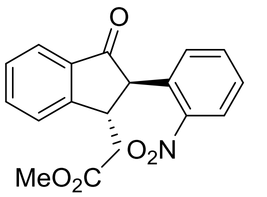

Methyl 2-(2-(4-fluorophenyl)-3-oxo-2,3-dihydro-1*H*-inden-1-yl)acetate (6v): Representative procedure A was followed using ketone **4o** (107 mg, 0.50 mmol, 1.00 equiv) and methyl acrylate **5a** (90 μL, 1.00 mmol, 2.00 equiv) for 14 h. Purification by column chromatography (pet.ether/EtOAc: 15/1 to 9/1) gave **6v** (95 mg, 0.32 mmol, 64%) as a white solid (dr =10:1). R_f = 0.32 (pet.ether/EtOAc: 9/1). ^{1}H-NMR (500 MHz, $CDCl_3$): δ = 7.82 (d, J = 7.6 Hz, 1H), 7.69 (dt, J = 7.7, 1.2 Hz, 1H), 7.54 (qd, J = 7.9, 0.9 Hz, 1H), 7.47 (tt, J = 7.4, 0.9 Hz, 1H), 7.13 – 7.10 (m, 2H), 7.03 – 7.00 (m, 2H), 3.85 – 3.82 (m, 1H), 3.64 (d, J = 4.6 Hz, 1H), 3.59 (s, 3H), 2.94 (dd, J = 15.6, 6.0 Hz, 1H), 2.75 (dd, J = 15.6, 8.0 Hz, 1H). ^{13}C-NMR (125 MHz, $CDCl_3$): δ = 204.4 (C_q), 172.0 (C_q), 162.2 (d, J_{CF} = 246.5 Hz, C_q), 155.0 (C_q), 135.8 (C_q), 135.6 (CH), 134.3 (d, J_{CF} = 3.3 Hz, C_q), 130.0 (d, J_{CF} = 8.0 Hz, CH), 128.7 (CH), 125.2 (CH), 124.6 (CH), 115.8 (d, J_{CF} = 21.3 Hz, CH), 59.9 (CH), 51.9 (OCH_3), 44.6 (CH), 39.2 (CH_2). ^{19}F-NMR (470 MHz, $CDCl_3$) δ = (-115.3 – -115.4) (m). MS (ESI) *m/z* (relative intensity): 299.1 (100) $[M+H]^+$, 321.2 (80) $[M+Na]^+$. HR-MS (EI) *m/z* calcd for $C_{18}H_{15}O_3F_1$ $[M^+]$ 298.1000, found 298.0989.

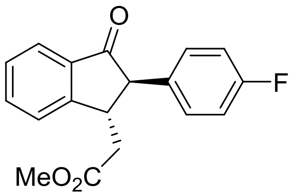

Methyl 2-(3-oxo-2-(4-(trifluoromethyl)phenyl)-2,3-dihydro-1*H*-inden-1-yl)acetate (6w): Representative procedure A was followed using ketone **4p** (132 mg, 0.50 mmol, 1.00 equiv) and methyl acrylate **5a** (90 μL, 1.00 mmol, 2.00 equiv) for 14 h. Purification by column chromatography (pet.ether/EtOAc: 15/1 to 9/1) gave **6w** (118 mg, 0.34 mmol, 68%) as a yellow oil (dr = 12:1). R_f = 0.34 (pet.ether/EtOAc: 9/1). ^{1}H-NMR (500 MHz, $CDCl_3$): δ = 7.82 (d, J = 7.8 Hz, 1H), 7.70 (dt, J = 7.7, 1.4 Hz, 1H), 7.58 (d, J = 8.2 Hz, 2H), 7.56 (d, J = 6.9 Hz, 1H), 7.49 (t, J = 7.3 Hz, 1H), 7.28 (d, J = 8.2 Hz, 2H), 3.91 – 3.87 (m, 1H), 3.74 (d, J = 4.7 Hz, 1H), 3.58 (s, 3H), 2.96 (dd, J = 15.9, 5.9 Hz, 1H), 2.76 (dd, J = 15.9, 8.0 Hz, 1H). ^{13}C-NMR (125 MHz, $CDCl_3$): δ = 203.6 (C_q), 171.8 (C_q), 155.0 (C_q), 142.7 (d, J_{CF} = 1.3

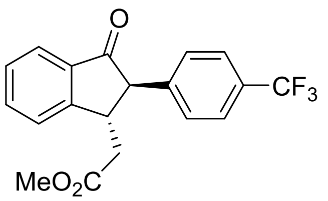

Hz, C_q), 135.8 (C_q), 135.7 (CH), 129.6 (q, J_{CF} = 97.4 Hz, C_q), 128.8 (CH), 125.9 (q, J_{CF} = 11.4 Hz, CH), 125.3 (CH), 124.7 (CH), 124.5 (q, J_{CF} = 273.1 Hz, C_q), 60.3 (CH), 51.9 (OCH_3), 44.3 (CH), 39.2 (CH_2). ^{19}F-NMR (470 MHz, $CDCl_3$) δ = -62.5 (s). MS (ESI) *m/z* (relative intensity): 349.2 (100) $[M+H]^+$, 371.3 (90) $[M+Na]^+$. HR-MS (EI) *m/z* calcd for $C_{19}H_{15}O_3F_3$ $[M^+]$ 348.0968, found 348.0953.

Ethyl 2-(3-oxo-2-(*p*-tolyl)-2,3-dihydro-1*H*-inden-1-yl)acetate (6x): Representative procedure A was followed using ketone **4q** (105 mg, 0.50 mmol, 1.00 equiv) and ethyl acrylate **5b** (109 μL, 1.00 mmol, 2.00 equiv) for 8 h. Purification by column chromatography (pet.ether/EtOAc: 9/1) gave **6x** (93 mg, 0.30 mmol, 60%) as a yellow soild (dr = 10:1). R_f = 0.22 (pet.ether/EtOAc: 9/1). ^{1}H-NMR (400 MHz, $CDCl_3$): δ = 7.81 (d, *J* = 7.7 Hz, 1H), 7.68 (dt, *J* = 7.7, 1.0 Hz, 1H), 7.56 (dd, *J* = 7.7, 0.8 Hz, 1H), 7.46 (tt, *J* = 7.6, 1.0 Hz, 1H), 7.13 (d, *J* = 7.9 Hz, 2H), 7.04 (d, *J* = 8.2 Hz, 2H), 4.03 (m, 2H), 3.87 (m, 1H), 3.64 (d, *J* = 4.4 Hz, 1H), 2.91 (dd, *J* = 15.6, 6.3 Hz, 1H), 2.78 (dd, *J* = 15.6, 7.6 Hz, 1H), 2.32 (s, 3H), 1.13 (t, *J* = 7.2 Hz, 3H). ^{13}C-NMR (101 MHz, $CDCl_3$): δ = 204.9 (C_q), 171.6 (C_q), 155.3 (C_q), 137.0 (C_q), 136.0 (C_q), 135.6 (C_q), 135.3 (CH), 129.6 (CH), 128.4 (CH), 128.3 (CH), 125.3 (CH), 124.4 (CH), 60.9 (CH_2), 60.3 (CH), 44.6 (CH), 39.4 (CH_2), 21.2 (CH_3), 14.1 (CH_3). MS (ESI) *m/z* (relative intensity): 331.2 (100) $[M+Na]^+$. HR-MS (ESI) *m/z* calcd for $C_{20}H_{20}O_3Na_1$ $[M+Na]^+$ 331.1305, found 331.1296.

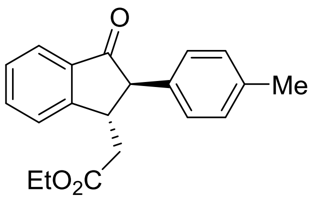

Methyl 2-(2-methyl-3-oxo-2-phenyl-2,3-dihydro-1*H*-inden-1-yl)acetate (6y): Representative procedure A is followed using ketone **4r** (105 mg, 0.50 mmol, 1.00 equiv) and methyl acrylate **5a** (90 μL, 1.00 mmol, 2.00 equiv) for 16 h. Purification by column chromatography using (pet.ether/EtOAc: 12/1) gave **6y** (72 mg, 0.25 mmol, 49%) as a colorless oil (single diastereomer), along with dicyclized product **7y** (51 mg, 0.18 mmol, 35%) as a colorless oil (single diastereomer). **6y**: R_f = 0.36 (pet.ether/EtOAc: 9/1). ^{1}H-NMR (500 MHz, $CDCl_3$): δ = 7.83 (dd, *J* = 8.4, 1.3 Hz, 1H), 7.63 (dt, *J* = 7.6, 1.2 Hz, 1H), 7.44 (d, *J* = 7.1 Hz, 2H), 7.31 – 7.28 (m, 2H), 7.26 – 7.24 (m, 2H), 7.21 (tt, *J* = 8.5, 1.4 Hz, 1H), 4.15 (t, *J* = 7.5 Hz, 1H), 3.62 (s, 3H), 2.75 (d, *J* = 7.5 Hz, 2H), 1.48 (s, 3H). ^{13}C-NMR (125 MHz, $CDCl_3$): δ = 208.4 (C_q), 172.7 (C_q), 154.5 (C_q), 143.7 (C_q), 135.5 (C_q), 134.9 (CH), 128.6 (CH), 128.4 (CH), 127.0 (CH), 126.7 (CH), 125.2 (CH), 124.7 (CH), 57.2 (C_q), 52.0 (OCH_3), 48.5 (CH), 35.9 (CH_2), 19.4 (CH_3). MS (ESI) *m/z* (relative intensity): 317.4 (100) $[M+Na]^+$. HR-MS (ESI) *m/z* calcd for $C_{19}H_{18}O_3Na_1$ $[M+Na]^+$ 317.1148, found 317.1143.

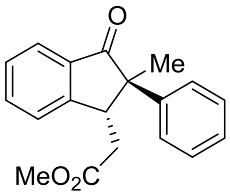

Methyl 9b-methyl-10-oxo-4b,5,9b,10-tetrahydroindeno[2,1-*a*]indene-5-carboxylate (7y): R_f = 0.41 (pet.ether/EtOAc: 9/1). ^{1}H-NMR (400 MHz, $CDCl_3$): δ = 7.71 (d, *J* = 7.8 Hz, 1H), 7.64 (dt, *J* = 7.8, 1.2, Hz, 1H), 7.58 (dd, *J* = 7.6, 0.8 Hz, 1H), 7.55 (dd, *J* = 8.0, 1.0 Hz, 1H), 7.38 (tt, *J* = 8.2, 1.0 Hz, 1H), 7.33 – 7.31 (m, 1H), 7.29 – 7.27 (m, 1H), 7.24 (dd, *J* = 7.4, 1.4 Hz, 1H), 4.22 (br, 2H), 3.82 (s, 3H), 1.71 (s, 3H). ^{13}C-NMR (101

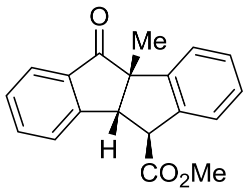

MHz, $CDCl_3$): δ = 206.5 (C_q), 173.6 (C_q), 155.0 (C_q), 144.6 (C_q), 138.7 (C_q), 135.4 (C_q), 128.9 (CH), 128.6 (CH), 128.5 (CH), 126.0 (CH), 125.8 (CH), 124.7 (CH), 124.6 (CH), 62.6 (C_q), 56.1 (CH), 53.7 (CH), 52.8 (OCH_3), 22.9 (CH_3). MS (ESI) *m/z* (relative intensity): 315.4 (100) $[M+Na]^+$. HR-MS (ESI) *m/z* calcd for $C_{19}H_{17}O_3$ $[M+H]^+$ 293.1172, found 293.1177.

Ethyl 2-(2-methyl-3-oxo-2-phenyl-2,3-dihydro-1*H*-inden-1-yl)acetate (6z): Representative procedure A

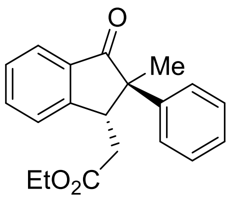

was followed using ketone **4r** (105 mg, 0.50 mmol, 1.00 equiv) and ethyl acrylate **5b** (109 μL, 1.00 mmol, 2.00 equiv) for 16 h. Purification by column chromatography (pet.ether/EtOAc: 15/1) gave **6z** (77 mg, 0.25 mmol, 50%) as a colorless oil (single diastereomer) along with dicyclized product **7z** (40 mg, 0.13 mmol, 26%) as a colorless oil (single diastereomer). **6z**: R_f = 0.44 (pet.ether/EtOAc: 9/1). ^{1}H-NMR (500 MHz, $CDCl_3$): δ = 7.82 (d, *J* = 7.8 Hz, 1H), 7.63 (dt, *J* = 7.6, 1.0 Hz, 1H), 7.45 (d, *J* = 7.9 Hz, 1H), 7.43 (d, *J* = 7.4 Hz, 1H), 7.31 – 7.24 (m, 4H), 7.21 (tt, *J* = 8.4, 1.4 Hz, 1H), 4.17 – 4.02 (m, 3H), 2.74 (dd, *J* = 7.3,1.1 Hz, 2H), 1.49 (s, 3H), 1.16 (t, *J* = 7.4 Hz, 3H). ^{13}C-NMR (125 MHz, $CDCl_3$): δ = 208.4 (C_q), 172.2 (C_q), 154.7 (C_q), 143.8 (C_q), 135.4 (C_q), 134.9 (CH), 128.6 (CH), 128.4 (CH), 127.0 (CH), 126.8 (CH), 125.3 (CH), 124.7 (CH), 61.0 (CH_2), 57.3 (C_q), 48.5 (CH), 36.2 (CH_2), 19.4 (CH_3), 14.1 (CH_3). MS (ESI) *m/z* (relative intensity): 331.5 (100) $[M+Na]^+$. HR-MS (ESI) *m/z* calcd for $C_{20}H_{20}O_3Na_1$ $[M+Na]^+$ 331.1305, found 331.1313.

Ethyl 9b-methyl-10-oxo-4b,5,9b,10-tetrahydroindeno[2,1-*a*]indene-5-carboxylate (7z): R_f = 0.50

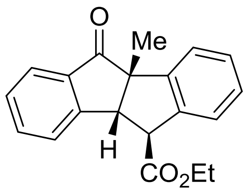

(pet.ether/EtOAc: 9/1). ^{1}H-NMR (500 MHz, $CDCl_3$): δ = 7.71 (d, *J* = 7.6 Hz, 1H), 7.64 (dt, *J* = 7.9, 1.2, Hz, 1H), 7.58 (dd, *J* = 7.7, 0.8 Hz, 1H), 7.56 (d, *J* = 7.4 Hz, 1H), 7.38 (tt, *J* = 7.3, 0.8 Hz, 1H), 7.33 (dd, *J* = 7.8, 0.6 Hz, 1H), 7.30 – 7.26 (m, 1H), 7.21 (dt, *J* = 7.5, 1.3 Hz, 1H), 4.27 (dq, *J* = 7.4, 1.6 Hz, 2H), 4.20 (dd, *J* = 8.0, 2.1 Hz, 2H), 1.71 (s, 3H), 1.34 (t, *J* = 7.2 Hz, 3H). ^{13}C-NMR (125 MHz, $CDCl_3$): δ = 206.6 (C_q), 173.1 (C_q), 155.1 (C_q), 144.6 (C_q), 138.9 (C_q), 135.4 (C_q), 128.9 (CH), 128.5 (CH), 128.5 (CH), 126.0 (CH), 125.7 (CH), 124.7 (CH), 124.6 (CH), 62.6 (C_q), 61.6 (CH_2), 56.1 (CH), 53.7 (CH), 22.9 (CH_3), 14.3 (CH_3). MS (ESI) *m/z* (relative intensity): 329.4 (100) $[M+Na]^+$. HR-MS (ESI) *m/z* calcd for $C_{20}H_{18}O_3Na_1$ $[M+Na]^+$ 329.1148, found 329.1154.

Additional indanones not included in the manuscript:

Ethyl 2-(3-oxo-2-phenyl-2,3-dihydro-1*H*-inden-1-yl)acetate (6ab): Representative procedure A was

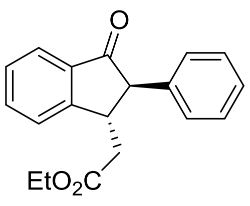

followed using ketone **4a** (98 mg, 0.50 mmol, 1.00 equiv) and ethyl acrylate **5b** (109 μL, 1.00 mmol, 2.00 equiv) for 8 h. Purification by column chromatography (pet.ether/EtOAc: 9/1) gave **6ab** (85 mg, 0.29 mmol, 58%) as a yellow oil (dr = 10:1), and dicyclized product **7ab** (12 mg, 0.04 mmol, 8%) as a yellow oil (single

diastereomer). **6ab**: R_f = 0.16 (pet.ether/EtOAc: 9/1). ^{1}H-NMR (400 MHz, $CDCl_3$): δ = 7.82 (d, *J* = 7.8 Hz, 1H), 7.67 (dt, *J* = 7.6, 1.1 Hz, 1H), 7.56 (dd, *J* = 7.6, 0.9 Hz, 1H), 7.47 (t, *J* = 7.4 Hz, 1H), 7.34 – 7.24 (m, 3H), 7.15 (dd, *J* = 8.5, 1.6 Hz, 2H), 4.08 (m, 2H), 3.90 (m, 1H), 3.66 (d, *J* = 4.8 Hz, 1H), 2.93 (dd, *J* = 15.5, 6.0 Hz, 1H), 2.77 (dd, *J* = 15.5, 7.8 Hz, 1H), 1.03 (t, *J* = 7.5 Hz, 3H). ^{13}C-NMR (101 MHz, $CDCl_3$): δ = 204.6 (C_q), 171.5 (C_q), 155.3 (C_q), 138.7 (C_q), 136.0 (C_q), 135.4 (CH), 128.9 (CH), 128.5 (CH), 128.4 (CH), 127.4 (CH), 125.3 (CH), 124.5 (CH), 60.9 (CH_2), 60.7 (CH), 44.6 (CH), 39.5 (CH_2), 14.1 (CH_3). MS (ESI) *m/z* (relative intensity): 317.2 (100) $[M+Na]^+$. HR-MS (ESI) *m/z* calcd for $C_{19}H_{18}O_3Na_1$ $[M+Na]^+$ 317.1148, found 317.1148.

Ethyl 10-oxo-4b,5,9b,10-tetrahydroindeno[2,1-*a*]indene-5-carboxylate (7ab): R_f = 0.21 (pet.ether/EtOAc: 9/1). ^{1}H-NMR (300 MHz, $CDCl_3$): δ = 7.73 (d, *J* = 7.7 Hz, 1H), 7.66 – 7.60 (m, 3H), 7.41 – 7.35 (m, 2H), 7.31 – 7.21 (m, 2H), 4.62 (dd, *J* = 6.9, 2.4 Hz, 1H), 4.41 (d, *J* = 6.7 Hz, 1H), 4.26 (q, *J* = 7.1 Hz, 2H), 4.23 (brs, 1H), 1.33 (t, *J* = 7.1 Hz, 3H). ^{13}C-NMR (75 MHz, $CDCl_3$): δ = 204.3 (C_q), 173.0 (C_q), 156.5 (C_q), 139.6 (C_q), 139.5 (C_q), 135.6 (C_q), 128.8 (CH), 128.5 (CH), 128.4 (CH), 126.2 (CH), 125.9 (CH), 125.4 (CH), 124.6 (CH), 61.7 (CH_2), 58.5 (CH), 56.7 (CH), 46.1 (CH), 14.4 (CH_3). MS (ESI) *m/z* (relative intensity): 315.1 (100) $[M+Na]^+$. HR-MS (ESI) *m/z* calcd for $C_{19}H_{16}O_3Na_1$ $[M+Na]^+$ 315.0997, found 315.0990.

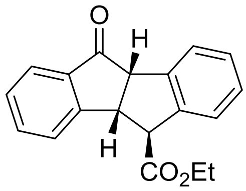

Butyl 2-(3-oxo-2-phenyl-2,3-dihydro-1*H*-inden-1-yl)acetate (6ac): Representative procedure A was followed using ketone **4a** (98 mg, 0.50 mmol, 1.00 equiv) and *n*-butyl acrylate **5c** (107 µL, 0.75 mmol, 1.50 equiv) for 8 h. Purification by column chromatography (pet.ether/EtOAc: 9/1) gave **6ac** (97 mg, 0.30 mmol, 60%) as a yellow oil (dr = 10:1). R_f = 0.23 (pet.ether/EtOAc: 9/1). ^{1}H-NMR (400 MHz, $CDCl_3$): δ = 7.76 (d, *J* = 7.8 Hz, 1H), 7.62 (dt, *J* = 7.5, 1.1 Hz, 1H), 7.51 (dd, *J* = 7.8, 0.9 Hz, 1H), 7.41 (t, *J* = 7.5 Hz, 1H), 7.28 – 7.18 (m, 3H), 7.09 (dd, *J* = 8.4, 1.6 Hz, 2H), 3.92 (m, 2H), 3.83 (m, 1H), 3.60 (d, *J* = 4.3 Hz, 1H), 2.87 (dd, *J* = 15.5, 6.0 Hz, 1H), 2.72 (dd, *J* = 15.5, 7.6 Hz, 1H), 1.43 – 1.36 (m, 2H), 1.24 – 1.14 (sextet, 2H), 0.81 (t, *J* = 7.3 Hz, 3H). ^{13}C-NMR (101 MHz, $CDCl_3$): δ = 204.6 (C_q), 171.6 (C_q), 155.3 (C_q), 138.6 (C_q), 136.0 (C_q), 135.4 (CH), 128.9 (CH), 128.5 (CH), 128.4 (CH), 127.3 (CH), 125.3 (CH), 124.5 (CH), 64.8 (CH_2), 60.7 (CH), 44.6 (CH), 39.4 (CH_2), 30.5 (CH_2), 19.4 (CH_2), 13.8 (CH_3). MS (ESI) *m/z* (relative intensity): 345.2 (100) $[M+Na]^+$. HR-MS (ESI) *m/z* calcd for $C_{21}H_{22}O_3Na_1$ $[M+Na]^+$ 345.1461, found 345.1471.

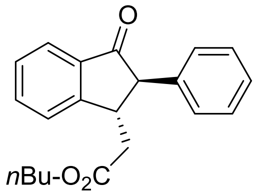

Ethyl 2-(6-methoxy-3-oxo-2-phenyl-2,3-dihydro-1*H*-inden-1-yl)acetate (6ad): Representative procedure A was followed using ketone **4b** (113 mg, 0.50 mmol, 1.00 equiv) and ethyl acrylate **5b** (109 µL, 1.00 mmol, 2.00 equiv) for 8 h. Purification by column chromatography (pet.ether/EtOAc: 9/1 to 4/1) gave **6ad** (97 mg, 0.30 mmol, 60%) as a yellow oil (dr = 15:1). R_f = 0.13 (pet.ether/EtOAc: 9/1). ^{1}H-NMR (500 MHz, $CDCl_3$): δ = 7.75 (d, *J* = 8.7 Hz, 1H), 7.23 (t, *J* = 7.5 Hz, 2H), 7.17 (tt, *J* = 7.4, 1.4 Hz, 1H), 7.09

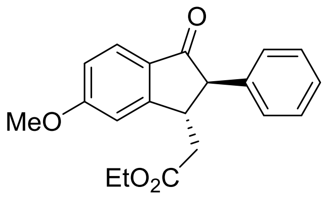

(dd, J = 8.7, 1.6 Hz, 2H), 6.92 – 6.89 (m, 2H), 4.08 (m, 2H), 3.83 (s, 3H), 3.75 (m, 1H), 3.56 (d, J = 4.2 Hz, 1H), 2.89 (dd, J = 15.6, 6.3 Hz, 1H), 2.74 (dd, J = 15.6, 7.7 Hz, 1H), 1.13 (t, J = 7.4 Hz, 3H). ^{13}C-NMR (125 MHz, $CDCl_3$): δ = 202.8 (C_q), 171.6 (C_q), 165.9 (C_q), 158.3 (C_q), 139.2 (C_q), 129.3 (C_q), 128.9 (CH), 128.3 (CH), 127.3 (CH), 126.3 (CH), 116.1 (CH), 108.9 (CH), 60.9 (CH_2), 60.8 (CH), 55.9 (s, OCH_3), 44.6 (CH), 39.8 (CH_2), 14.1 (CH_3). MS (ESI) *m/z* (relative intensity): 347.2 (95) $[M+Na]^+$. HR-MS (ESI) *m/z* calcd for $C_{20}H_{20}O_4Na_1$ $[M+Na]^+$ 347.1254, found 347.1255.

Isobutyl 2-(6-chloro-3-oxo-2-phenyl-2,3-dihydro-1*H*-inden-1-yl)acetate (6ae): Representative procedure A was followed using ketone **4c** (115 mg, 0.50 mmol, 1.00 equiv) and isobutyl acrylate **5d** (108 µL, 0.75 mmol, 1.50 equiv) for 14 h. Purification by column chromatography (pet.ether/EtOAc: 9/1) gave **6ae** (112 mg, 0.31 mmol, 63%) as a yellow oil (dr = 10:1). R_f = 0.35 (pet.ether/EtOAc: 9/1). ^{1}H-NMR (400 MHz, $CDCl_3$): δ = 7.68 (d, J = 8.2 Hz, 1H), 7.50 (bs, 1H), 7.37 (ddd, J = 8.2, 1.7, 0.7 Hz, 1H), 7.28 – 7.19 (m, 3H), 7.07 (dd, J = 8.4, 1.5, 2H), 3.80 (m, 1H), 3.73 (m, 2H), 3.62 (d, J = 4.5 Hz, 1H), 2.84 (dd, J = 15.7, 6.3 Hz, 1H), 2.75 (dd, J = 15.7, 7.1 Hz, 1H), 1.78 – 1.68 (septet, 1H), 0.79 (d, J = 2.5 Hz, 3H), 0.77 (d, J = 2.5 Hz, 3H). ^{13}C-NMR (101 MHz, $CDCl_3$): δ = 203.1 (C_q), 171.4 (C_q), 156.8 (C_q), 142.0 (C_q), 138.2 (C_q), 134.5 (C_q), 129.3 (CH), 129.1 (CH), 128.4 (CH), 127.6 (CH), 125.8 (CH), 125.6 (CH), 71.2 (CH_2), 60.6 (CH), 44.3 (CH), 38.9 (CH_2), 27.7 (CH), 19.2 (CH_3), 19.1 (CH_3). MS (ESI) *m/z* (relative intensity): 379.3 (100) $[M+Na]^+$. HR-MS (ESI) *m/z* calcd for $C_{21}H_{21}O_3Cl_1Na_1$ $[M+Na]^+$ 379.1071, found 379.1080.

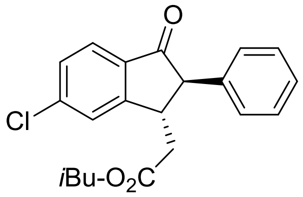

Cyclohexyl 2-(4-fluoro-3-oxo-2-phenyl-2,3-dihydro-1*H*-inden-1-yl)acetate (6af): Representative procedure A was followed using ketone **4h** (107 mg, 0.50 mmol, 1.00 equiv) and cyclohexyl acrylate **5e** (118 µL, 0.75 mmol, 1.50 equiv) for 14 h. Purification by column chromatography (pet.ether/EtOAc: 10/1) gave **6af** (100 mg, 0.28 mmol, 55%) as a yellow oil (dr = 15:1). R_f = 0.21 (pet.ether/EtOAc: 9/1). ^{1}H-NMR (400 MHz, $CDCl_3$): δ = 7.64 (dt, J = 7.9, 5.1 Hz, 1H), 7.35 – 7. 25 (m, 4H), 7.16 (dd, J =8.4, 1.5, 2H), 7.07 (t, J = 8.6 Hz, 1H), 4.68 (m, 1H), 3.87 (m, 1H), 3.71 (d, J = 4.5 Hz, 1H), 2.88 (dd, J = 15.5, 5.9 Hz, 1H), 2.79 (dd, J = 15.5, 7.1 Hz, 1H), 1.79 – 1.49 (m, 6H), 1.32 – 1.22 (m, 4H). ^{13}C-NMR (101 MHz, $CDCl_3$): δ = 200.8 (C_q), 170.7 (C_q), 159.1 (d, J_{CF} = 264.7 Hz, C_q), 157.4 (d, J_{CF} = 2.1 Hz, C_q), 138.2 (C_q), 137.2 (d, J_{CF} = 8.4 Hz, C_q), 129.0 (CH), 128.4 (CH), 127.5 (CH), 124.0 (d, J_{CF} = 12.9 Hz, CH), 121.0 (d, J_{CF} = 4.2 Hz, CH), 115.4 (d, J_{CF} = 19.4 Hz, CH), 73.6 (CH), 60.8 (CH), 44.5 (CH), 39.5 (CH_2), 31.6 (CH_2), 31.5 (CH_2), 25.3 (CH_2), 23.9 (CH_2), 23.8 (CH_2). ^{19}F-NMR (376 MHz, $CDCl_3$) δ = (-113.4 – -113.5) (m). MS (ESI) *m/z* (relative intensity): 389.3 (100) $[M+Na]^+$. HR-MS (ESI) *m/z* calcd for $C_{23}H_{23}O_3F_1Na_1$ $[M+Na]^+$ 389.1523, found 389.1519.

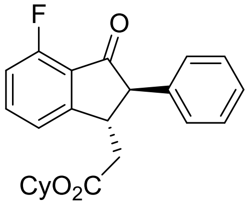

Methyl 2-(4-methyl-3-oxo-2-(4-(trifluoromethyl)phenyl)-2,3-dihydro-1*H*-inden-1-yl)acetate (6ag):

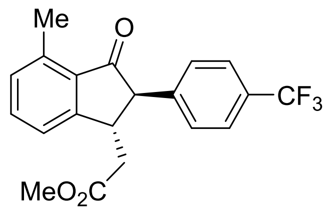

Representative procedure A was followed using ketone **4s** (139 mg, 0.50 mmol, 1.00 equiv) and methyl acrylate **5a** (90 μL, 1.00 mmol, 2.00 equiv) for 24 h. Purification by column chromatography using (pet.ether/EtOAc: 9/1) gave **6ag** (47 mg, 0.15 mmol, 20%) as a yellow oil (single diastereomer). R_f = 0.41 (pet.ether/EtOAc: 9/1). ^{1}H-NMR (500 MHz, $CDCl_3$): δ = 7.58 (d, J = 8.3 Hz, 2H), 7.54 (t, J = 7.6 Hz, 1H), 7.36 (d, J = 7.6 Hz, 1H), 7.28 (d, J = 8.3 Hz, 2H), 7.23 (dd, J = 7.5, 0.7 Hz, 1H), 3.86 – 3.72 (m, 1H), 3.70 (d, J = 4.5 Hz, 1H), 3.57 (s, 3H), 2.95 (dd, J = 15.7, 5.7 Hz, 1H), 2.71 (dd, J = 15.7, 8.2 Hz, 1H), 2.64 (s, 3H). ^{13}C-NMR (125 MHz, $CDCl_3$): δ = 204.4 (C_q), 171.9 (C_q), 155.7 (C_q), 143.0 (d, J_{CF} = 1.4 Hz, C_q), 139.9 (C_q), 134.9 (CH), 133.1 (C_q), 130.6 (CH), 129.7 (d, J_{CF} = 32.7 Hz, C_q), 128.8 (CH), 125.8 (q, J_{CF} = 3.7 Hz, CH), 124.3(q, J_{CF} = 271.3 Hz, C_q), 122.5 (CH), 60.7 (CH), 51.9 (OCH_3), 43.5 (CH), 39.5 (CH_2), 18.6 (CH_3). ^{19}F-NMR (470 MHz, $CDCl_3$) δ = -62.5 (s). MS (ESI) *m/z* (relative intensity): 363.4 (30) $[M+H]^+$, 385.4 (70) $[M+Na]^+$. HR-MS (ESI) *m/z* calcd for $C_{20}H_{17}O_3F_3Na_1$ $[M+Na]^+$ 385.1027, found 385.1020.

Synthesis of indole derivative methyl 2-(5,10-dihydroindeno[1,2-*b*]indol-10-yl)acetate, **8**.

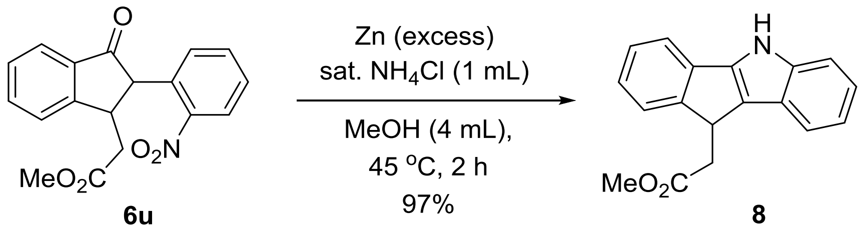

6u **8**

Monocyclized product **6u** (50 mg, 0.153 mmol) was dissolved in MeOH (4 mL). An excess of Zn (900 mg, 14 mmol) and 1 mL of saturated NH_4Cl aqueous solution were added, and the mixture heated at 45ºC for 2 h. The reaction mixture was quenched by adding saturated aq. $NaHCO_3$ and extracted with EtOAc (3 × 15mL). The organic layers were combined and dried over $MgSO_4$. The suspension was filtered and the filtrate was concentrated to ca. 1 mL. The crude was purified by columm cromatography on silica gel deactivated with Et_3N (eluent EtOAc: petroleum ether, 1:4). Compound **8** was obtained as a pale yellow oil (41 mg, 0.148 mmol, 97%). ^{1}H-NMR (500 MHz, $CDCl_3$): δ = 8.39 (s, 1H), 7.69 (dd, J = 7.8, 0.6 Hz, 1H), 7.50 (dd, J = 7.6, 0.9 Hz, 1H), 7.43 (tt, J = 7.5, 0.9 Hz, 1H), 7.41 (m, 1H), 7.33 (qt, J = 7.5, 0.5 Hz, 1H), 7.23 (dt, J = 7.5, 1.1 Hz, 1H), 7.19 (dt, J = 7.3, 1.4 Hz, 1H), 7.16 (dt, J = 7.3, 1.3 Hz, 1H), 4.41 (t, J = 7.6 Hz, 1H), 3.84 (s, 3H), 2.87 (dd, J = 16.0, 6.7 Hz, 1H), 2.75 (dd, J = 16.0, 8.3 Hz, 1H). ^{13}C-NMR (125 MHz, $CDCl_3$): δ = 173.7 (C_q), 151.2 (Cq), 142.6 (Cq), 140.8 (Cq), 134.4 (Cq), 127.4 (CH), 125.5 (CH), 124.8 (Cq), 124.7 (CH), 124.4 (Cq), 122.0 (CH), 120.5 (CH), 119.4 (CH), 117.7 (CH), 112.3 (CH), 52.1 (OCH_3), 39.2 (CH), 37.9 (CH_2). MS (ESI) *m/z* (relative intensity): 300.3 (50) $[M+Na]^+$. HR-MS (ESI) *m/z* calcd for $C_{18}H_{16}O_2N_1$ $[M+H]^+$ 278.1176, found 278.1178.

Synthesis of benzofuran derivative **10**.

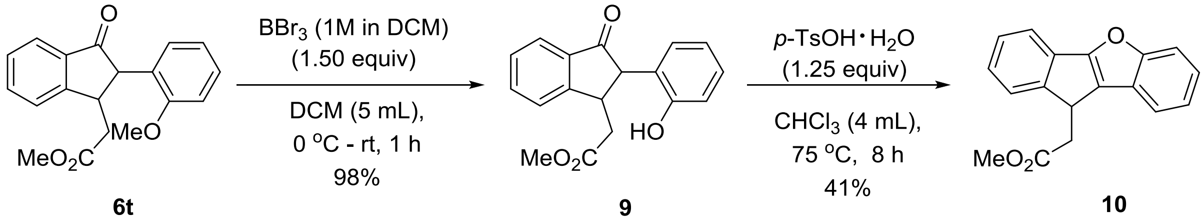

Preparation of the benzofuran (compound **10**) was carried out according to the following literature procedure:[20] Monocyclized product **6t** (150 mg, 0.484 mmol) was dissolved in CH_2Cl_2 (5 mL). The solution was cooled to 0 °C and a solution of BBr_3 in CH_2Cl_2 (1M, 0.7 mL, 0.7 mmol) was added dropwise and the mixture stirred for 1 h. The reaction was quenched with crushed ice and NH_4Cl (aq) and extracted with CH_2Cl_2 (3 × 15 mL). The organic layers were combined and dried over $MgSO_4$. The suspension was filtered and the filtrate was concentrated to afford the crude product. Further purification by column chromatography on silica gel (eluent EtOAc: petroleum ether, 1:4) afforded phenol **9** as an oily residue (140 mg, 0.473 mmol, 98%).

Methyl 2-(2-(2-hydroxyphenyl)-3-oxo-2,3-dihydro-1H-inden-1-yl)acetate (9): (dr = 10:1). ^{1}H-NMR (500 MHz, $CDCl_3$): δ = 7.80 (d, J = 7.6 Hz, 1H), 7.65 (dt, J = 7.5, 1.2 Hz, 1H), 7.51 (dd, J = 7.6, 0.8 Hz, 1H), 7.43 (tt, J = 7.5, 0.8 Hz, 1H), 7.10 (dt, J = 7.8, 1.7 Hz, 1H), 7.05 (dd, J = 7.6, 1.7 Hz, 1H), 6.86 (dt, J = 7.5, 1.2 Hz, 1H), 6.78 (dd, J = 8.0, 1.1 Hz, 1H), 6.59 (s, 1H), 4.03 (m, 1H), 3.82 (d, J = 4.9 Hz, 1H), 3.58 (s, 3H), 2.91 (dd, J = 15.6, 6.4 Hz, 1H), 2.80 (dd, J = 15.6, 7.3 Hz, 1H). ^{13}C-NMR (125 MHz, $CDCl_3$): δ = 206.3 (C_q), 172.6 (C_q), 155.3 (C_q), 154.4 (C_q), 135.8 (C_q), 135.5 (CH), 129.9 (CH), 128.9 (CH), 128.3 (CH), 125.1 (C_q), 125.0 (CH), 124.4 (CH), 120.9 (CH), 116.9 (CH), 56.9 (CH), 52.0 (OCH_3), 42.0 (CH), 38.8 (CH_2). MS (ESI) m/z (relative intensity): 318.9 (100) $[M+Na]^+$. HR-MS (ESI) m/z calcd for $C_{18}H_{16}O_4Na_1$ $[M+Na]^+$ 319.0941, found 319.0947.

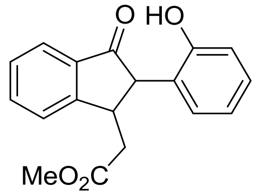

The phenol **9** (140 mg, 0.473 mmol) was dissolved in $CHCl_3$ (4 mL) and p-TsOH·H_2O (110 mg, 0.580 mmol) was added. The mixture was heated at 75 °C in a sealed vial for 8 h. After cooling the reaction mixture, it was concentrated to afford a crude reaction mixture that was purified by column chromatography on silica gel (eluent EtOAc: petroleum ether, 1:4), affording furan **10** as a colorless oil (63 mg, 0.227 mmol, 48%). The synthesis of furan **10** could alternatively be carried out in one pot without the isolation of intermediate phenol **9**, by adding p-TsOH·H_2O to the reaction mixture in CH_2Cl_2 and heating at 40 °C for 16 h. The crude product was then purified by column chromatography on silica gel to yield benzofuran **10** in 41% yield.

Methyl 2-(10*H*-indeno[1,2-*b*]benzofuran-10-yl)acetate (10): ^{1}H-NMR (500 MHz, $CDCl_3$): δ = 7.59 – 7.54 (m, 3H), 7.48 (qd, J = 7.3, 0.8 Hz, 1H), 7.36 (t, J = 7.5 Hz, 1H), 7.28 – 7.23 (m, 3H), 4.32 (dd, J = 9.4, 5.8 Hz, 1H), 3.81 (s, 3H), 2.97 (dd, J = 16.3, 5.9 Hz, 1H),

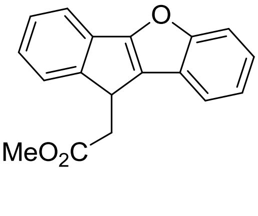

2.59 (dd, J = 16.3, 9.5 Hz, 1H). ^{13}C-NMR (125 MHz, $CDCl_3$): δ = 173.2 (C_q), 161.0 (Cq), 159.9 (Cq), 132.6 (Cq), 127.8 (CH), 126.4 (Cq), 126.3 (CH), 125.8 (Cq), 124.6 (CH), 123.7 (CH), 123.5 (CH), 120.0 (CH), 118.0 (CH), 112.5 (CH), 52.1 (OCH_3), 38.2 (CH), 36.9 (CH_2). MS (ESI) *m/z* (relative intensity): 300.9 (100) $[M+Na]^+$. HR-MS (ESI) *m/z* calcd for $C_{18}H_{14}O_3Na_1$ $[M+Na]^+$ 301.0835, found 301.0827.

Representative procedure B for the synthesis of dicyclized compounds 12a –12l.

To an oven dried 5 mL microwave vial containing a stir bar was added ketone **11a** (136 mg, 0.50 mmol, 1.00 equiv), Ru[(p-cymene)Cl_2]$_2$ (15.2 mg, 5.0 mol%) and $Cu(OAc)_2$•H_2O (150 mg, 0.75 mmol, 1.50 equiv). The vial was sealed with a rubber septum and purged with N_2. A freshly prepared stock solution of $AgSbF_6$ (17.5 mg, 10 mol%) in dry 1,2-dichloroethane (0.50 mL) was added to this vial and the mixture was stirred for 10 min at room temperature. Then methyl acrylate **5a** (90 μL, 1.00 mmol, 2.00 equiv) was added via syringe and the rubber septum was replaced with aluminium crimp cap [the reaction was open to air during the process of swapping seals]. The vial was tightly sealed and the mixture was heated with stirring in a preheated oil bath at 100 °C for 16 h. After the allotted time the mixture was cooled to room temperature, diluted with EtOAc (5 mL) and filtered through a celite pad. The pad was further washed with EtOAc (25 mL) and the combined organic solvent evaporated *in vacuo* to afford a crude mixture. This was purified by column chromatography (silica, pet.ether/EtOAc: 3/1) to yield **12a** (158 mg, 0.45 mmol, 89%, single diastereoisomer) as a crystalline white solid.

Methyl 10-oxo-9b-phenyl-4b,5,9b,10-tetrahydroindeno[2,1-*a*]indene-5-carboxylate (12a): M. p. = 140 - 142 °C. R_f = 0.33 (pet.ether/EtOAc: 3/1). ^{1}H-NMR (400 MHz, $CDCl_3$): δ = 7.75 (d, J = 7.7 Hz, 1H), 7.61 (dt, J = 7.7, 1.2 Hz, 1H), 7.55 (dd, J = 7.7, 0.9 Hz, 1H), 7.54 – 7.50 (m, 1H), 7.38 (td, J = 7.7, 0.9 Hz, 1H), 7.35 (d, J = 7.5 Hz, 1H), 7.27 – 7.25 (m, 2H), 7.22 – 7. 15 (m, 3H), 7.03 (dd, J = 8.4, 1.6 Hz, 2H), 4.47 (d, J = 2.2 Hz, 1H), 4.18 (d, J = 2.2 Hz, 1H), 3.65 (s, 3H). ^{13}C-NMR (101 MHz, $CDCl_3$): δ = 205.3 (C_q), 173.0 (C_q), 155.5 (C_q), 143.2 (C_q), 142.1 (C_q), 139.9 (C_q), 136.0 (C_q), 135.9 (CH), 129.0 (CH), 128.9 (CH), 128.7 (CH), 128.5 (CH), 127.7 (CH), 127.1 (CH), 126.8 (CH), 126.1 (CH), 126.0 (CH), 124.8 (CH), 71.3 (C_q), 57.3 (CH), 56.6 (CH), 52.6 (OCH_3). MS (ESI) *m/z* (relative intensity): 377.2 (100) $[M+Na]^+$. HR-MS (ESI) *m/z* calcd for $C_{24}H_{18}O_3Na_1$ $[M+Na]^+$ 377.1148, found 377.1161.

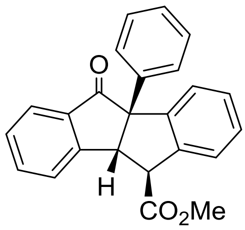

Ethyl 10-oxo-9b-phenyl-4b,5,9b,10-tetrahydroindeno[2,1-*a*]indene-5-carboxylate (12b): Representative procedure B was followed using ketone **11a** (136 mg, 0.50 mmol, 1.00 equiv) and ethyl acrylate **5b** (109 μL, 1.00 mmol, 2.00 equiv) for 16 h. Purification by column chromatography (pet.ether/EtOAc: 3/1) gave **12b** (141 mg, 0.38 mmol, 77%) as a yellow oil. R_f = 0.34 (pet.ether/EtOAc: 3/1). ^{1}H-NMR (400 MHz, $CDCl_3$): δ = 7.75 (d, J = 7.8 Hz, 1H), 7.61 (dt, J = 7.7, 1.2 Hz, 1H), 7.55 (dd, J = 7.7, 0.9 Hz, 1H), 7.54 – 7.51 (m, 1H), 7.37 (td, J = 7.5, 1.0, 1H), 7.35 (d, J = 7.7 Hz, 1H), 7.27 – 7.25 (m, 2H), 7.22 – 7. 15 (m,

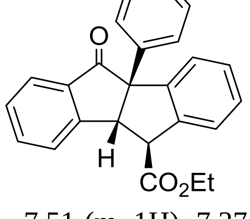

3H), 7.04 (dd, J = 8.4, 1.6 Hz, 2H), 4.47 (d, J = 2.2 Hz, 1H), 4.15 (d, J = 2.2 Hz, 1H), 4.08 (m, 2H), 1.10 (t, J = 7.2 Hz, 3H). ^{13}C-NMR (101 MHz, $CDCl_3$): δ = 205.4 (C_q), 172.5 (C_q), 155.5 (C_q), 143.3 (C_q), 142.0 (C_q), 140.1 (C_q), 136.0 (C_q), 135.9 (CH), 129.0 (CH), 128.9 (CH), 128.7 (CH), 128.5 (CH), 127.7 (CH), 127.1 (CH), 126.8 (CH), 126.1 (CH), 126.0 (CH), 124.8 (CH), 71.4 (C_q), 61.5 (CH_2), 57.4 (CH), 56.8 (CH), 14.1 (CH_3). MS (ESI) *m/z* (relative intensity): 391.2 (100) $[M+Na]^+$. HR-MS (ESI) *m/z* calcd for $C_{25}H_{21}O_3$ $[M+H]^+$ 369.1485, found 369.1489.

Isobutyl 10-oxo-9b-phenyl-4b,5,9b,10-tetrahydroindeno[2,1-*a*]indene-5-carboxylate (12c):

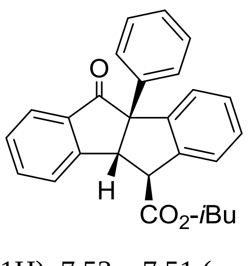

Representative procedure B was followed using ketone **11a** (136 mg, 0.50 mmol, 1.00 equiv) and isobutyl acrylate **5d** (144 µL, 1.00 mmol, 2.00 equiv) for 24 h. Purification by column chromatography (pet.ether/EtOAc: 15/1) gave **12c** (139 mg, 0.35 mmol, 70%) as a yellow oil. R_f = 0.54 (pet.ether/EtOAc: 9/1). ^{1}H-NMR (500 MHz, $CDCl_3$): δ = 7.74 (d, J = 7.8 Hz, 1H), 7.62 (dt, J = 7.7, 1.2 Hz, 1H), 7.55 (dd, J = 7.6, 0.9 Hz, 1H), 7.53 – 7.51 (m, 1H), 7.37 (td, J = 7.4, 1.0 Hz, 1H), 7.35 (d, J = 7.7 Hz, 1H), 7.27 - 7.25 (m, 2H), 7.21 – 7. 16 (m, 3H), 7.04 (dd, J = 8.4, 1.6 Hz, 2H), 4.48 (d, J = 2.2 Hz, 1H), 4.18 (d, J = 2.2 Hz, 1H), 3.83 (d, J = 6.5 Hz, 2H), 1.82 – 1.74 (m, 1H), 0.77 (t, J = 6.8 Hz, 6H). ^{13}C-NMR (125 MHz, $CDCl_3$): δ = 205.3 (C_q), 172.5 (C_q), 155.6 (C_q), 143.2 (C_q), 142.0 (C_q), 140.1 (C_q), 135.6 (C_q), 129.0 (CH), 128.9 (CH), 128.7 (CH), 128.5 (CH), 127.7 (CH), 127.1 (CH), 126.8 (CH), 126.1 (CH), 126.0 (CH), 124.8 (CH), 71.6 (C_q), 71.3 (CH_2), 57.4 (CH), 56.9 (CH), 27.8 (CH), 19.1 (CH_3), 19.0 (CH_3). MS (ESI) *m/z* (relative intensity): 419.2 (100) $[M+Na]^+$. HR-MS (ESI) *m/z* calcd for $C_{27}H_{24}O_3Na_1$ $[M+Na]^+$ 419.1618, found 419.1616.

Cyclohexyl 10-oxo-9b-phenyl-4b,5,9b,10-tetrahydroindeno[2,1-*a*]indene-5-carboxylate (12d):

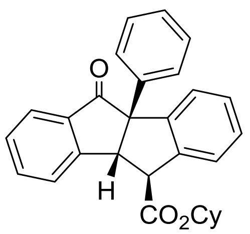

Representative procedure B was followed using ketone **11a** (136 mg, 0.50 mmol, 1.00 equiv) and cyclohexyl acrylate **5e** (158 µL, 1.00 mmol, 2.00 equiv) for 24 h. Purification by column chromatography (pet.ether/EtOAc: 15/1) gave **12d** (158 mg, 0.37 mmol, 75%) as a dense yellow oil. R_f = 0.5 (pet.ether/EtOAc: 9/1). ^{1}H-NMR (400 MHz, $CDCl_3$): δ = 7.73 (d, J = 7.7 Hz, 1H), 7.60 (dt, J = 7.8, 1.2 Hz, 1H), 7.55 (dd, J = 7.7, 1.0 Hz, 1H), 7.52 – 7.50 (m, 1H), 7.37 – 7.34 (m, 2H), 7.27 - 7.25 (m, 2H), 7.21 – 7. 16 (m, 3H), 7.03 (dd, J = 8.2, 1.5 Hz, 2H), 4.73 – 4.69 (m, 1H), 4.48 (d, J = 2.2 Hz, 1H), 4.14 (d, J = 2.2 Hz, 1H), 1.71 – 1.64 (m, 2H), 1.57 – 1.54 (m, 2H), 1.44 – 1.39 (m, 1H), 1.28 – 1.18 (m, 5H). ^{13}C-NMR (101 MHz, $CDCl_3$): δ = 205.4 (C_q), 171.8 (C_q), 155.6 (C_q), 143.2 (C_q), 141.9 (C_q), 140.3 (C_q), 135.9 (C_q), 135.8 (CH), 128.9 (CH), 128.8 (CH), 128.7 (CH), 128.5 (CH), 127.7 (CH), 127.0 (CH), 126.7 (CH), 126.1 (CH), 126.0 (CH), 124.7 (CH), 73.8 (CH), 71.6 (C_q), 57.4 (CH), 57.0 (CH), 31.4 (CH_2), 31.3 (CH_2), 25.3 (CH_2), 23.6 (CH_2), 23.5 (CH_2). MS (ESI) *m/z* (relative intensity): 445.3 (100) $[M+Na]^+$. HR-MS (ESI) *m/z* calcd for $C_{29}H_{26}O_3Na_1$ $[M+Na]^+$ 445.1774, found 445.1786.

4b-Phenyl-10-(phenylsulfonyl)-9b,10-dihydroindeno[2,1-*a*]inden-5(4b*H*)-one (12e): Representative

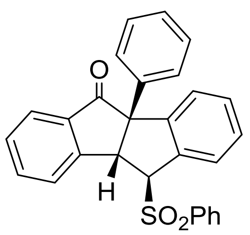

procedure B was followed using ketone **11a** (136 mg, 0.50 mmol, 1.00 equiv) and

phenyl vinyl sulfone **5f** (126 mg, 0.75 mmol, 1.50 equiv) for 16 h. Purification by column chromatography (pet.ether/EtOAc: 6/1) gave **12e** (203 mg, 0.47 mmol, 93%) as a white crystalline solid. M. p. = 180 – 182 ^oC. R_f = 0.3 (pet.ether/EtOAc: 9/1). ^{1}H-NMR (300 MHz, $CDCl_3$): δ = 7.77 (d, J = 7.5 Hz, 1H), 7.73 (d, J = 7.3 Hz, 1H), 7.66 – 7.63 (m, 2H), 7.56 – 7.41 (m, 7H), 7.21 (t, J = 7.4 Hz, 2H), 7.12 (d, J = 7.4 Hz, 1H), 7.04 (t, J = 7.7 Hz, 2H), 6.56 (d, J = 7.4 Hz, 2H), 4.84 (d, J = 2.2 Hz, 1H), 4.49 (d, J = 2.2 Hz, 1H). ^{13}C-NMR (75 MHz, $CDCl_3$): δ = 204.0 (C_q), 154.0 (C_q), 142.6 (C_q), 142.2 (C_q), 136.4 (CH), 135.9 (C_q), 135.3 (C_q), 134.6 (C_q), 134.0 (CH), 130.2 (CH), 129.7 (CH), 129.4 (CH), 129.2 (CH), 128.9 (CH), 128.6 (CH), 127.4 (CH), 127.1 (CH), 126.8 (CH), 126.6 (CH), 126.3 (CH), 125.0 (CH), 77.4 (C_q), 69.8 (CH), 56.3 (CH). MS (ESI) *m/z* (relative intensity): 459.2 (100) $[M+Na]^+$. HR-MS (ESI) *m/z* calcd for $C_{28}H_{20}O_3Na_1S_1$ $[M+Na]^+$ 459.1025, found 459.1025.

2,2,2-Trifluoroethyl10-oxo-9b-phenyl-4b,5,9b,10-tetrahydro indeno[2,1-*a*]indene-5-carboxylate (12f):

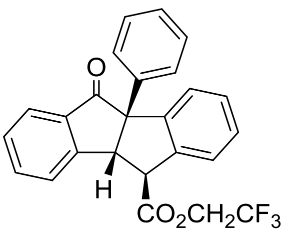

Representative procedure B was followed using ketone **11a** (136 mg, 0.50 mmol, 1.00 equiv) and 2,2,2-trifluoroethyl acrylate **5g** (127 µl, 1.00 mmol, 2.00 equiv) for 16 h. Purification by column chromatography (pet.ether/EtOAc: 9/1) gave **12f** (171 mg, 0.41 mmol, 81%) as a colorless oil. R_f = 0.3 (pet.ether/EtOAc: 9/1). ^{1}H-NMR (500 MHz, $CDCl_3$): δ = 7.73 (d, J = 7.7 Hz, 1H), 7.61 (dt, J = 7.7, 1.3 Hz, 1H), 7.51 (dd, J = 6.8, 0.9 Hz, 1H), 7.50 – 7.49 (m, 1H), 7.36 (t, J = 7.5 Hz, 1H), 7.33 – 7.31 (m, 1H), 7.28 – 7.25 (m, 2H), 7.18 – 7.14 (m, 3H), 6.98 (dd, J = 8.4, 1.6 Hz, 2H), 4.44 – 4.39 (m, 2H), 4.42 (qd, J = 8.4, 1.3 Hz, 1H), 4.25 (d, J = 2.3 Hz, 1H). ^{13}C-NMR (125 MHz, $CDCl_3$): δ = 204.9 (C_q), 171.0 (C_q), 154.9 (C_q), 143.0 (C_q), 142.2 (C_q), 138.8 (C_q), 136.1 (CH), 136.0 (CH), 129.4 (CH), 129.4 (CH), 128.9 (CH), 128.6 (CH), 127.6 (CH), 127.3 (CH), 127.0 (CH), 126.1 (CH), 126.0 (CH), 125.0 (CH), 122.9 (q, J_{CF} = 278.0 Hz, C_q), 121.8 (CH), 71.2 (CH), 61.0 (q, J_{CF} = 111.0 Hz, CH_2), 57.3 (CH), 56.1 (CH). 19F-NMR (470 MHz, $CDCl_3$): δ = -73.7 (t, J_{FH} = 8.3 Hz, CH_2CF_3). MS (ESI) *m/z* (relative intensity): 445.4 (90) $[M+Na]^+$, 377.4 (100) $[M^+ +Na-CF_3]$. HR-MS (ESI) *m/z* calcd for $C_{25}H_{21}O_3N_1F_3$ $[M^+ +NH_4]$ 440.1468, found 440.1456.

Methyl 7-methoxy-9b-(4-methoxyphenyl)-10-oxo-4b,5,9b,10-tetrahydroindeno[2,1-*a*]indene-5-carboxylate (12g):

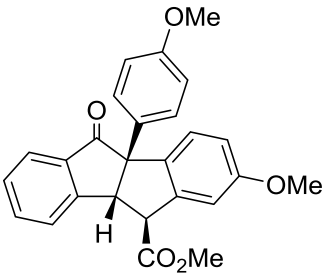

Representative procedure B was followed using ketone **11b** (166 mg, 0.50 mmol, 1.00 equiv) and methyl acrylate **5a** (90 µL, 1.00 mmol, 2.00 equiv) for 16 h. Purification by column chromatography using (pet.ether/EtOAc: 9/1) gave **12g** (176 mg, 0.43 mmol, 85%) as a dark brown oil. R_f = 0.2 (pet.ether/EtOAc: 3/1). ^{1}H-NMR (400 MHz, $CDCl_3$): δ = 7.80 (d, J = 7.8 Hz, 1H), 7.63 (dt, J = 7.7, 1.1 Hz, 1H), 7.60 (dd, J = 7.7, 0.9 Hz, 1H), 7.46 (d, J = 7.2 Hz, 1H), 7.42 (d, J = 7.5, 1H), 7.02 (d, J = 8.8 Hz, 2H), 6.91 – 6.89 (m, 2H), 6.81 (d, J = 8.8 Hz, 2H), 4.47 (d, J = 2.2 Hz, 1H), 4.19 (d, J = 2.2 Hz, 1H), 3.77 (s, 3H), 3.76 (s, 3H), 3.73 (s, 3H). ^{13}C-NMR (101 MHz, $CDCl_3$): δ = 205.8 (C_q), 172.9 (C_q), 160.7 (C_q), 158.5 (C_q), 155.3 (C_q), 141.2 (C_q), 141.1 (C_q), 135.4 (C_q), 134.5 (C_q), 134.4 (CH), 128.8 (CH), 128.6 (CH), 127.4 (CH), 126.0 (CH), 124.8 (CH), 115.5 (CH), 113.9 (CH), 110.6 (CH), 69.9 (C_q), 57.8 (CH), 56.6 (CH), 55.5 (OCH_3), 55.3 (OCH_3), 52.7 (OCH_3).

MS (ESI) *m/z* (relative intensity): 437.2 (100) [M+Na]$^+$. HR-MS (ESI) *m/z* calcd for $C_{26}H_{22}O_5Na_1$ [M+Na]$^+$ 437.1359, found 437.1357.

***n*-Butyl 7-methoxy-9b-(4-methoxyphenyl)-10-oxo-4b,5,9b,10-tetrahydroindeno[2,1-*a*]indene-5-carboxylate (12h):**

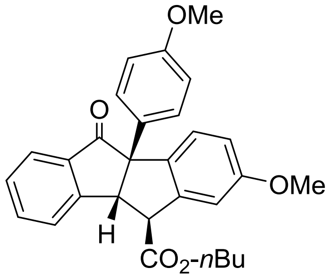

Representative procedure B was followed using ketone **11b** (166 mg, 0.50 mmol, 1.00 equiv) and butyl acrylate **5c** (145 μL, 1.00 mmol, 2.00 equiv) for 16 h. Purification by column chromatography using (pet.ether/EtOAc: 9/1) gave **12h** (173 mg, 0.38 mmol, 76%) as a dark yellow oil. R_f = 0.4 (pet.ether/EtOAc: 3/1). ^{1}H-NMR (400 MHz, $CDCl_3$): δ = 7.80 (d, *J* = 7.7 Hz, 1H), 7.67 (dt, *J* = 7.1, 1.0 Hz, 1H), 7.61 (dd, *J* = 7.7, 0.8 Hz, 1H), 7.47 (d, *J* = 8.4 Hz, 1H), 7.42 (tt, *J* = 7.3, 0.9, 1H), 7.03 (d, *J* = 8.9 Hz, 2H), 6.93 – 6.89 (m, 2H), 6.81 (d, *J* = 8.9 Hz, 2H), 4.50 (d, *J* = 2.3 Hz, 1H), 4.18 (d, *J* = 2.3 Hz, 1H), 4.12 (dt, *J* = 6.7, 2.3 Hz, 2H), 3.77 (s, 3H), 3.75 (s, 3H), 1.57 – 1.50 (m, 2H), 1.32 – 1.22 (m, 2H), 0.88 (t, *J* = 7.9 Hz, 3H). ^{13}C-NMR (101 MHz, $CDCl_3$): δ = 205.8 (C_q), 172.4 (C_q), 160.6 (C_q), 158.4 (C_q), 155.3 (C_q), 141.3 (C_q), 135.8 (C_q), 135.7 (CH), 135.4 (C_q), 134.3 (C_q), 128.7 (CH), 128.6 (CH), 127.3 (CH), 125.9 (CH), 124.7 (CH), 115.7 (CH), 113.8 (CH), 110.4 (CH), 69.9 (C_q), 65.4 (CH_2), 57.8 (CH), 56.8 (CH), 55.4 (OCH_3), 55.2 (OCH_3), 30.6 (CH_2), 19.1 (CH_2), 13.7 (CH_3). MS (ESI) *m/z* (relative intensity): 479.2 (100) [M+Na]$^+$. HR-MS (ESI) *m/z* calcd for $C_{29}H_{28}O_5Na_1$ [M+Na]$^+$ 479.1829, found 479.1841.

Methyl 3-methyl-10-oxo-9b-phenyl-4b,5,9b,10-tetrahydroindeno[2,1-*a*]indene-5-carboxylate (12i):

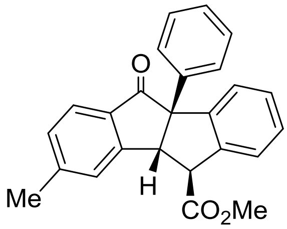

Representative procedure B was followed using ketone **11c** (143 mg, 0.50 mmol, 1.00 equiv) and methyl acrylate **5a** (90 μL, 1.00 mmol, 2.00 equiv) for 16 h. Purification by column chromatography (pet.ether/EtOAc: 10/1) gave **12i** (169 mg, 0.46 mmol, 92%) as a dense yellow oil. R_f = 0.3 (pet.ether/EtOAc: 9/1). ^{1}H-NMR (400 MHz, $CDCl_3$): δ = 7.64 (d, *J* = 7.9 Hz, 1H), 7.53 – 7.51 (m, 1H), 7.36 (bs, 1H), 7.35 – 7.32 (m, 1H), 7.27 – 7.25 (m, 2H), 7.21 – 7.15 (m, 4H), 7.04 (dd, *J* = 8.3, 1.5 Hz, 2H), 4.41 (d, *J* = 2.2 Hz, 1H), 4.18 (d, *J* = 2.2 Hz, 1H), 3.65 (s, 3H), 2.40 (s, 3H). ^{13}C-NMR (101 MHz, $CDCl_3$): δ = 204.8 (C_q), 173.0 (C_q), 156.0 (C_q), 147.4 (C_q), 143.4 (C_q), 142.2 (C_q), 139.9 (C_q), 133.6 (C_q), 130.0 (CH), 128.9 (CH), 128.8 (CH), 128.5 (CH), 127.7 (CH), 127.0 (CH), 126.8 (CH), 126.3 (CH), 126.0 (CH), 124.6 (CH), 71.5 (C_q), 57.2 (CH), 56.5 (CH), 52.6 (OCH_3), 22.3 (CH_3). MS (ESI) *m/z* (relative intensity): 391.5 (100) [M+Na]$^+$. HR-MS (ESI) *m/z* calcd for $C_{25}H_{20}O_3Na_1$ [M+Na]$^+$ 391.1305, found 391.1316.

8-Methyl-4b-phenyl-10-(phenylsulfonyl)-9b,10-dihydroindeno[2,1-*a*]inden-5(4b*H*)-one (12j):

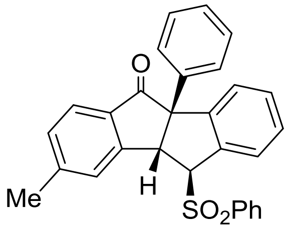

Representative procedure B was followed using ketone **11c** (143 mg, 0.50 mmol, 1.00 equiv) and phenyl vinyl sulfone **5f** (126 mg, 0.75 mmol, 1.50 equiv) for 16 h. Purification by column chromatography (pet.ether/EtOAc: 8/1) gave **12j** (210 mg, 0.47 mmol, 93%) as a light yellow solid. M. p. = 198- 200°C. R_f = 0.2

(pet.ether/EtOAc: 9/1). ^{1}H-NMR (400 MHz, $CDCl_3$): δ = 7.66 (d, J = 7.5 Hz, 2H), 7.56 – 7.54 (m, 1H), 7.52 – 7.47 (m, 3H), 7.44 – 7.40 (m, 3H), 7.28 (td, J = 7.9, 0.7 Hz, 1H), 7.22 – 7.18 (m, 2H), 7.11 (tt, J = 7.4, 1.3, 1H), 7.03 (tt, J = 7.7, 1.5 Hz, 2H), 6.55 (dd, J = 7.0, 1.4 2H), 4.84 (d, J = 2.3 Hz, 1H), 4.43 (d, J = 2.3 Hz, 1H), 2.5 (s, 3H). ^{13}C-NMR (101 MHz, $CDCl_3$): δ = 203.6 (C_q), 154.7 (C_q), 148.2 (C_q), 142.9 (C_q), 142.5 (C_q), 136.0 (C_q), 134.7 (C_q), 134.2 (CH), 133.1 (C_q), 130.6 (CH), 130.2 (CH), 129.9 (CH), 129.2 (CH), 129.0 (CH), 128.7 (CH), 127.5 (CH), 127.2 (CH), 126.8 (CH), 126.7 (CH), 125.0 (CH), 77.5 (C_q), 70.1 (CH), 56.3 (CH), 22.3 (CH_3). MS (ESI) *m/z* (relative intensity): 451.3 (100) $[M+H]^+$. HR-MS (ESI) *m/z* calcd for $C_{29}H_{26}O_3N_1S_1$ $[M+NH_4]^+$ 468.1628, found 468.1622.

2-Fluoro-4b-(4-fluorophenyl)-10-(phenylsulfonyl)-9b,10-dihydroindeno[2,1-*a*]inden-5(4b*H*)-one (12k):

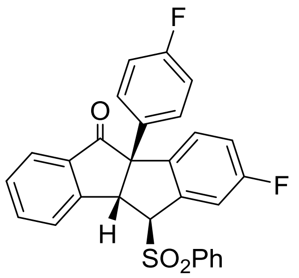

Representative procedure B was followed using ketone **11d** (124 mg, 0.40 mmol, 1.00 equiv), phenyl vinyl sulfone **5f** (101 mg, 0.60 mmol, 1.50 equiv), Ru[(*p*-cymene)Cl_2]$_2$ (12.5 mg, 5.0 mol%) and $Cu(OAc)_2$,•H_2O (120 mg, 0.60 mmol, 1.50 equiv). The vial was sealed with a rubber septum and purged with N_2. A freshly prepared stock solution of $AgSbF_6$ (14.0 mg, 10 mol%) in 1,2-dichloroethane (0.50 mL) was added and mixture was heated with stirring at 100 ^{o}C for 16 h. Purification by column chromatography (pet.ether/EtOAc: 9/1 to 3/1) gave **12k** (158 mg, 0.47 mmol, 83%) as a dense colorless oil. R_f = 0.2 (pet.ether/EtOAc: 9/1). ^{1}H-NMR (400 MHz, $CDCl_3$): δ = 7.78 (d, J = 7.7 Hz, 1H), 7.73 (dt, J = 7.6, 1.2 Hz, 1H), 7.60 – 7.53 (m, 4H), 7.50 (d, J = 8.3 Hz, 1H), 7.49 (dd, J = 8.7, 1.5 Hz, 1H), 7.34 – 7.30 (m, 2H), 7.29 (bd, J = 2.4 Hz, 1H), 7.14 (ddt, J = 8.7, 2.6, 0.6 Hz, 1H), 6.76 (tt, J = 8.7, 2.2 Hz, 2H), 6.57 (tdd, J = 7.0, 5.1, 2.2 Hz, 2H), 4.78 (d, J = 2.7 Hz, 1H), 4.45 (d, J = 2.7 Hz, 1H). ^{13}C-NMR (101 MHz, $CDCl_3$): δ = 203.7 (C_q), 163.8 (d, J_{CF} = 196.6 Hz, C_q), 161.3 (d, J_{CF} = 193.8 Hz, C_q), 153.5 (C_q), 139.5 (C_q), 138.5 (d, J_{CF} = 2.9 Hz, C_q), 137.9 (d, J_{CF} = 3.0 Hz, C_q), 136.8 (Cq), 136.5 (d, J_{CF} = 8.9 Hz, C_q), 135.9 (CH), 134.9 (CH), 134.5 (CH), 133.7 (CH), 129.7 (CH), 129.5 (CH), 129.4 (CH), 129.2 (CH), 128.4 (d, J_{CF} = 8.9 Hz, CH), 127.9 (CH), 127.8 (CH), 125.6 (d, J_{CF} = 109 Hz, CH), 118.1 (d, J_{CF} = 22.8 Hz, CH), 115.5 (d, J_{CF} = 21.2 Hz, CH), 114.1 (d, J_{CF} = 22.8 Hz, CH), 76.7 (d, J_{CF} = 2.4 Hz, CH), 68.4 (C_q), 56.7 (CH). ^{19}F-NMR (376 MHz, $CDCl_3$), δ = (-111.36 – -111.42) (m), (-115.12 – -115.19) (m). MS (ESI) *m/z* (relative intensity): 495.5 (100) $[M+Na]^+$. HR-MS (ESI) *m/z* calcd for $C_{28}H_{18}O_3F_2Na_1S_1$ $[M+Na]^+$ 495.0837, found 495.0830.

Additional example not included in Scheme 3

Isobutyl 3-methyl-10-oxo-9b-phenyl-4b,5,9b,10-tetrahydroindeno[2,1-*a*]indene-5-carboxylate (12l):

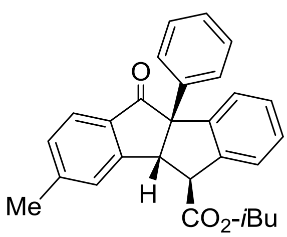

Representative procedure B was followed using ketone **11c** (143 mg, 0.50 mmol, 1.00 equiv) and methyl acrylate **5d** (108 μL, 0.75 mmol, 1.50 equiv) for 16 h. Purification by column chromatography (pet.ether/EtOAc: 12/1) gave **12l** (185 mg, 0.45 mmol, 90%) as a dense yellow oil. R_f = 0.5 (pet.ether/EtOAc: 9/1). ^{1}H-

NMR (400 MHz, $CDCl_3$): δ = 7.55 (d, J = 7.9 Hz, 1H), 7.45 – 7.43 (m, 1H), 7.28 (bs, 1H), 7.28 – 7.26 (m, 1H), 7.18 – 7.16 (m, 2H), 7.10 – 7.05 (m, 4H), 6.94 (dd, J = 8.3, 1.5 Hz, 2H), 4.35 (d, J = 2.4 Hz, 1H), 4.10 (d, J = 2.4 Hz, 1H), 3.74 (d, J = 6.9 Hz, 2H), 2.31 (s, 3H), 1.73 – 1.66 (m, 1H), 0.69 (t, J = 6.6 Hz, 6H). ^{13}C-NMR (101 MHz, $CDCl_3$): δ = 204.9 (C_q), 172.6 (C_q), 156.1 (C_q), 147.3 (C_q), 143.4 (C_q), 142.1 (C_q), 140.1 (C_q), 133.6 (C_q), 130.0 (CH), 128.9 (CH), 128.8 (CH), 128.5 (CH), 127.7 (CH), 127.0 (CH), 126.8 (CH), 126.3 (CH), 126.1 (CH), 124.6 (CH), 71.6 (CH_2), 71.5 (C_q), 57.3 (CH), 56.8 (CH), 27.8 (CH), 22.3 (CH_3), 19.1 (CH_3), 19.0 (CH_3). MS (ESI) *m/z* (relative intensity): 433.6 (100) $[M+Na]^+$. HR-MS (ESI) *m/z* calcd for $C_{28}H_{26}O_3Na_1$ $[M+Na]^+$ 433.1774, found 433.1787.

Representative procedure C for the synthesis of lactones 13a-m.

To an oven dried 5 mL microwave vial containing a stir bar was added ketone **4a** (98 mg, 0.50 mmol, 1.00 equiv), Ru[(p-cymene)Cl_2]$_2$ (15.2 mg, 5.0 mol%) and $Cu(OAc)_2$•H_2O (150 mg, 0.75 mmol, 1.50 equiv). The vial was sealed with a rubber septum and purged with N_2. A freshly prepared stock solution of $AgSbF_6$ (17.5 mg, 10 mol%) in dry 1,2-dichloroethane (0.50 mL) was added to this vial and the mixture was stirred for 10 min at room temperature. Then methyl acrylate **5a** (90 μL, 1.00 mmol, 2.00 equiv) was added via syringe and the rubber septum replaced with an aluminium crimp cap [the reaction was open to air during the process of swapping seals]. The vial was tightly sealed and the mixture heated with stirring in a preheated oil bath at 100 °C for 8 h. Then mixture was allowed to cool to room temperature, HBF_4 (48% soln in H_2O, 1.00 equiv) and $Cu(OAc)_2$•H_2O (100 mg, 0.50 mmol, 1.00 equiv) were added. The vial was again sealed and the mixture was further heated with stirring at 100 °C for 16 h. After allotted time the mixture was cooled to room temperature, diluted with EtOAc (5 mL) and filtered through celite pad. The pad was further washed with EtOAc (25 mL) and the combined organic solvent was evaporated *in vacuo* to afford a crude mixture. This was purified using column chromatography (silica, pet.ether/EtOAc: 3/1) to yield **13a** (81 mg, 0.23 mmol, 61%; single diastereoisomer) as a white crystalline solid.

8a-Phenyl-3,3a-dihydro-2*H*-indeno[2,1-*b*]furan-2,8(8a*H*)-dione (13a): M. p. = 130 - 132 °C. R_f = 0.2 (pet.ether/EtOAc: 3/1) ^{1}H-NMR (400 MHz, $CDCl_3$): δ = 7.90 (d, J = 7.9 Hz, 1H), 7.80 (dd, J = 7.5, 1.4 Hz, 1H), 7.59 – 7.55 (m, 2H), 7.43 – 7. 34 (m, 5H), 4.24 (dd, J = 10.8, 3.3 Hz, 1H), 3.25 (dd, J = 18.2, 10.9 Hz, 1H), 2.79 (dd, J = 18.2, 3.3 Hz, 1H). ^{13}C-NMR (101 MHz, $CDCl_3$): δ = 197.8 (C_q), 174.4 (C_q), 153.0 (C_q), 137.3 (C_q), 136.5 (CH), 133.9 (C_q), 129.9 (CH), 129.1 (CH), 129.0 (CH), 126.2 (CH), 126.1 (CH), 125.0 (CH), 90.3 (C_q), 46.9 (CH), 35.0 (CH_2). IR (neat): 1786, 1714, 1602, 1163, 676, 627, 595 cm^{-1}. MS (ESI) *m/z* (relative intensity): 287.1 (10) $[M+Na]^+$. HR-MS (ESI) *m/z* calcd for $C_{17}H_{13}O_3$ $[M+H]^+$ 265.0859, found 265.0864.

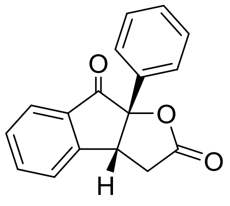

5-Methoxy-8a-phenyl-3,3a-dihydro-2*H*-indeno[2,1-*b*]furan-2,8(8a*H*)-dione (13b): Representative procedure C was followed using ketone **4b** (113 mg, 0.50 mmol, 1.00 equiv) for 24 h (8h + 16h). Purification by column chromatography (pet.ether/EtOAc: 3/1)

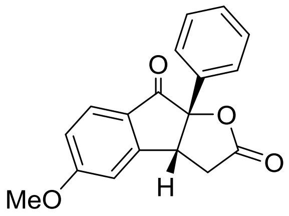

gave **13b** (85 mg, 0.29 mmol, 58%) as a white solid. M. p. = 170 – 172 °C. R_f = 0.15 (pet.ether/EtOAc: 3/1)) ^{1}H-NMR (400 MHz, $CDCl_3$): *δ* = 7.80 (d, *J* = 8.4 Hz, 1H), 7.36 – 7. 32 (m, 5H), 7.05 (dd, *J* = 8.9, 2.6 Hz, 1H), 6.97 (d, *J* = 2.2 Hz, 1H), 4.15 (dd, *J* = 10.7, 3.4 Hz, 1H), 3.94 (s, 3H), 3.21 (dd, *J* = 18.7, 10.7 Hz, 1H), 2.76 (dd, *J* = 18.7, 3.4 Hz, 1H). ^{13}C-NMR (101 MHz, $CDCl_3$): *δ* = 196.0 (C_q), 174.5 (C_q), 167.3 (C_q), 156.2 (C_q), 137.0 (C_q), 129.0 (CH), 128.8 (CH), 127.8 (CH), 126.8 (CH), 124.9 (C_q), 117.6 (CH), 109.5 (CH), 90.9 (C_q), 56.1 (OCH_3), 46.8 (CH), 35.0 (CH_2). IR (neat): 1775, 1710, 1602, 1119, 694, 614, 5962 cm^{-1}. MS (ESI) *m/z* (relative intensity): 317.1 (40) $[M+Na]^+$. HR-MS (ESI) *m/z* calcd for $C_{18}H_{14}O_4Na_1$ $[M+Na]^+$ 317.0784, found 317.0778.

5-Chloro-8a-phenyl-3,3a-dihydro-2*H*-indeno[2,1-*b*]furan-2,8(8a*H*)-dione (13c): Representative procedure C was followed using ketone **4c** (115 mg, 0.50 mmol, 1.00 equiv) for 24 h (8h + 16h). Purification by column chromatography (pet.ether/EtOAc: 4/1) gave **13c** (92 mg, 0.31 mmol, 62%) as light yellow solid. M. p. = 144 - 146 °C. R_f = 0.17 (pet.ether/EtOAc: 3/1) ^{1}H-NMR (400 MHz, $CDCl_3$): *δ* = 7.81 (d, *J* = 8.3 Hz, 1H), 7.57 (bd, 1H), 7.54 (ddd, *J* = 8.2, 1.8, 0.9 Hz, 1H), 7.40 – 7.35 (m, 3H), 7.33 – 7.31 (m 2H), 4.21 (dd, *J* = 10.7, 3.2 Hz, 1H), 3.24 (dd, *J* = 18.4, 10.7 Hz, 1H), 2.75 (dd, *J* = 18.4, 3.2 Hz, 1H). ^{13}C-NMR (101 MHz, $CDCl_3$): *δ* = 196.5 (C_q), 173.9 (C_q), 156.4 (C_q), 143.8 (C_q), 136.0 (C_q), 132.3 (C_q), 130.7 (CH), 129.1 (CH), 129.0 (CH), 127.1 (CH), 126.6 (CH), 125.0 (CH), 90.2 (C_q), 46.5 (CH), 34.7 (CH_2). IR (neat): 1791, 1718, 1597, 1187, 689, 632, 577 cm^{-1}. MS (ESI) *m/z* (relative intensity): 321.1 (50) $[M+Na]^+$. HR-MS (ESI) *m/z* calcd for $C_{17}H_{11}O_3Cl_1Na_1$ $[M+Na]^+$ 321.0289, found 321.0294.

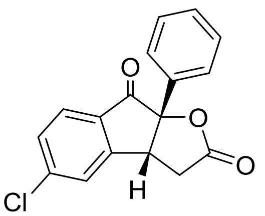

6-Methyl-8a-phenyl-3,3a-dihydro-2*H*-indeno[2,1-*b*]furan-2,8(8a*H*)-dione (13d): Representative procedure C was followed using ketone **4d** (105 mg, 0.50 mmol, 1.00 equiv) for 32 h (16h + 16h). Purification by column chromatography (pet.ether/EtOAc: 3/1) gave **13d** (67 mg, 0.24 mmol, 48%) as a yellow crystalline solid. M. p. = 156 – 158 °C. R_f = 0.28 (pet.ether/EtOAc: 3/1) ^{1}H-NMR (400 MHz, $CDCl_3$): *δ* = 7.67 (s, 1H), 7.61 (d, *J* = 8.0 Hz, 1H), 7.45 (d, *J* = 8.0 Hz, 1H), 7.43 – 7.33 (m, 5H), 4.19 (dd, *J* = 10.6, 3.3 Hz, 1H), 3.21 (dd, *J* = 18.6, 10.6 Hz, 1H), 2.74 (dd, *J* = 18.6, 3.3 Hz, 1H), 2.46 (s, 3H). ^{13}C-NMR (101 MHz, $CDCl_3$): *δ* = 198.0 (C_q), 174.5 (C_q), 150.5 (C_q), 140.2 (C_q), 138.5 (C_q), 136.7 (CH), 134.0 (CH), 128.9 (CH), 125.9 (CH), 125.8 (CH), 125.0 (CH), 90.7 (C_q), 46.6 (CH), 35.1 (CH_2), 21.3 (CH_3). IR (neat): 1783, 1717, 1615, 1193, 681, 627, 565 cm^{-1}. MS (ESI) *m/z* (relative intensity): 301.4 (60) $[M+Na]^+$. HR-MS (ESI) *m/z* calcd for $C_{18}H_{15}O_3$ $[M+H]^+$ 279.1016, found 279.1011.

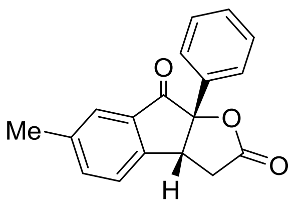

4-Fluoro-8a-phenyl-3,3a-dihydro-2*H*-indeno[2,1-*b*]furan-2,8(8a*H*)-dione (13e): Representative procedure C was followed using ketone **4e** (107 mg, 0.50 mmol, 1.00 equiv) for 32 h (16h + 16h). Purification by column chromatography (pet.ether/EtOAc: 5/1) gave **13e** (81 mg, 0.29 mmol, 57%) as a white solid. M. p. = 184 – 186 °C. R_f = 0.21

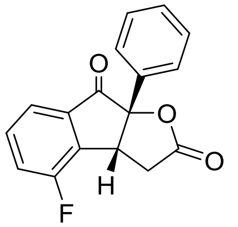

(pet.ether/EtOAc: 3/1) ^{1}H-NMR (400 MHz, $CDCl_3$): δ = 7.73 (d, J = 7.6 Hz, 1H), 7.60 (td, J = 7.6, 4.8 Hz, 1H), 7.48 (dt, J = 9.0, 0.9 Hz, 1H), 7.44 – 7.41 (m, 3H), 7.37 – 7.34 (m, 2H), 4.32 (dd, J = 10.7, 2.9 Hz, 1H), 3.28 (dd, J = 18.7, 10.7 Hz, 1H), 2.90 (dd, J = 18.7, 2.9 Hz, 1H). ^{13}C-NMR (101 MHz, $CDCl_3$): δ = 196.7 (d, J_{CF} = 2.9 Hz, C_q), 174.1 (C_q), 160.0 (d, J_{CF} = 250.8 Hz, C_q), 138.5 (d, J_{CF} = 17.5 Hz, C_q), 136.5 (d, J_{CF} = 3.8 Hz, C_q), 136.0 (C_q), 132.1 (d, J_{CF} = 7.6 Hz, CH), 129.3 (CH), 129.2 (CH), 125.1 (CH), 123.3 (d, J_{CF} = 18.7 Hz, CH), 121.9 (d, J_{CF} = 3.9 Hz, CH), 89.9 (C_q), 43.7 (CH), 33.6 (CH_2). ^{19}F-NMR (470 MHz, $CDCl_3$) δ = (-118.00 – -118.04) (m). IR (neat): 1778, 1726, 1614, 1178, 650, 627, 562 cm^{-1}. MS (ESI) *m/z* (relative intensity): 305.2 (100) $[M+Na]^+$. HR-MS (ESI) *m/z* calcd for $C_{17}H_{11}O_3F_1Na_1$ $[M+Na]^+$ 305.0584, found 305.0574.

7-Methoxy-8a-(*p*-tolyl)-3,3a-dihydro-2*H*-indeno[2,1-*b*]furan-2,8(8a*H*)-dione (13f): Representative procedure C was followed using ketone **4g** (120 mg, 0.50 mmol, 1.00 equiv) for 32 h (16h + 16h). Purification by column chromatography (pet.ether/EtOAc: 4/1 to 2/1) gave **13f** (80 mg, 0.26 mmol, 52%) as a dark brown oil. R_f = 0.15 (pet.ether/EtOAc: 3/1) ^{1}H-NMR (400 MHz, $CDCl_3$): δ = 7.73 (dd, J = 8.4, 7.6 Hz, 1H), 7.28 (t, J = 6.0 Hz, 2H), 7.20 (d, J = 8.0 Hz, 2H), 7.09 (d, J = 7.6 Hz, 2H), 6.97 (d, J = 8.4 Hz, 2H), 4.15 (dd, J = 10.6, 3.3 Hz, 2H), 3.98 (s, 3H), 3.25 (dd, J = 18.4, 10.6 Hz, 2H), 2.77 (dd, J = 18.4, 3.3 Hz, 2H), 2.36 (s, 3H). ^{13}C-NMR (101 MHz, $CDCl_3$): δ = 211.1 (C_q), 195.4 (C_q), 174.6 (C_q), 159.5 (C_q), 155.1 (C_q), 138.7 (CH), 133.7 (C_q), 129.5 (CH), 125.1 (CH), 122.0 (C_q), 117.5 (CH), 111.2 (CH), 90.6 (C_q), 56.1 (OCH_3), 46.2 (CH), 35.2 (CH_2), 21.2 (CH_3). IR (neat): 1778, 1715, 1591, 1174, 684, 638, 563 cm^{-1}. MS (ESI) *m/z* (relative intensity): 331.4 (100) $[M+Na]^+$. HR-MS (ESI) *m/z* calcd for $C_{19}H_{16}O_4Na_1$ $[M+Na]^+$ 331.0941, found 331.0948.

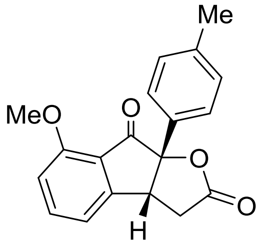

7-Fluoro-8a-phenyl-3,3a-dihydro-2*H*-indeno[2,1-*b*]furan-2,8(8a*H*)-dione (13g): Representative procedure C was followed using ketone **4h** (107 mg, 0.50 mmol, 1.00 equiv) for 32 h (16h + 16h). Purification by column chromatography (pet.ether/EtOAc: 5/1 to 3/1) gave **13g** (38 mg, 0.13 mmol, 28%) as a white solid. M. p. = 144 – 146 °C. R_f = 0.22 (pet.ether/EtOAc: 3/1). ^{1}H-NMR (400 MHz, $CDCl_3$): δ = 7.78 (td, J = 8.1, 5.9 Hz, 1H), 7.42 – 7. 33 (m, 6H), 7.18 (t, J = 8.8 Hz, 1H), 4.25 (dd, J = 10.5, 3.1 Hz, 1H), 3.25 (dd, J = 18.7, 10.5 Hz, 1H), 2.78 (dd, J = 18.7, 3.1 Hz, 1H). ^{13}C-NMR (101 MHz, $CDCl_3$): δ = 194.0 (d, J_{CF} = 2.0 Hz, C_q), 173.9 (C_q), 160.0 (d, J_{CF} = 269.3 Hz, C_q), 154.5 (d, J_{CF} = 1.5 Hz, C_q), 139.3 (d, J_{CF} = 8.5 Hz, C_q), 136.0 (C_q), 129.1 (CH), 129.0 (CH), 125.1 (CH), 122.1 (q, J_{CF} = 14.0 Hz, CH), 116.7 (d, J_{CF} = 18.6 Hz, CH), 90.4 (C_q), 46.6 (d, J_{CF} = 1.7 Hz, CH), 35.0 (CH_2). ^{19}F-NMR (470 MHz, $CDCl_3$) δ = (-110.4 – -110.5) (m). IR (neat): 1778, 1733, 1614, 1185, 690, 636, 594 cm^{-1}. MS (ESI) *m/z* (relative intensity): 305.3 (20) $[M+Na]^+$. HR-MS (ESI) *m/z* calcd for $C_{17}H_{12}O_3F_1$ $[M+H]^+$ 283.0765, found 283.0777.

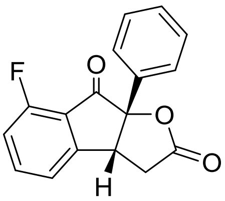

8a-(4-Fluorophenyl)-7-methyl-3,3a-dihydro-2*H*-indeno[2,1-*b*]furan-2,8(8a*H*)-dione (13h):

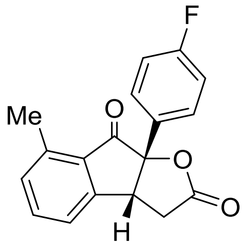

Representative procedure C was followed using ketone **4i** (114 mg, 0.50 mmol, 1.00 equiv) for 32 h (16h + 16h). Purification by column chromatography (pet.ether/EtOAc: 4/1) gave **13h** (44.5 mg, 0.15 mmol, 30%) as a yellow crystalline solid. M. p. = 128 – 130 °C. R_f = 0.28 (pet.ether/EtOAc: 3/1) ^{1}H-NMR (400 MHz, $CDCl_3$): δ = 7.64 (t, J = 7.5 Hz, 1H), 7.37 – 7.34 (m, 3H), 7.31 (dd, J = 7.5, 0.6 Hz, 1H), 7.09 (t, J = 8.8 Hz, 2H), 4.19 (dd, J = 10.6, 3.1 Hz, 1H), 3.25 (dd, J = 18.2, 10.6 Hz, 1H), 2.77 (dd, J = 18.2, 3.1 Hz, 1H), 2.65 (s, 3H). ^{13}C-NMR (101 MHz, $CDCl_3$): δ = 198.3 (C_q), 174.3 (C_q), 163.2 (d, J_{CF} = 247.0 Hz, C_q), 153.5 (C_q), 141.6 (C_q), 136.6 (CH), 132.5 (d, J_{CF} = 3.2 Hz, C_q), 131.6 (CH), 131.2 (C_q), 127.2 (d, J_{CF} = 8.4 Hz, CH), 123.4 (CH), 116.1 (d, J_{CF} = 21.8 Hz, CH), 89.9 (C_q), 45.9 (CH), 35.2 (CH_2), 18.6 (CH_3). ^{19}F-NMR (376 MHz, $CDCl_3$) δ = (-112.75 – -112.81) (m). IR (neat): 1776, 1719, 1589, 1180, 697, 589, 553 cm^{-1}. MS (ESI) *m/z* (relative intensity): 319.4 (95) $[M+Na]^+$. HR-MS (ESI) *m/z* calcd for $C_{18}H_{17}O_3N_1F_1$ $[M^{+}+NH_4]$ 314.1187, found 314.1194.

8a-([1,1'-Biphenyl]-4-yl)-7-methyl-3,3a-dihydro-2*H*-indeno[2,1-*b*]furan-2,8(8a*H*)-dione (13i):

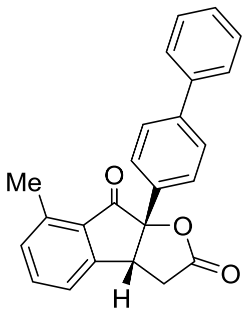

Representative procedure C was followed using ketone **4j** (144 mg, 0.50 mmol, 1.00 equiv) for 46 h (30h + 16h). Purification by column chromatography (pet.ether/EtOAc: 5/1) gave **13i** (65 mg, 0.18 mmol, 37%) as an orange solid. M. p. = 158 – 160 °C. R_f = 0.3 (pet.ether/EtOAc: 3/1) ^{1}H-NMR (500 MHz, $CDCl_3$): δ = 7.65 (t, J = 8.0 Hz, 1H), 7.62 (d, J = 8.0 Hz, 2H), 7.57 (dd, J = 6.9, 1.3 Hz, 2H), 7.45 (d, J = 8.0 Hz, 2H), 7.44 (t, J = 6.3 Hz, 2H), 7.39 (d, J = 7.9 Hz, 1H), 7.36 (dt, J = 8.0, 1.9 Hz, 1H), 7.32 (d, J = 7.6 Hz, 1H), 4.25 (dd, J = 10.5, 3.2 Hz, 1H), 3.27 (dd, J = 18.5, 10.5 Hz, 1H), 2.78 (dd, J = 18.5, 3.2 Hz, 1H), 2.68 (s, 3H). ^{13}C-NMR (125 MHz, $CDCl_3$): δ = 198.5 (C_q), 174.5 (C_q), 153.7 (C_q), 141.9 (C_q), 141.6 (C_q), 140.5 (C_q), 135.7 (C_q), 131.6 (CH), 131.4 (CH), 129.0 (CH), 127.8 (CH), 127.7 (CH), 127.3 (CH), 125.7 (CH), 123.4 (CH), 90.3 (C_q), 46.1 (CH), 35.4 (CH_2), 18.6 (CH_3). IR (neat): 1775, 1711, 1593, 1199, 691, 621, 561 cm^{-1}. MS (ESI) *m/z* (relative intensity): 377.3 (100) $[M+Na]^+$. HR-MS (ESI) *m/z* calcd for $C_{24}H_{18}O_3Na_1$ $[M+Na]^+$ 377.1148, found 377.1150.

8a-(2-Methoxyphenyl)-3,3a-dihydro-2*H*-indeno[2,1-*b*]furan-2,8(8a*H*)-dione (13j): Representative

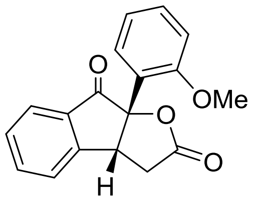

procedure C was followed using ketone **4m** (113 mg, 0.50 mmol, 1.00 equiv) for 32 h (16h + 16h). Purification by column chromatography (pet.ether/EtOAc: 3/1) gave **13j** (84 mg, 0.285 mmol, 57%) as a light yellow solid. M. p. = 198 - 200 °C. R_f = 0.17 (pet.ether/EtOAc: 3/1) ^{1}H-NMR (500 MHz, $CDCl_3$): δ = 7.89 (d, J = 7.4 Hz, 1H), 7.75 (dt, J = 7.6, 0.9 Hz, 1H), 7.55 – 7.49 (m, 3H), 7.37 (dt, J = 7.5, 1.7 Hz, 1H), 7.06 (dt, J = 7.6, 0.9 Hz, 1H), 6.88 (d, J = 8.3 Hz, 1H), 4.16 (dd, J = 10.5, 1.9 Hz, 1H), 3.51 (s, 3H), 3.10 (dd, J = 18.5, 10.5 Hz, 1H), 2.79 (dd, J = 18.5, 1.9 Hz, 1H). ^{13}C-NMR (125 MHz, $CDCl_3$): δ = 197.9 (C_q), 175.0 (C_q), 155.4 (C_q), 152.0 (C_q), 136.4 (C_q), 134.8 (C_q), 130.2 (CH), 129.2 (CH), 126.6 (CH), 125.8 (CH), 125.7 (CH), 125.3 (CH), 121.2 (CH), 111.2 (CH), 89.6 (C_q), 55.5 (OCH_3), 45.6 (CH), 34.2 (CH_2). IR

(neat): 1782, 1729, 1603, 1178, 679, 634, 598 cm^{-1}. MS (ESI) *m/z* (relative intensity): 317.2 (30) [M+Na]$^+$. HR-MS (ESI) *m/z* calcd for $C_{18}H_{14}O_4Na_1$ [M+Na]$^+$ 317.0784, found 317.0774.

8a-(4-Fluorophenyl)-3,3a-dihydro-2H-indeno[2,1-*b*]furan-2,8(8a*H*)-dione (13k): The representative procedure C was followed using ketone **4o** (107 mg, 0.50 mmol, 1.00 equiv) for 30 h (14h + 16h). Purification by column chromatography (pet.ether/EtOAc: 4/1) gave **13l** (90 mg, 0.32 mmol, 64%) as a white crystalline solid. M. p. = 134 – 136 °C. R_f = 0.10 (pet.ether/EtOAc: 9/1) ^{1}H-NMR (400 MHz, $CDCl_3$): δ = 7.90 (d, *J* = 8.2, Hz, 1H), 7.81 (dt, *J* = 7.5, 1.2 Hz, 1H), 7.58 (t, *J* = 7.5 Hz, 2H), 7.37 – 7.33 (m, 2H), 7.12 – 7.08 (m, 2H), 4.22 (dd, *J* = 10.5, 3.1 Hz, 1H), 3.26 (dd, *J* = 18.4, 10.5 Hz, 1H), 2.89 (dd, *J* = 18.4, 3.1 Hz, 1H). ^{13}C-NMR (101 MHz, $CDCl_3$): δ = 197.5 (C_q), 174.1 (C_q), 163.1 (d, J_{CF} = 248.6 Hz, C_q), 152.8 (C_q), 137.4 (CH), 133.8 (C_q), 132.3 (d, J_{CF} = 3.3 Hz, C_q), 130.0 (CH), 127.2 (d, J_{CF} = 8.6 Hz, CH), 126.3 (CH), 126.2 (CH), 116.1 (d, J_{CF} = 22.1 Hz, CH), 89.8 (C_q), 46.7 (CH), 35.1 (CH_2). ^{19}F-NMR (376 MHz, $CDCl_3$) δ = (-112.50 – -112.57) (m). IR (neat): 1785, 1724, 1611, 1192, 685, 535 cm^{-1}. MS (ESI) *m/z* (relative intensity): 283.1 (50) [M+H]$^+$. HR-MS (ESI) *m/z* calcd for $C_{17}H_{11}O_3Na_1F_1$ [M+Na]$^+$ 305.0584, found 305.0585.

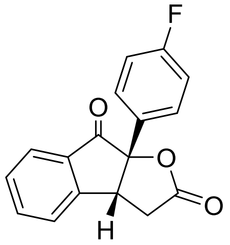

8a-(*p*-Tolyl)-3,3a-dihydro-2*H*-indeno[2,1-*b*]furan-2,8(8a*H*)-dione (13l): Representative procedure C was followed using ketone **4q** (105 mg, 0.50 mmol, 1.00 equiv) for 24 h (8h + 16h). Purification by column chromatography (pet.ether/EtOAc: 3/1) gave **13m** (98 mg, 0.35 mmol, 61%) as light yellow oil. R_f = 0.22 (pet.ether/EtOAc: 3/1) ^{1}H-NMR (400 MHz, $CDCl_3$): δ = 7.80 (d, *J* = 7.6 Hz, 1H), 7.71 (dt, *J* = 7.9, 1.2 Hz, 1H), 7.50 – 7.46 (m, 2H), 7.18 – 7. 11 (m, 4H), 4.15 (dd, *J* = 10.7, 3.3 Hz, 1H), 3.16 (dd, *J* = 18.7, 10.7 Hz, 1H), 2.70 (dd, *J* = 18.7, 3.3 Hz, 1H), 2.27 (s, 3H). ^{13}C-NMR (101 MHz, $CDCl_3$): δ = 199.0 (C_q), 174.5 (C_q), 153.0 (C_q), 138.9 (C_q), 137.2 (CH), 133.9 (C_q), 133.4 (C_q), 129.8 (CH), 129.7 (CH), 126.2 (CH), 126.0 (CH), 125.0 (CH), 90.3 (C_q), 46.7 (CH), 35.0 (CH_2), 21.3 (CH_3). IR (neat): 1783, 1720, 1602, 1161, 676, 637, 548 cm^{-1}. MS (ESI) *m/z* (relative intensity): 301.1 (20) [M+Na]$^+$. HR-MS (ESI) *m/z* calcd for $C_{18}H_{14}O_3Na_1$ [M+Na]$^+$ 301.0835, found 301.0833.

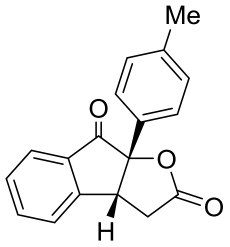

Example not in Scheme 4:

8a-(2,4-Dimethoxyphenyl)-7-methoxy-3,3a-dihydro-2*H*-indeno[2,1-*b*]furan-2,8(8a*H*)-dione (13n): Representative procedure C was followed using ketone **4l** (143 mg, 0.50 mmol, 1.00 equiv) for 32 h (16h + 16h). Purification by column chromatography (pet.ether/EtOAc: 4/1 to 2/1) to yield the title compound (25.4 mg, 0.07 mmol, 20%) as a yellow solid. M. p. = 186 – 188 °C. R_f = 0.12 (pet.ether/EtOAc: 3/1) ^{1}H-NMR (400 MHz, $CDCl_3$): δ = 7.66 (t, *J* = 7.6 Hz, 1H), 7.42 (d, *J* = 8.6 Hz, 1H), 7.00 (d, *J* = 7.6 Hz, 1H), 6.92 (d, *J* = 9.2 Hz, 1H), 6.53 (dd, *J* = 8.6, 2.3 Hz, 1H),

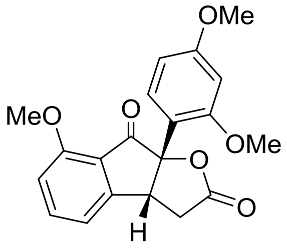

6.45 (d, J = 2.3 Hz, 1H), 4.15 (dd, J = 10.1, 1.9 Hz, 1H), 3.97 (s, 3H), 3.80 (s, 3H), 3.55 (s, 3H), 3.10 (dd, J = 18.0, 10.1 Hz, 1H), 2.74 (dd, J = 18.0, 1.9 Hz, 1H). ^{13}C-NMR (101 MHz, $CDCl_3$): δ = 196.0 (C_q), 174.3 (C_q), 161.6 (C_q), 159.1 (C_q), 156.8 (C_q), 154.5 (C_q), 138.1 (C_q), 127.7 (CH), 122.3 (C_q), 117.9 (CH), 117.3 (CH), 110.8 (CH), 104.2 (CH), 99.4 (CH), 90.0 (C_q), 56.1 (OCH_3), 55.7 (OCH_3), 55.6 (OCH_3), 45.0 (CH), 34.5 (CH_2). IR (neat): 1778, 1721, 1594, 1182, 685, 654, 626 cm^{-1}. MS (ESI) m/z (relative intensity): 375.5 (100) $[M+Na]^+$. HR-MS (ESI) m/z calcd for $C_{20}H_{19}O_6$ $[M+H]^+$ 355.1176, found 355.1175.

Mechanistic Studies

Preparation of (*E*)-Methyl 3-(2-(2-phenylacetyl)phenyl)acrylate (**14**):

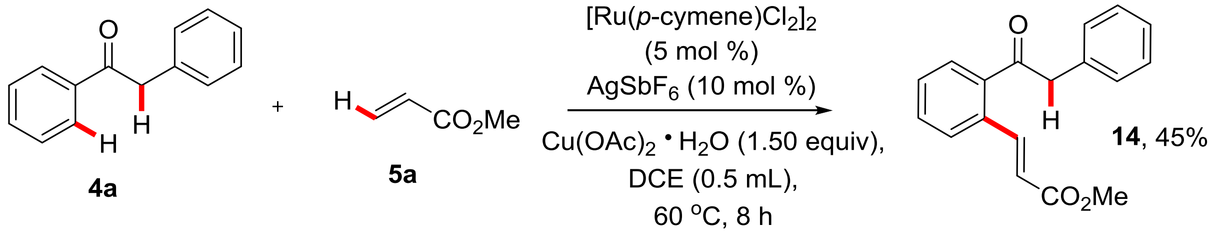

To an oven dried 5 mL microwave vial containing a stir bar was added ketone **4a** (98 mg, 0.50 mmol, 1.00 equiv), Ru[(*p*-cymene)Cl_2]$_2$ (15.2 mg, 5.0 mol%) and $Cu(OAc)_2•H_2O$ (150 mg, 0.75 mmol, 1.50 equiv). The vial was sealed with a rubber septum and purged with N_2. A freshly prepared stock solution of $AgSbF_6$ (17.5 mg, 10 mol%) in dry 1,2-dichloroethane (0.50 mL) was added to this vial and the mixture stirred for 10 min at room temperature. Then, methyl acrylate **5a** (90 µL, 1.00 mmol, 2.00 equiv) was added via syringe and the rubber septum was replaced with an aluminium crimp cap. The vial was tightly sealed and the mixture heated with stirring in a preheated oil bath at 60 °C for 8 h. After 8 hr the mixture was cooled to room temperature, diluted with EtOAc (5 mL) and filtered through a celite pad. The pad was further washed with EtOAc (25 mL) and the combined organic solvent evaporated *in vacuo* to afford a crude mixture. This was purified by column chromatography (silica, pet.ether/EtOAc: 9/1) to yield **14** (63 mg, 0.23 mmol, 45%) as a yellow oil. ^{1}H-NMR (400 MHz, $CDCl_3$): δ = 8.03 (d, J = 15.8 Hz, 1H), 7.78 (dd, J = 7.2, 1.2 Hz, 1H), 7.57 (dd, J = 7.7, 1.5 Hz, 1H), 7.51 (dt, J = 7.5, 1.6 Hz, 1H), 7.45 (td, J = 7.6, 1.6, Hz, 1H), 7.35 – 7.31 (m, 2H), 7.28 – 7.24 (m, 3H), 6.26 (d, J = 15.8 Hz, 1H), 4.24 (s, 2H), 3.79 (s, 3H). ^{13}C-NMR (101 MHz, $CDCl_3$): δ = 200.7 (C_q), 167.0 (C_q), 144.0 (CH), 138.1 (C_q), 135.2 (C_q), 134.0 (C_q), 132.0 (CH), 129.6 (CH), 129.5 (CH), 128.9 (CH), 128.8 (CH), 128.6 (CH), 127.2 (CH), 120.8 (CH), 51.9 (OCH_3), 48.4 (CH_2). MS (ESI) *m/z* (relative intensity): 303.4 (100). HR-MS (ESI) *m/z* calcd for $C_{18}H_{17}O_3$ [M+H]$^+$ 281.1172, found 281.1161.

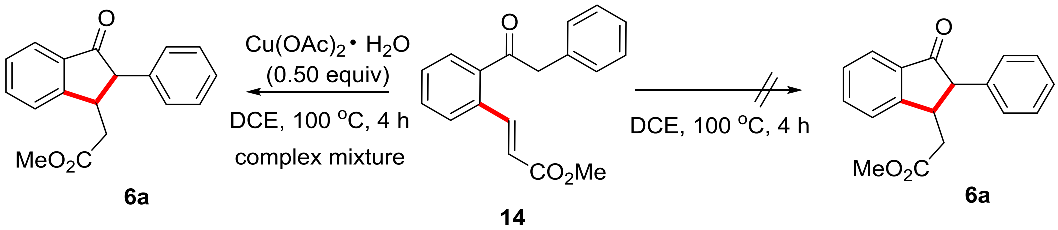

To an oven dried 5 mL microwave vial containing a stirr bar was added **14** (30 mg, 0.107 mmol, 1.00 equiv) in dry 1,2-dichloroethane (0.50 mL) under N_2 and the vial sealed with an aluminium crimp cap. The resulting yellow solution was heated at 100 °C for 4 h and then the vial then cooled to room temperature. The solution was diluted with EtOAc (5 mL) and the resulting solution analysed by LC-MS and TLC. Based on these analyses, no desired monocyclized product **6a** was observed and unreacted starting material was reisolated via column chromatography. (>90%)

The reaction was repeated with the addition of $Cu(OAc)_2•H_2O$ (11 mg, 0.05 mmol, 0.50 equiv) in dry 1,2-dichloroethane (0.5 mL) to the reaction mixture. The crude mixture was diluted with EtOAc (5 mL) and then

passed through a pad of celite, then analysed using LC-MS. Multiple products were observed, including the monocyclized product **6a** (23%), and the tetracycle **7a** (12%).

References

(1) P. Strazzolini, A. G. Giumanini, A. Runcio, M. Scuccato. *J. Org. Chem.* **1998**, *63*, 952-958.

(2) I. Artaud, G. Torossian, P. Viout, *Tetrahedron* **1985**, *41*, 5031-5037.

(3) M. Palucki, S. L. Buchwald, *J. Am. Chem. Soc.* **1997**, *119*, 11108-11109.

(4) F. Churruca, R. SanMartin, I. Tellitu, E. Dominguez, *Tetrahedron Lett.* **2003**, *44,* 5925-5929.

(5) B. Zhao, X. Lu, *Org. Lett.* **2006**, *8,* 5987-5990.

(6) J. Ruan, O. Saidi, J. A. Iggo, J. Xiao, *J. Am. Chem. Soc.* **2008**, *130,* 10510-10511.

(7) L. J. Gooßen, P. Mamone, C. Oppel, *Adv. Synth. Catal.* **2011**, *353,* 57–63.

(8) K. Huang, G. Li, W.-P. Huang, D.-G. Yu, Z.-J. Shi, *Chem. Commun.* **2011**, *47*, 7224-7226.

(9) Y. C. Wong, K. Parthasarathy, C. H. Cheng, *Org. Lett.* **2010**, *12*, 1736-1739.

(10) M. Jacubert, A. Hamze, O. Provot, J.-F. Peyrat, J.-D. Brion, M. Alami, *TetrahedronLett.* **2009**, *50*, 3588-3592.

(11) G. Le Brass, O. Provot, J.-F. Peyrat, M. Alami, J.-D. Brion, *Tetrahedron Lett.* **2006**, *47*, 5497-5501.

(12) A. J. Wommack, D.C. Moebius, A. L. Travis, J. S. Kingsbury, *Org. Lett.* **2009**, *11*, 3202-3205.

(13) M. Lessi, T. Masini, L. Nucara, F. Bellina, R. Rossi, *Adv. Synth. Catal.* **2011**, *353*, 501 – 507.

(14) A. Battace, M. Feuerstein, M. Lemhadri, T. Zair, H. Doucet, M. Santelli, *Eur. J. Org. Chem.* **2007**, 3122–3132.

(15) S. Inaba, R. D. Rieke, *J. Org. Chem.* **1985**, *50*, 1373-1381.

(16) B. Landers, C. Berini, C. Wang, O. Navarro, *J. Org. Chem.* **2011**, *76*, 1390-1397.

(17) M. Padmanaban, A.T. Biju, F. Glorius, *Org. Lett.* **2011**, *13,* 98-101.

(18) G. A. Grasa, T. Colacot, *J. Org. Lett.* **2007**, *9*, 5489-5492.

(19) F. Churruca, R. SanMartin, M. Carril, Tellitu, E. Domınguez, *Tetrahedron*, **2004**, *60*, 2393–2408.

(20) S. K. Chittimalla, T. –C. Chang, T. –C. Liu, H. –P. Hsieh, C. –C. Liao, *Tetrahedron*, **2008**, *64*, 2586-2595.

^{1}H NMR data of compound **4b** (300 MHz, $CDCl_3$)

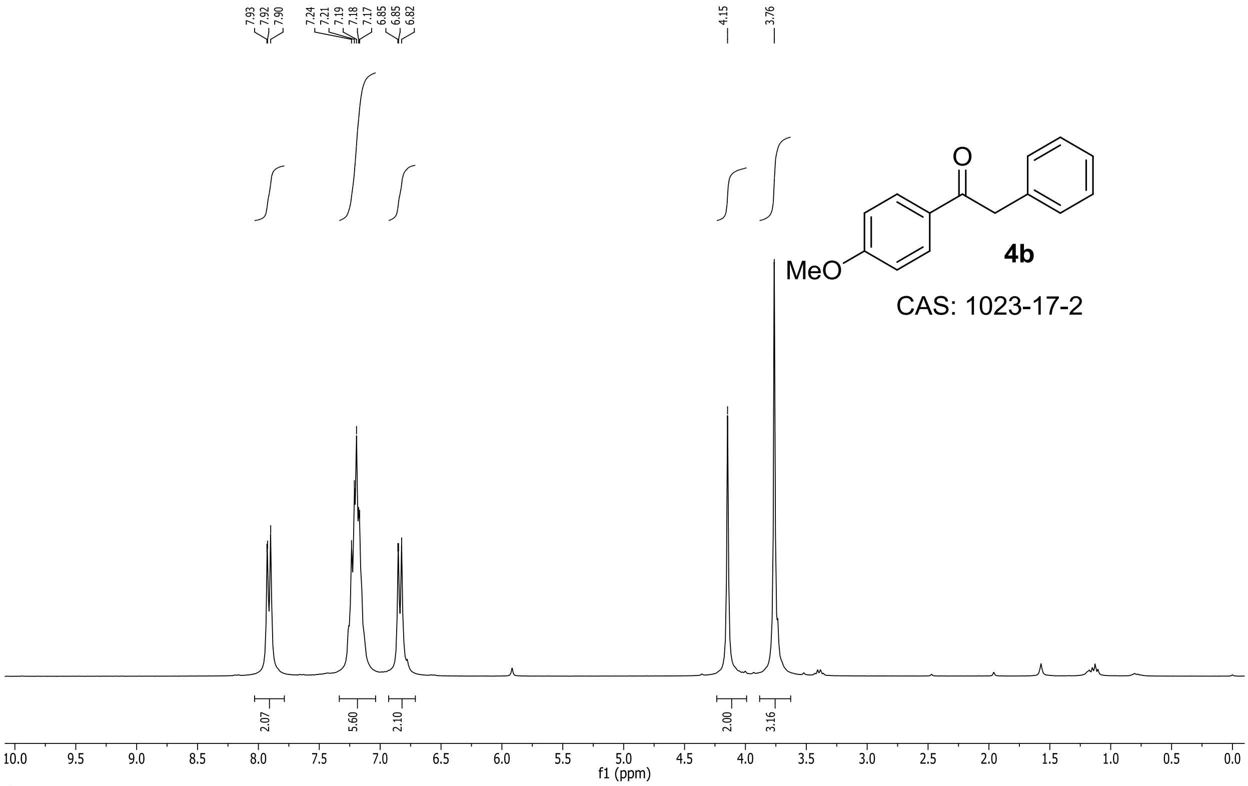

^{1}H NMR data of compound **4d** (300 MHz, $CDCl_3$)

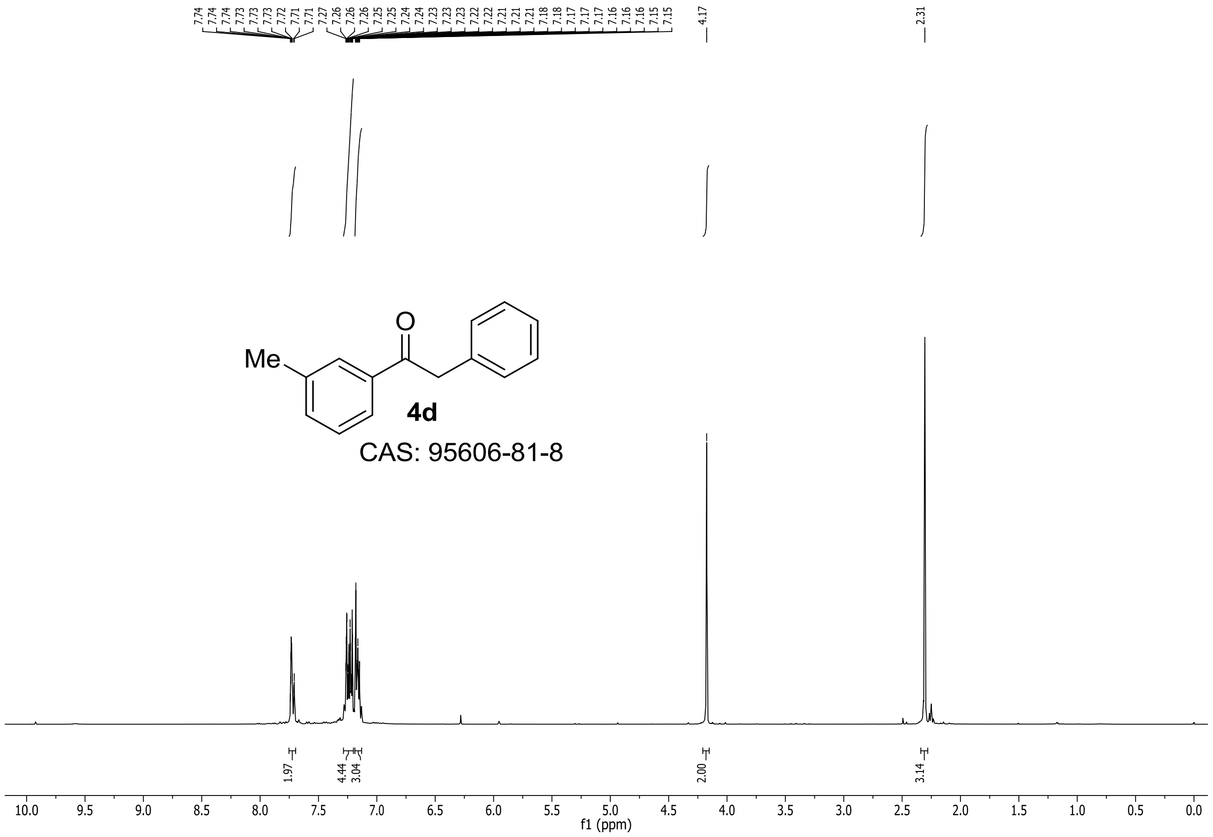

^{1}H NMR data of compound **4e** (300 MHz, $CDCl_3$)

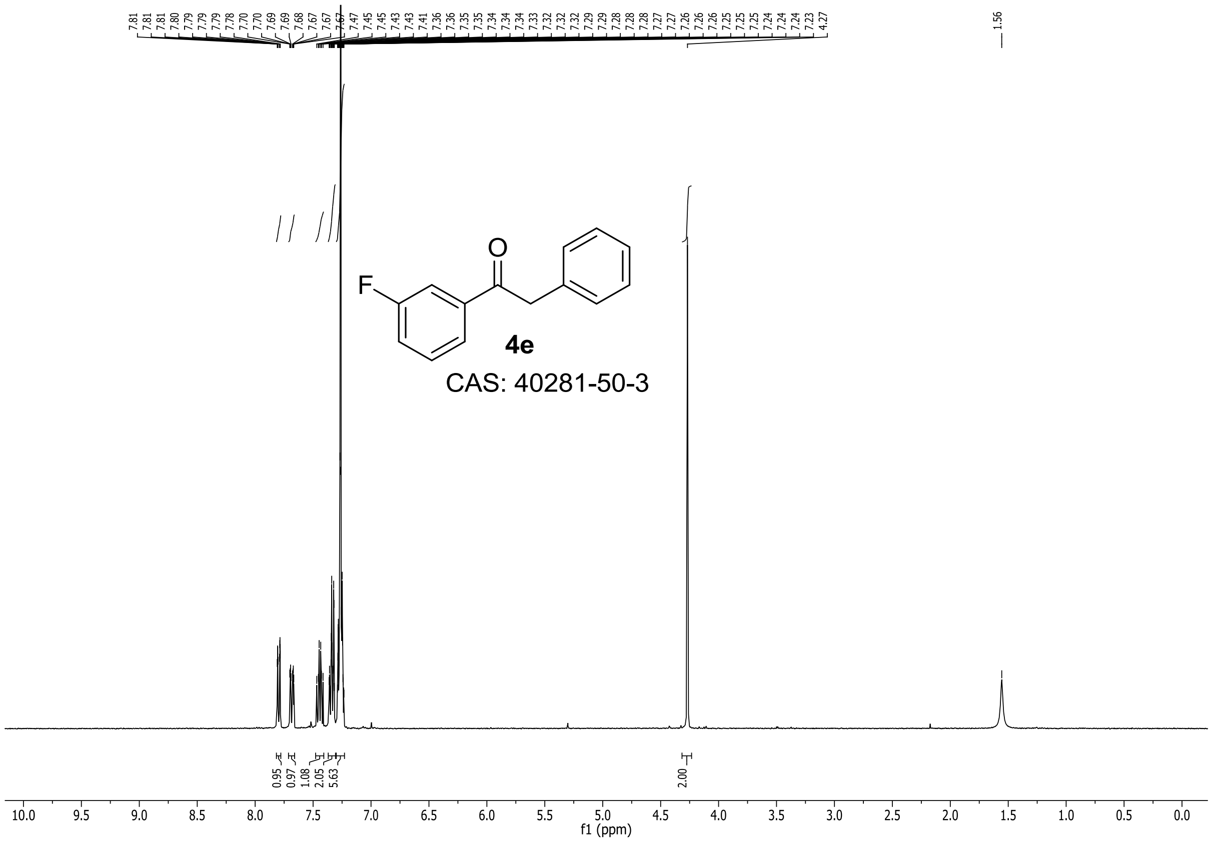

^{1}H NMR data of compound **4f** (300 MHz, $CDCl_3$)

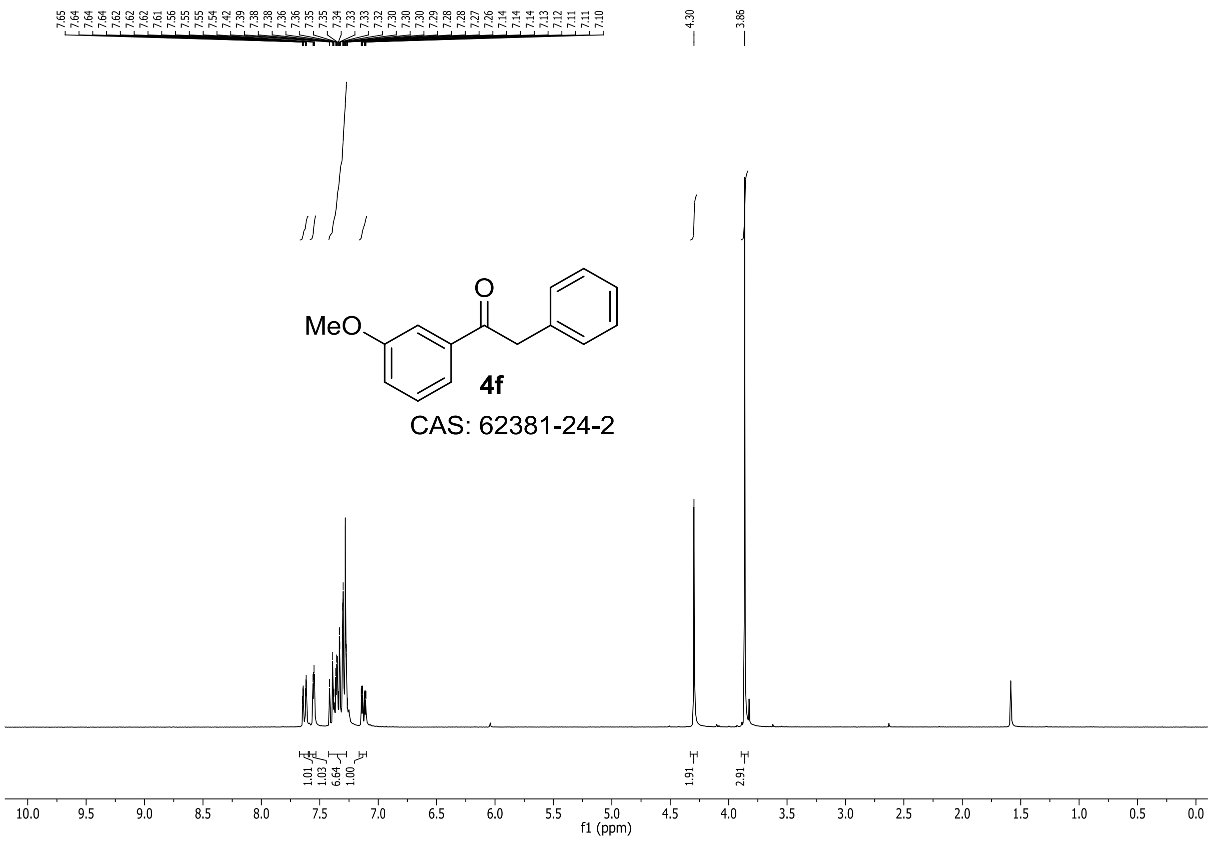

^{1}H NMR data of compound **4g** (300 MHz, $CDCl_3$)

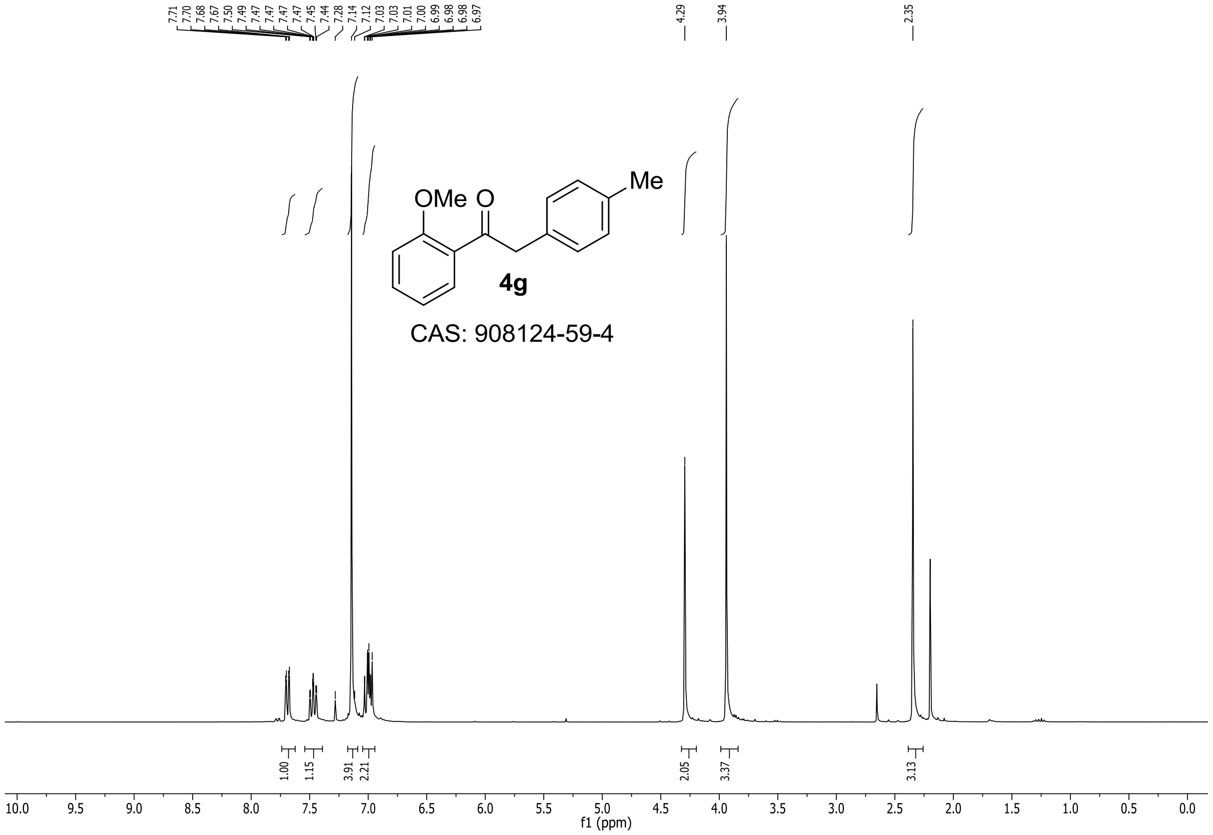

^{1}H NMR data of compound **4h** (300 MHz, $CDCl_3$)

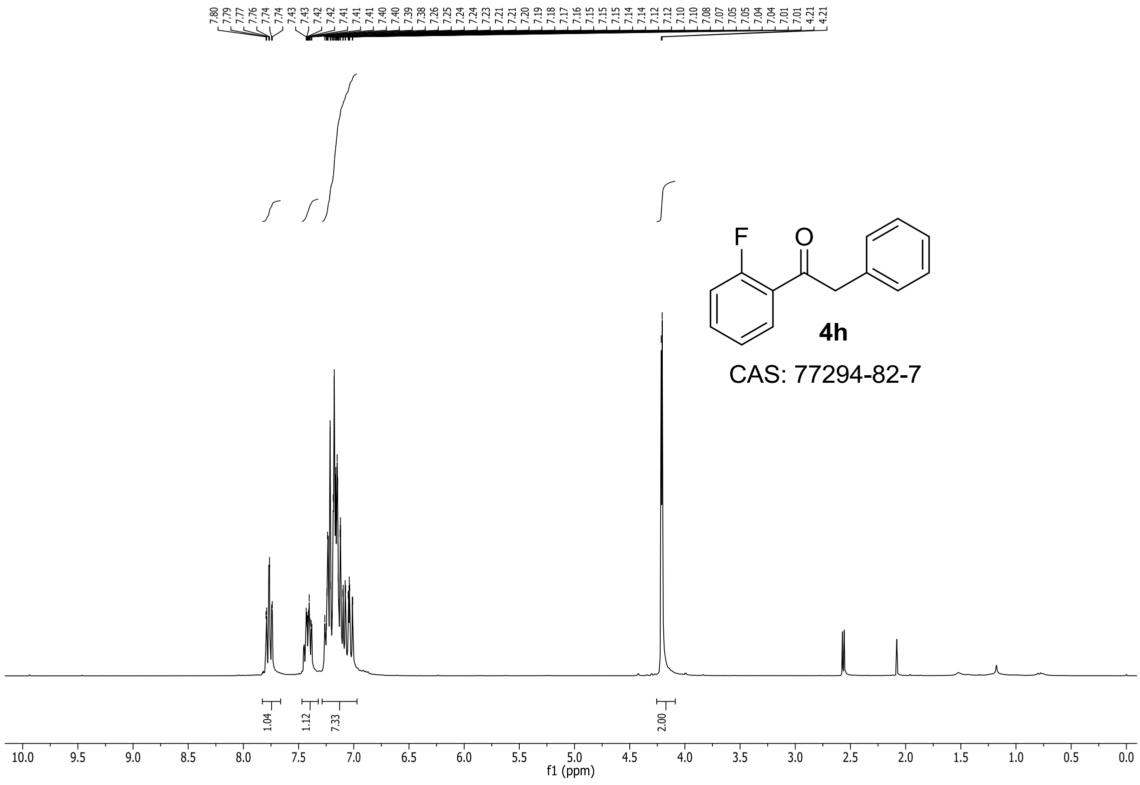

^{1}H,^{13}C and ^{19}F NMR data of compound **4i** (300, 75, 376 MHz, $CDCl_3$)

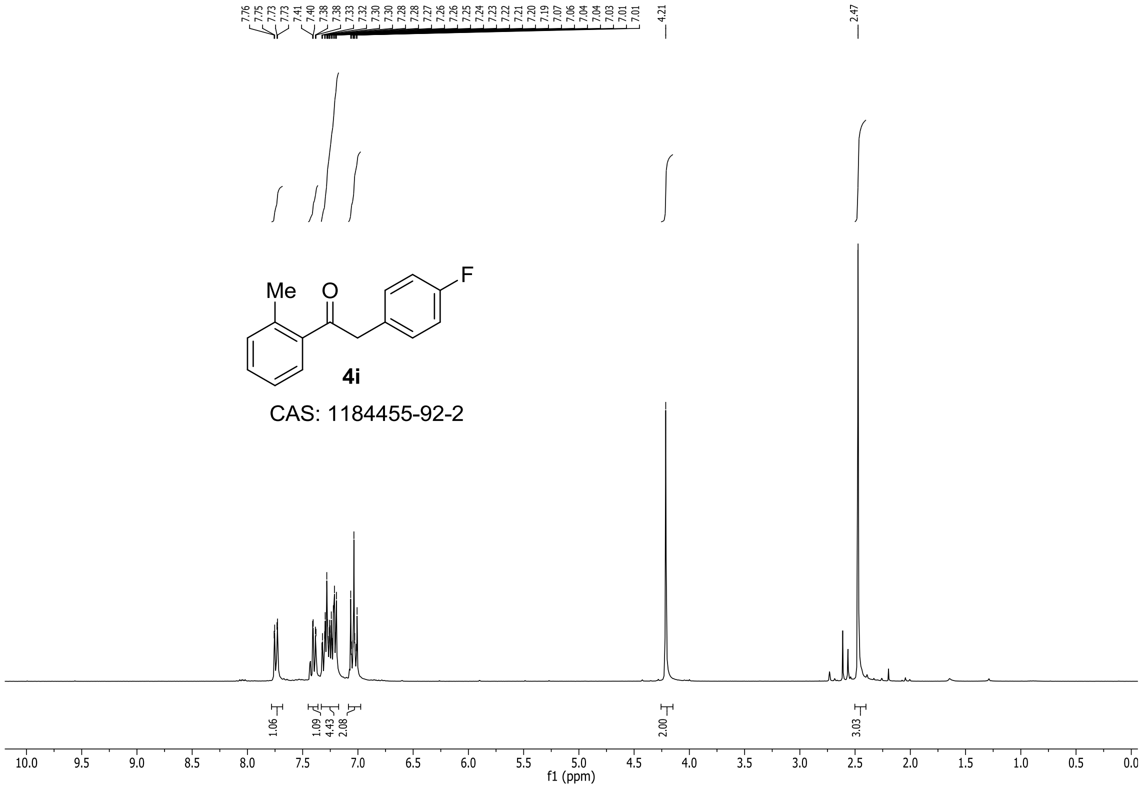

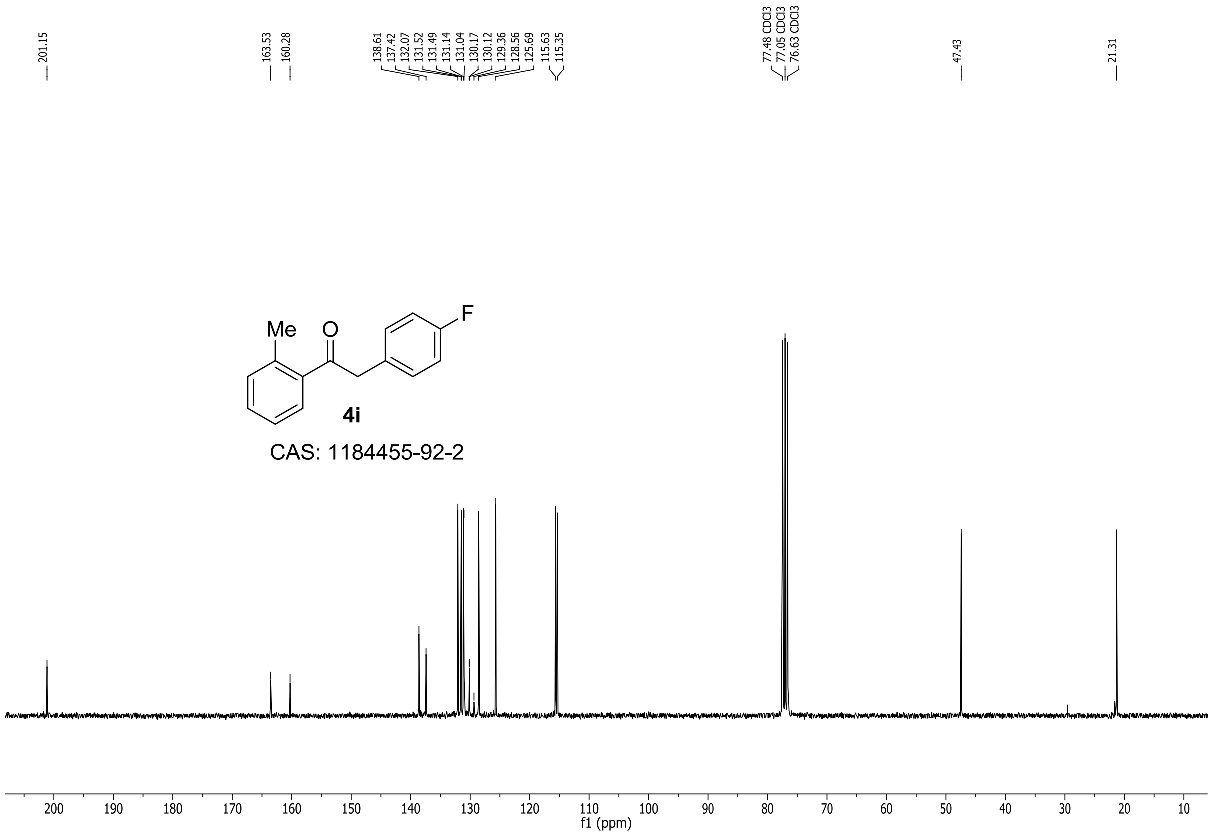

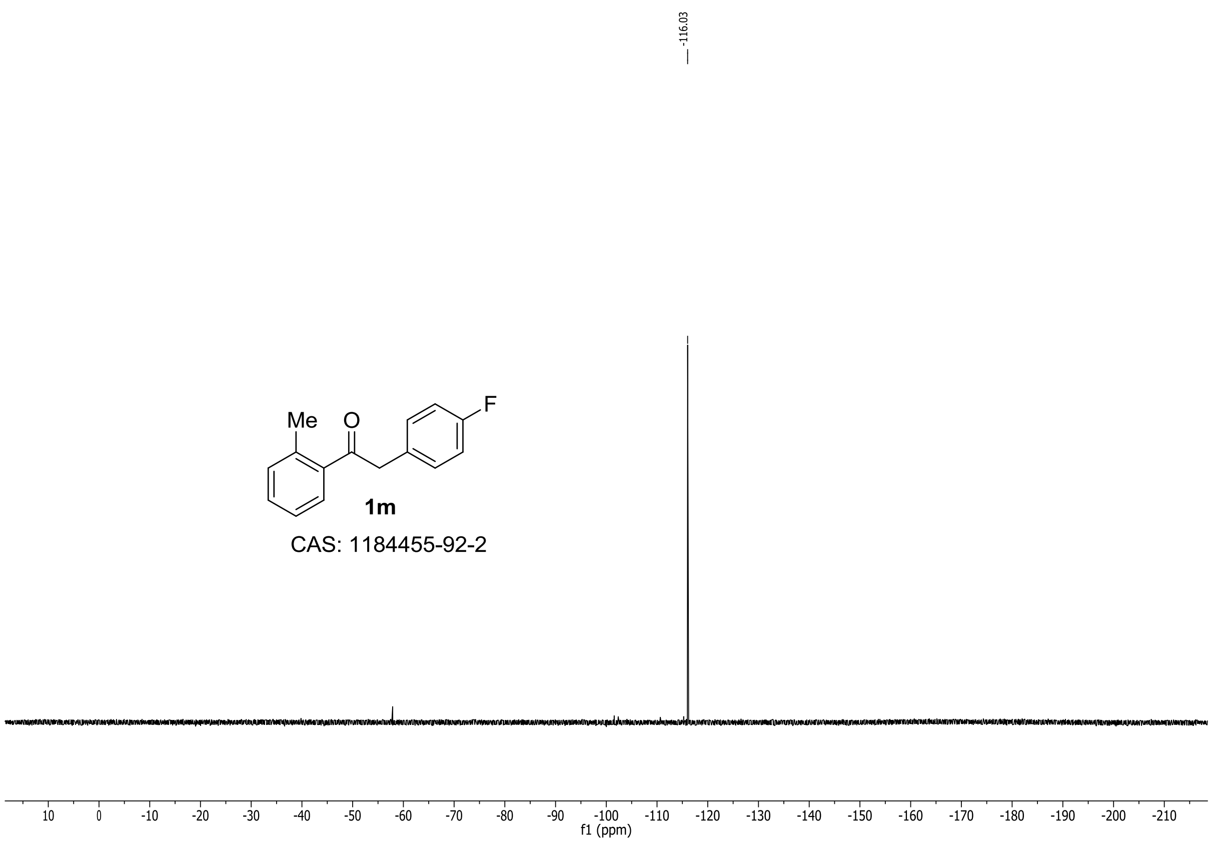

^{1}H and ^{13}C NMR data of compound **4j** (300, 75 MHz, $CDCl_3$)

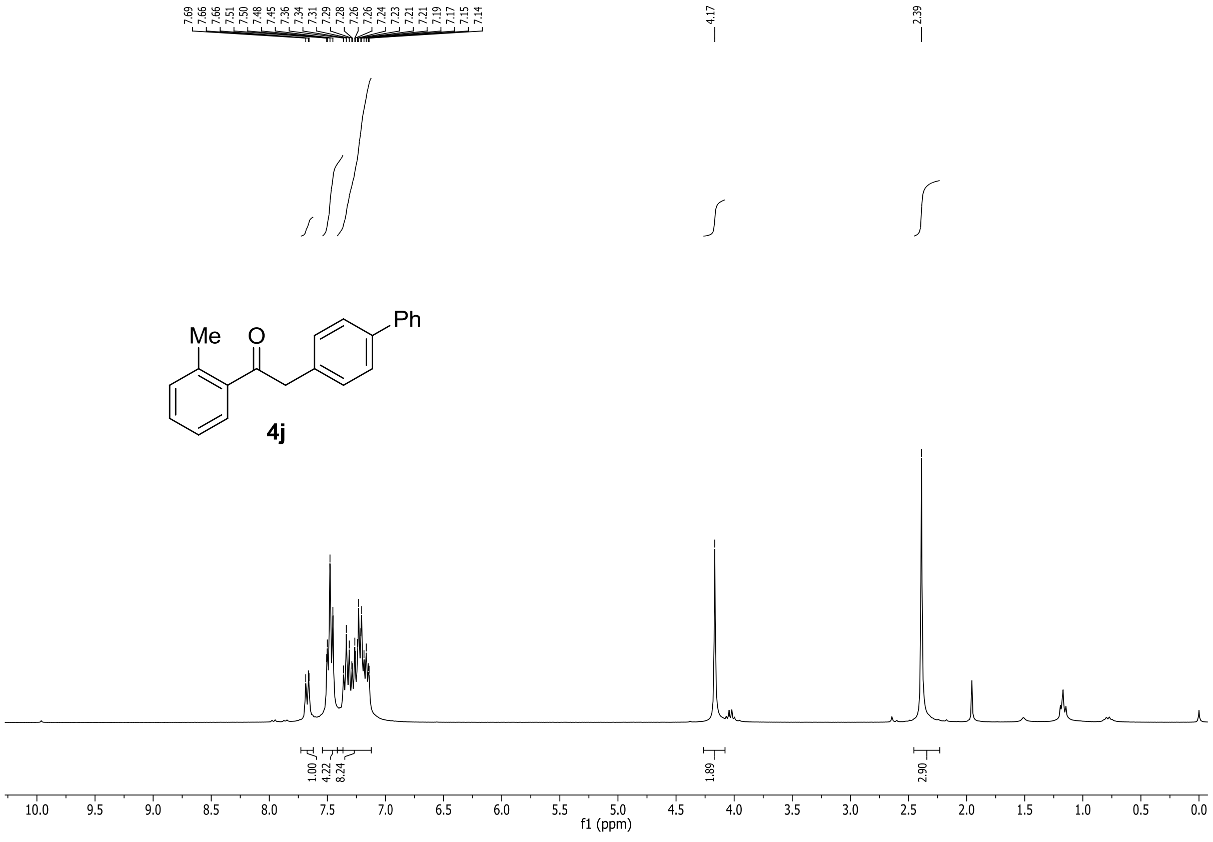

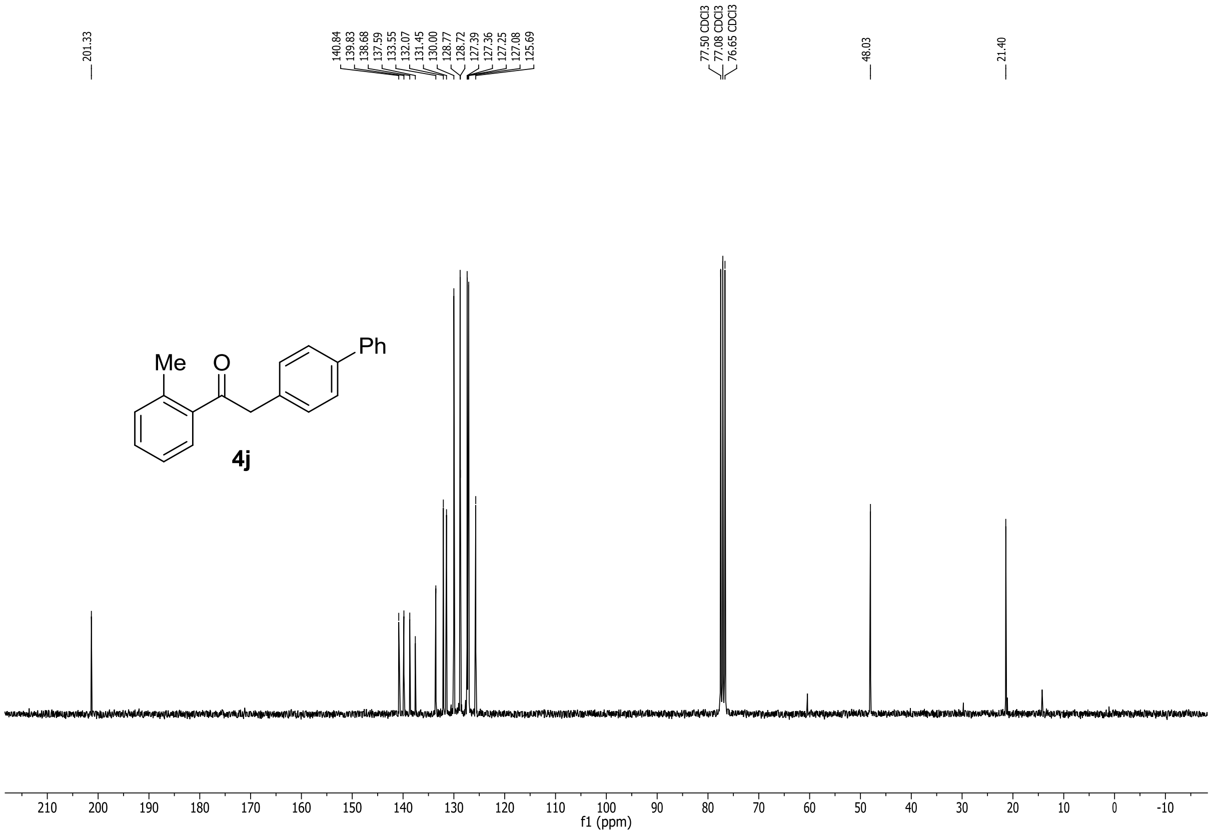

^{1}H NMR data of compound **4k** (300 MHz, $CDCl_3$)

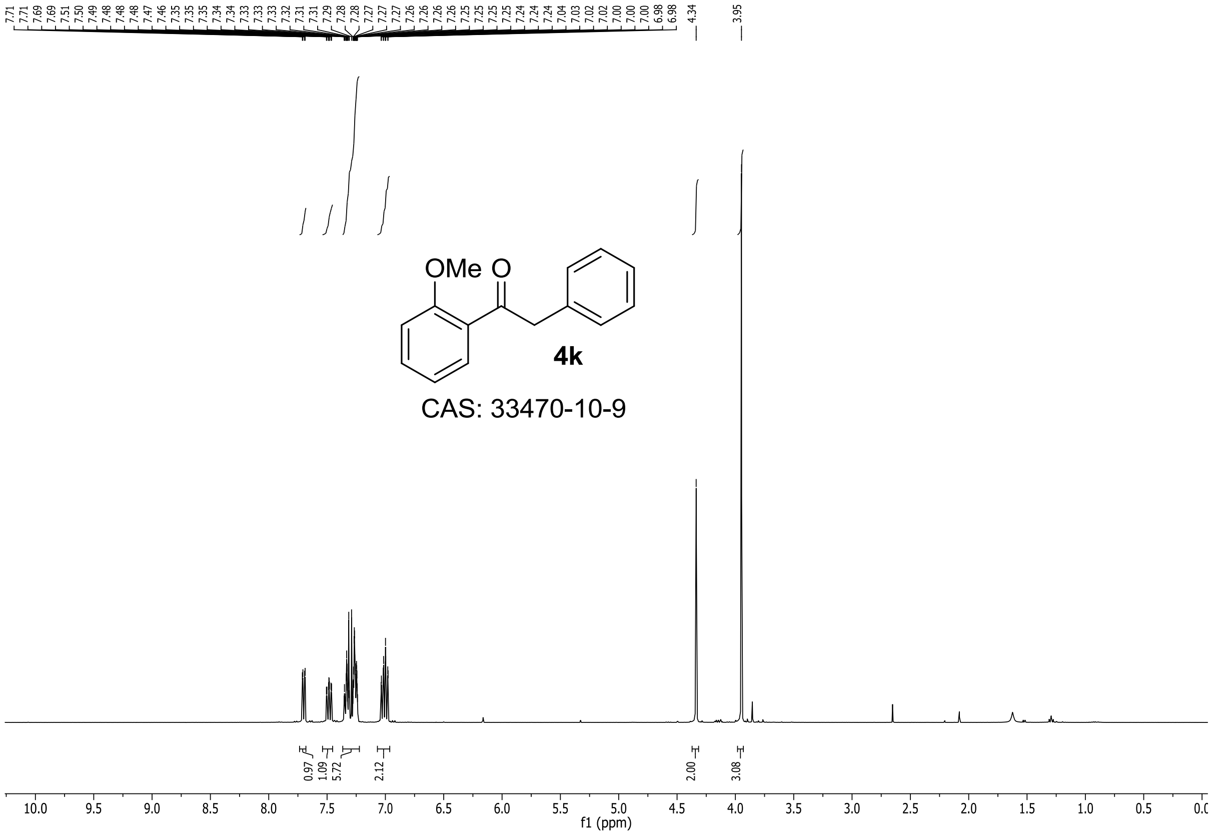

^{1}H and ^{13}C NMR data of compound **4l** (300, 75 MHz, $CDCl_3$)

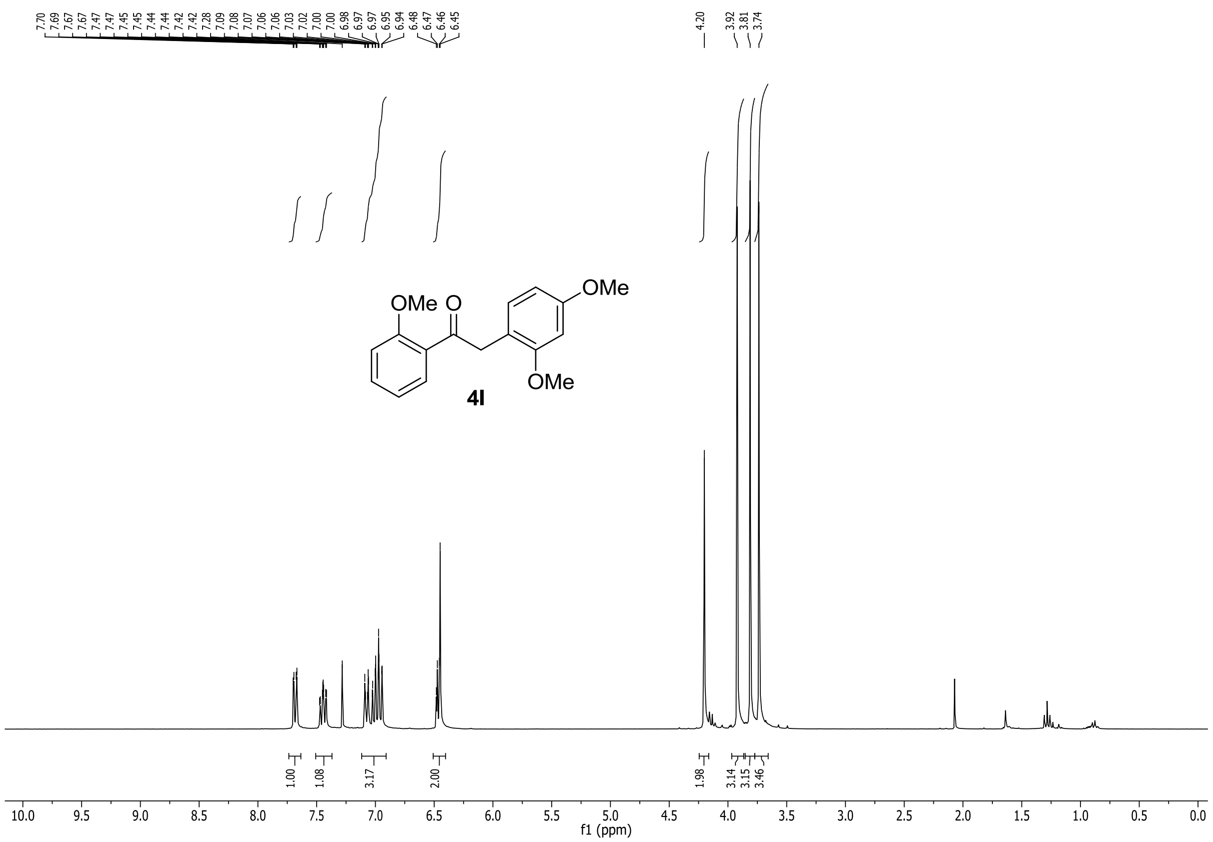
7.70
7.69
7.67
7.67
7.47
7.47
7.45
7.45
7.44
7.42
7.42
7.28
7.09
7.08
7.07
7.06
7.06
7.03
7.02
7.00
7.00
6.98
6.97
6.97
6.95
6.94
6.48
6.47
6.46
6.45
4.20
3.92
3.81
3.74
OMe
O
OMe
OMe
4l
1.00
1.08
3.17
2.00
1.98
3.14
3.15
3.46
f1 (ppm)

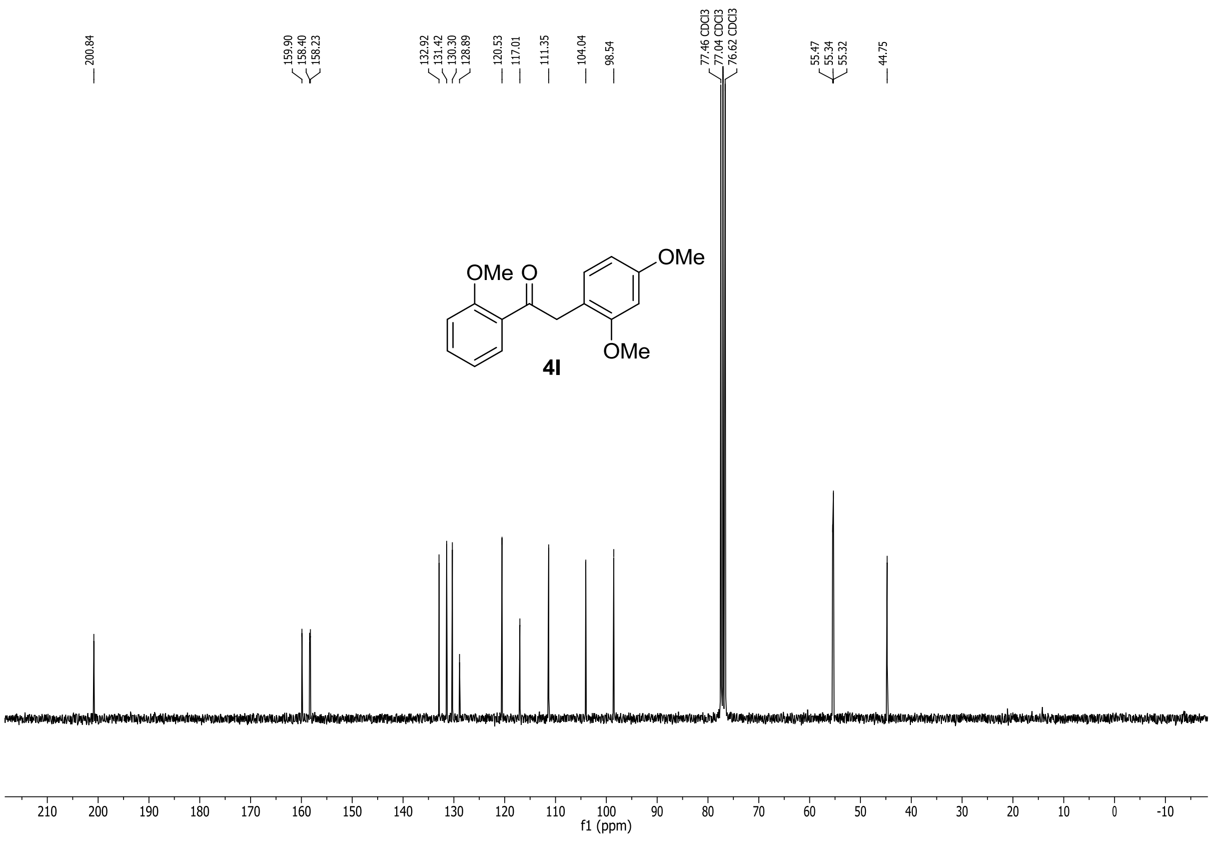
200.84
159.90
158.40
158.23
132.92
131.42
130.30
128.89
120.53
117.01
111.35
104.04
98.54
77.46 CDCl3
77.04 CDCl3
76.62 CDCl3
55.47
55.34
55.32
44.75
OMe
O
OMe
OMe
4l
f1 (ppm)

^{1}H NMR data of compound **4m** (300 MHz, $CDCl_3$)

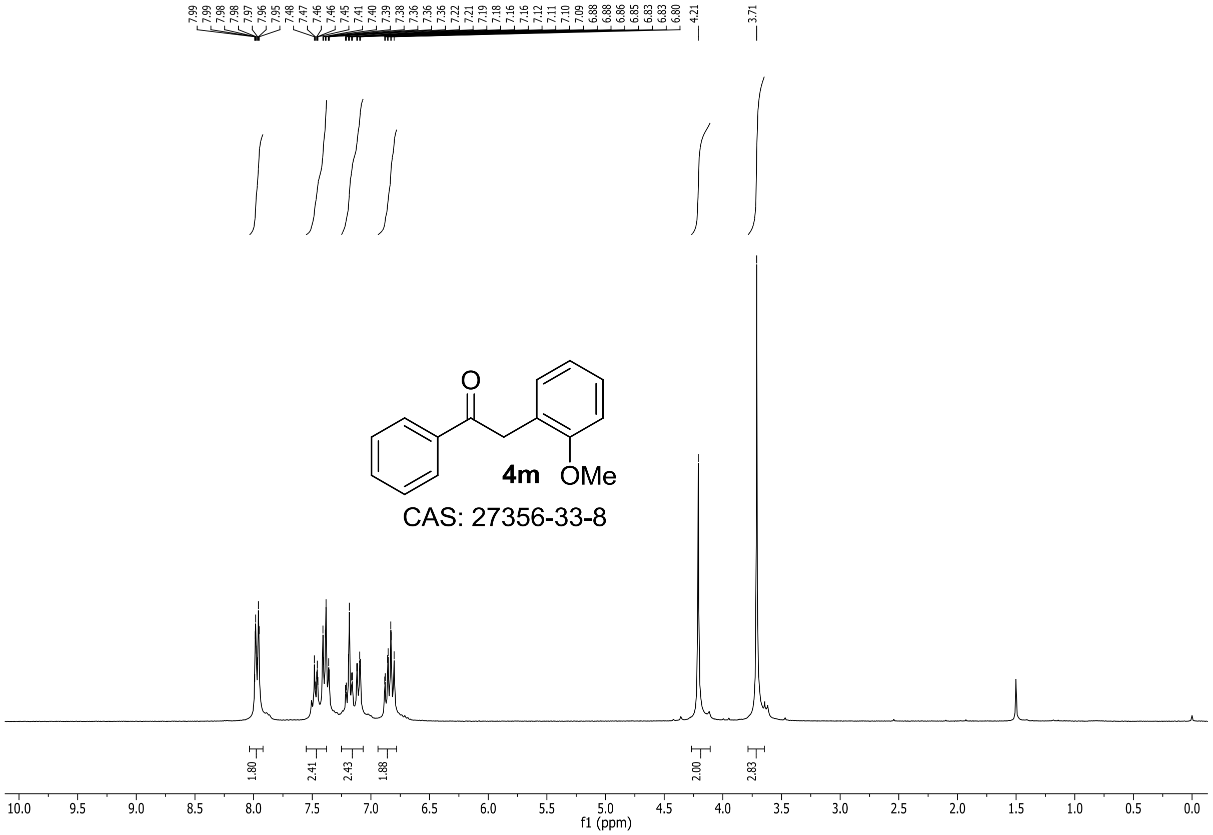

^{1}H NMR data of compound **4n** (300 MHz, $CDCl_3$)

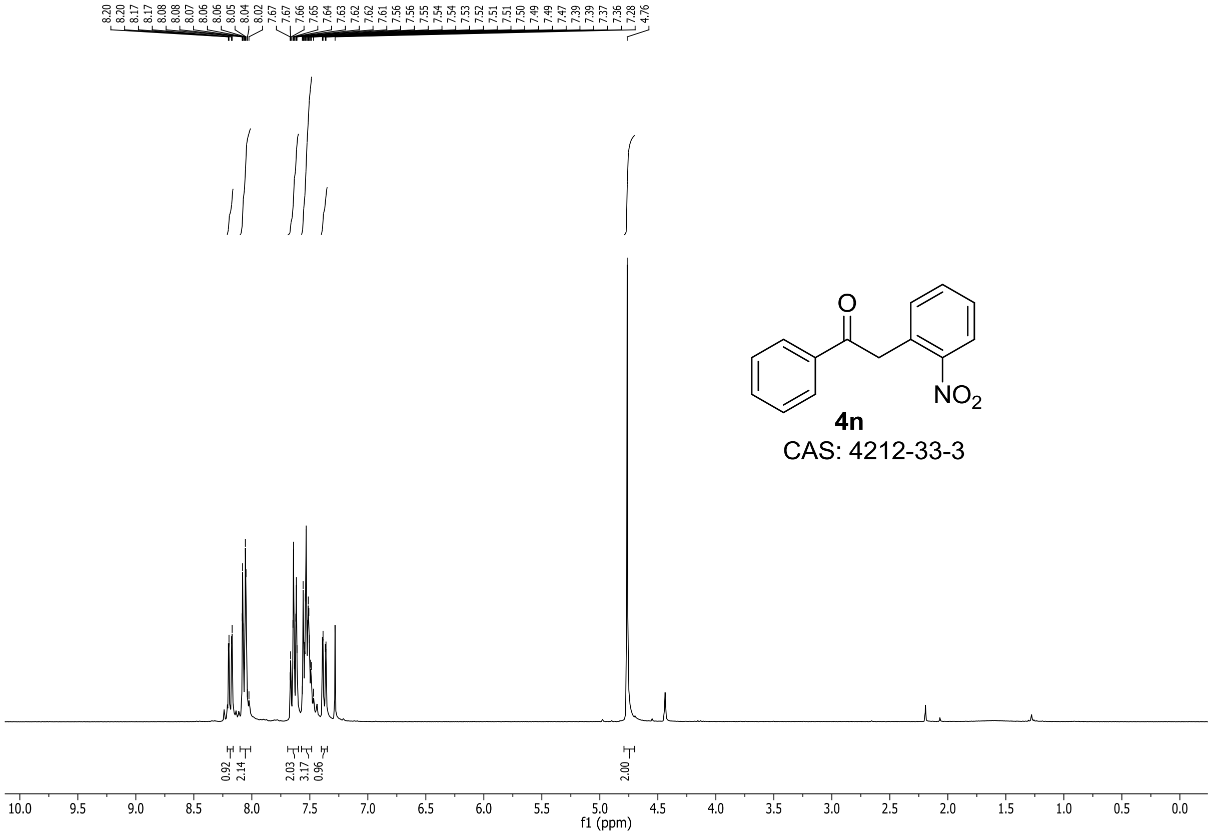

^{1}H NMR data of compound **4o** (300 MHz, $CDCl_3$)

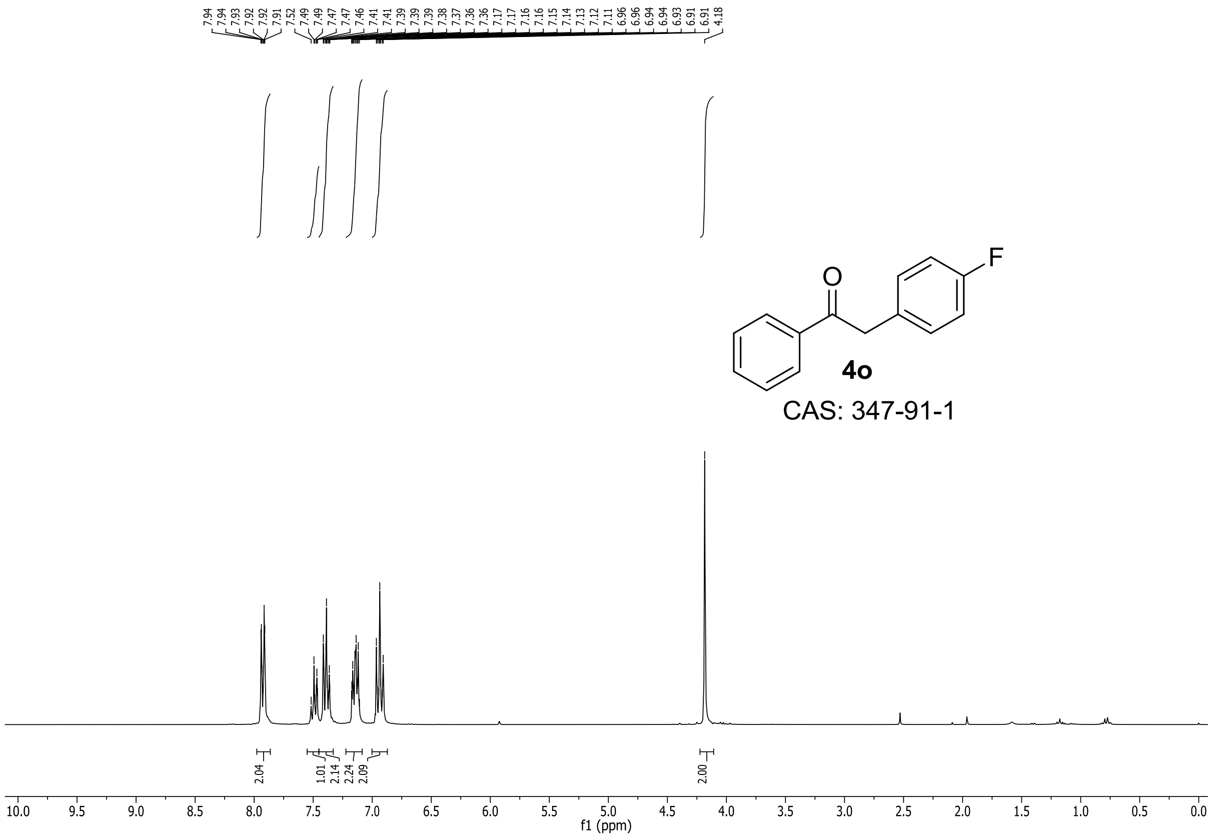

^{1}H NMR data of compound **4p** (300 MHz, $CDCl_3$)

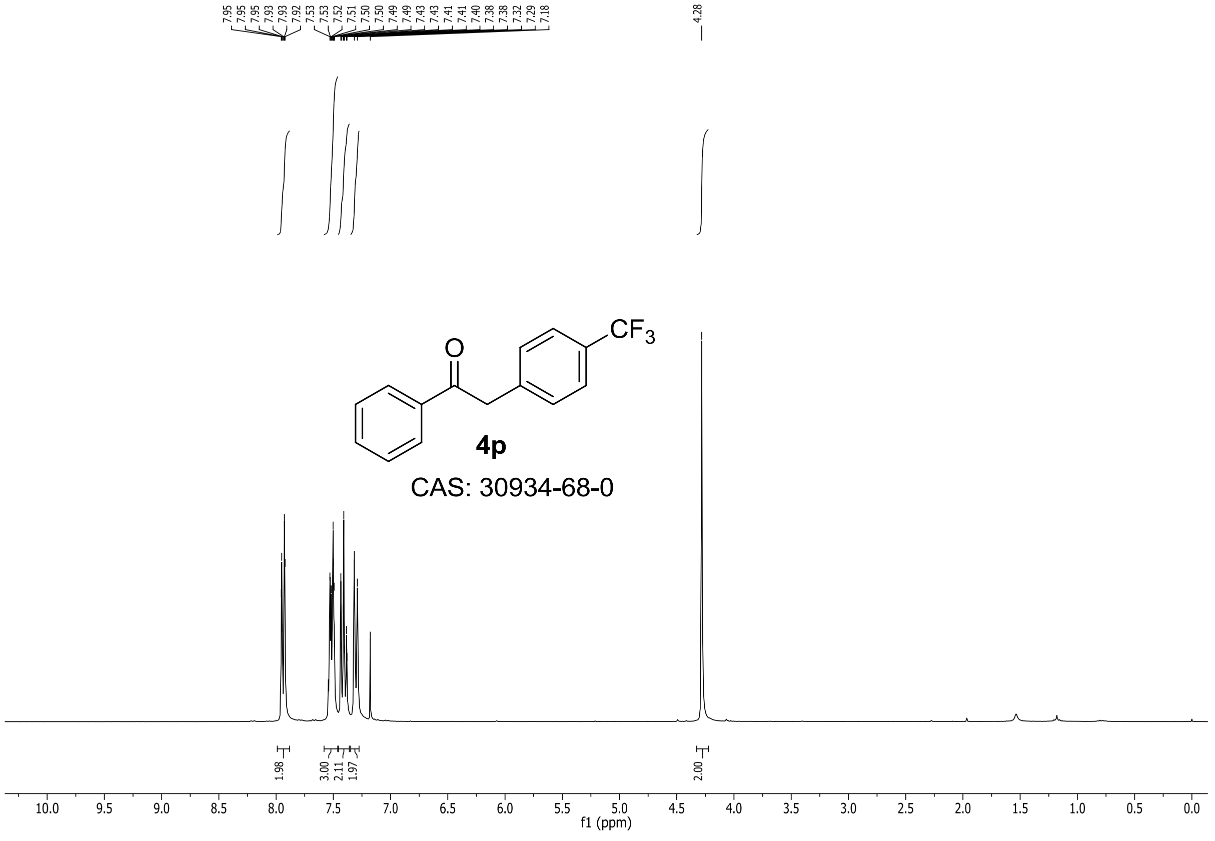

^{1}H NMR data of compound **4q** (300 MHz, $CDCl_3$)

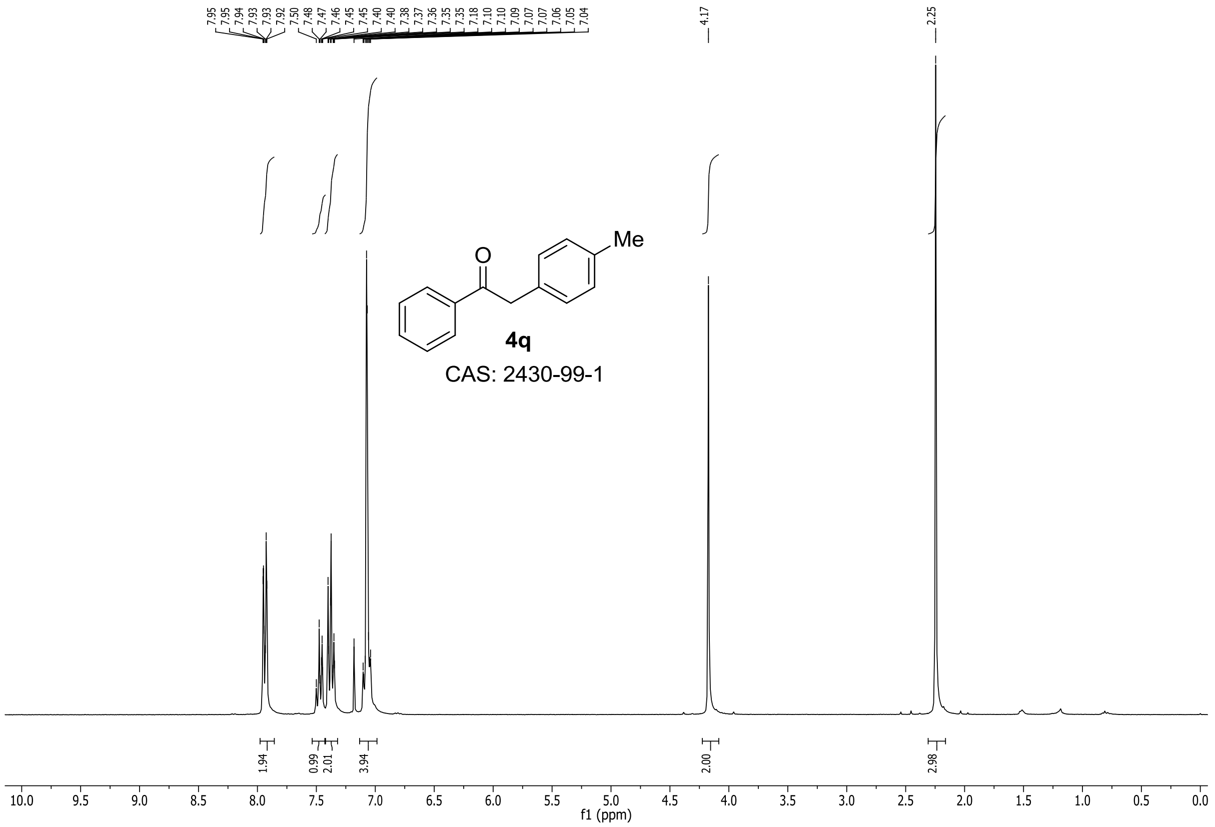

^{1}HNMR data of compound **4r** (300, 75, 376 MHz, $CDCl_3$)

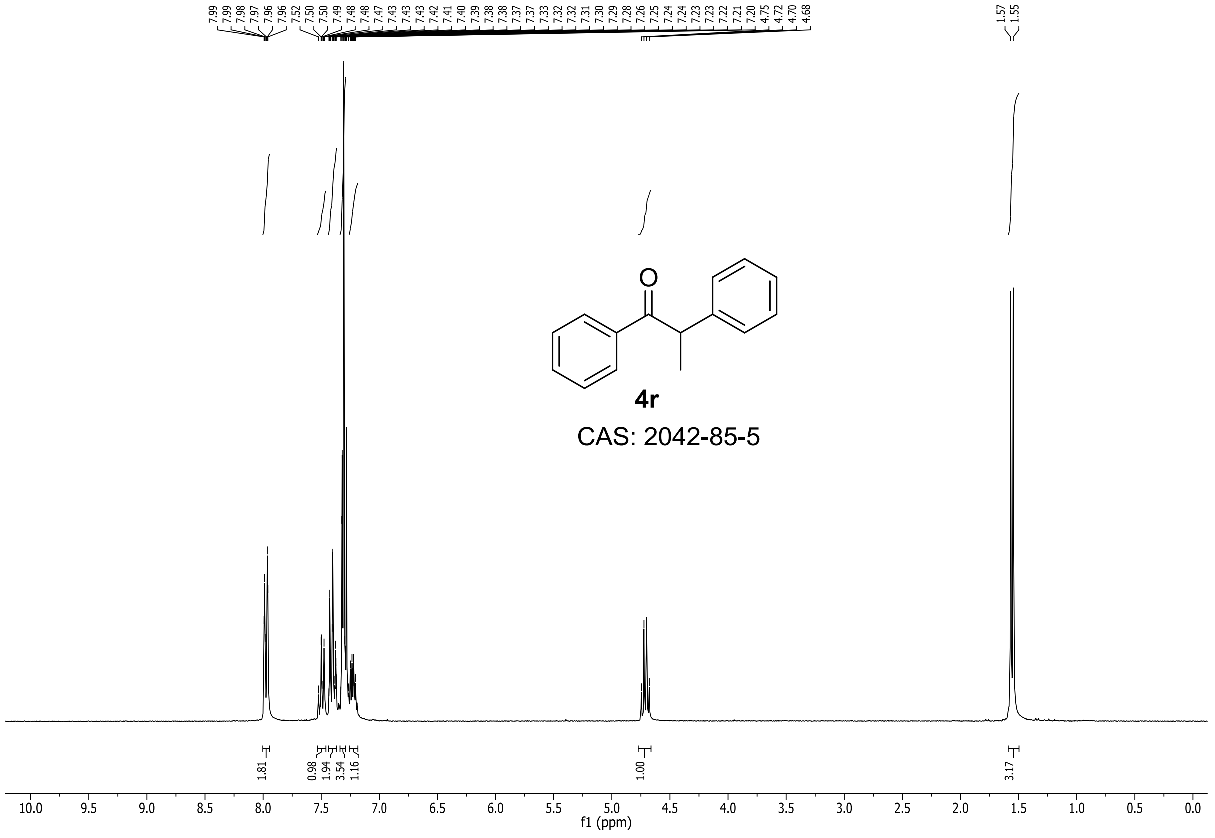

^{1}H,^{13}C and ^{19}F NMR data of compound **4s** (300, 75, 376 MHz, $CDCl_3$)

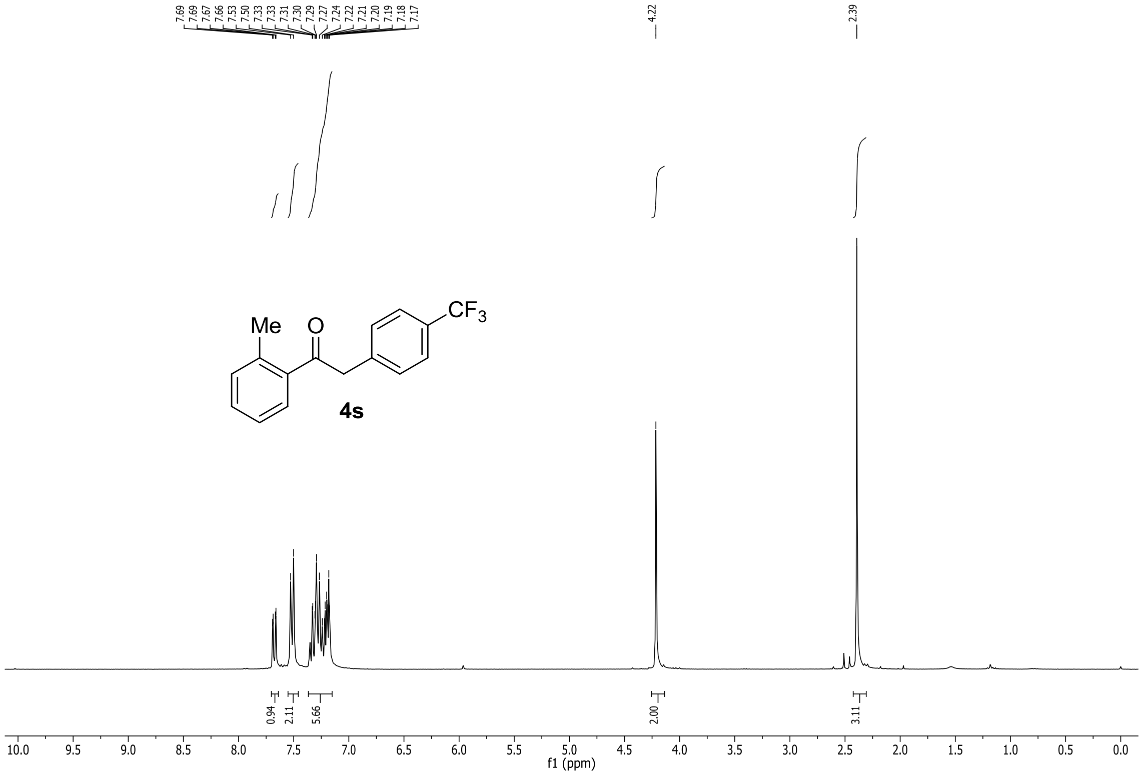

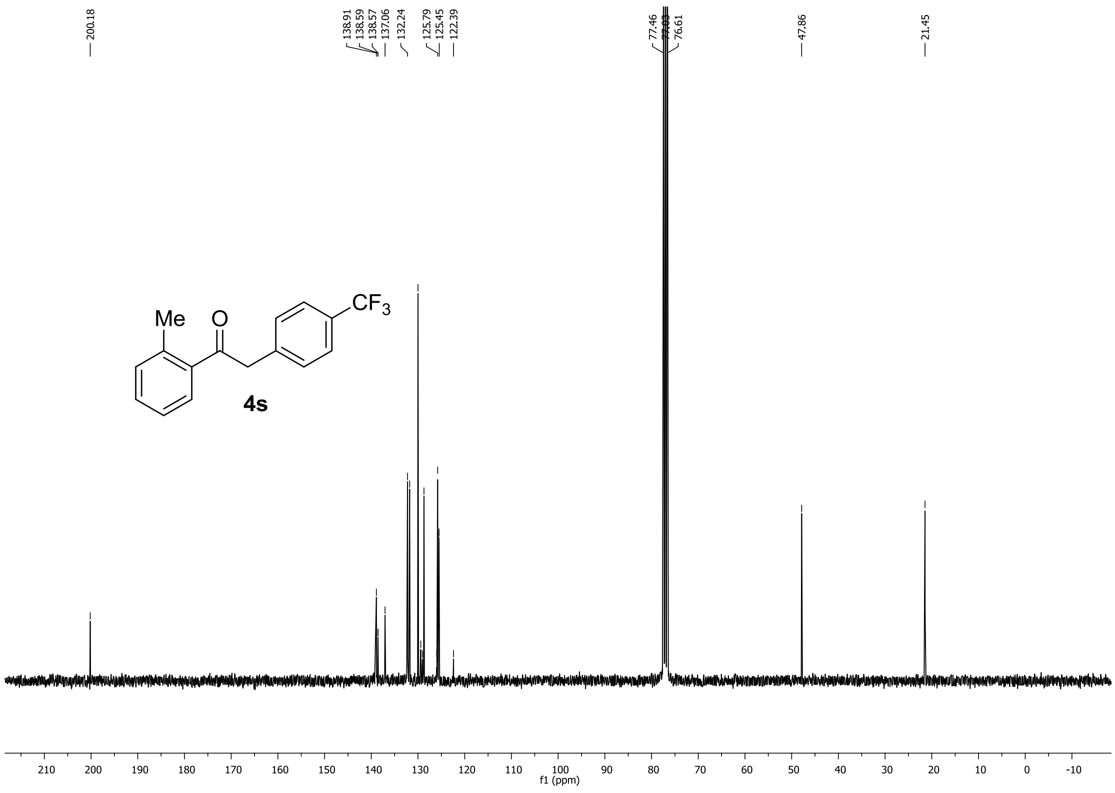

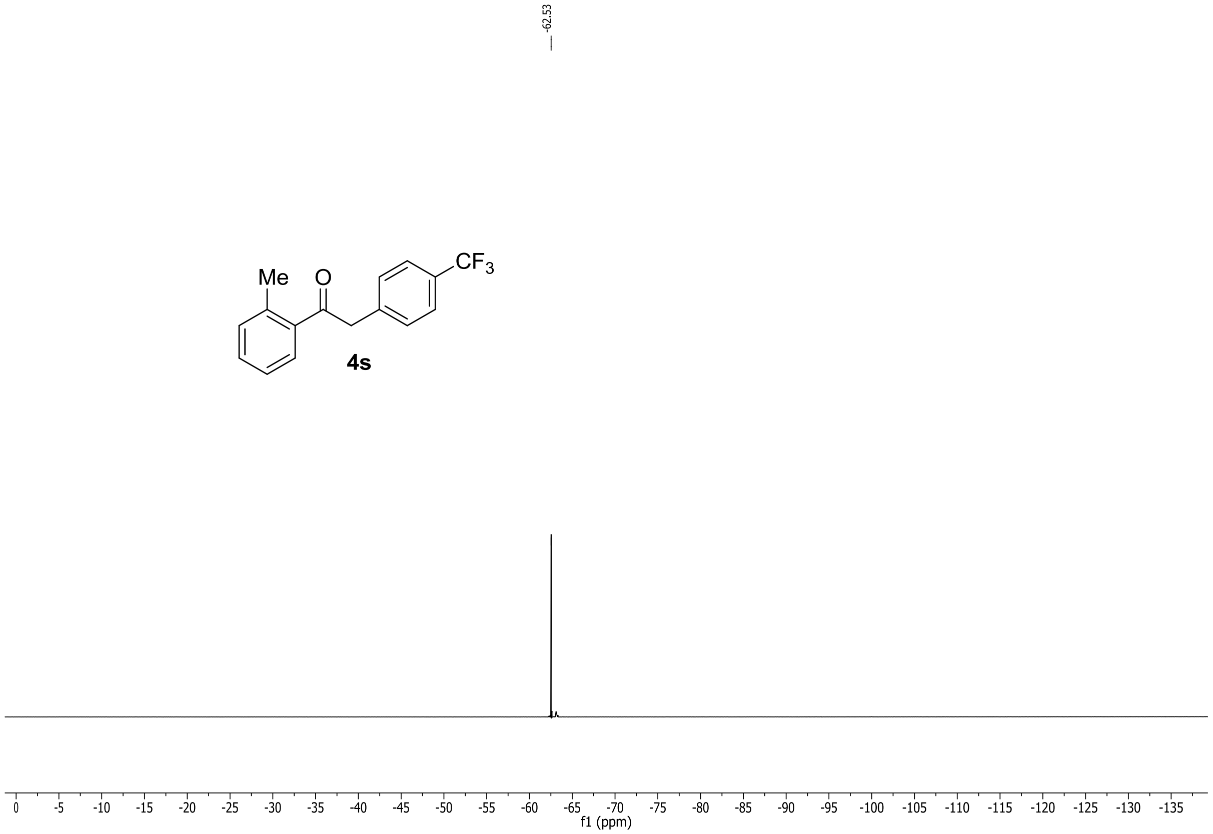

^{1}H,^{13}C and ^{19}F NMR data of compound **4t** (300, 75, 376 MHz, $CDCl_3$)

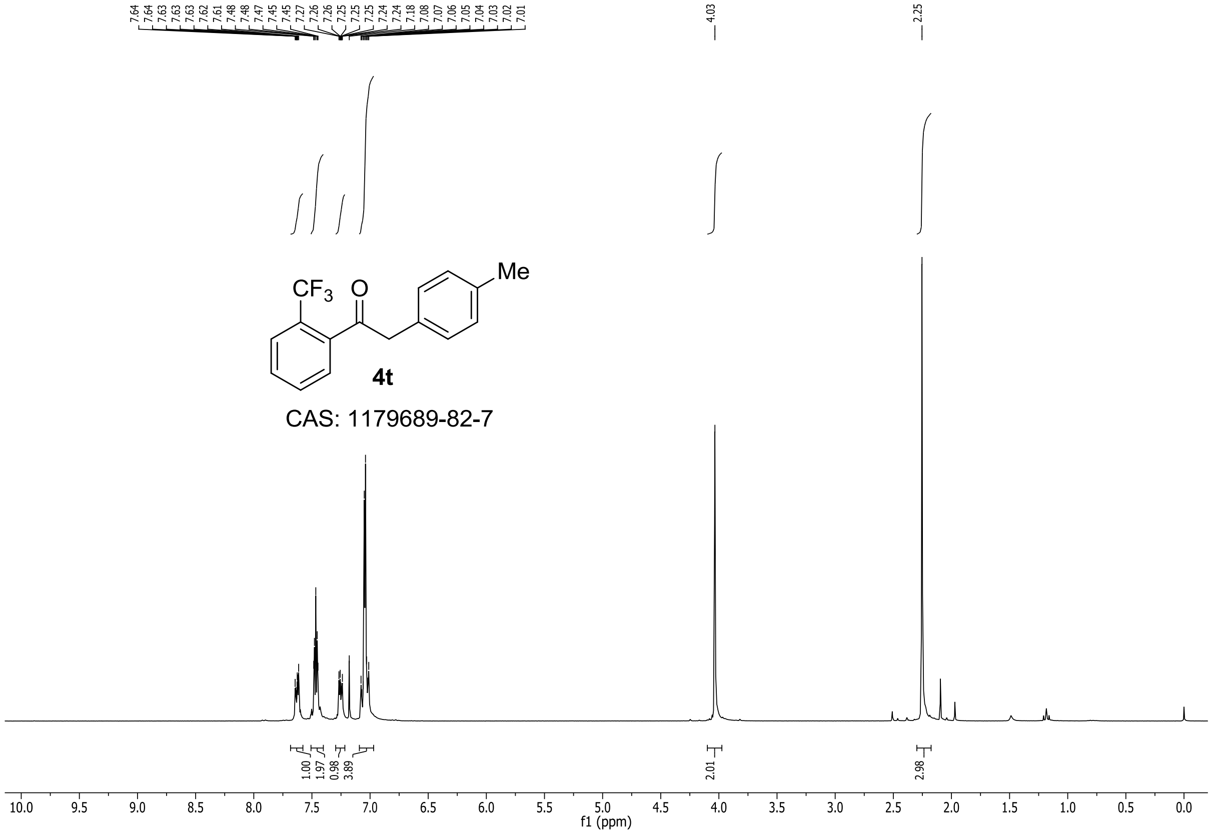

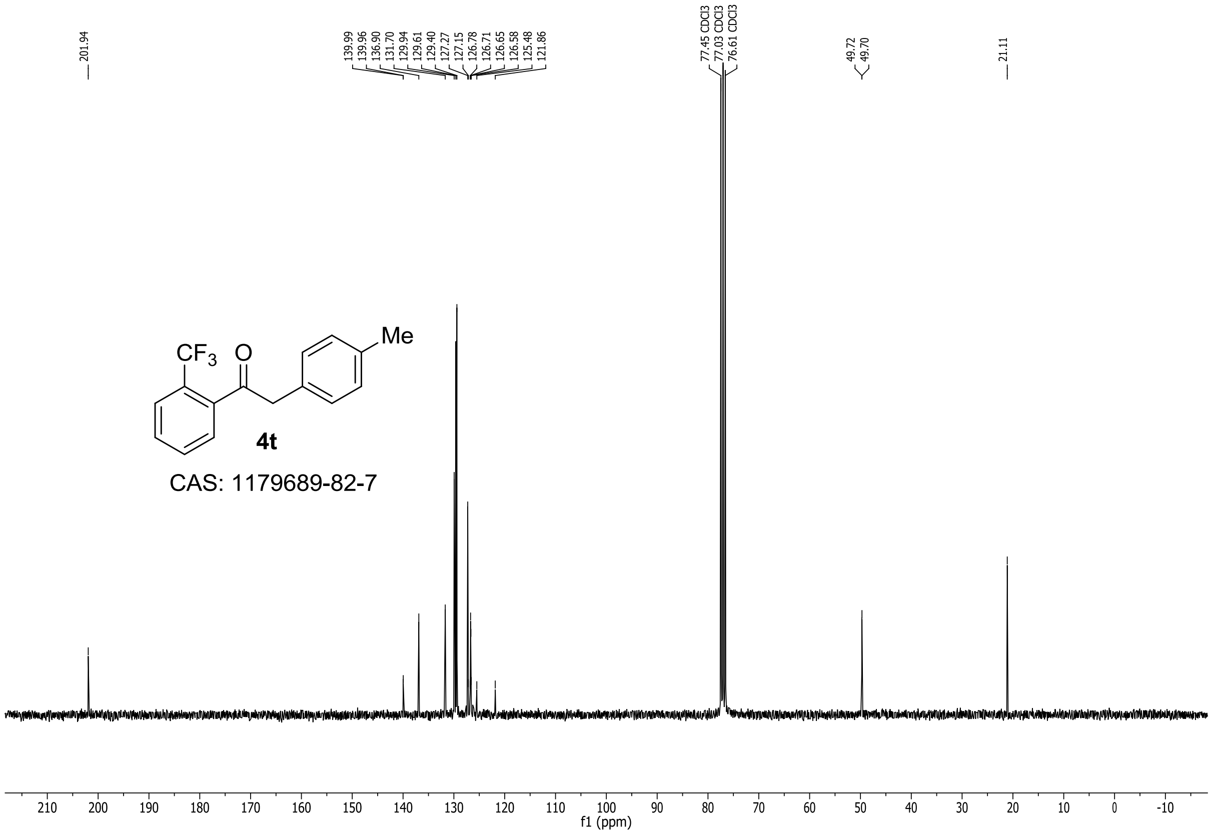
201.94
139.99
139.96
136.90
131.70
129.94
129.61
129.40
127.27
127.15
126.78
126.71
126.65
126.58
125.48
121.86
77.45 CDCl3
77.03 CDCl3
76.61 CDCl3
49.72
49.70
21.11
CF3
O
Me
4t
CAS: 1179689-82-7
f1 (ppm)

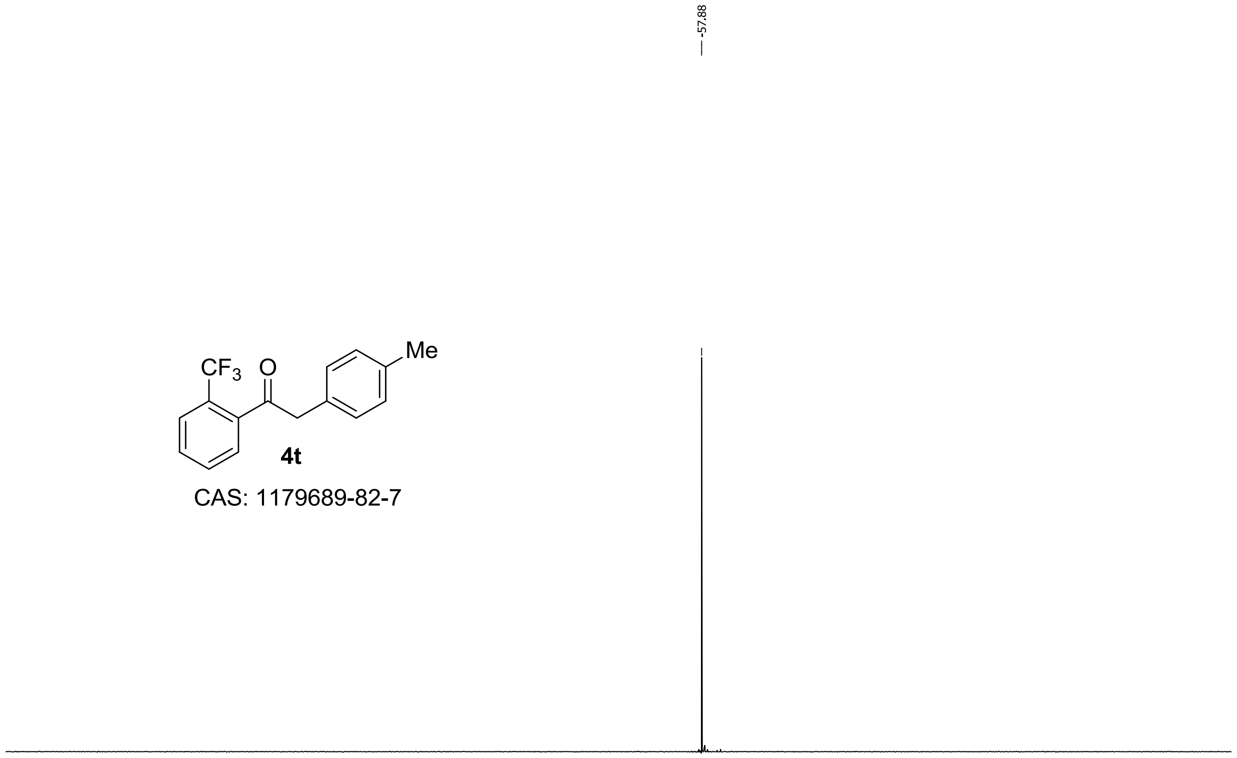
-57.88
CF3
O
Me
4t
CAS: 1179689-82-7
f1 (ppm)

^{1}HNMR data of compound **11a** (300 MHz, $CDCl_3$)

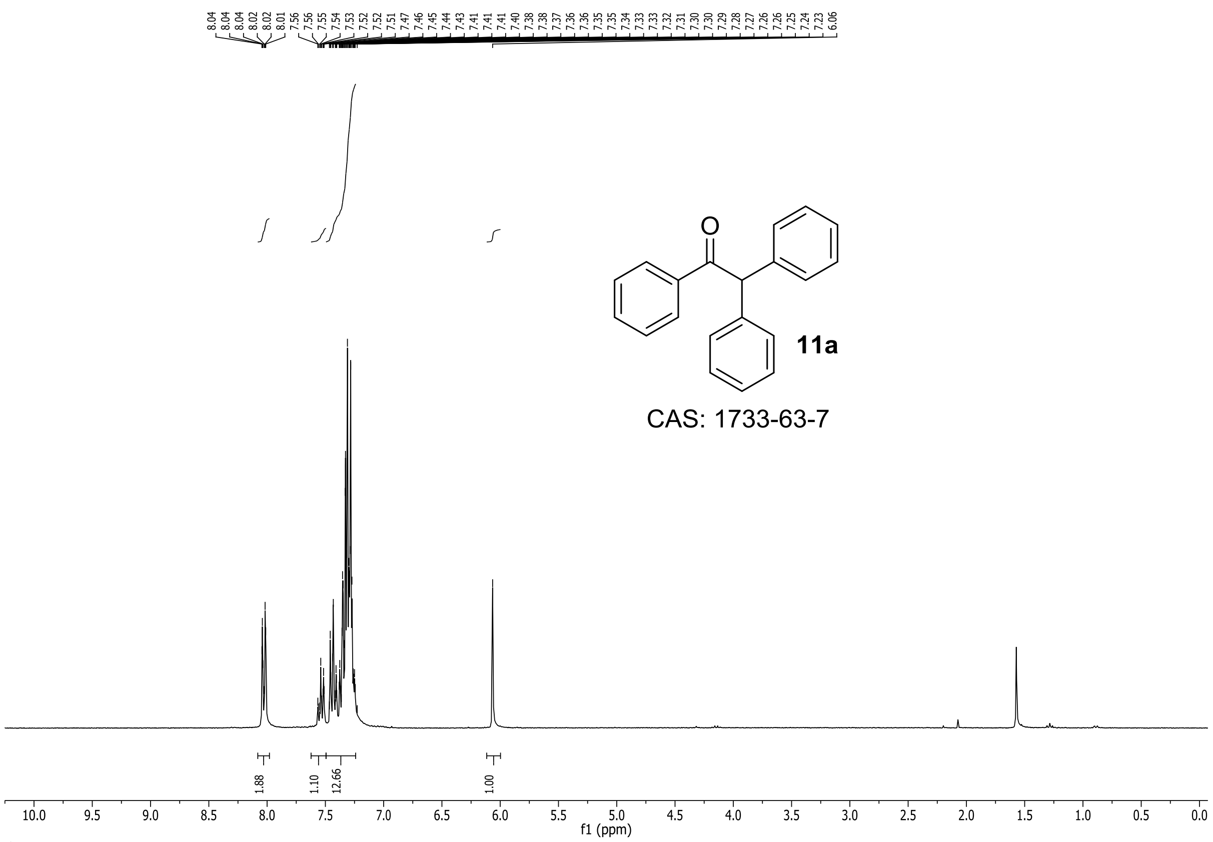

^{1}HNMR data of compound **11b** (300 MHz, $CDCl_3$)

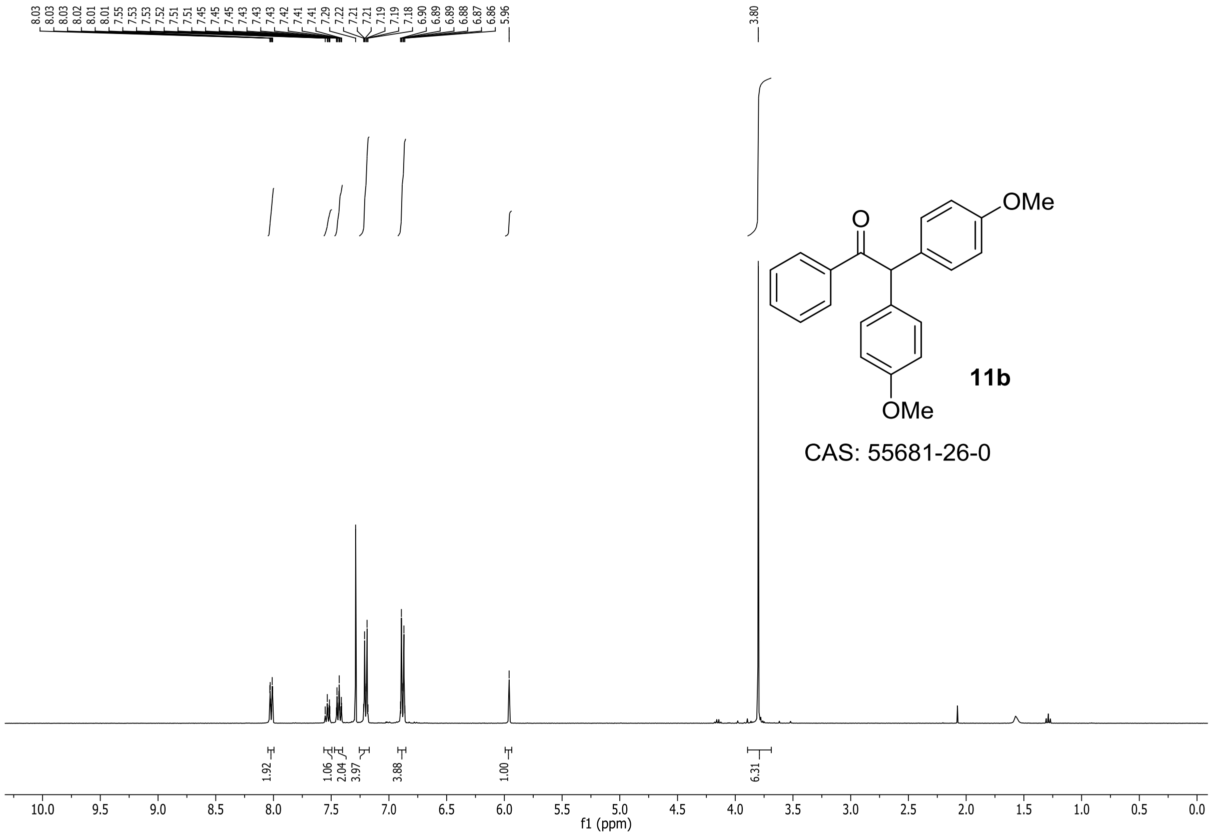

^{1}HNMR data of compound **11c** (300 MHz, $CDCl_3$)

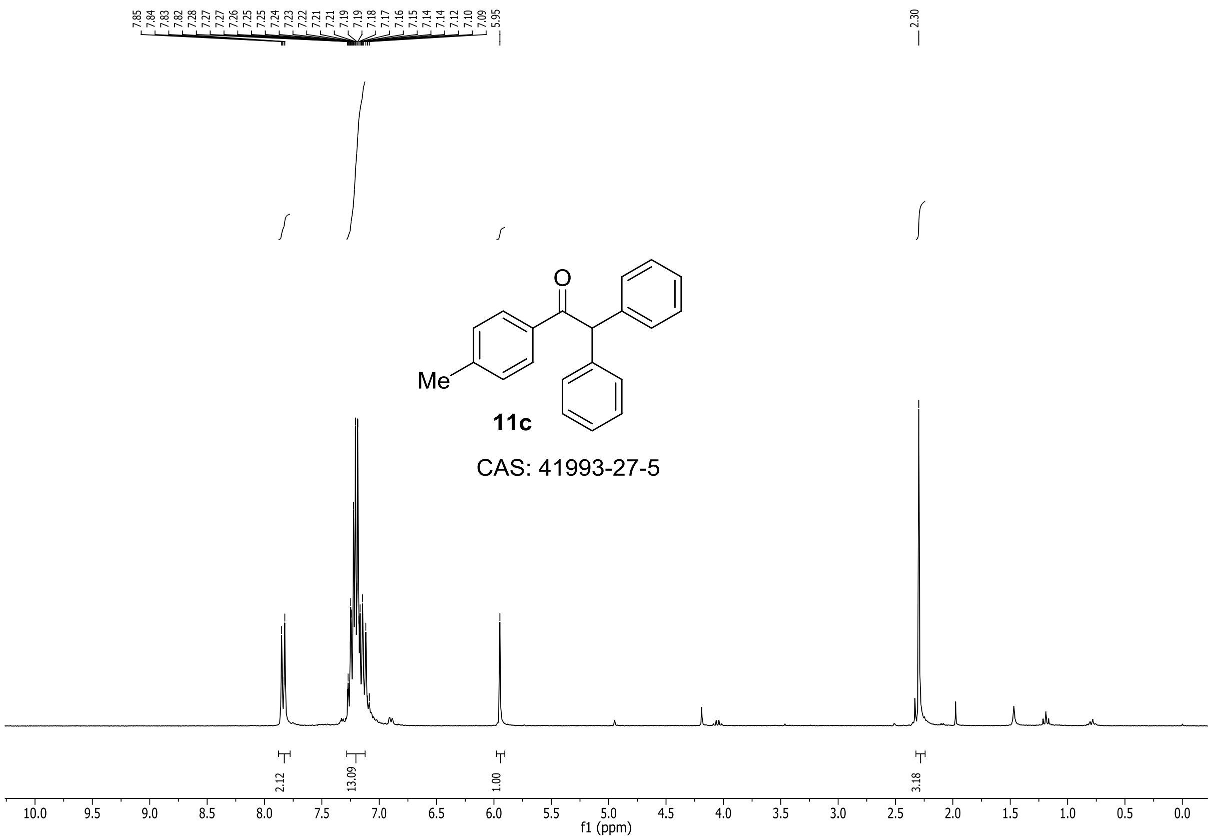

^{1}HNMR data of compound **11d** (300 MHz, $CDCl_3$)

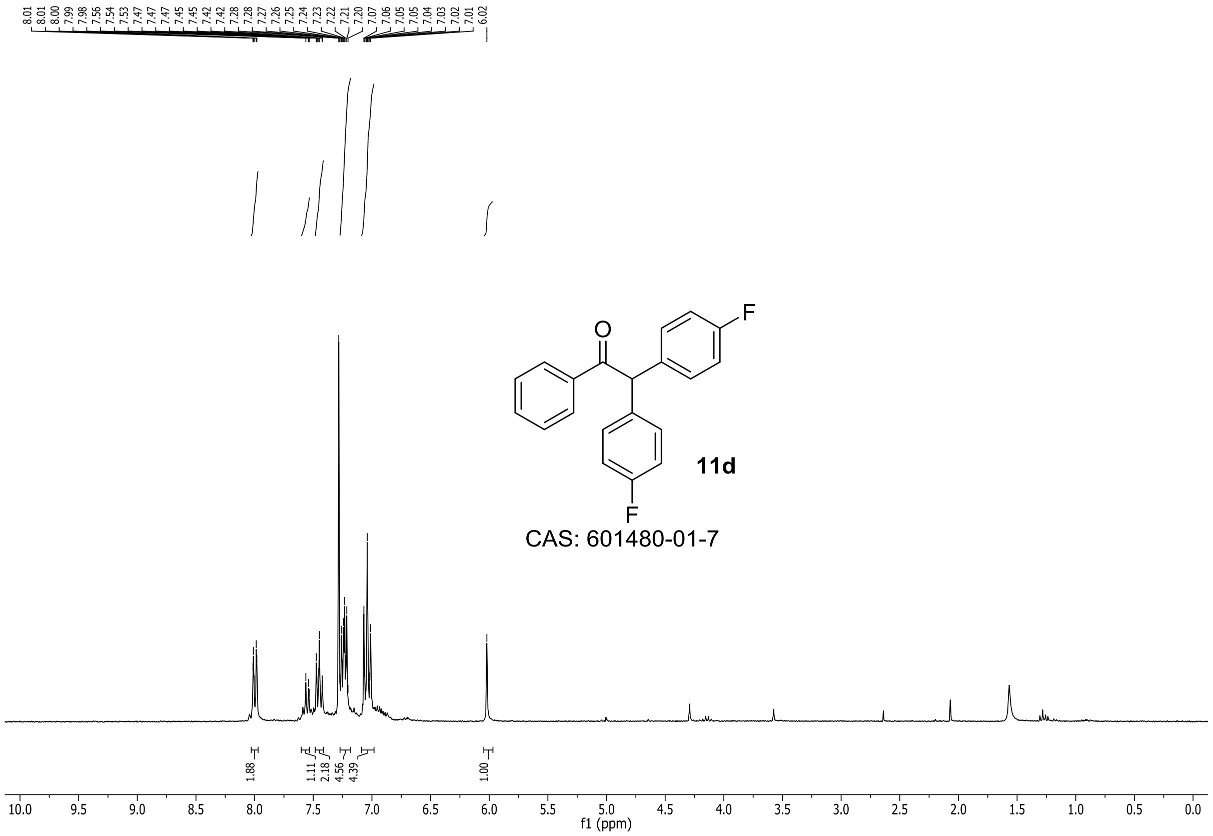

^{1}H and ^{13}C NMR data of compound **6a** (300, 75 MHz, $CDCl_3$)

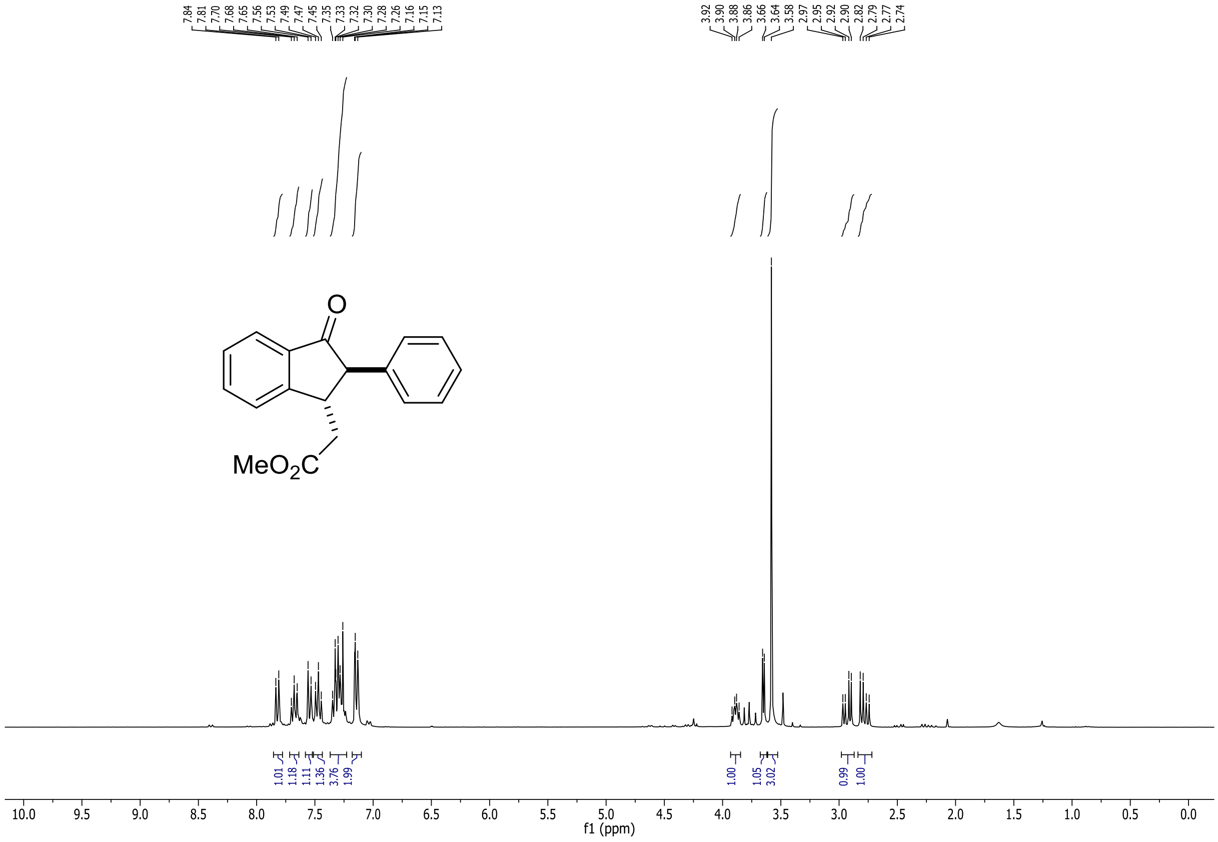

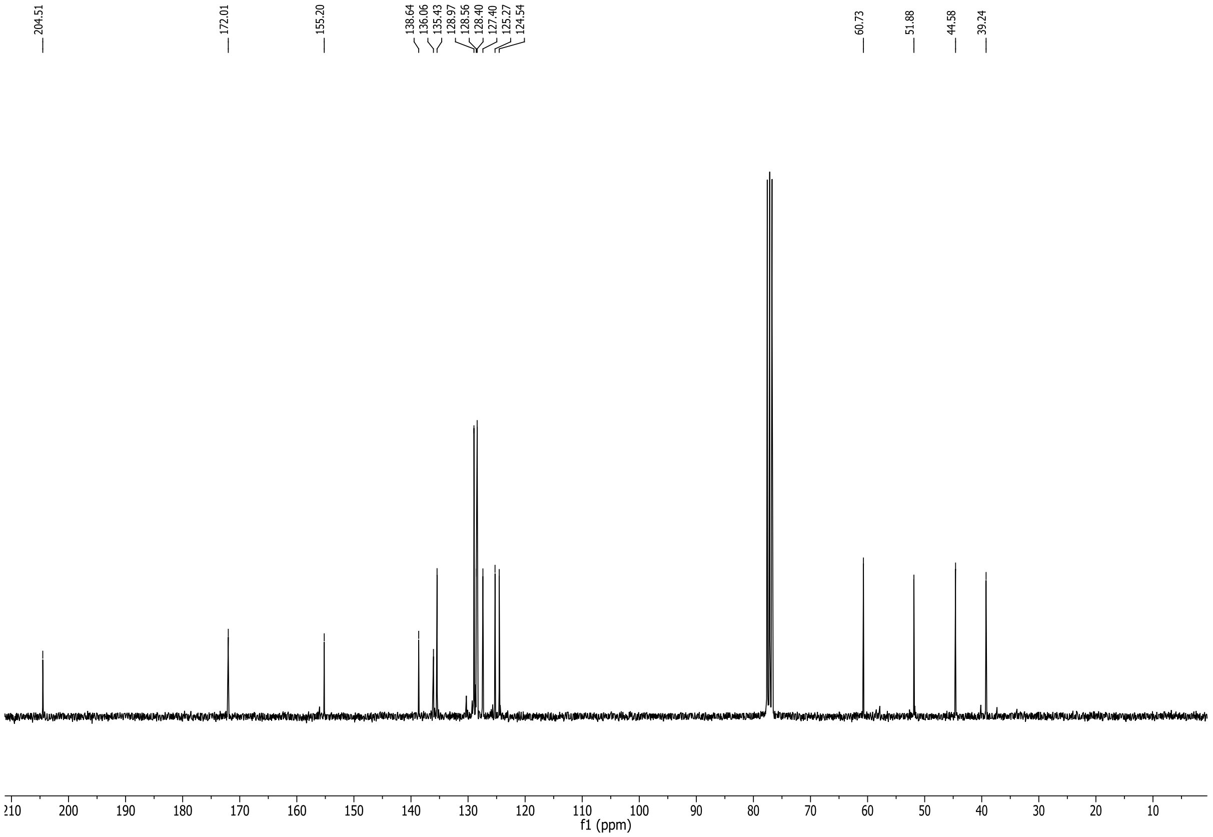

^{1}H and ^{13}C NMR data of compound **7a** (300, 75 MHz, $CDCl_3$)

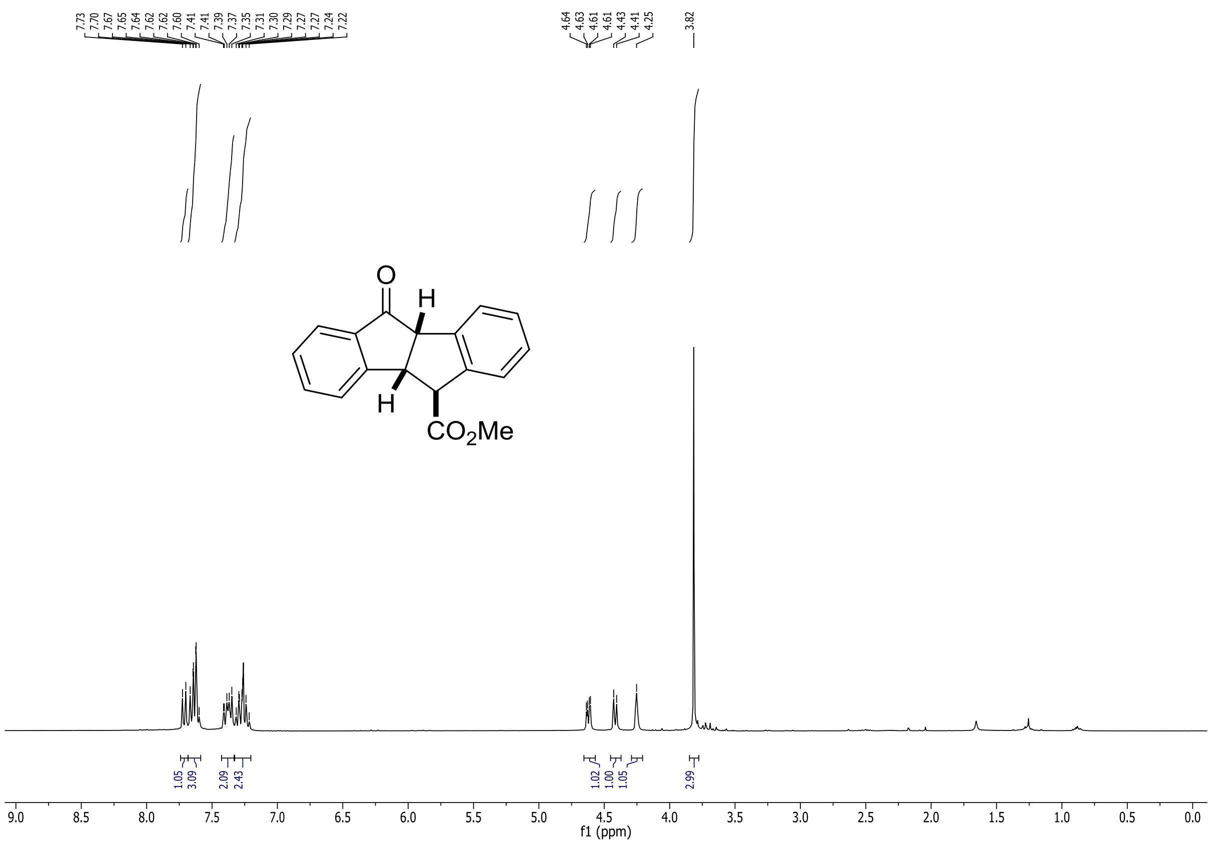

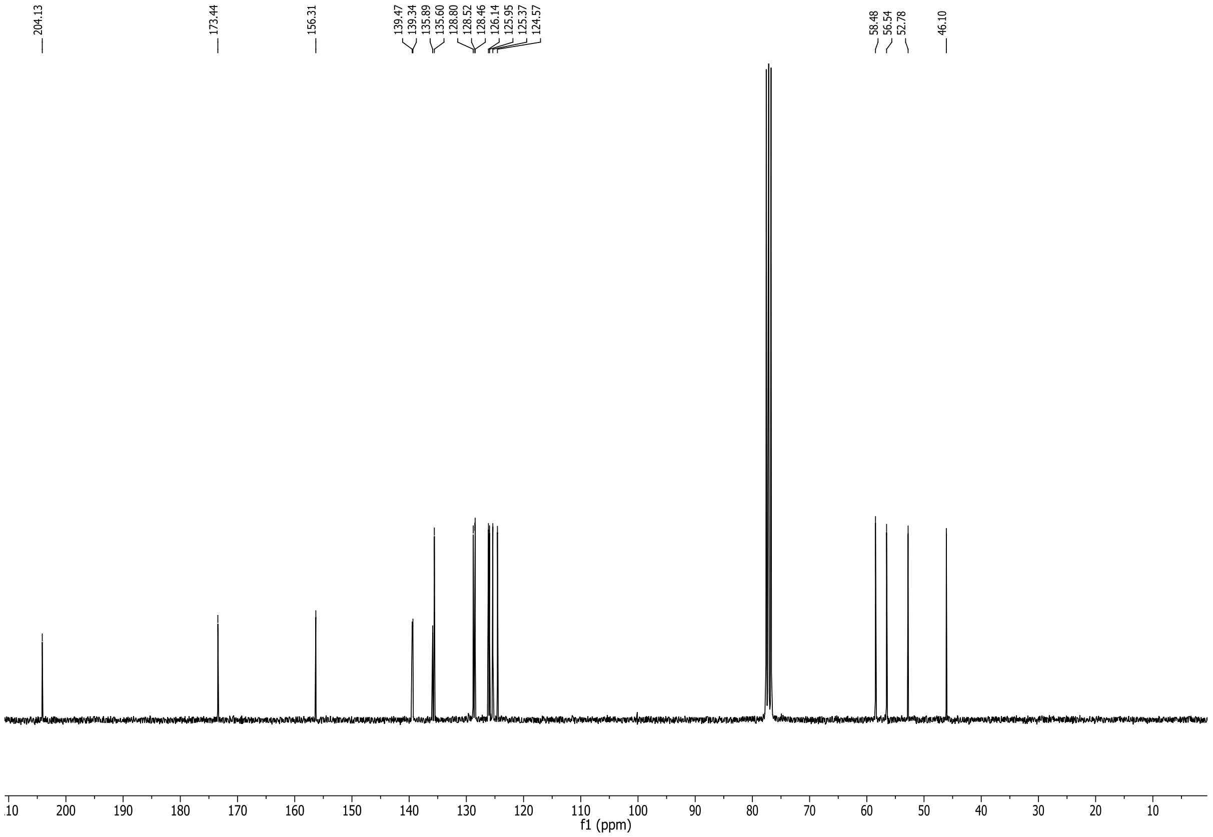

^{1}H and ^{13}C NMR data of compound **6b** (400, 101 MHz, $CDCl_3$)

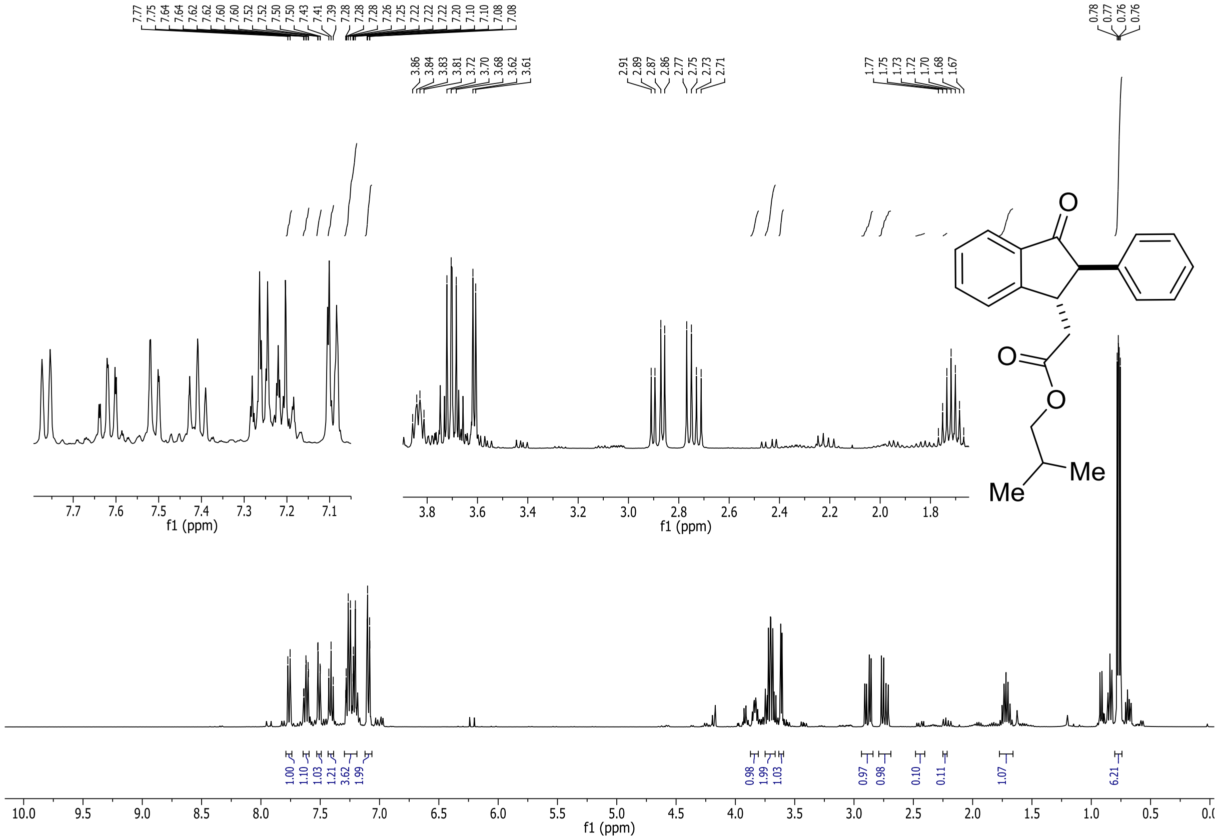

7.77 7.75 7.64 7.62 7.62 7.60 7.60 7.52 7.52 7.50 7.50 7.43 7.41 7.39 7.28 7.28 7.28 7.26 7.25 7.22 7.22 7.22 7.20 7.10 7.10 7.08 7.08
3.86 3.84 3.83 3.81 3.72 3.70 3.68 3.62 3.61
2.91 2.89 2.87 2.86 2.77 2.75 2.73 2.71
1.77 1.75 1.73 1.72 1.70 1.68 1.67
0.78 0.77 0.76 0.76
O
O
O
Me
Me
f1 (ppm)
f1 (ppm)
1.00 1.10 1.03 1.21 3.62 1.99 0.98 1.99 1.03 0.97 0.98 0.10 0.11 1.07 6.21
f1 (ppm)

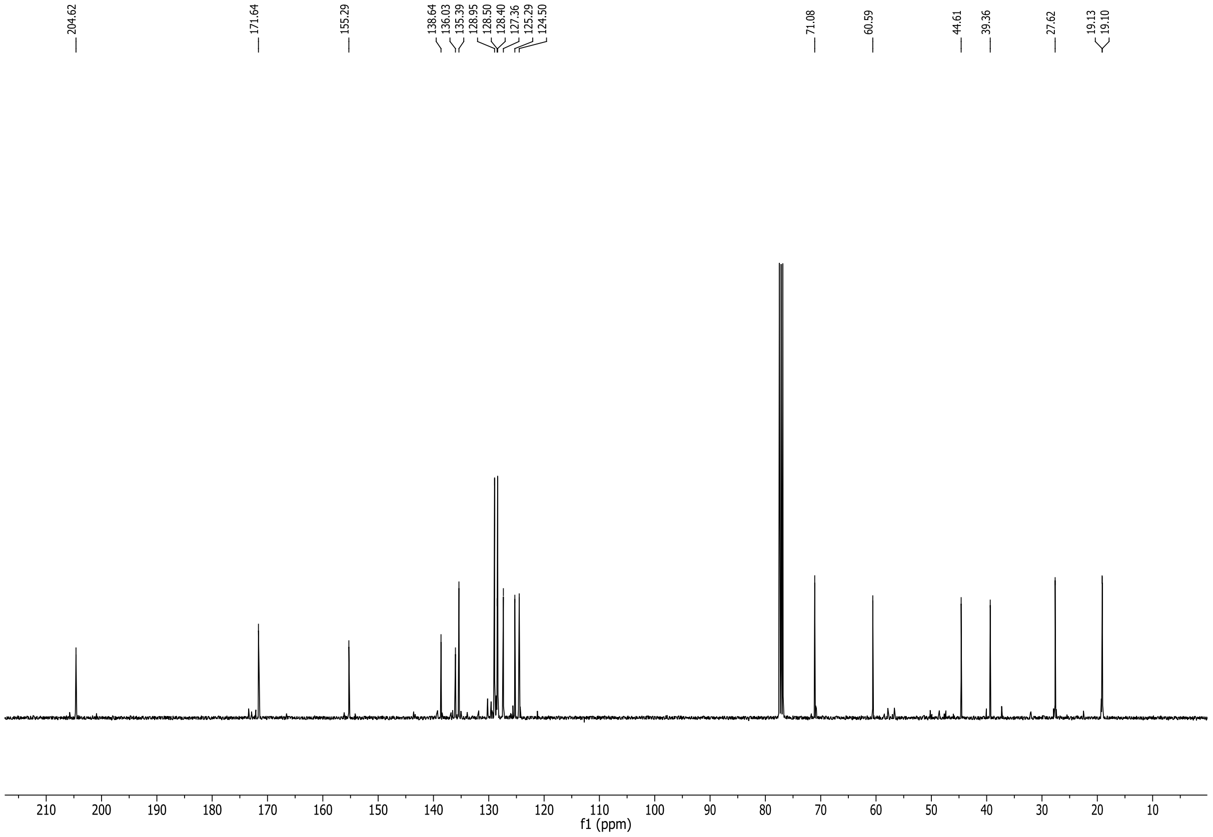

204.62 171.64 155.29 138.64 136.03 135.39 128.95 128.50 128.40 127.36 125.29 124.50 71.08 60.59 44.61 39.36 27.62 19.13 19.10
f1 (ppm)

^{1}H and ^{13}C NMR data of compound **6c** (500, 125 MHz, $CDCl_3$)

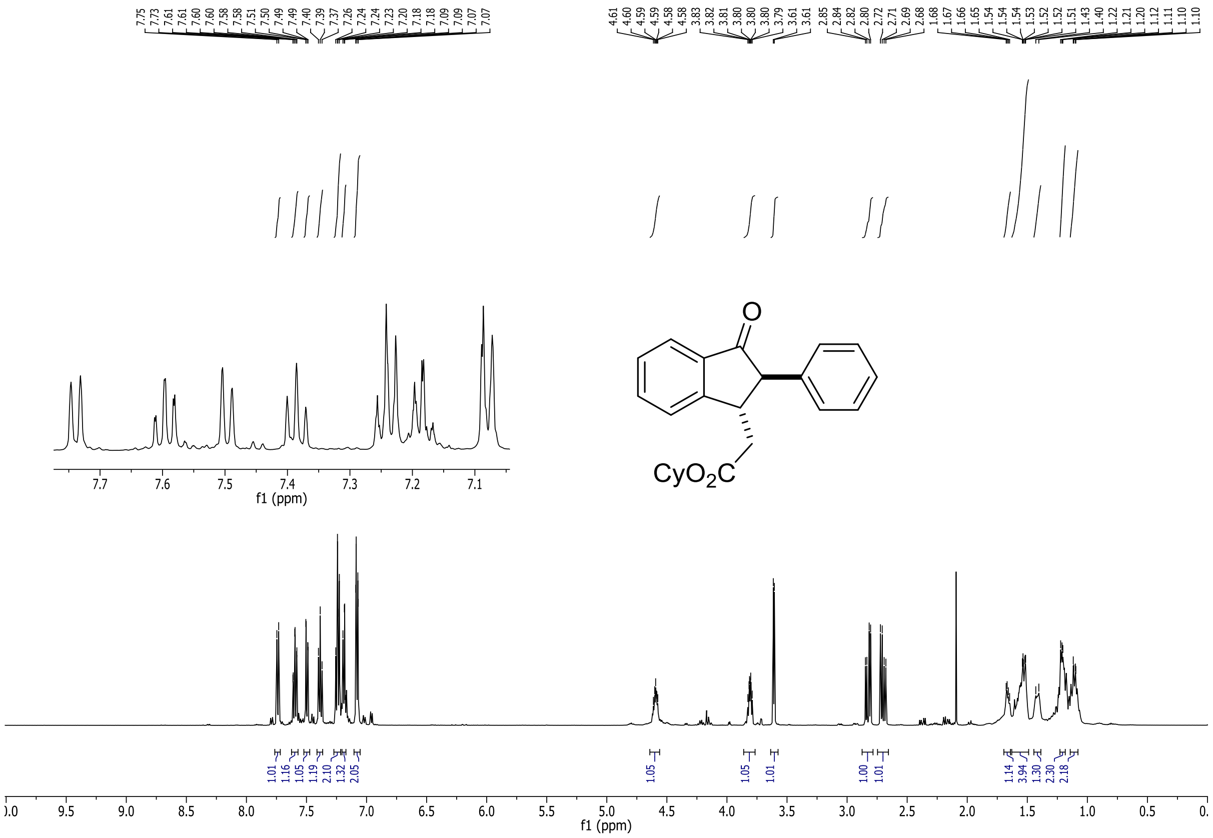

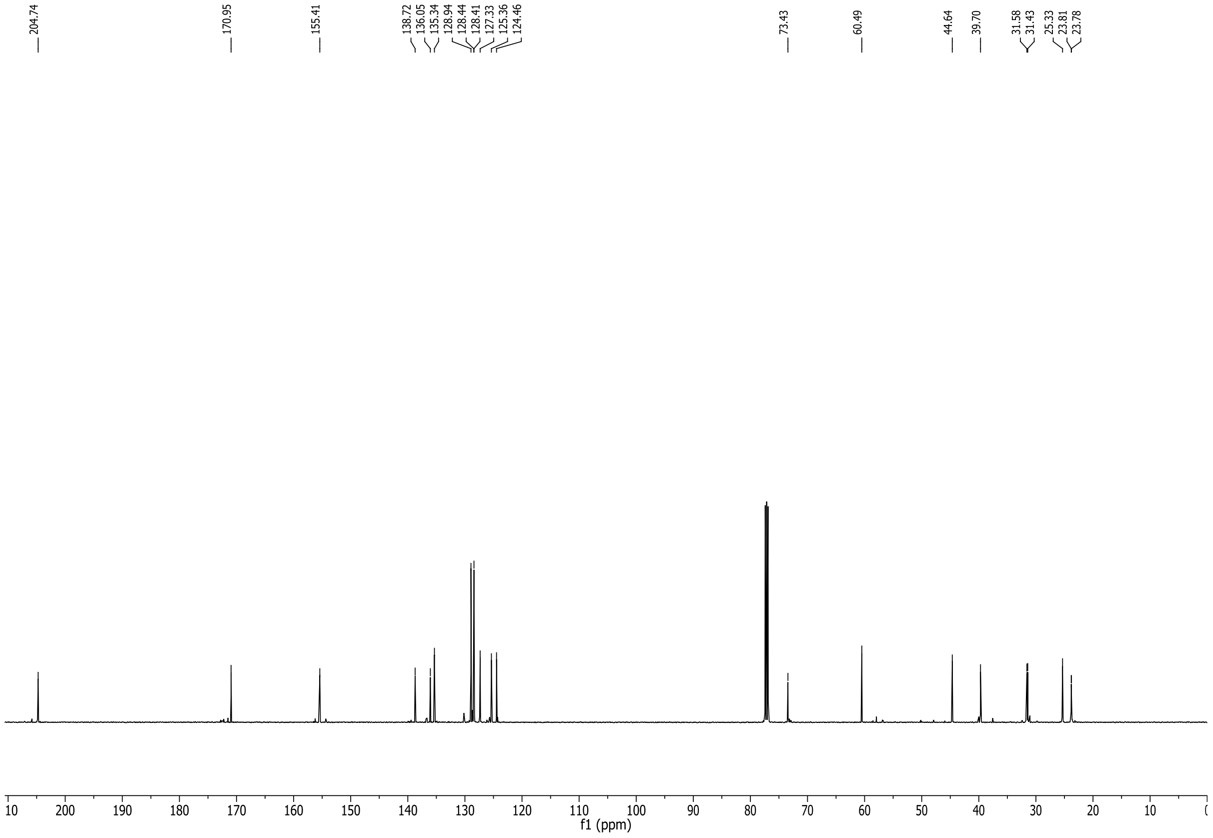

^{1}H and ^{13}C NMR data of compound **6d** (500, 125 MHz, $CDCl_3$)

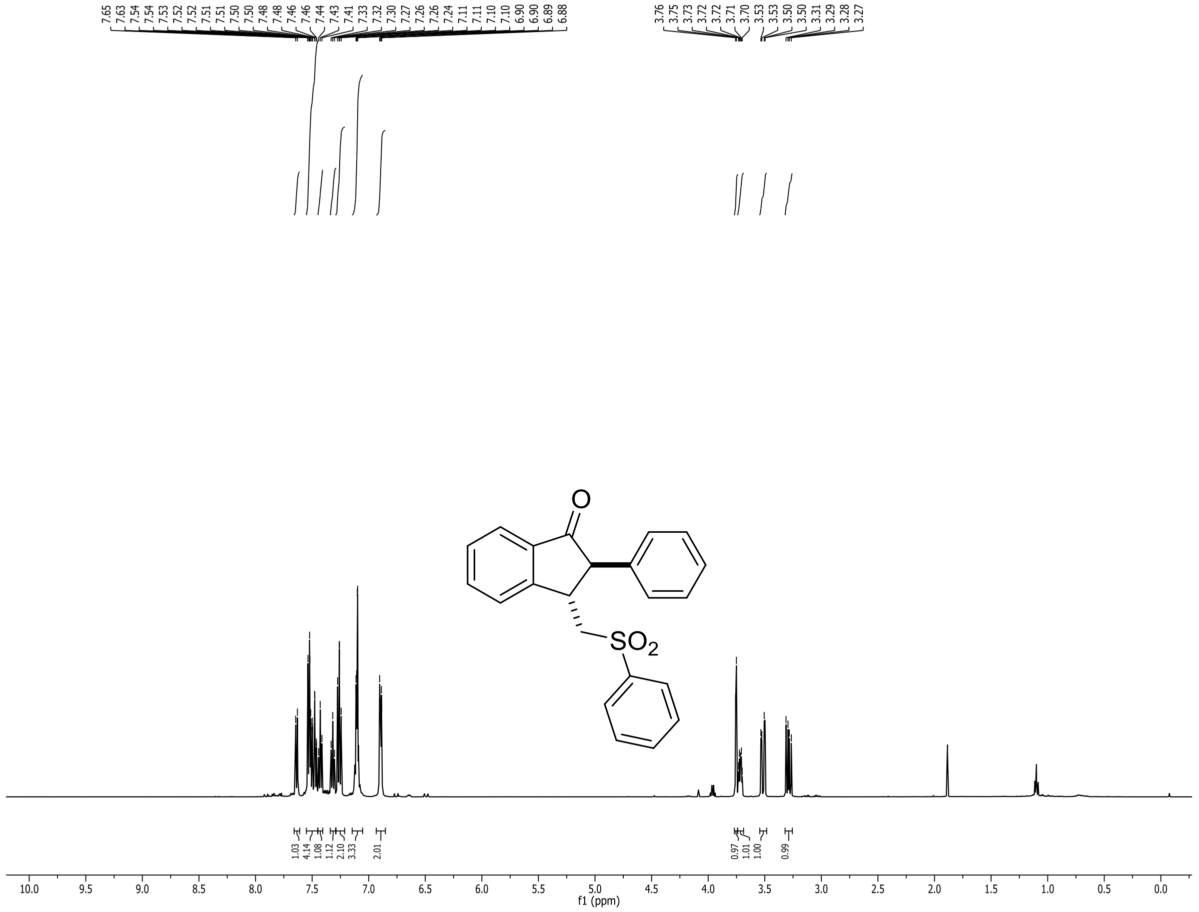

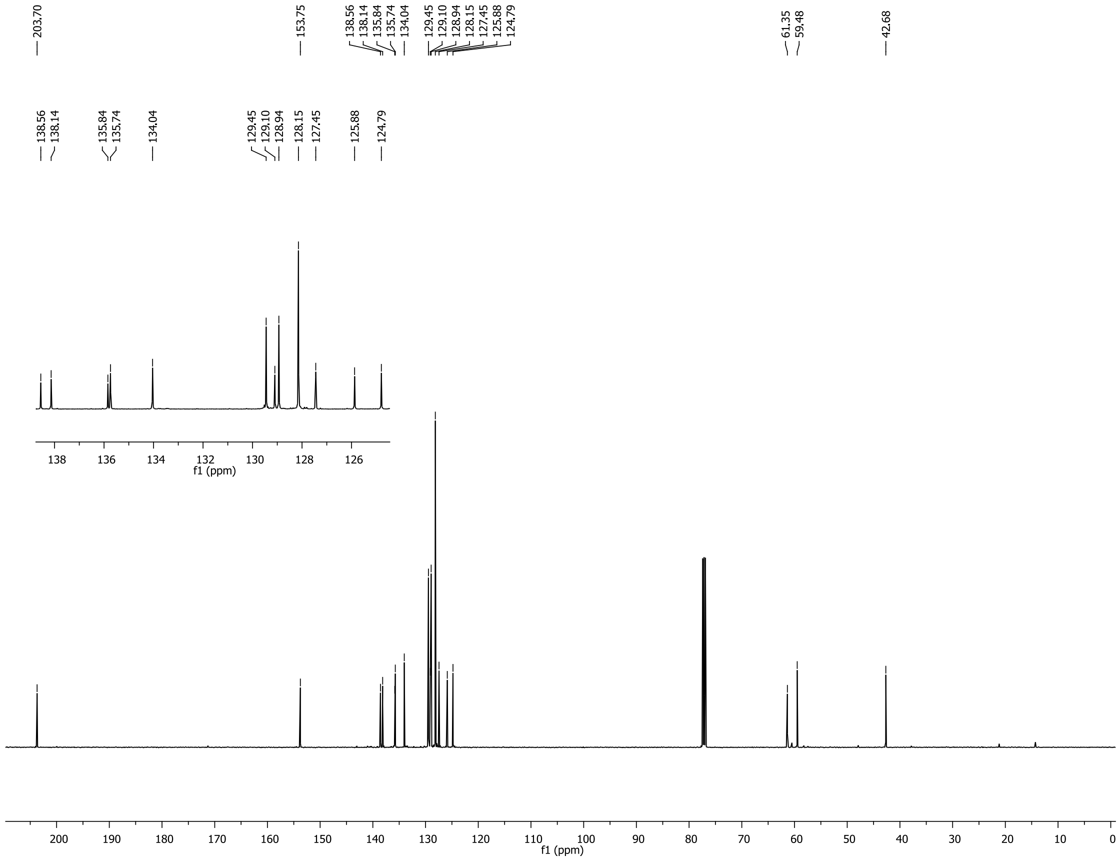

^{1}H, ^{13}C and ^{19}F NMR data of compound **6e** (400, 75, 376 MHz, $CDCl_3$)

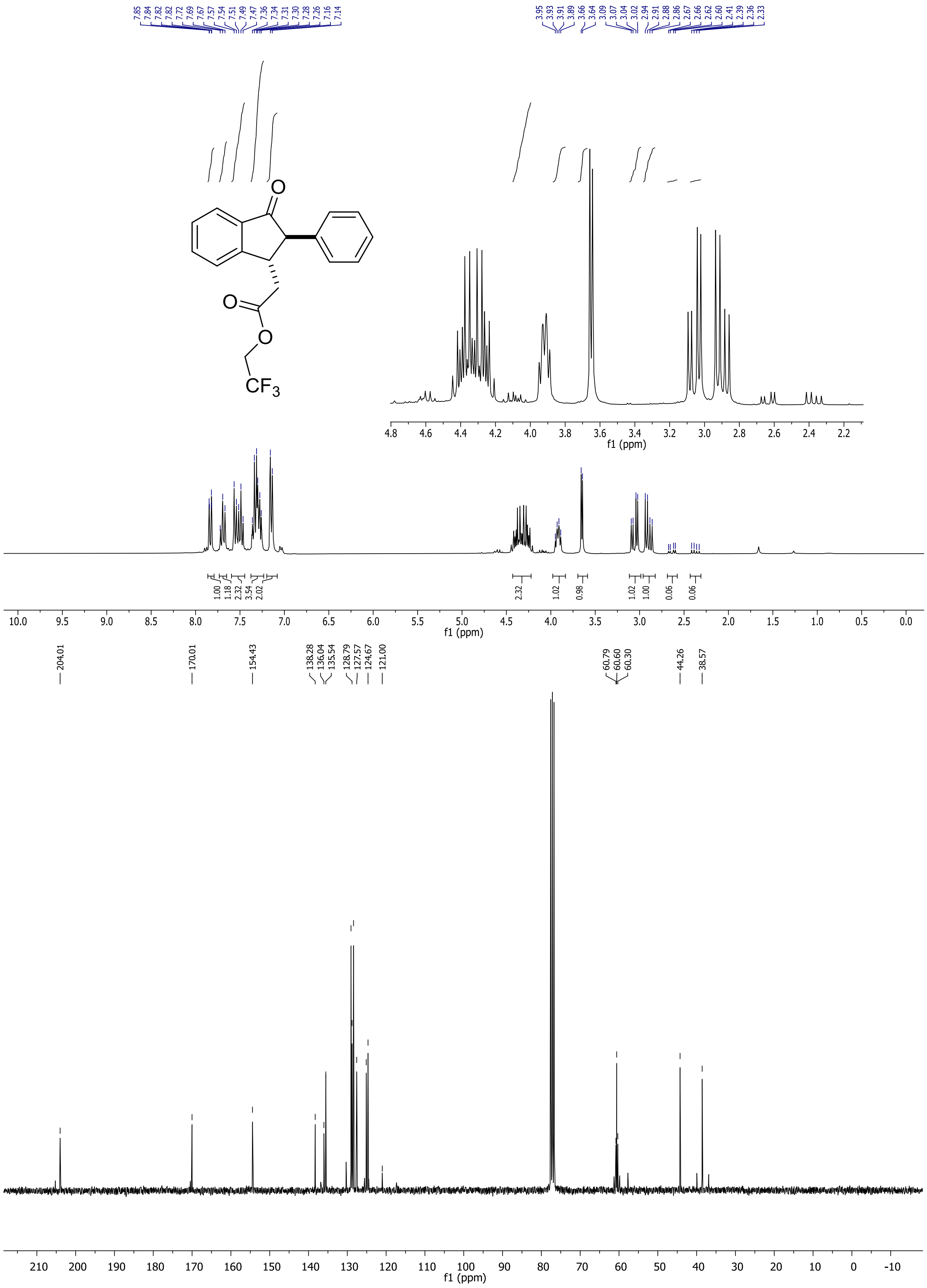

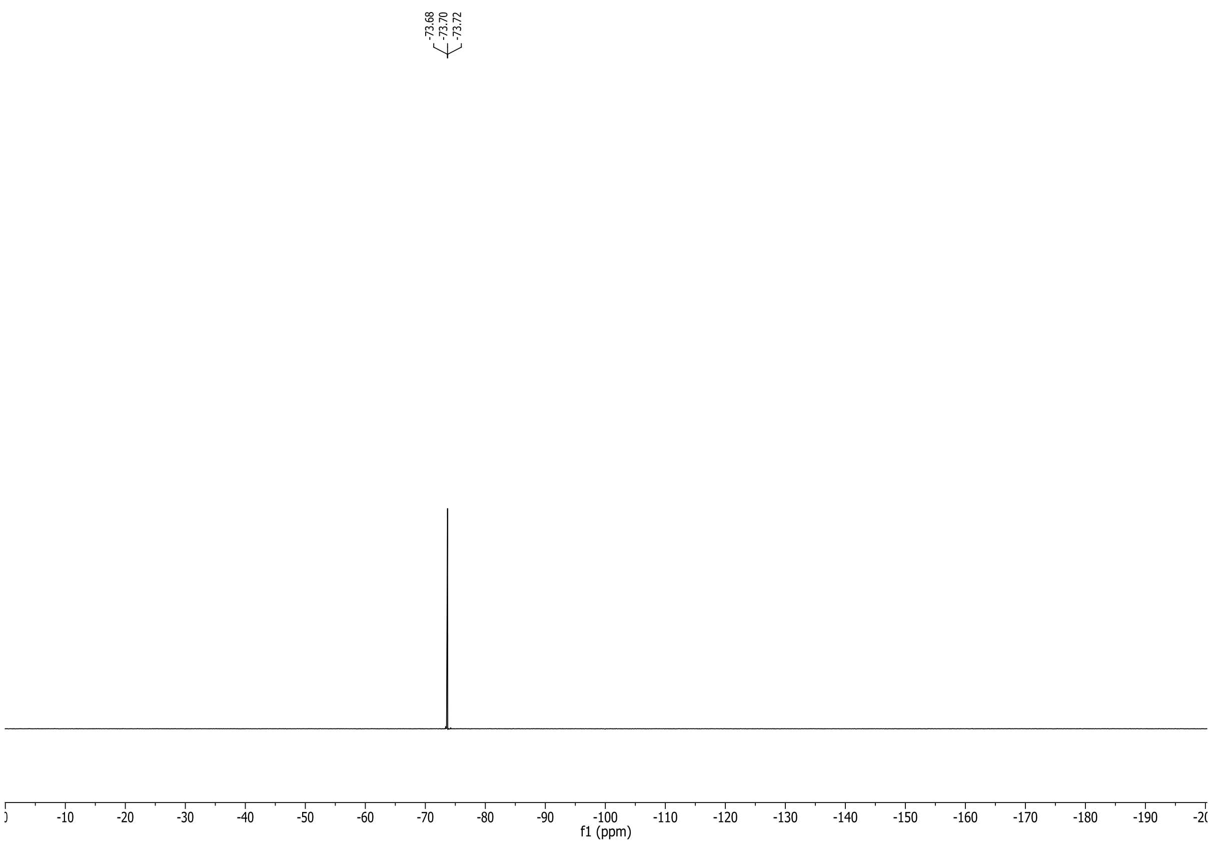

^{1}H, ^{13}C and ^{19}F NMR data of compound **7e** (400, 75, 376 MHz, $CDCl_3$)

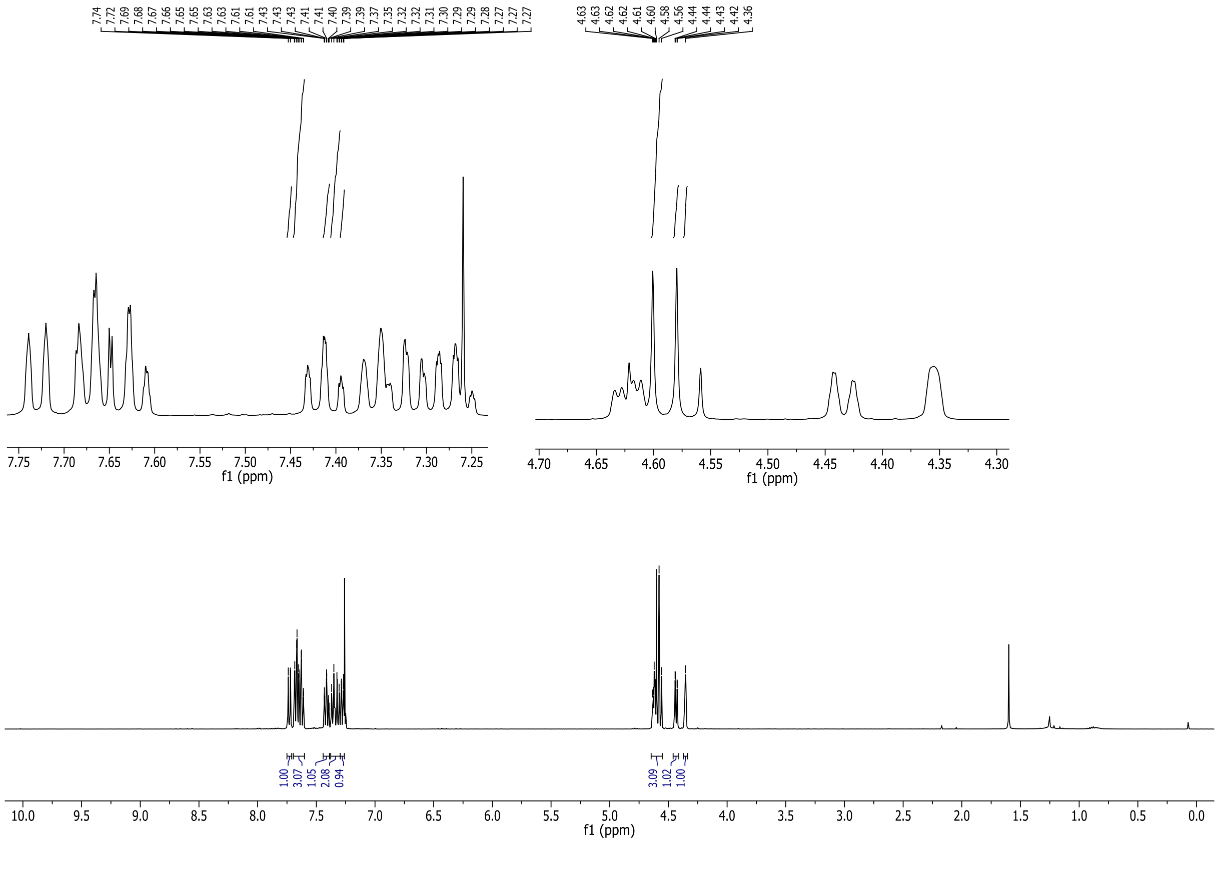

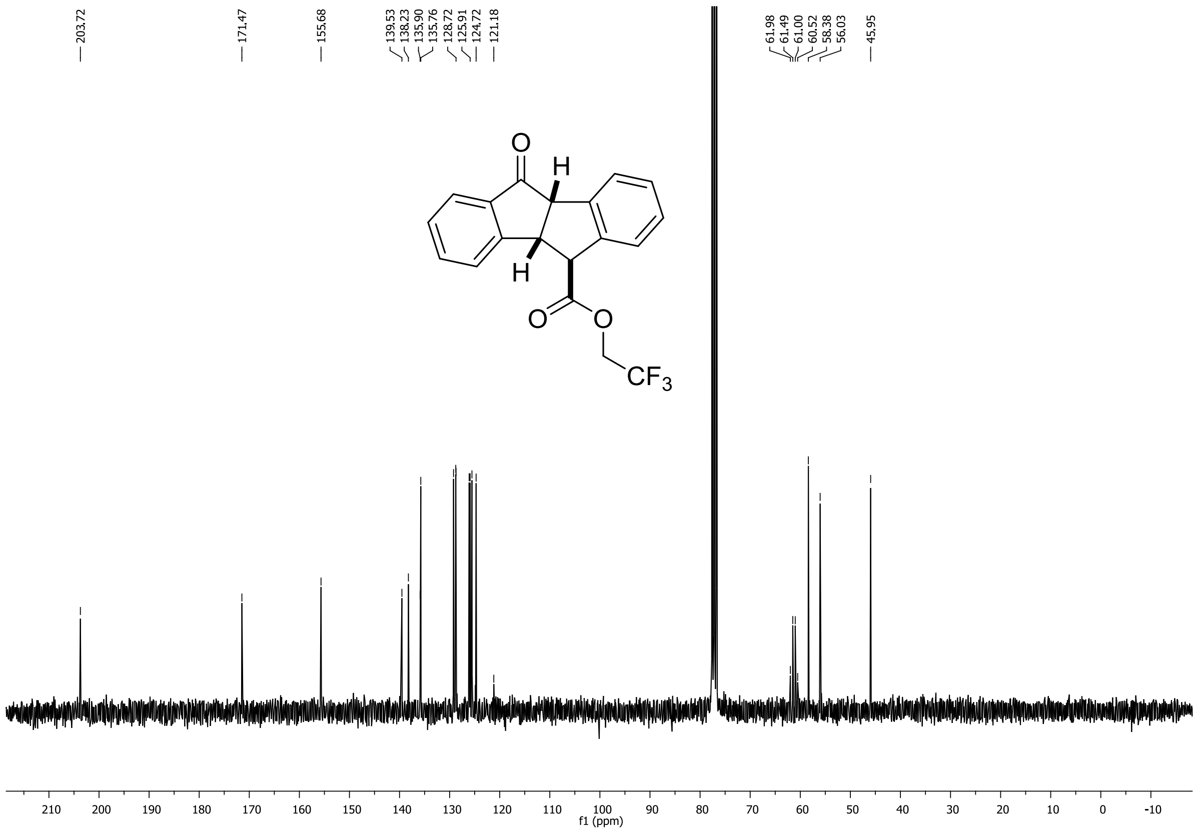
203.72
171.47
155.68
139.53
138.23
135.90
135.76
128.72
125.91
124.72
121.18
61.98
61.49
61.00
60.52
58.38
56.03
45.95
O
H
H
O
O
CF3
f1 (ppm)

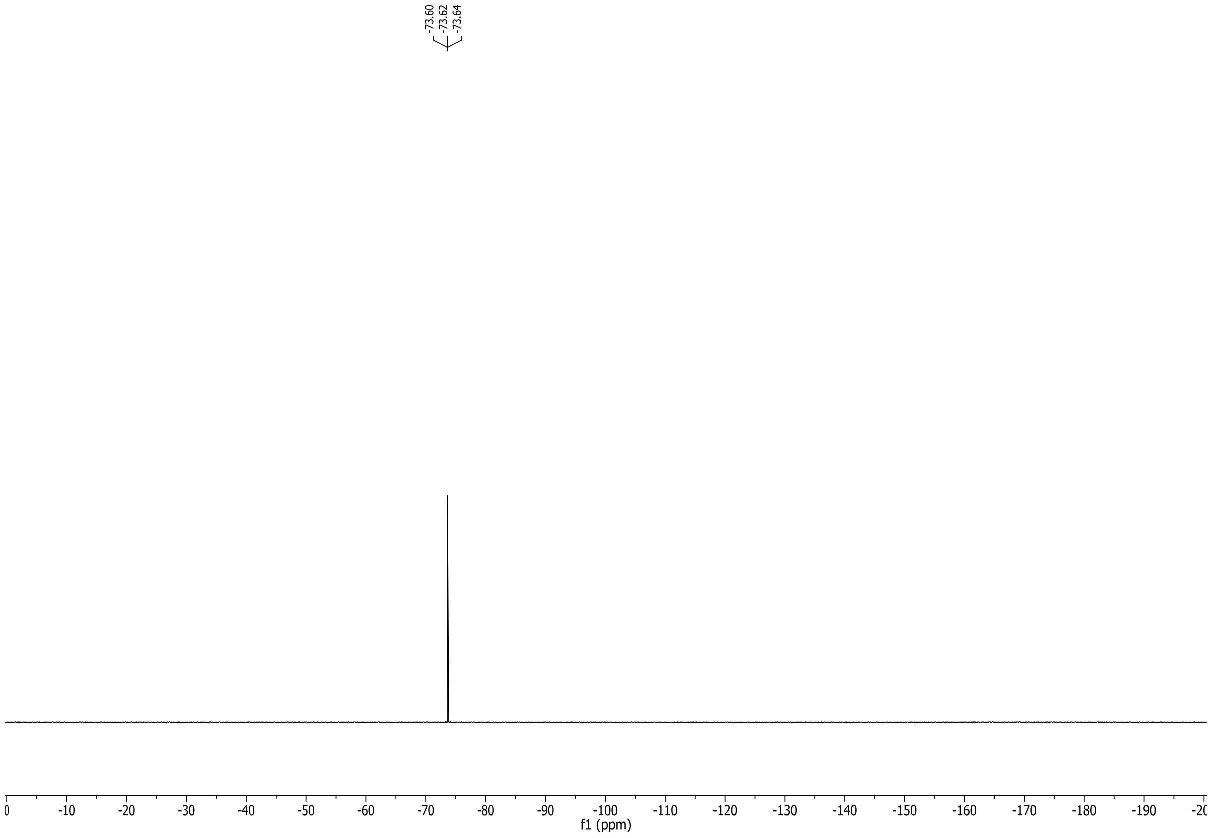
-73.60
-73.62
-73.64
f1 (ppm)

^{1}H and ^{13}C NMR data of compound **6f** (400, 101 MHz, $CDCl_3$)

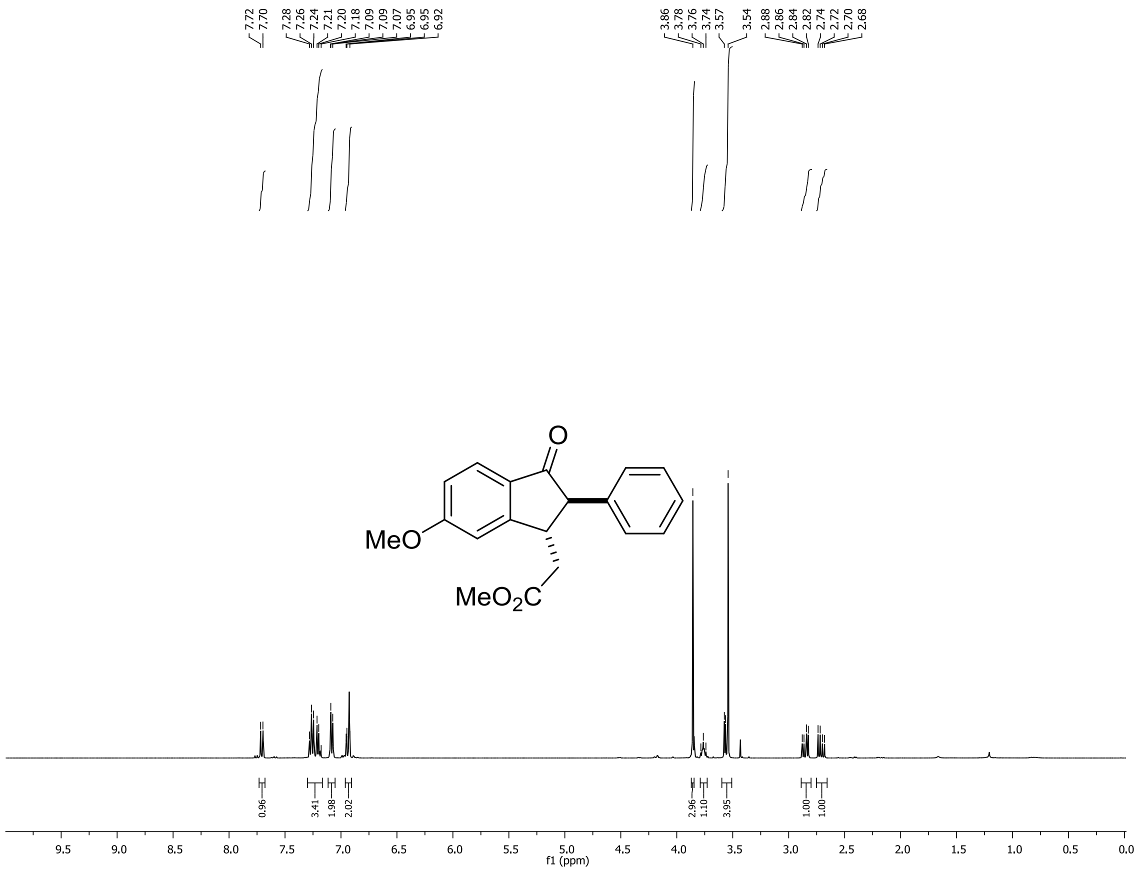

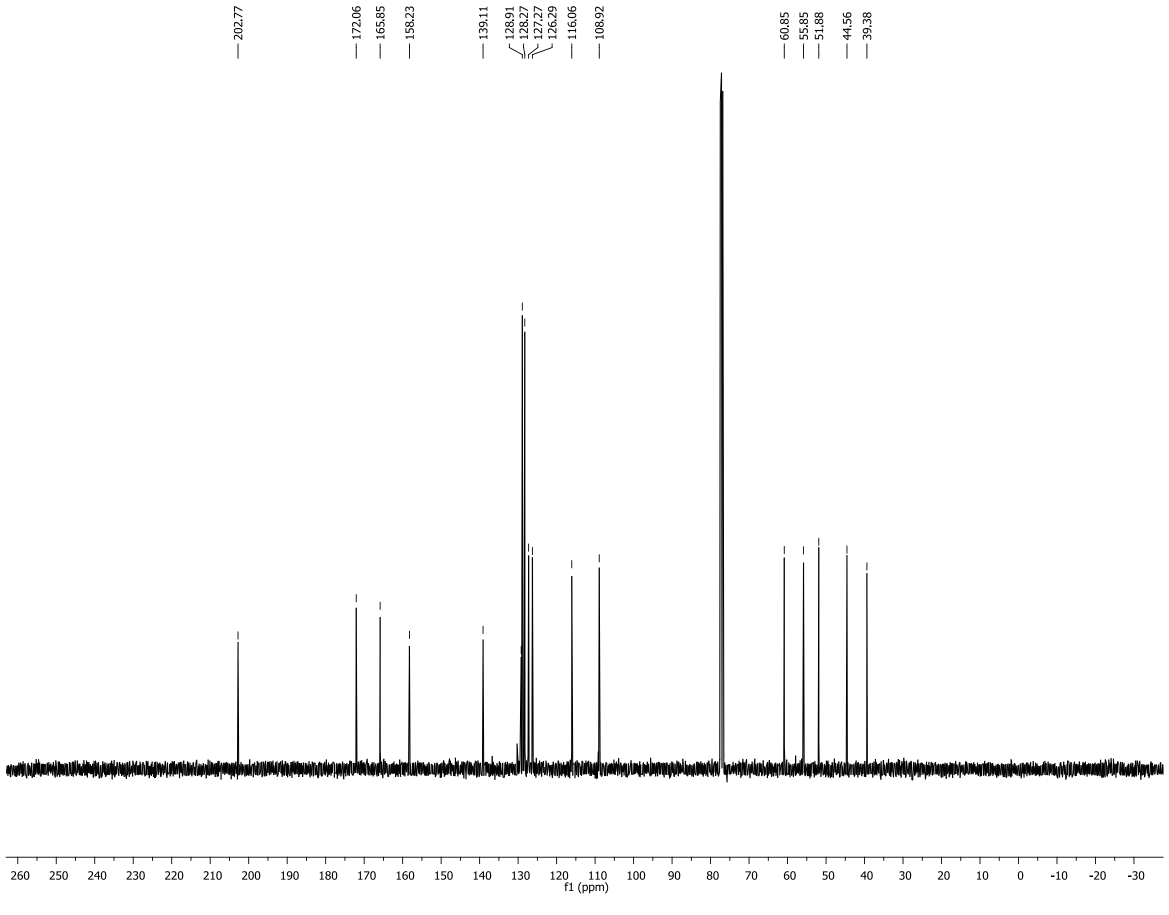

^{1}H and ^{13}C NMR data of compound **6g** (500, 125 MHz, $CDCl_3$)

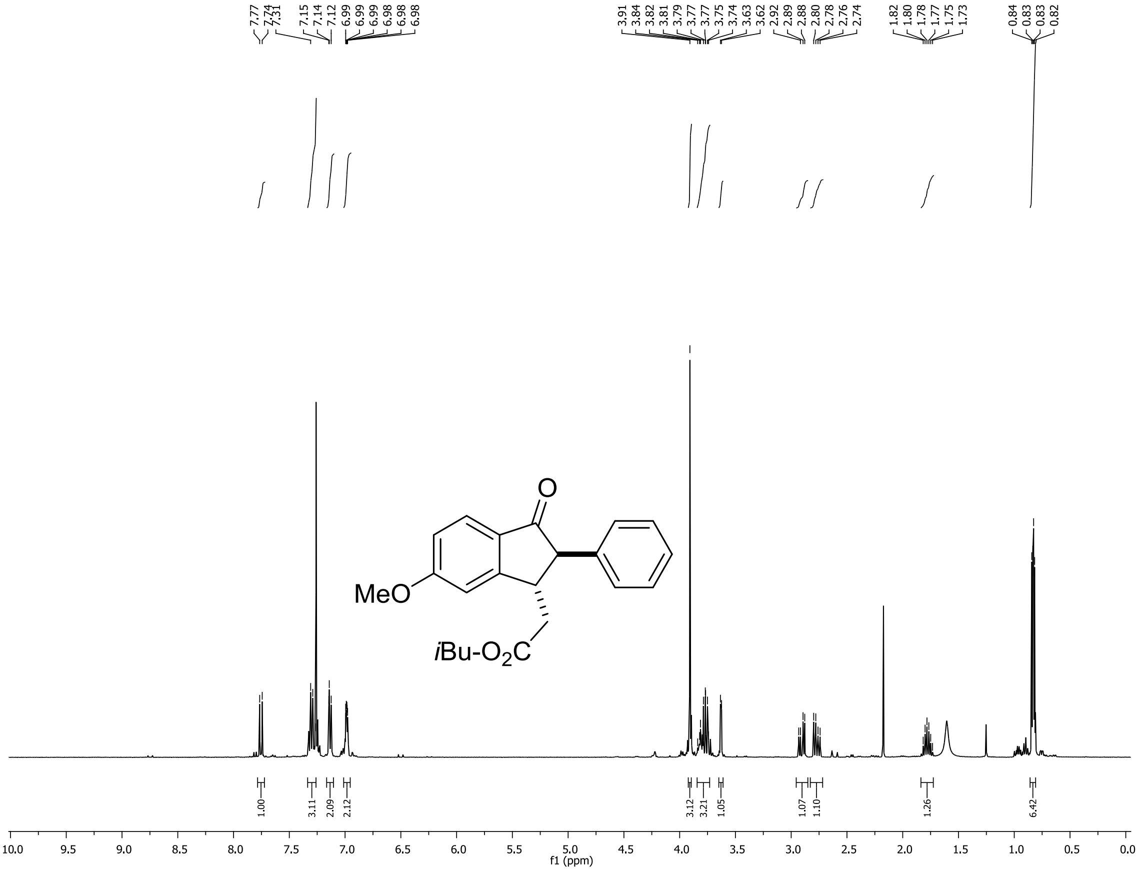

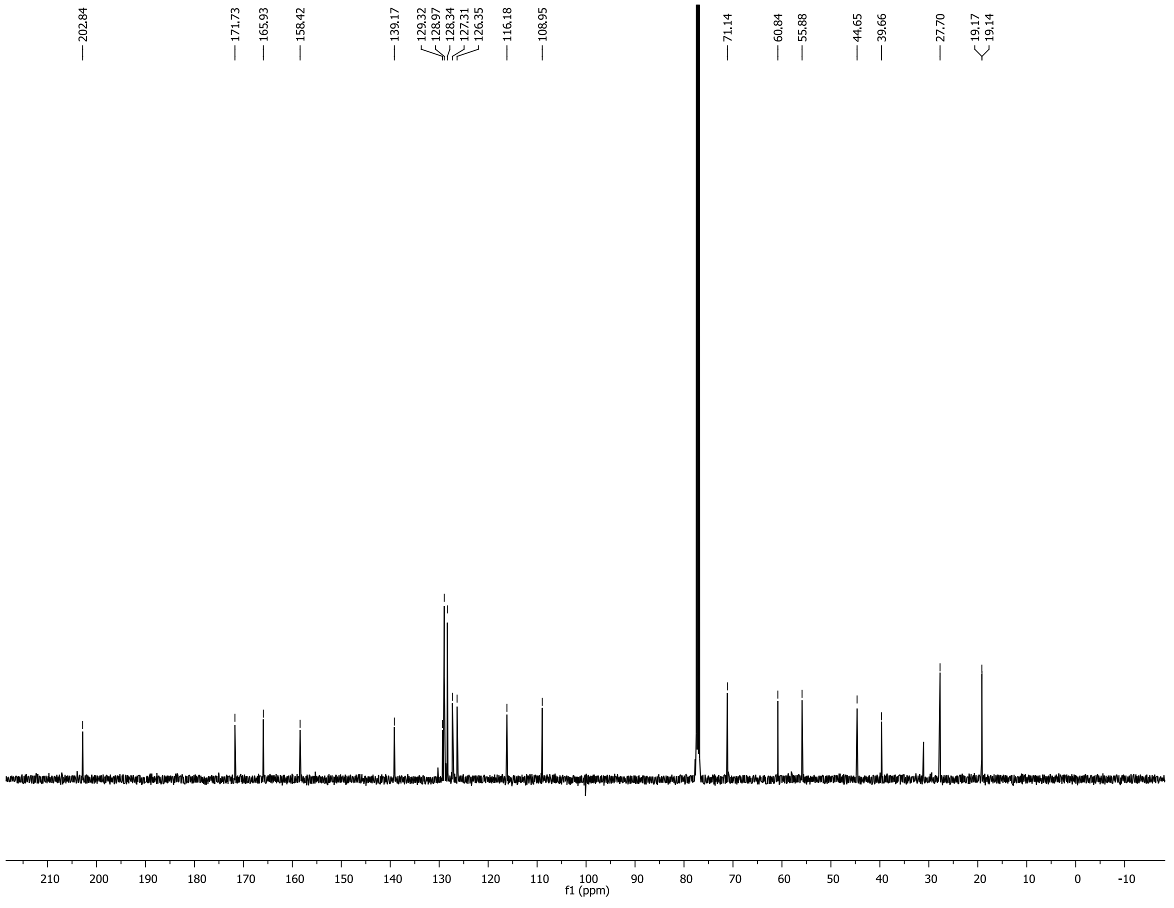

^{1}H and ^{13}C NMR data of compound **6h** (500, 125 MHz, $CDCl_3$)

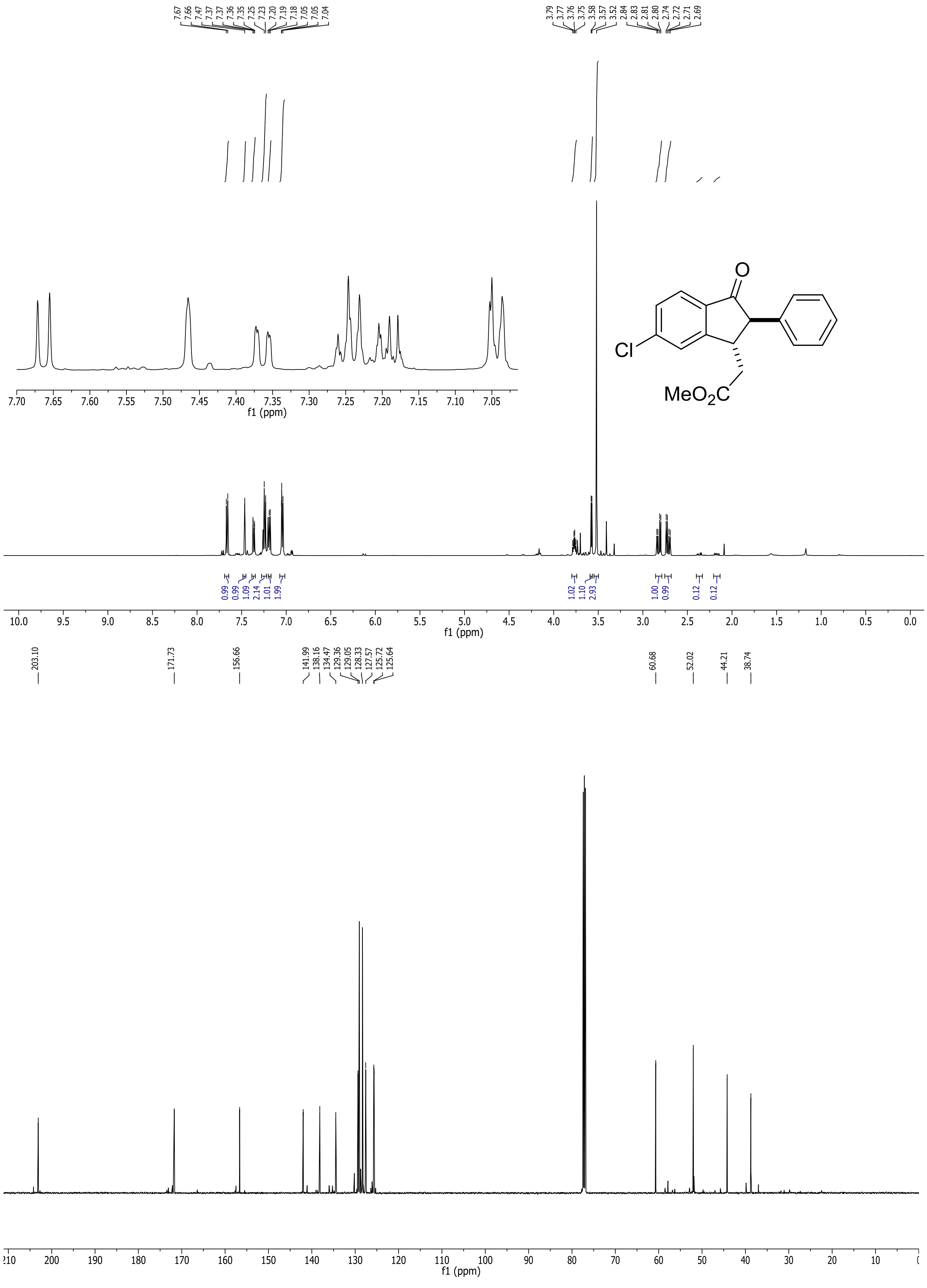

^{1}H and ^{13}C NMR data of compound **6i** (400, 101 MHz, $CDCl_3$)

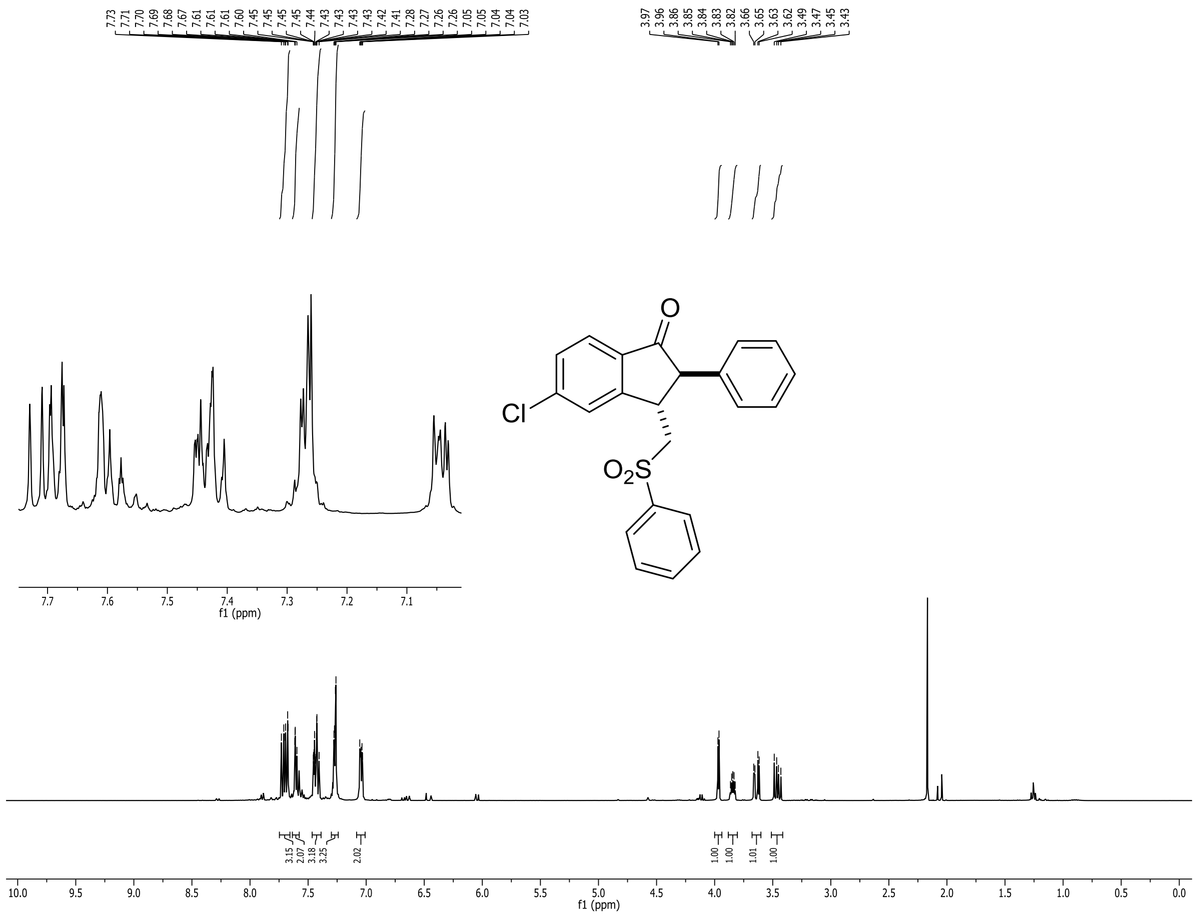

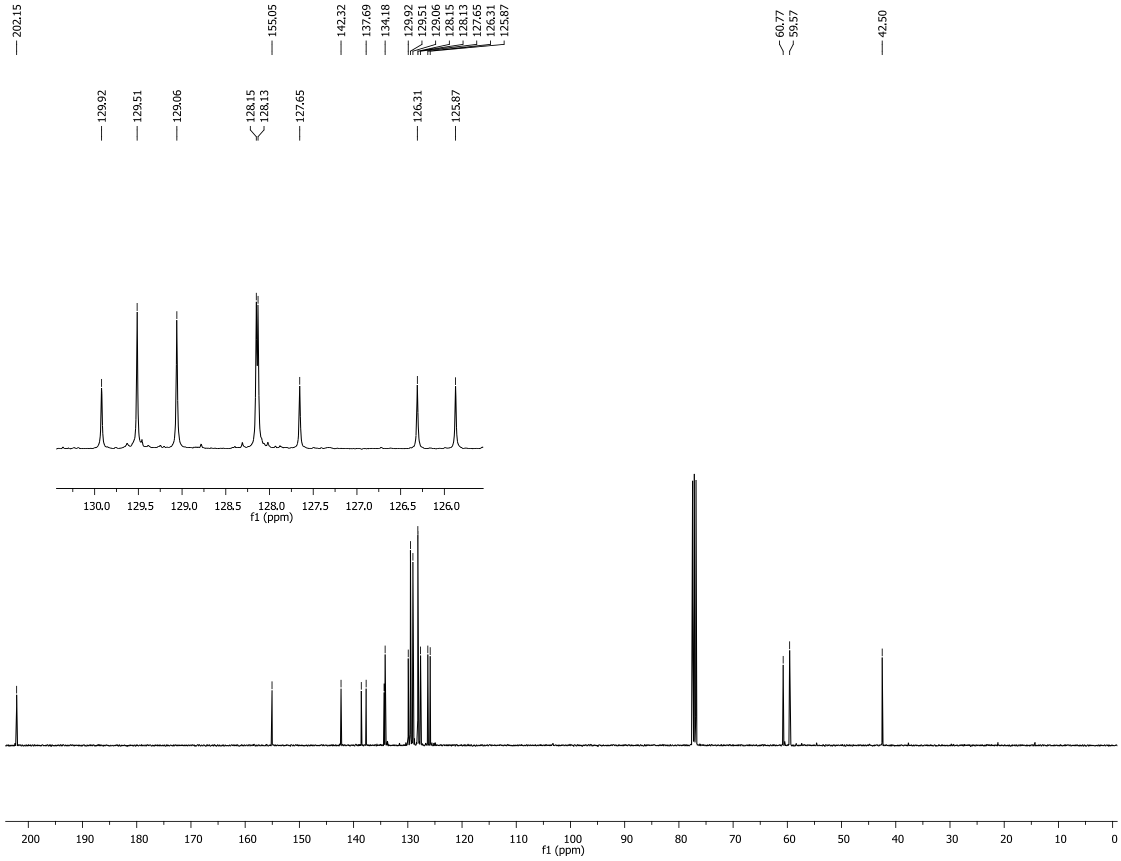

^{1}H and ^{13}C NMR data of compound **6j** (400, 101 MHz, $CDCl_3$)

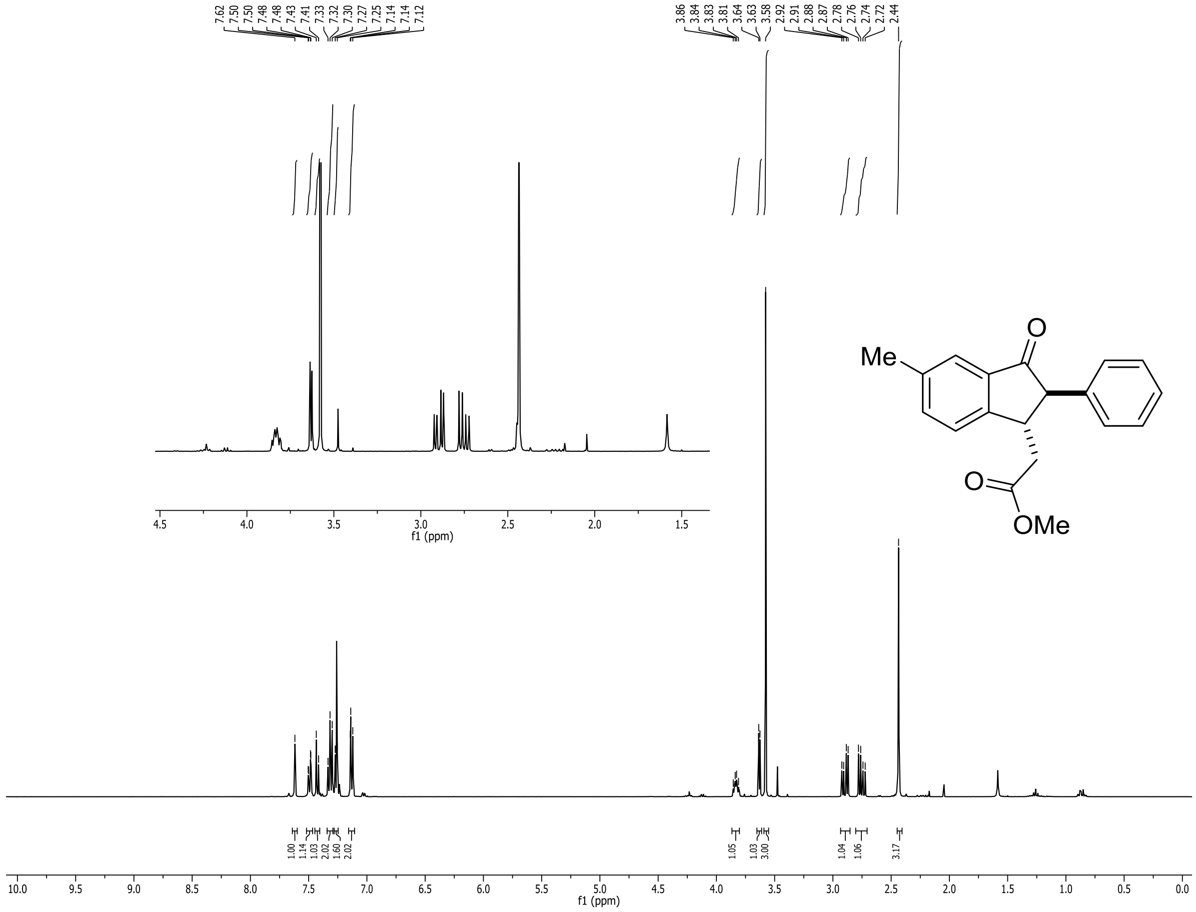

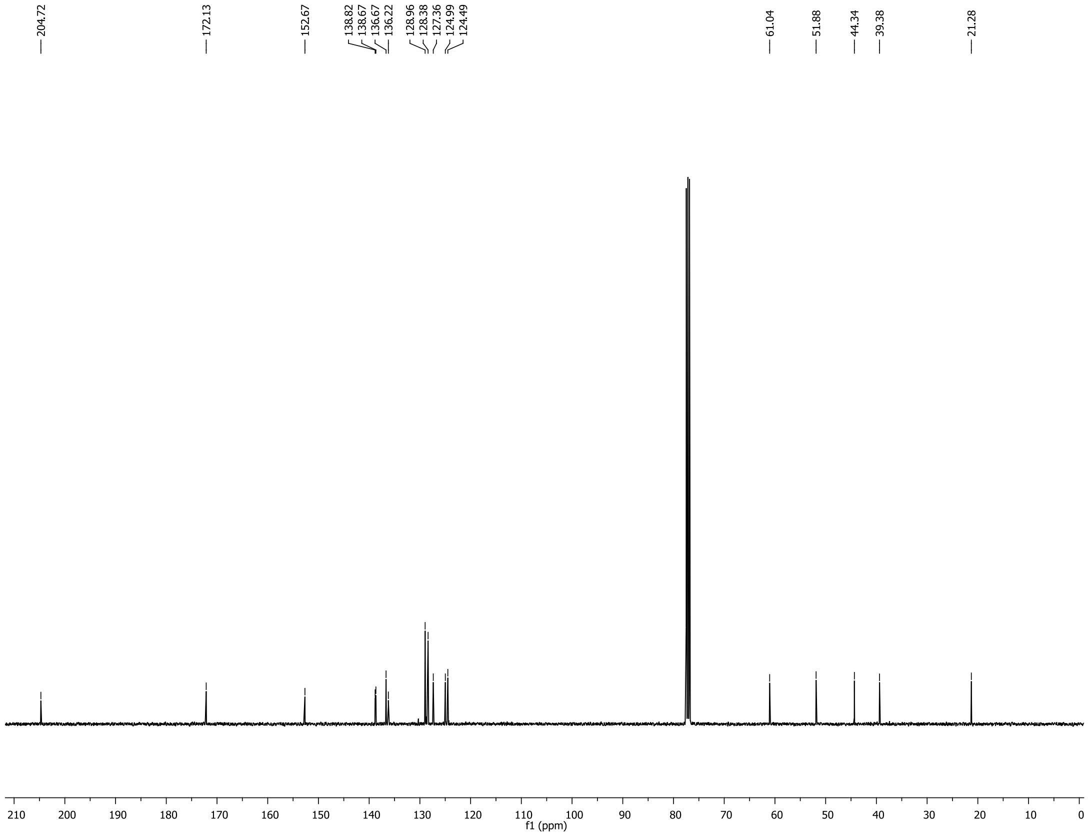

NOE spectra of compound **6j** (400 MHz, $CDCl_3$)

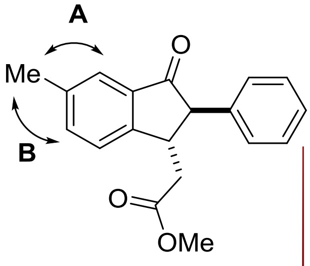

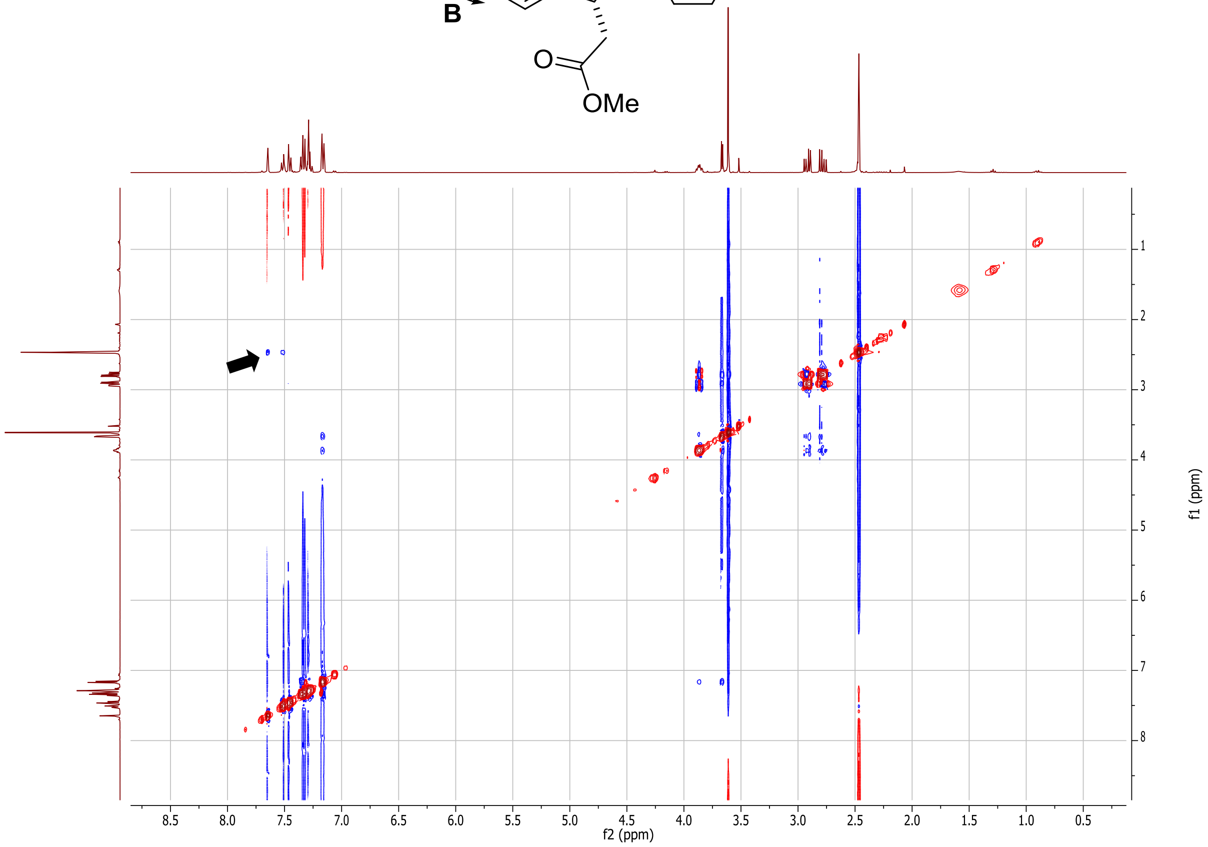

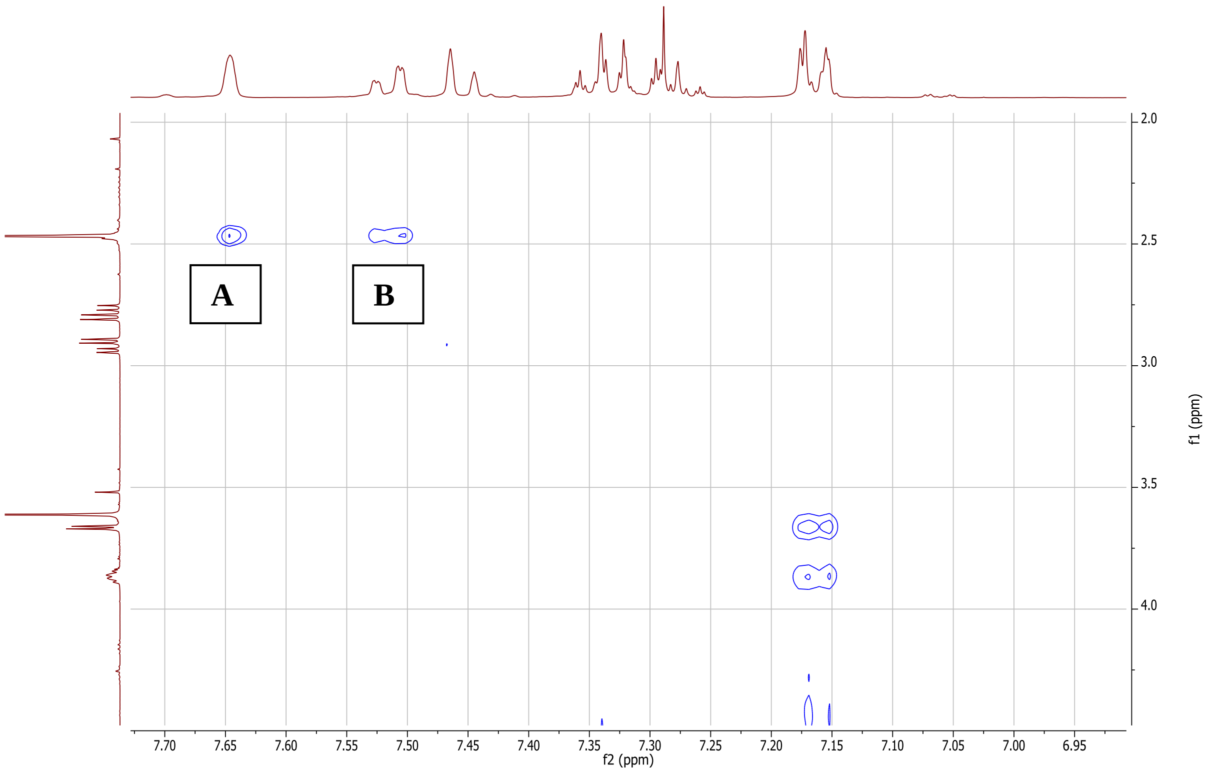

^{1}H, ^{13}C and ^{19}F NMR data of compound **6k** (400, 101, 376 MHz, $CDCl_3$)

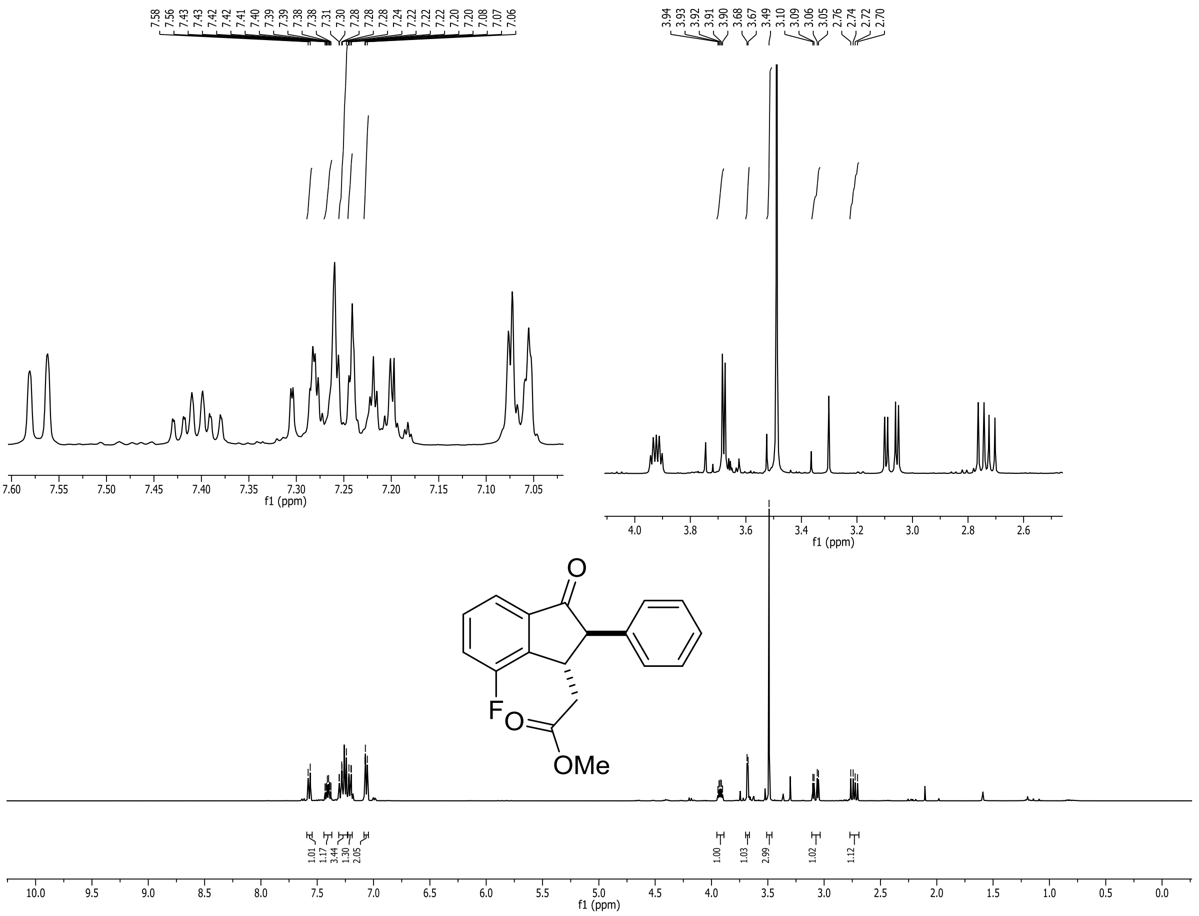

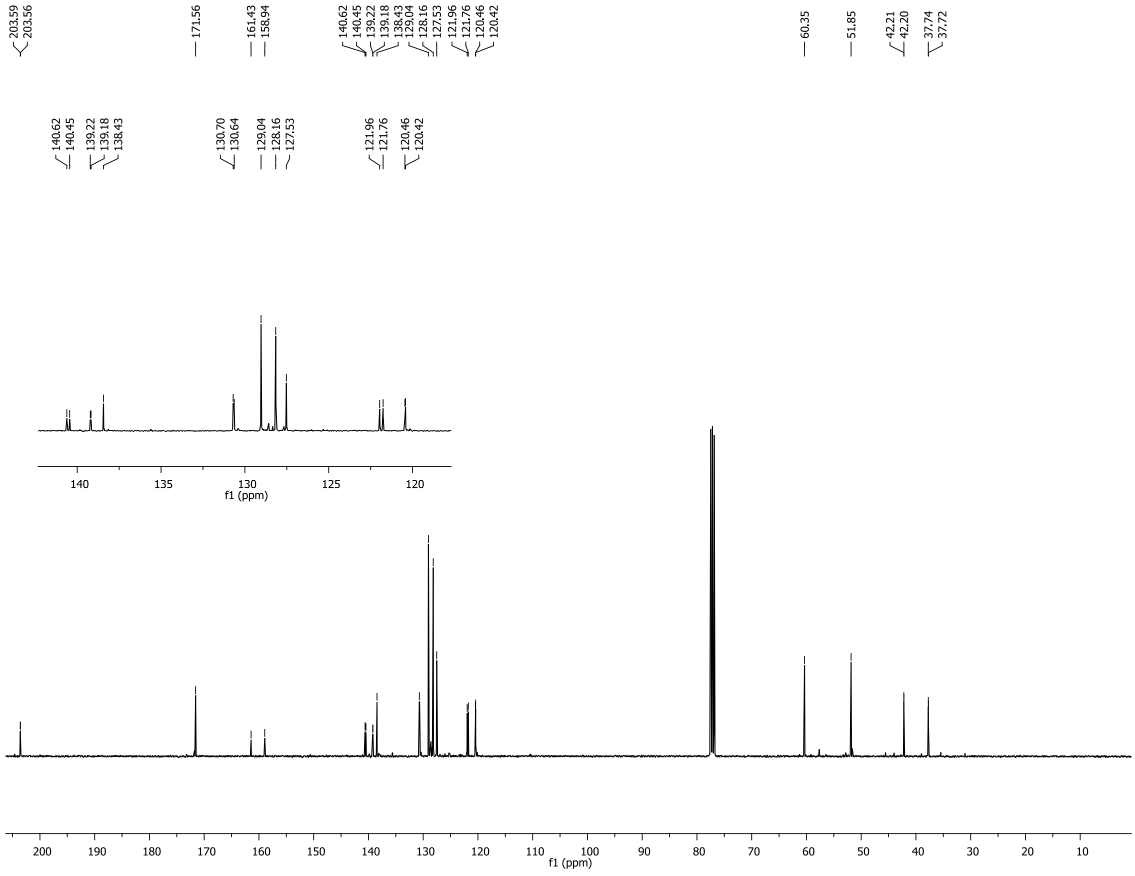

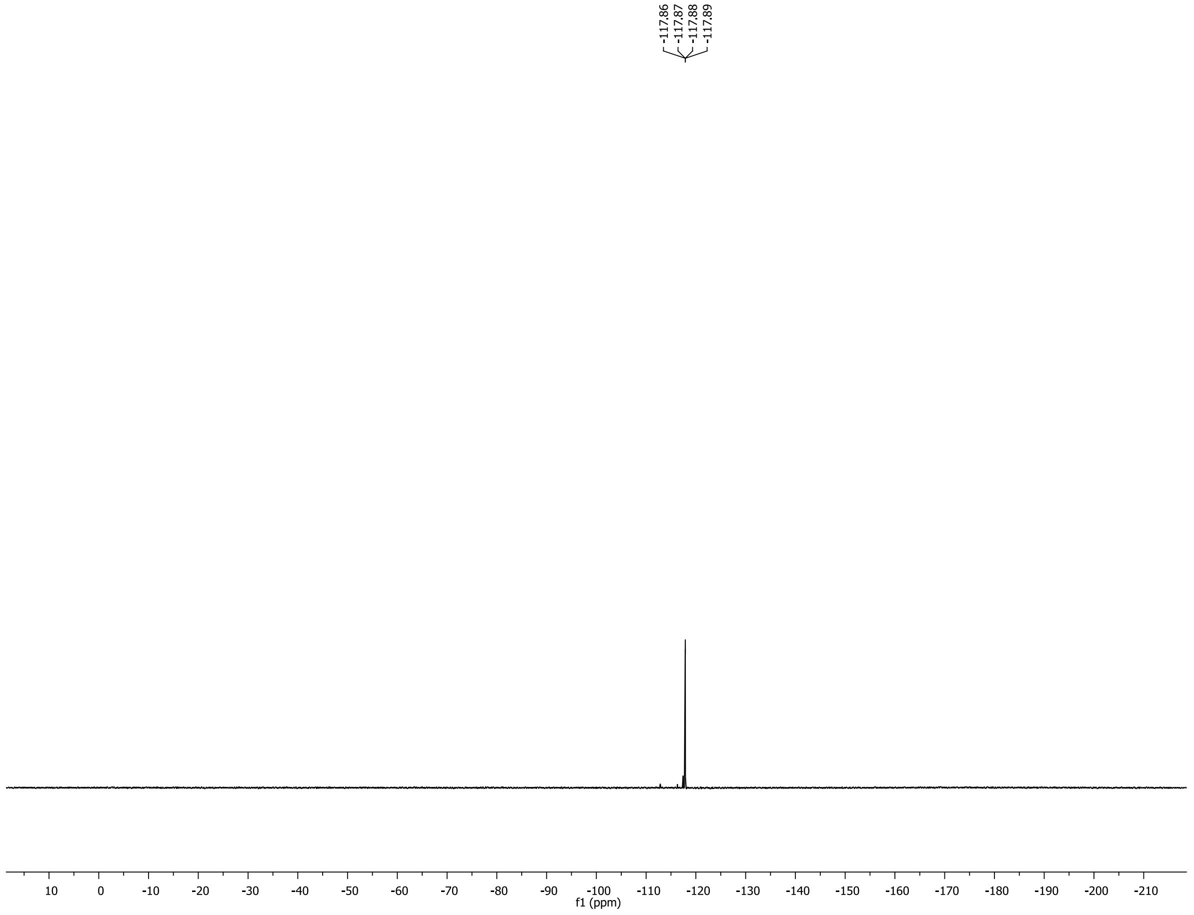

^{1}H and ^{13}C NMR data of compound **6l** (500, 125 MHz, $CDCl_3$)

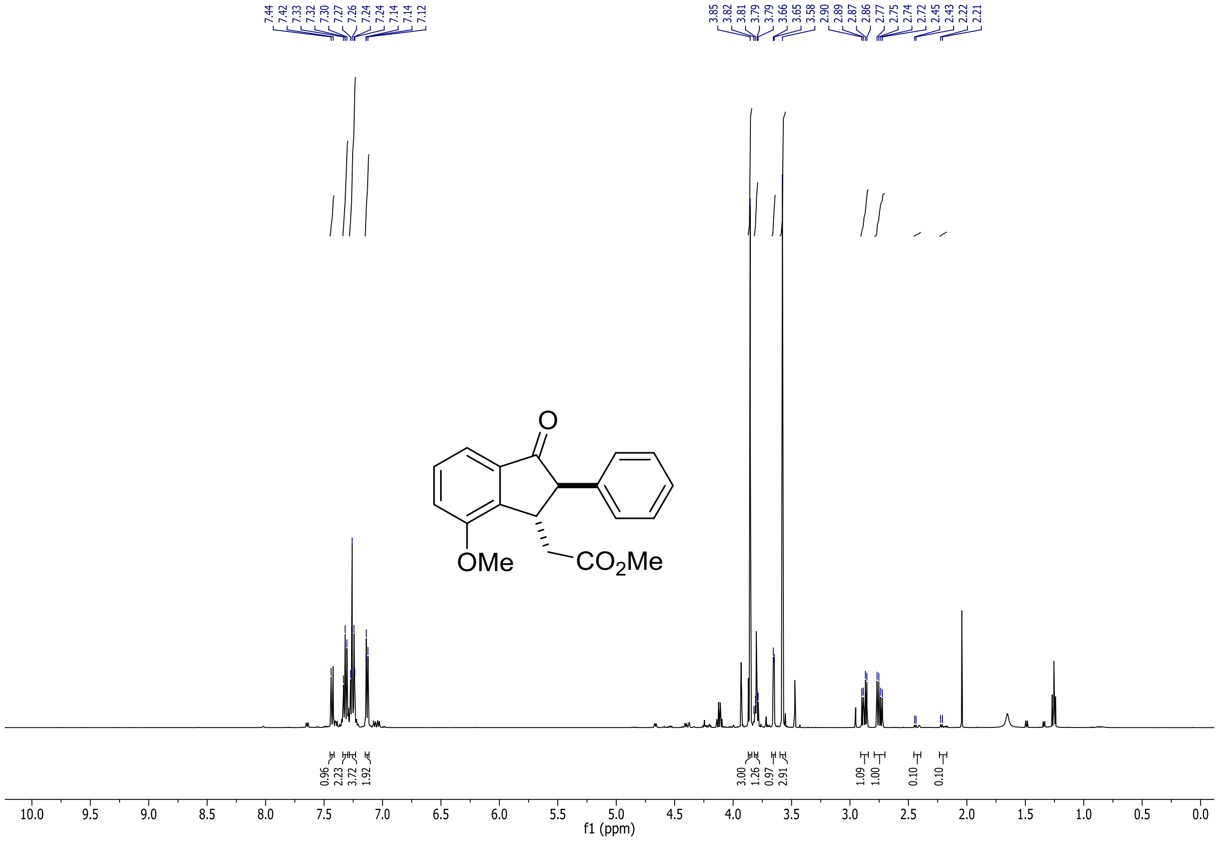

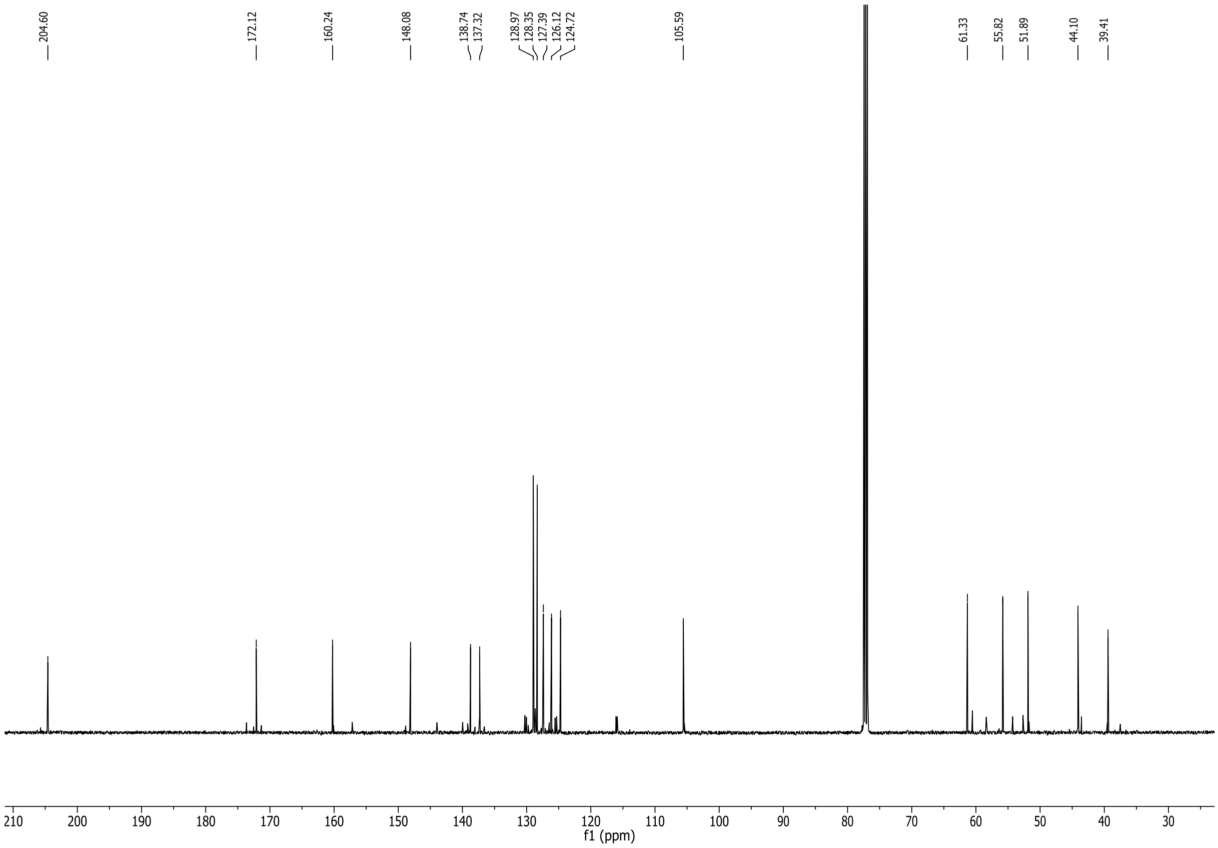

^{1}H and ^{13}C NMR data of compound **6m** (400, 101 MHz, $CDCl_3$)

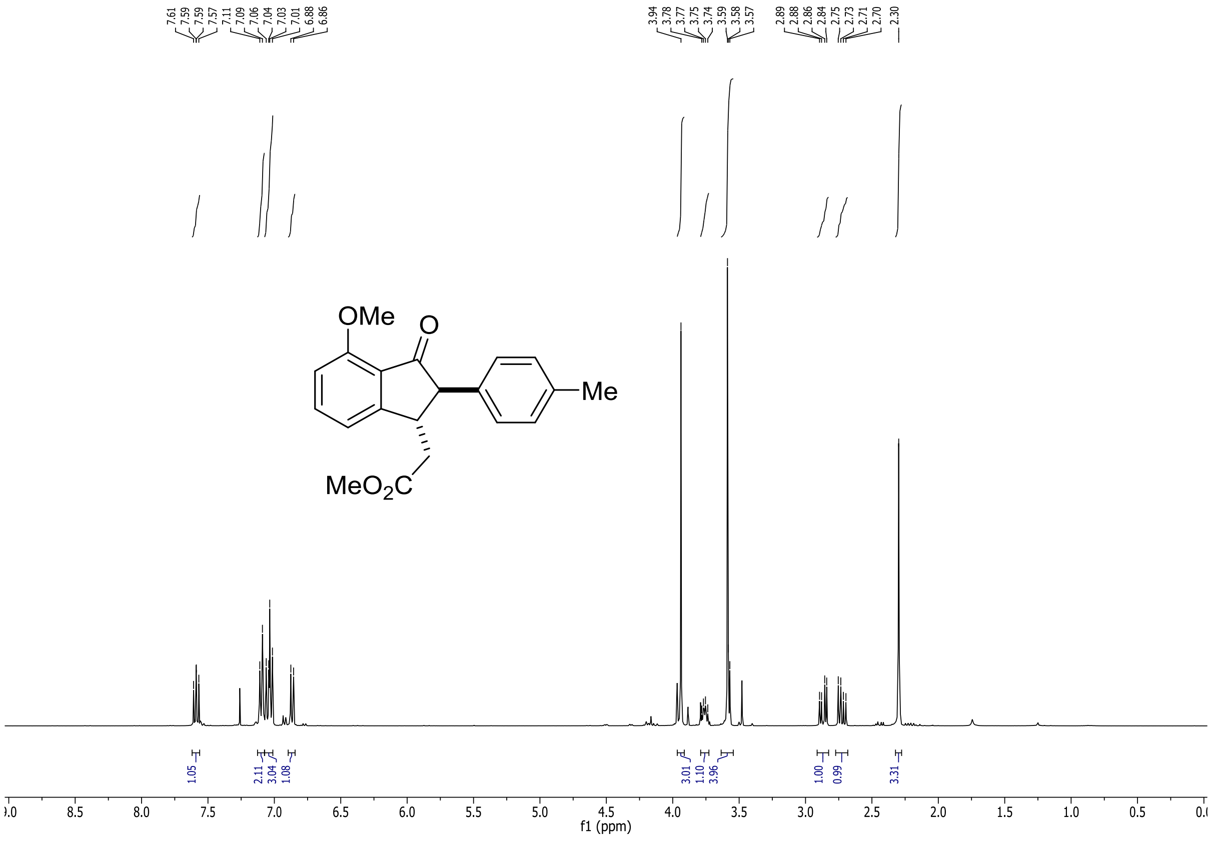

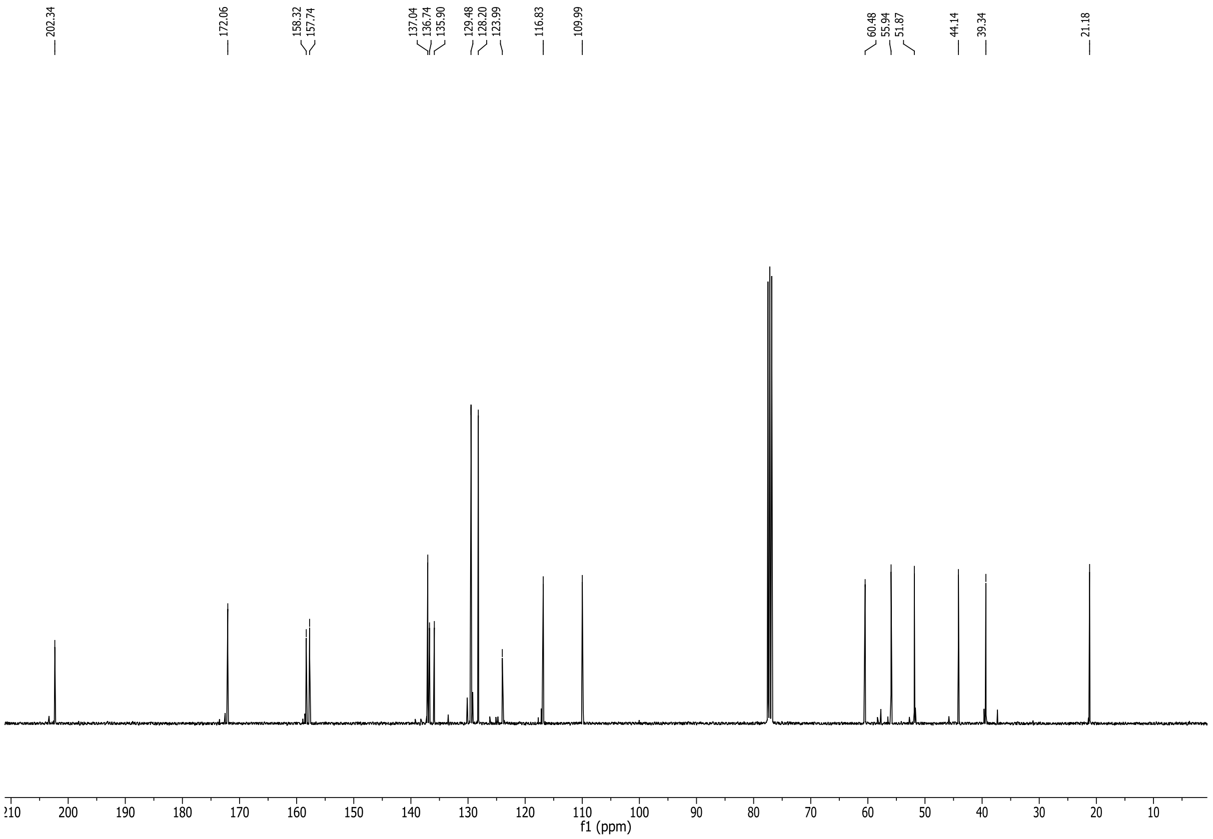

202.34
172.06
158.32
157.74
137.04
136.74
135.90
129.48
128.20
123.99
116.83
109.99
60.48
55.94
51.87
44.14
39.34
21.18
f1 (ppm)

^{1}H and ^{13}C NMR data of compound **6n** (400, 101 MHz, $CDCl_3$)

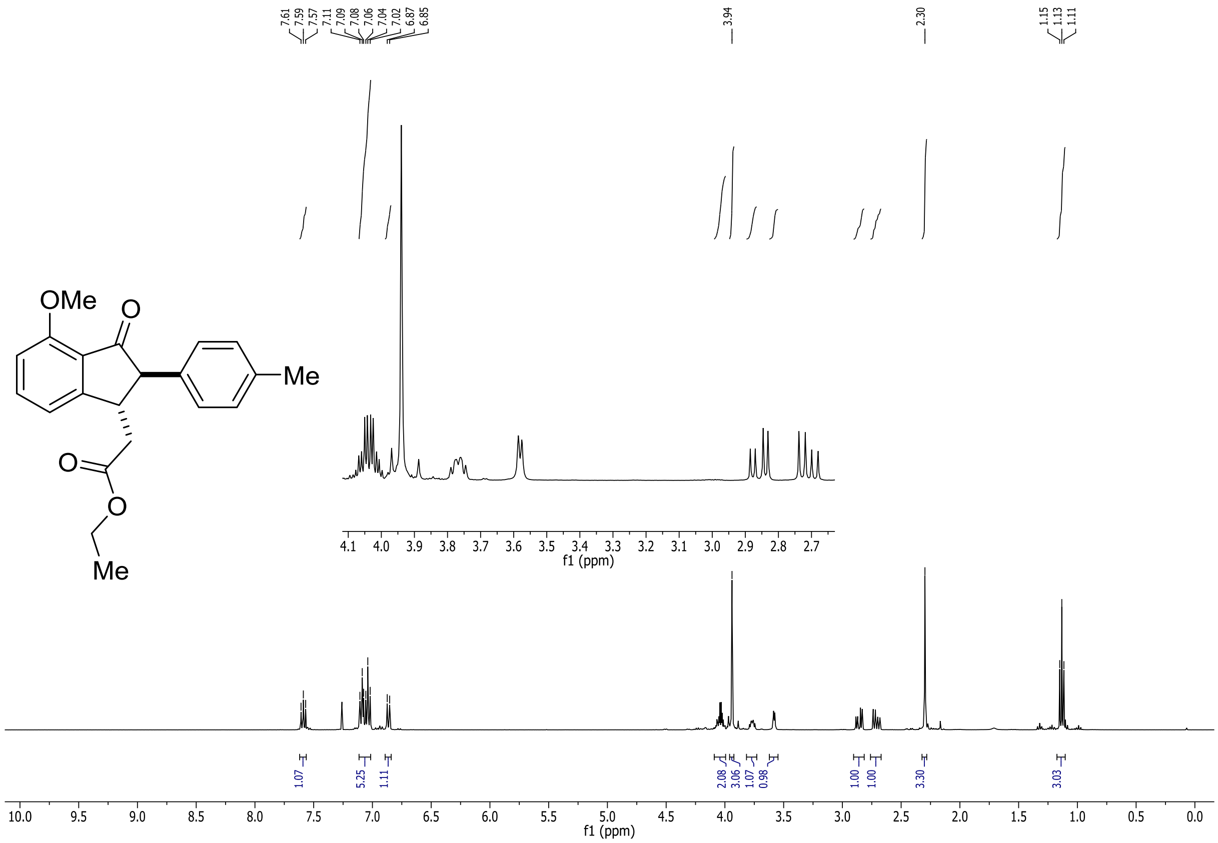

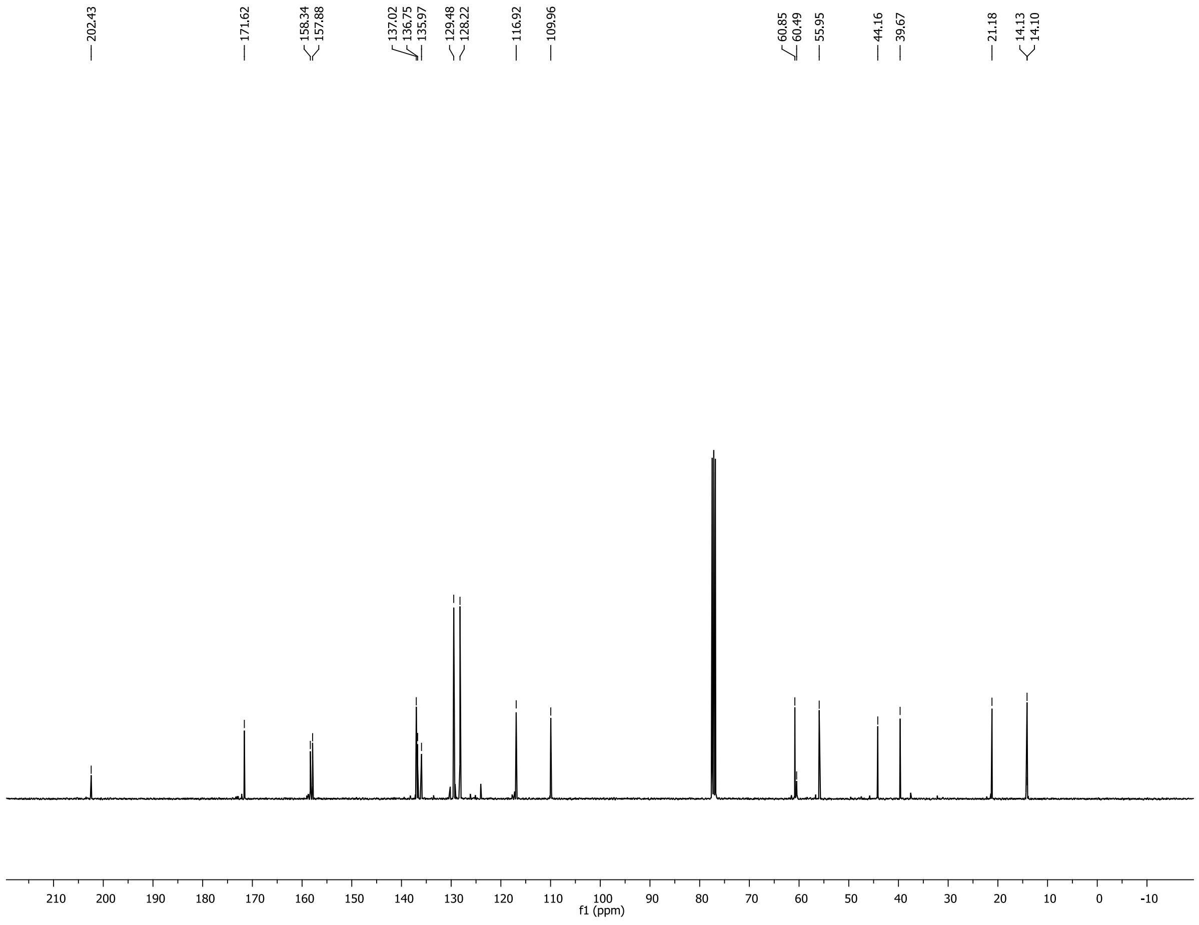

^{1}H, and ^{13}C data of compound **6o** (300, 75 MHz, $CDCl_3$)

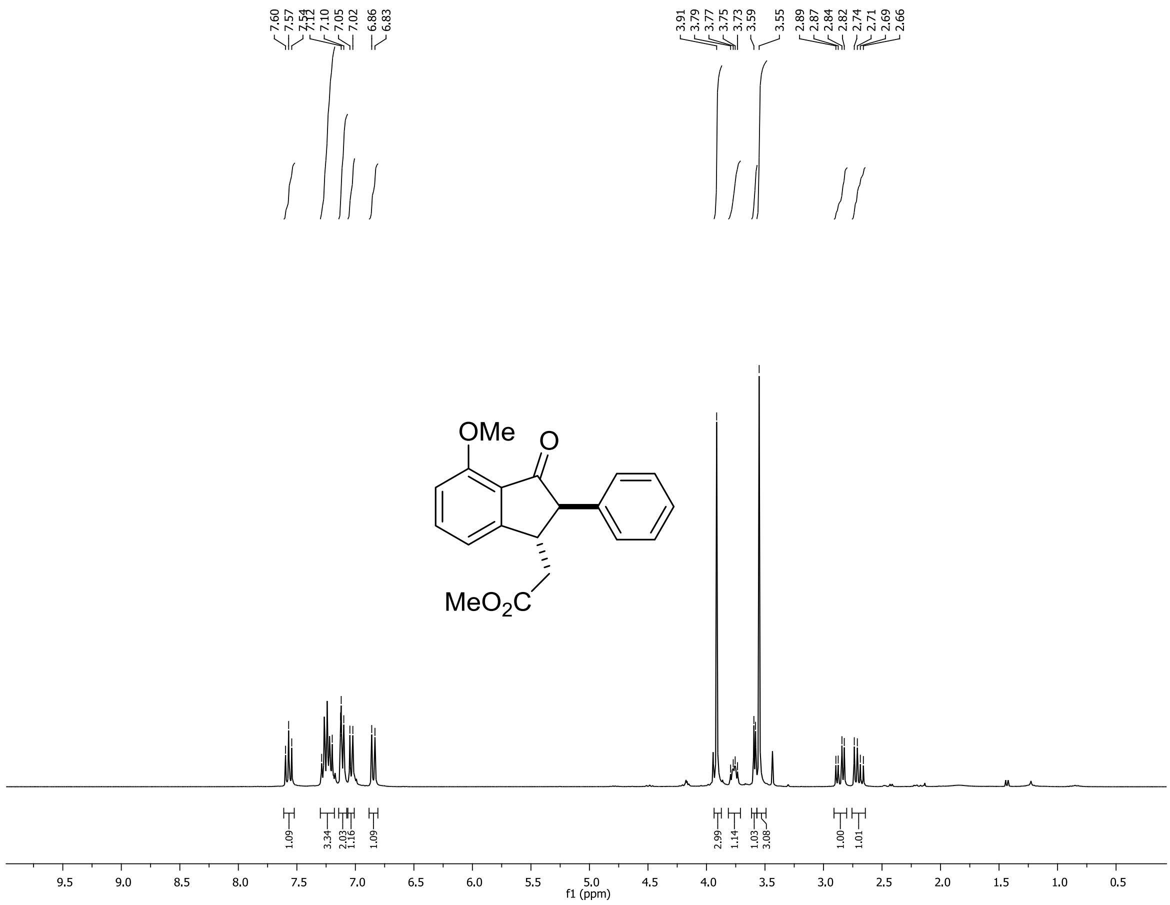

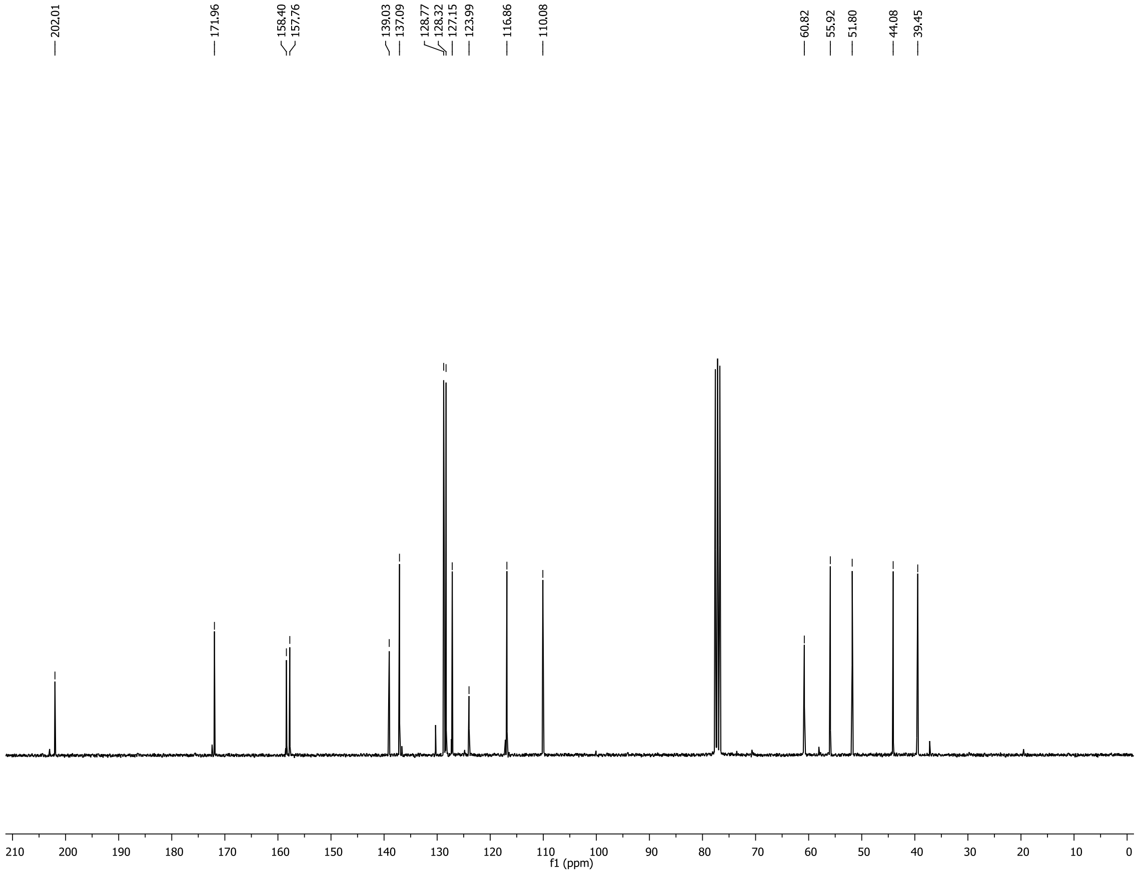

^{1}H, and ^{13}C data of compound **7o** (300, 75 MHz, $CDCl_3$)

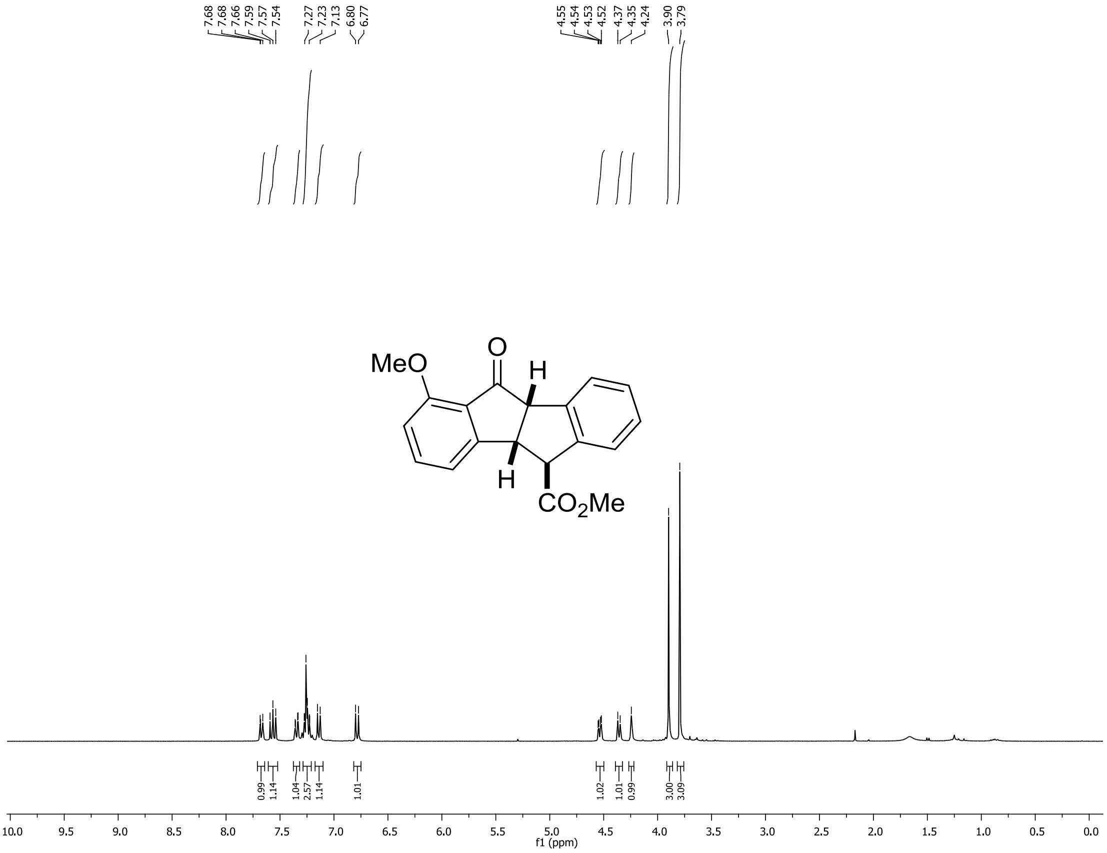
7.68
7.68
7.66
7.59
7.57
7.54
7.27
7.23
7.13
6.80
6.77
4.55
4.54
4.53
4.52
4.37
4.35
4.24
3.90
3.79
MeO
O
H
H
CO2Me
0.99
1.14
1.04
2.57
1.14
1.01
1.02
1.01
0.99
3.00
3.09
10.0
9.5
9.0
8.5
8.0
7.5
7.0
6.5
6.0
5.5
5.0
4.5
4.0
3.5
3.0
2.5
2.0
1.5
1.0
0.5
0.0
f1 (ppm)

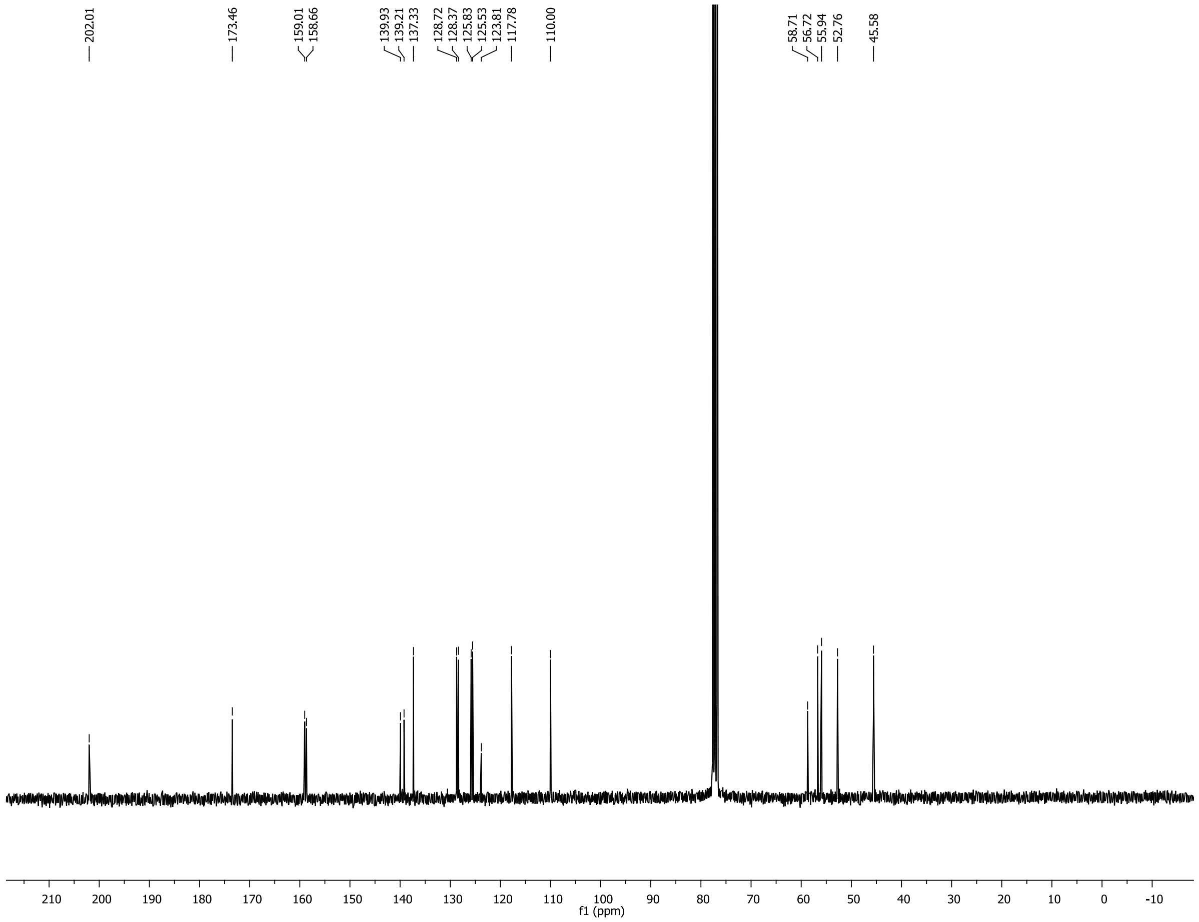
202.01
173.46
159.01
158.66
139.93
139.21
137.33
128.72
128.37
125.83
125.53
123.81
117.78
110.00
58.71
56.72
55.94
52.76
45.58
210
200
190
180
170
160
150
140
130
120
110
100
90
80
70
60
50
40
30
20
10
0
-10
f1 (ppm)

^{1}H and ^{13}C NMR data of compound **6p** (500, 125 MHz, $CDCl_3$)

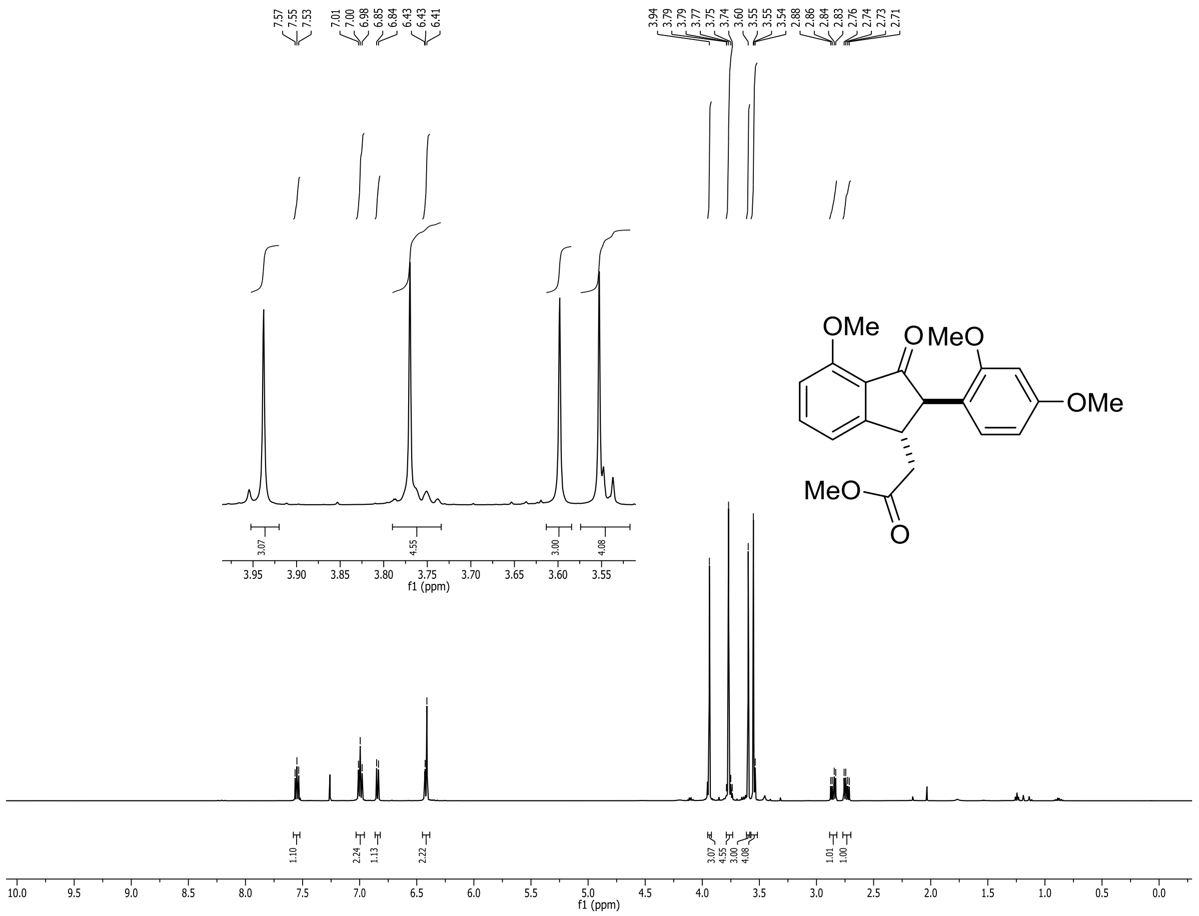

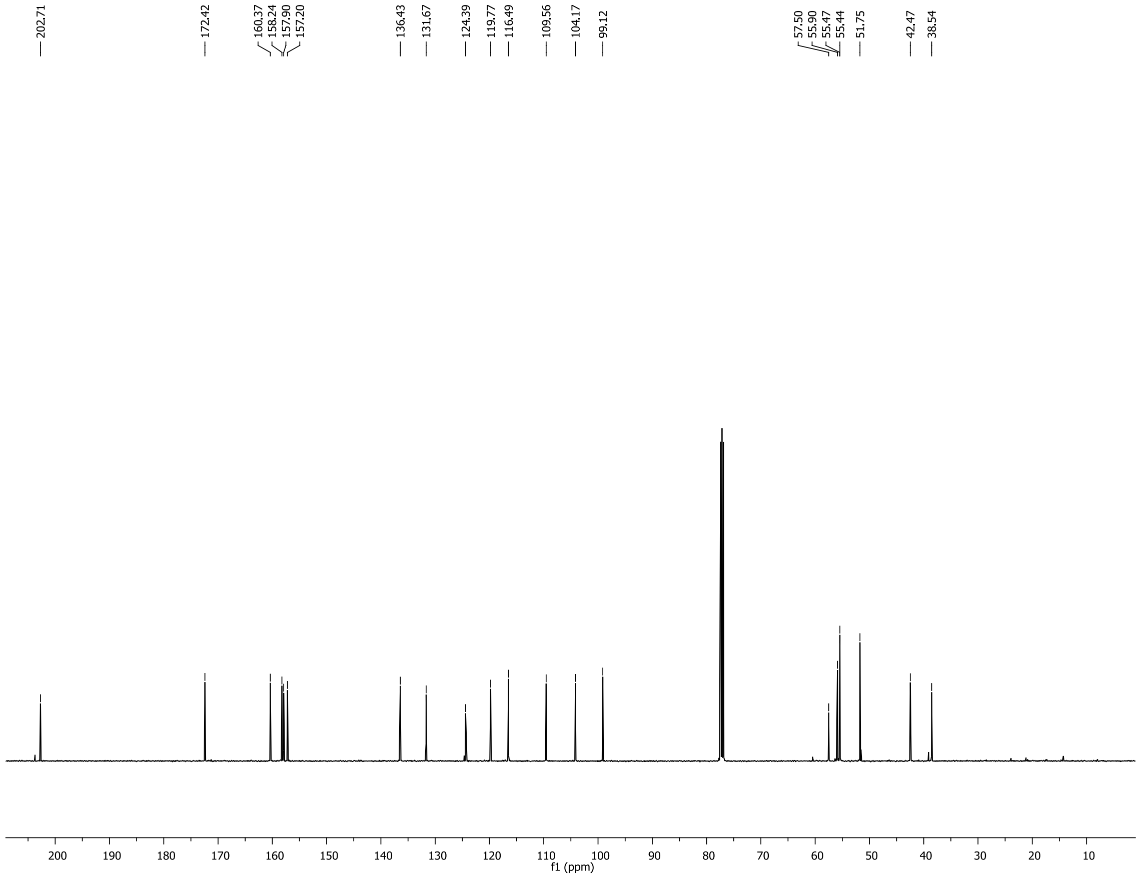

^{1}H, ^{13}C and ^{19}F NMR data of compound **6q** (400, 101, 376 MHz, $CDCl_3$)

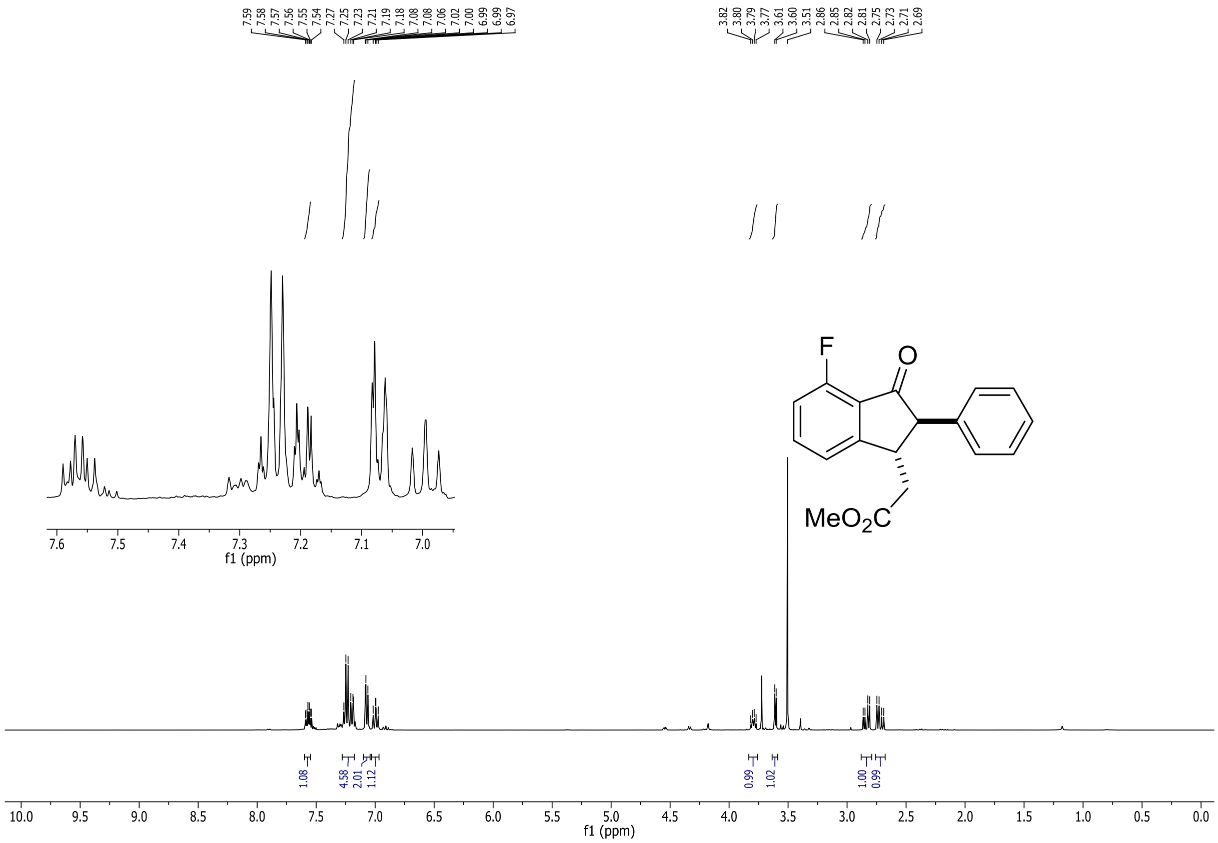

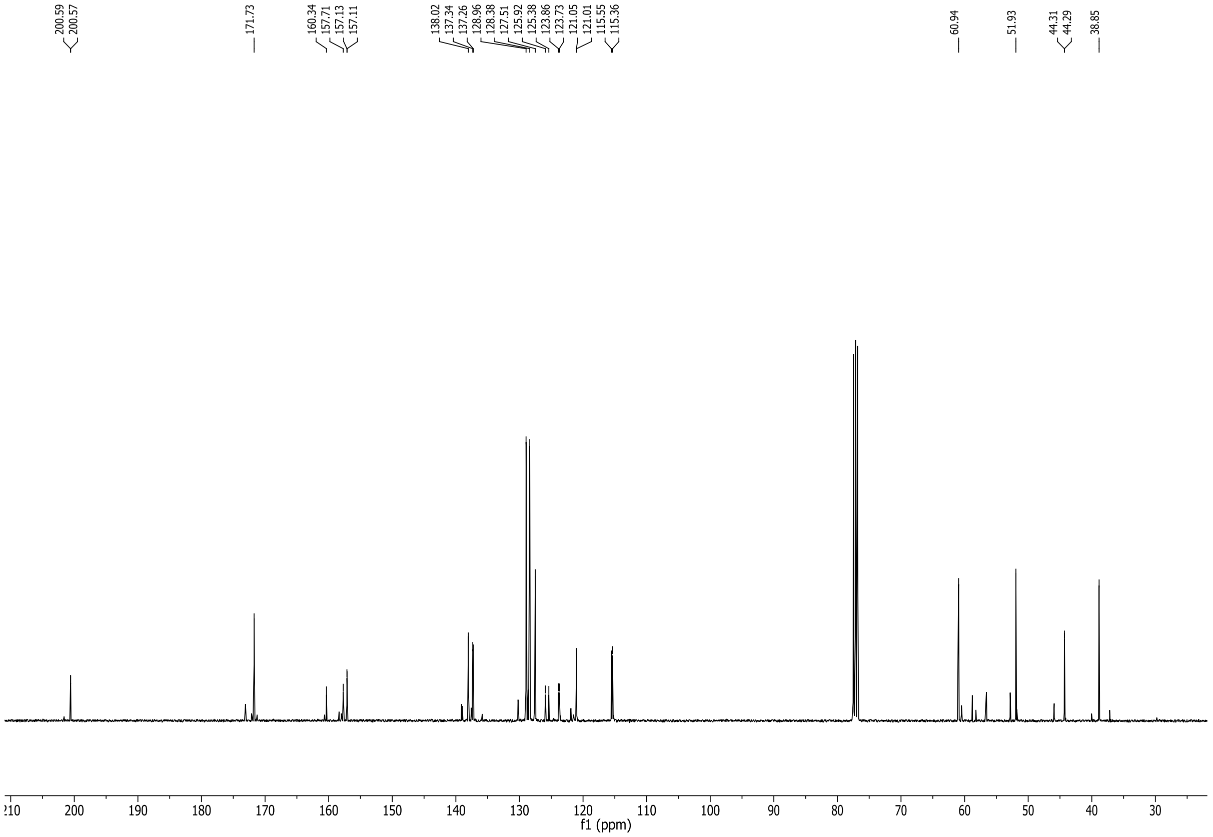

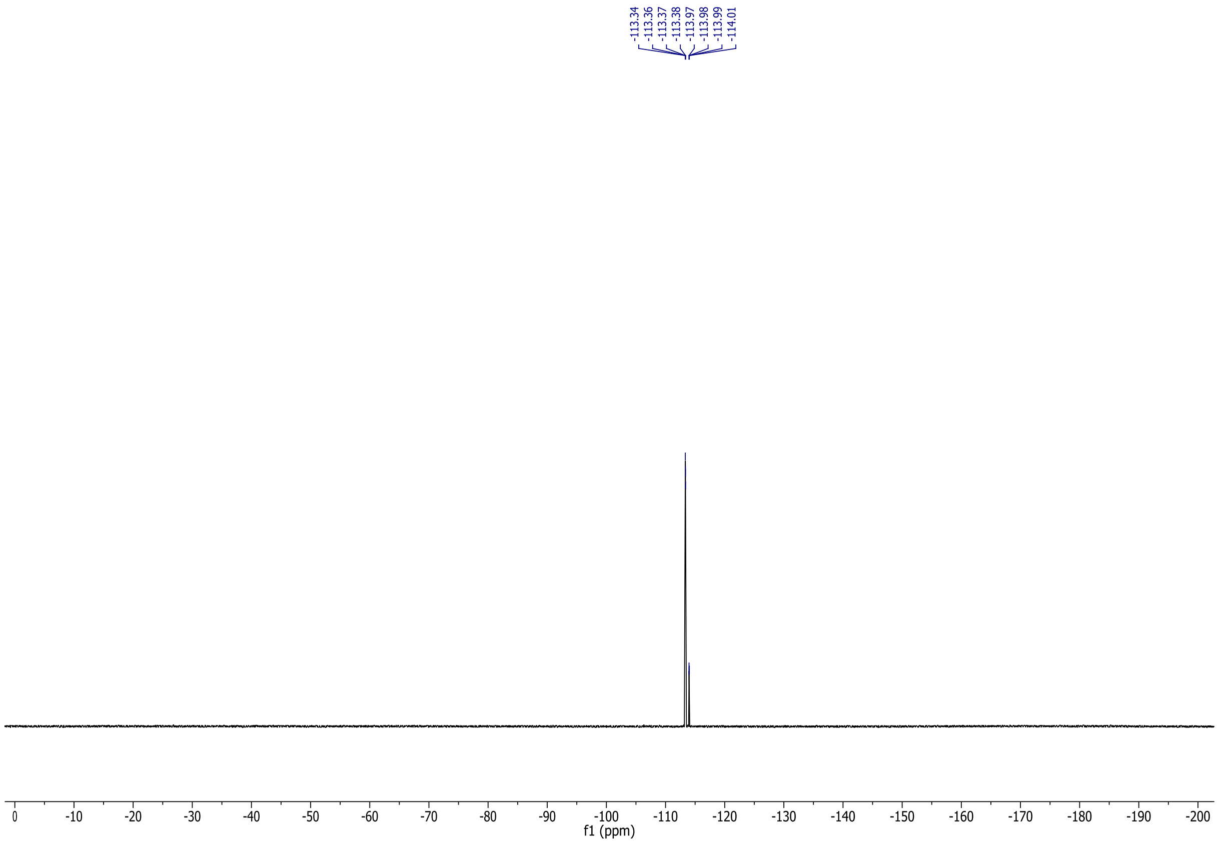

^{1}H, ^{13}C and ^{19}F NMR data of compound **6r** (500, 125, 376 MHz, $CDCl_3$)

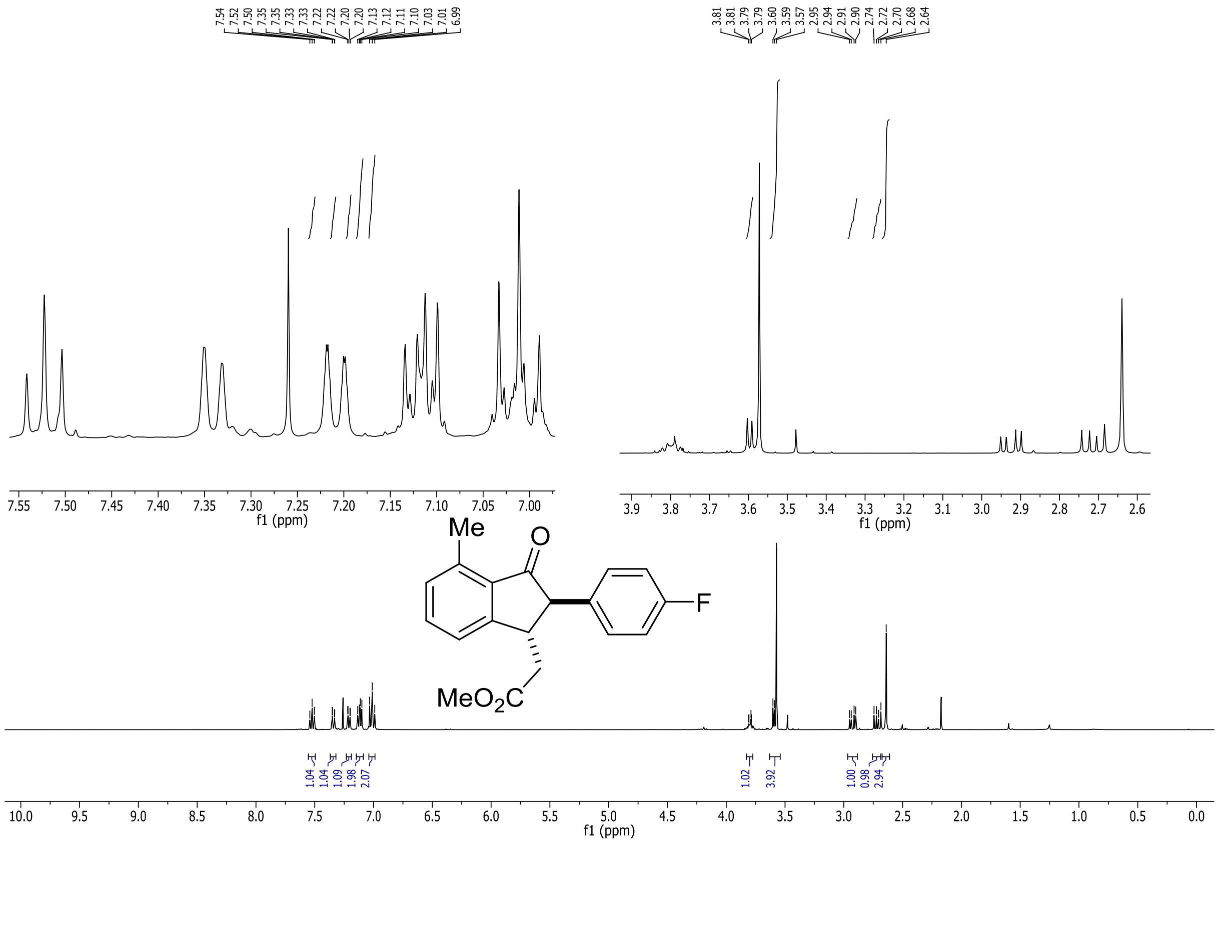

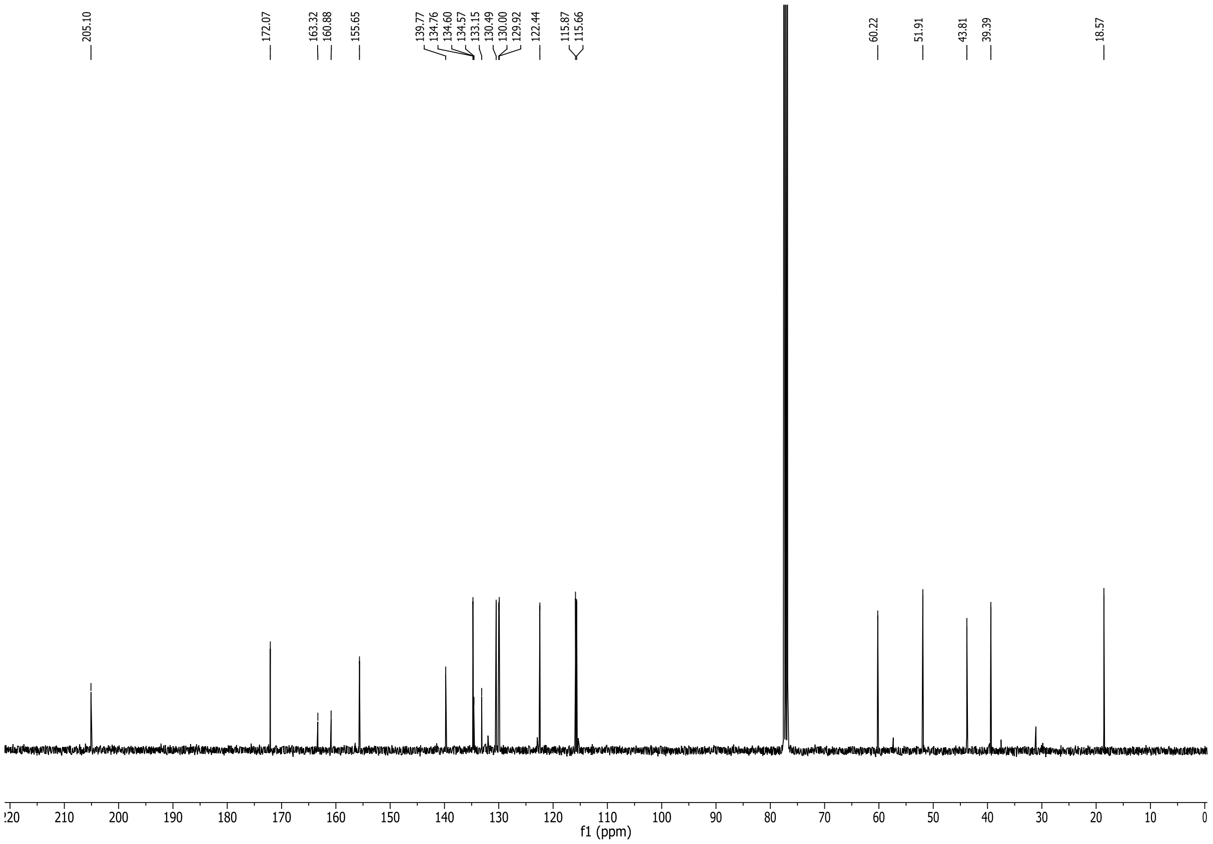
205.10
172.07
163.32
160.88
155.65
139.77
134.76
134.60
134.57
133.15
130.49
130.00
129.92
122.44
115.87
115.66
60.22
51.91
43.81
39.39
18.57
f1 (ppm)

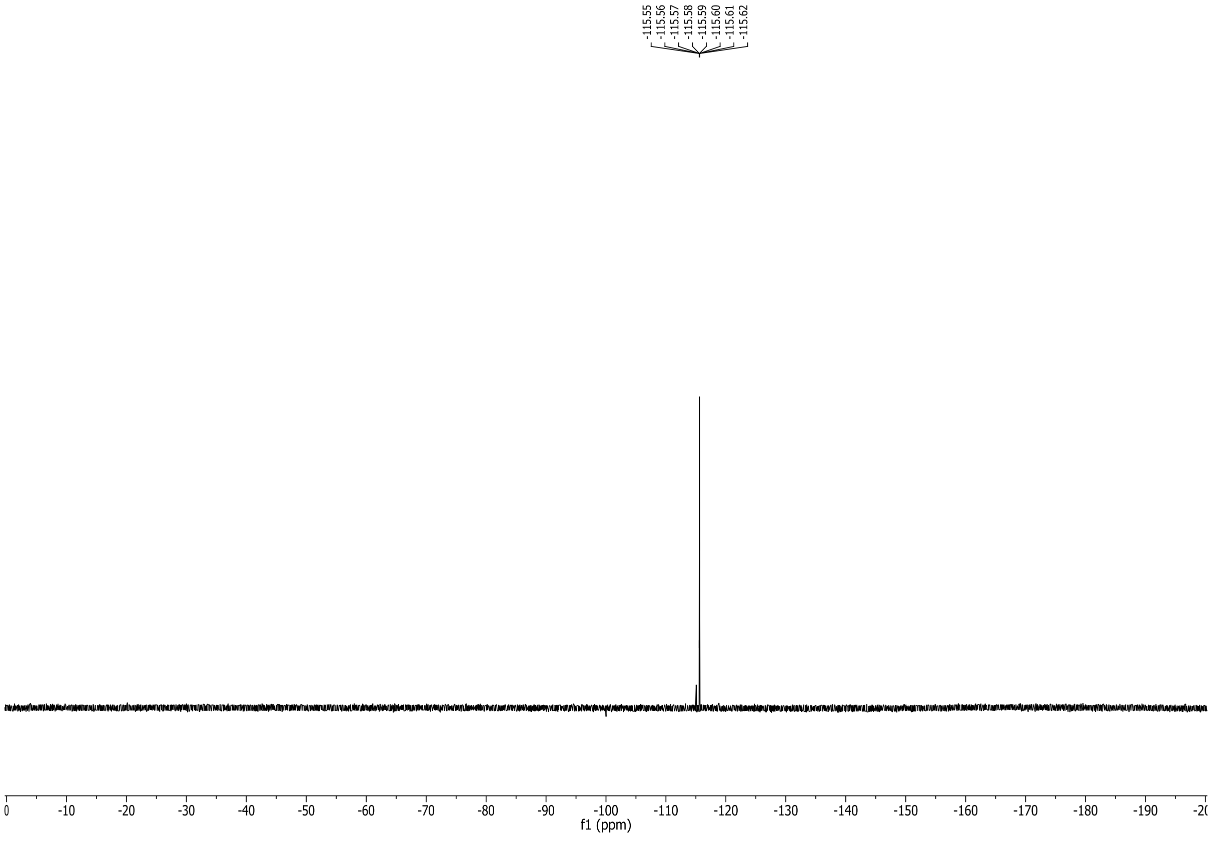
-115.55
-115.56
-115.57
-115.58
-115.59
-115.60
-115.61
-115.62
f1 (ppm)

^{1}H and ^{13}C NMR data of compound **6s** (300, 75 MHz, $CDCl_3$)

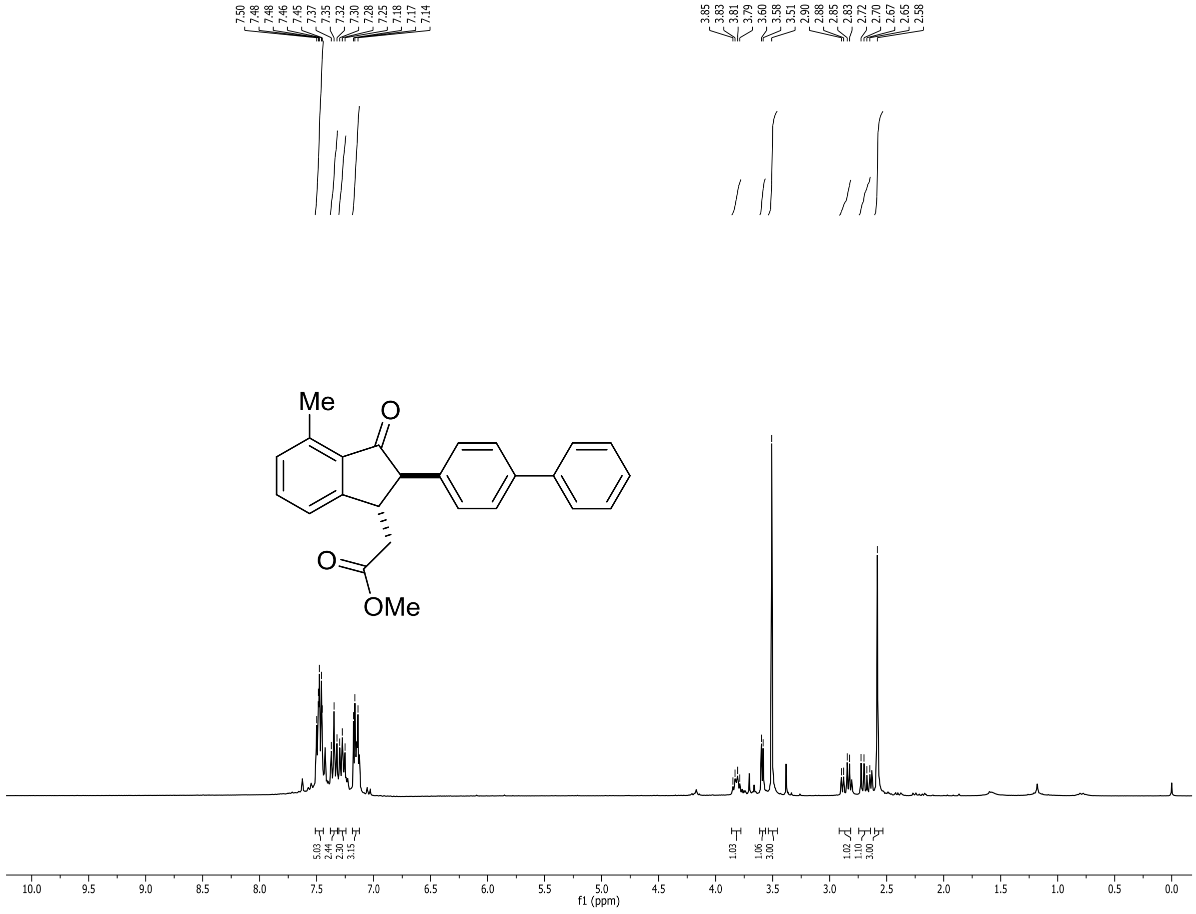

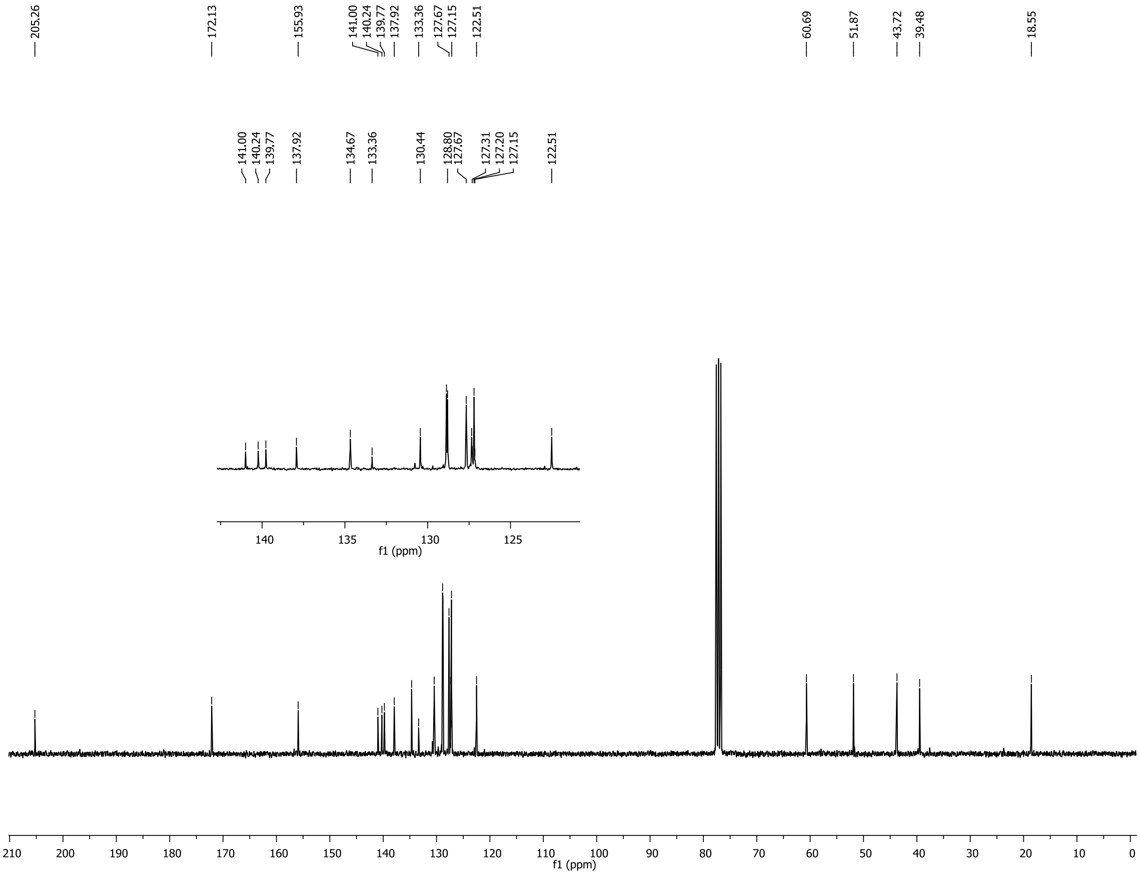

^{1}H and ^{13}C NMR data of compound **6t** (400, 101 MHz, $CDCl_3$)

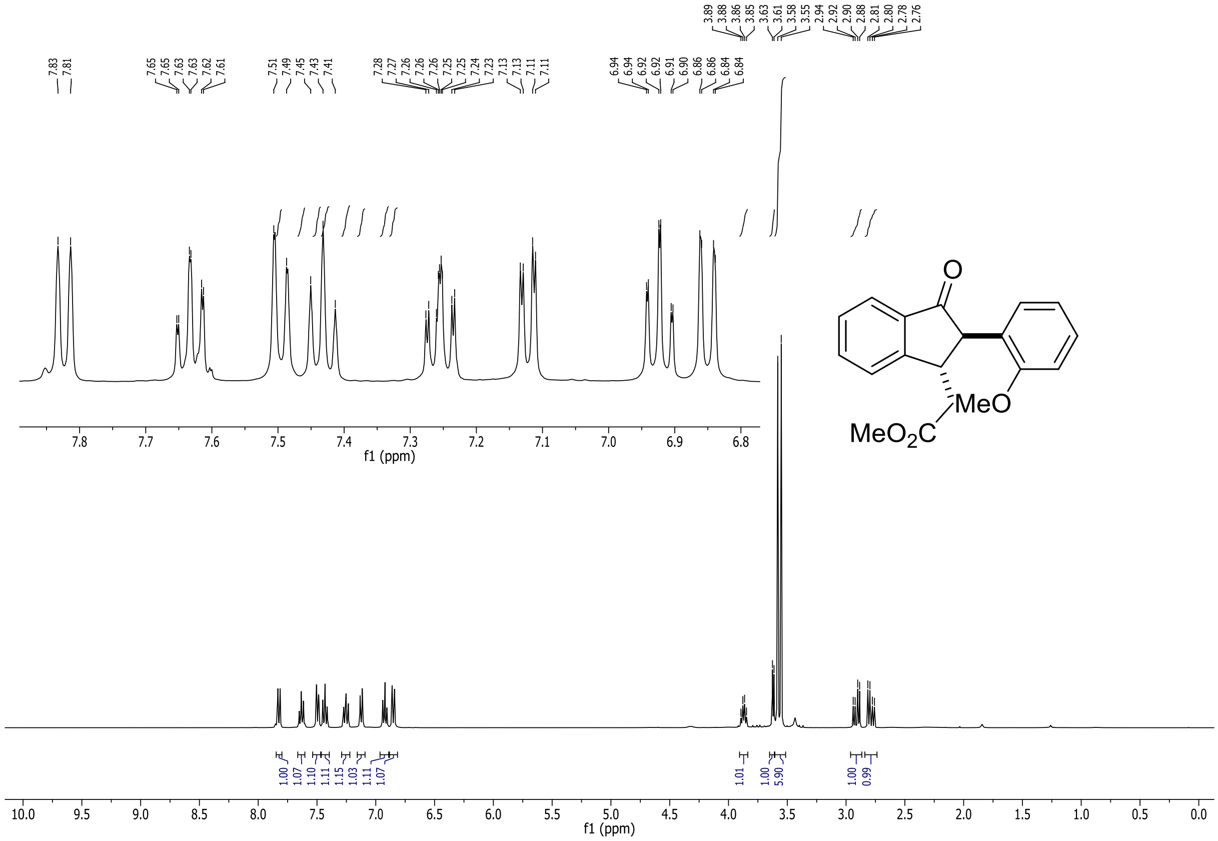

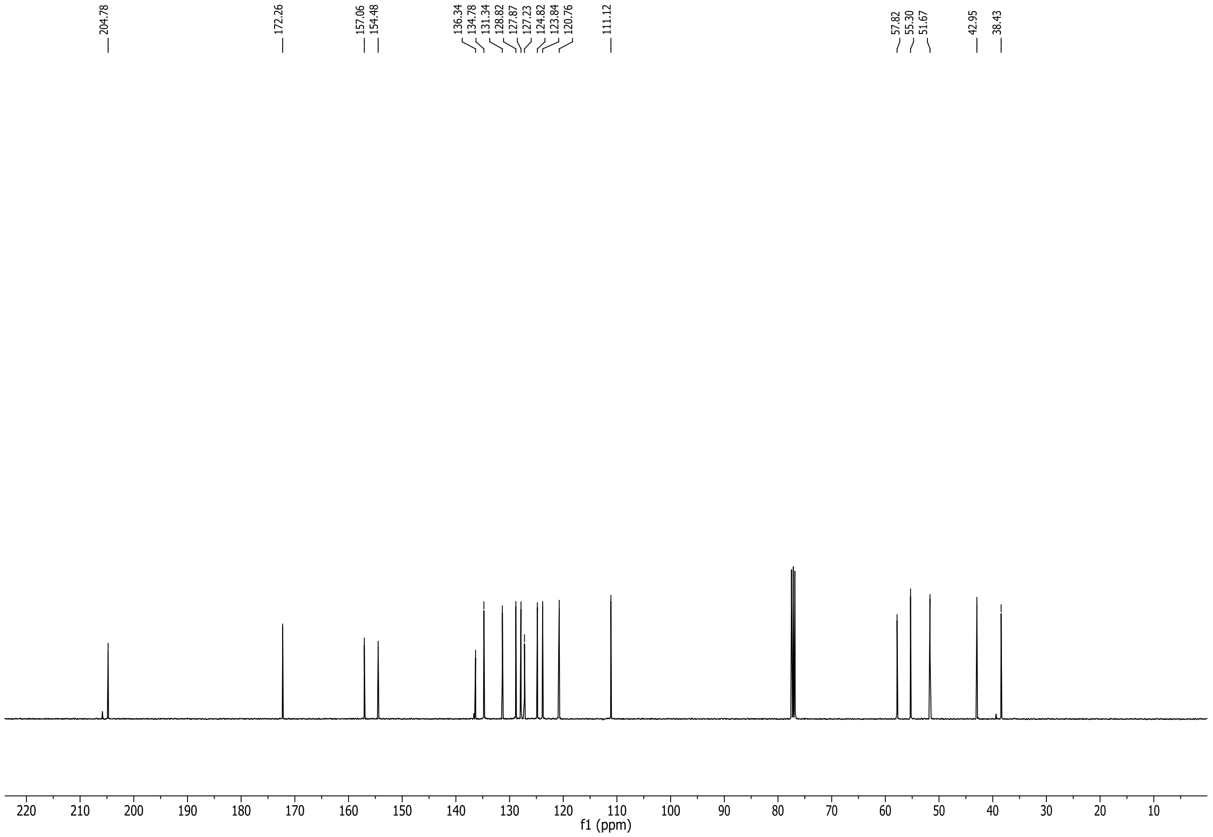

^{1}H and ^{13}C NMR data of compound **6u** (400, 101 MHz, $CDCl_3$)

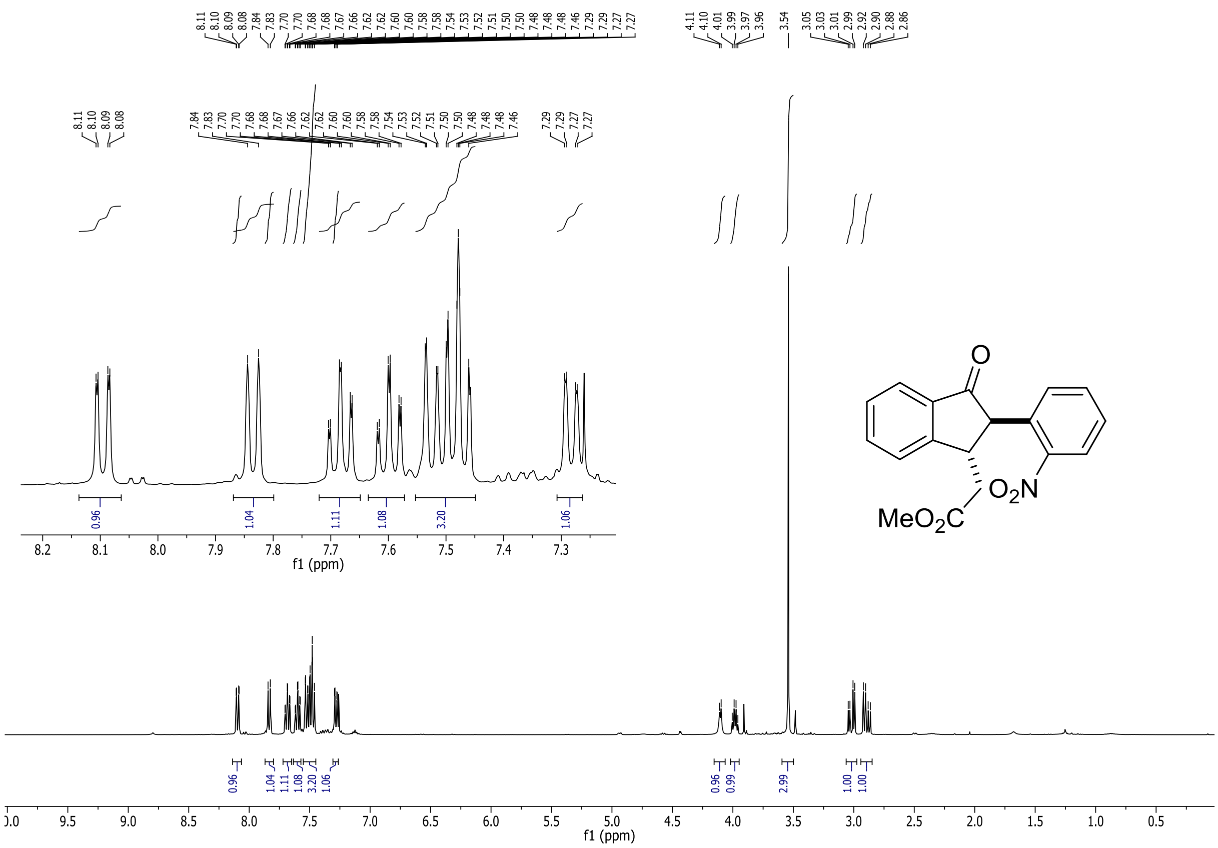
O
O2N
MeO2C
f1 (ppm)

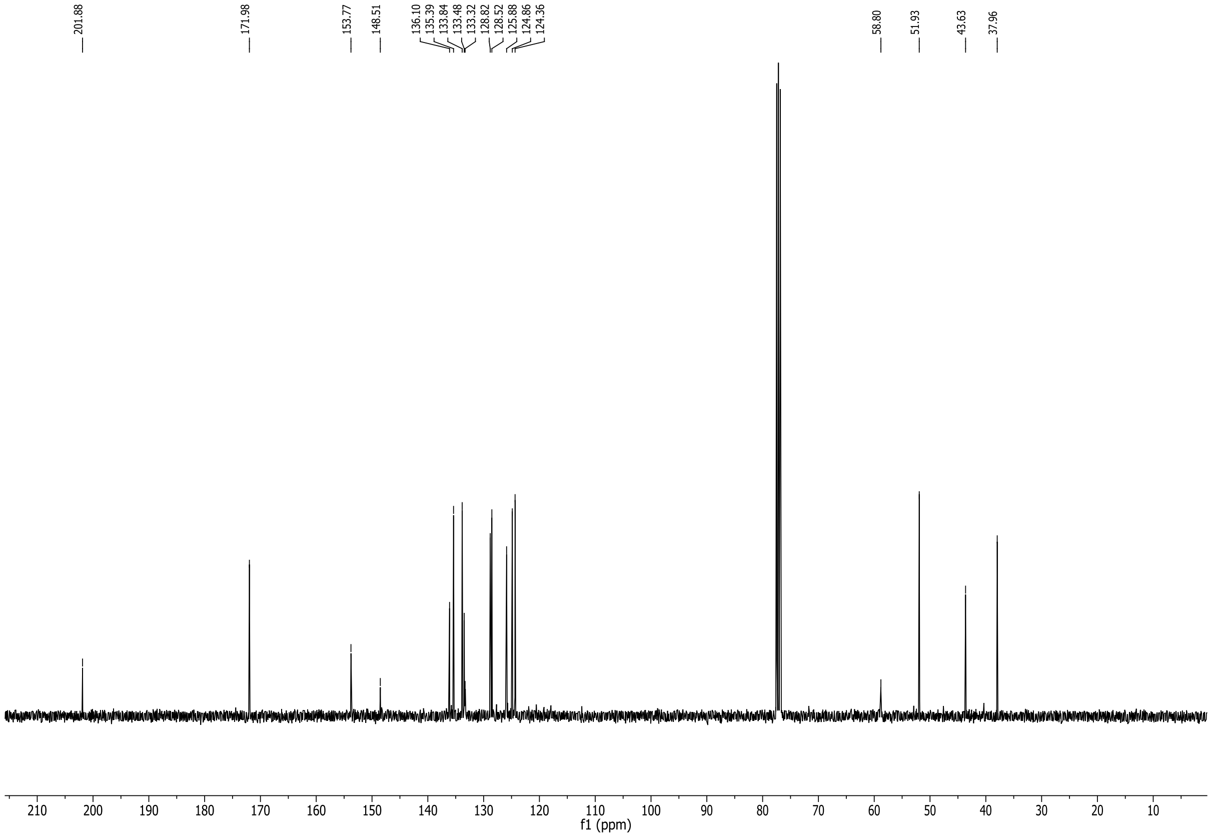
201.88
171.98
153.77
148.51
136.10
135.39
133.84
133.48
133.32
128.82
128.52
125.88
124.86
124.36
58.80
51.93
43.63
37.96
f1 (ppm)

^{1}H, ^{13}C and ^{19}F NMR data of compound **6v** (500, 125, 470 MHz, $CDCl_3$)

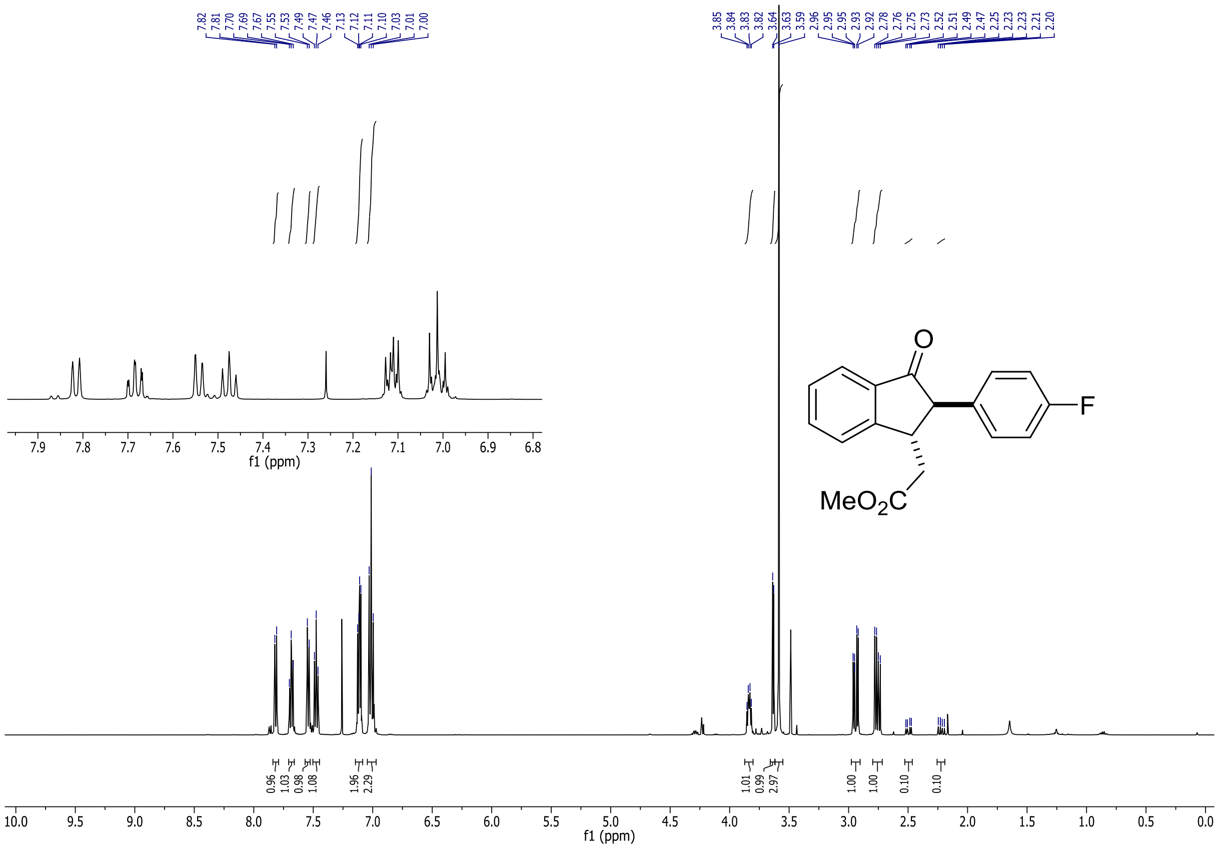

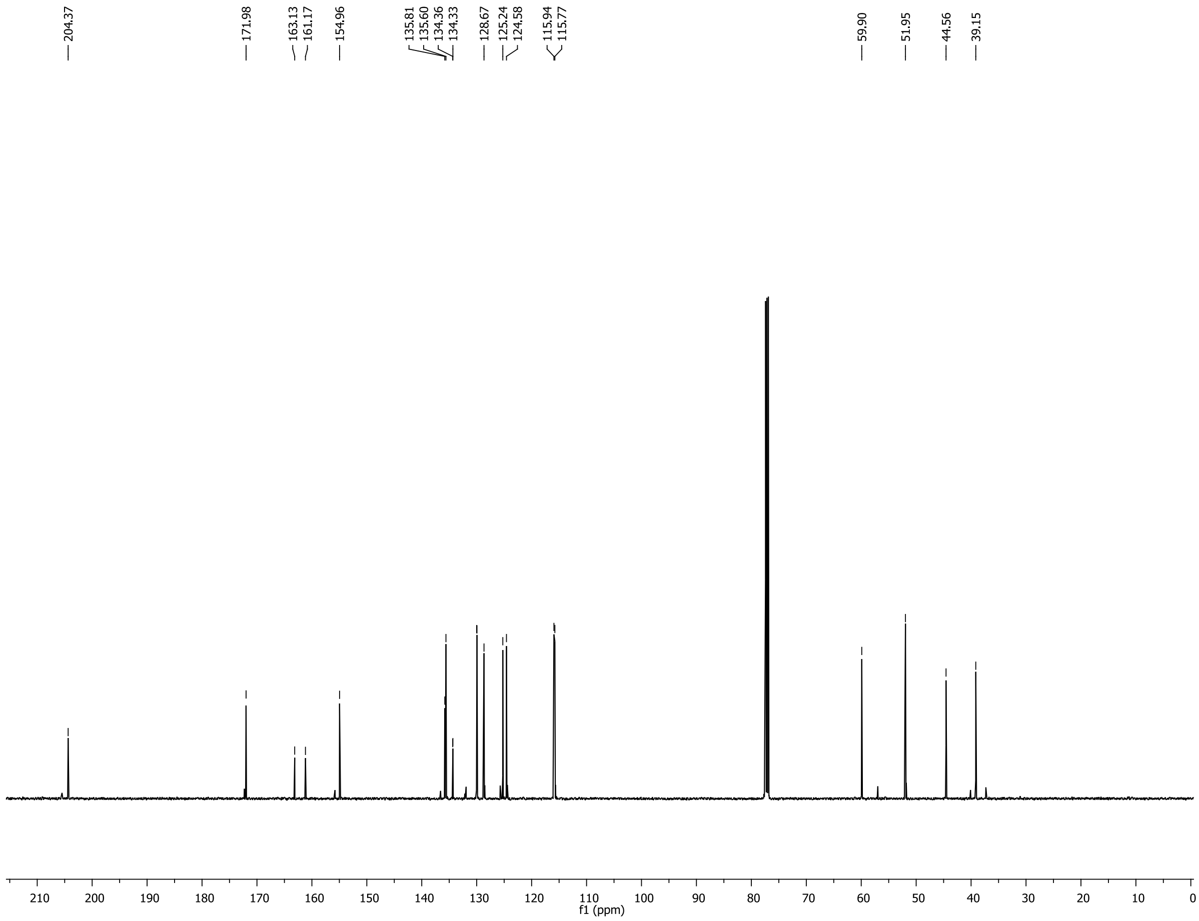

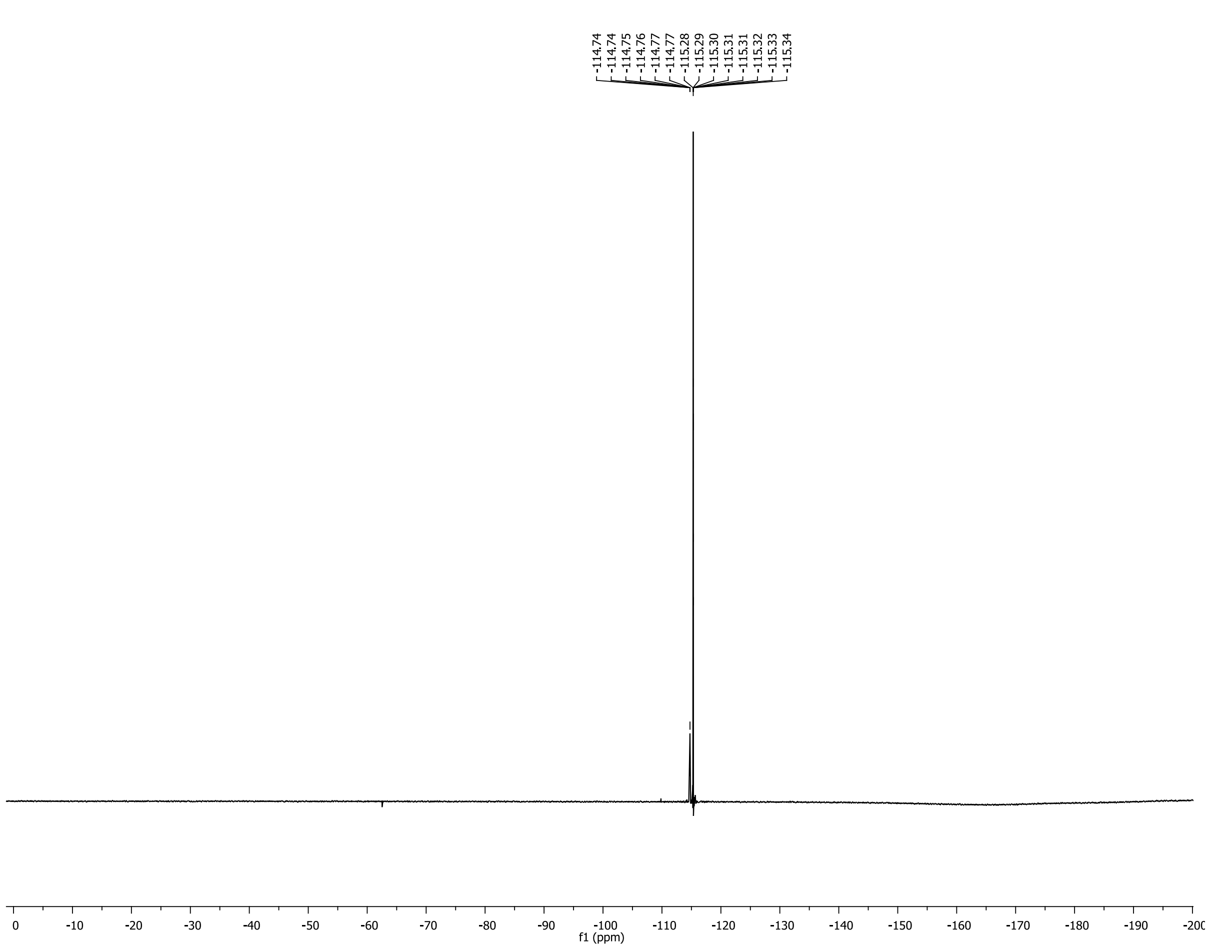

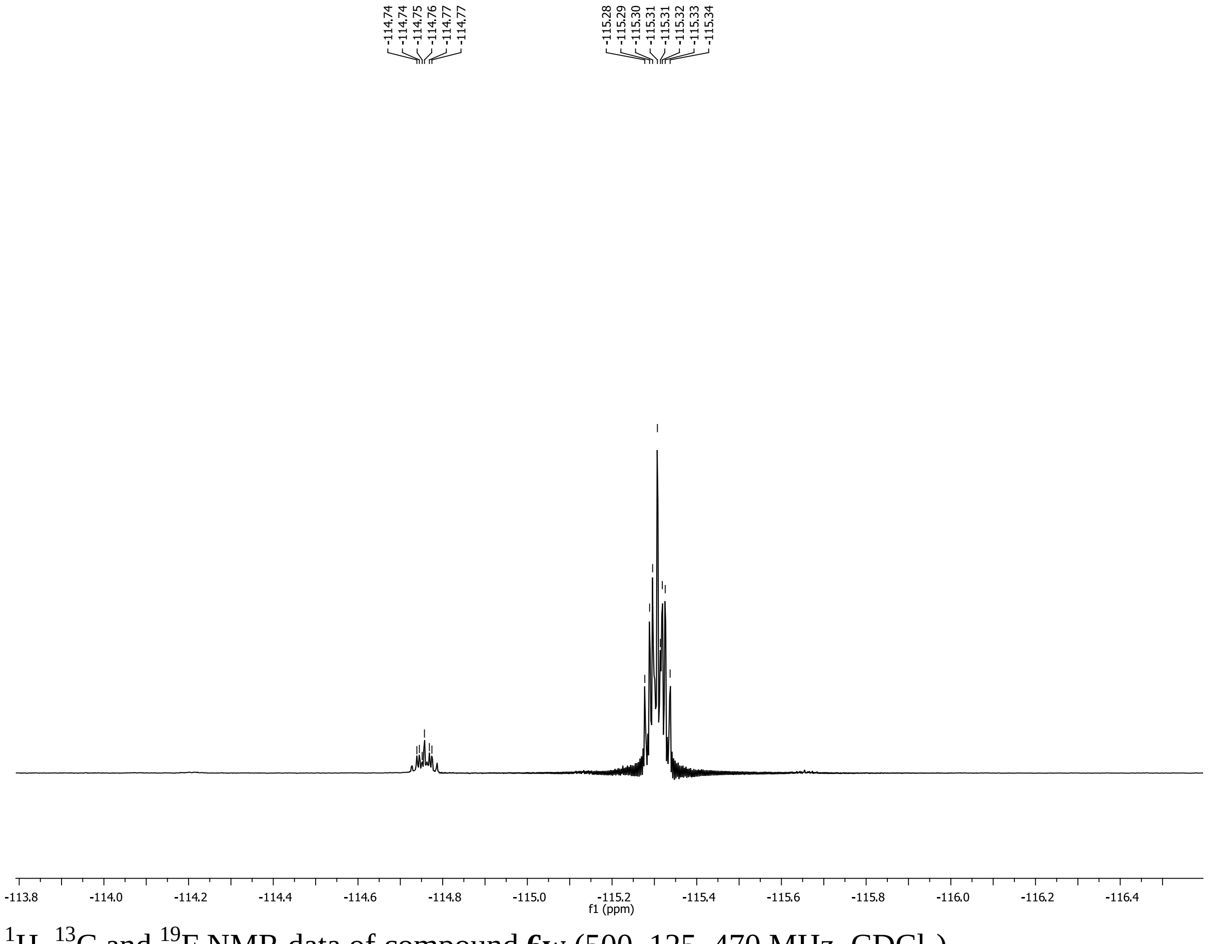

^{1}H, ^{13}C and ^{19}F NMR data of compound **6w** (500, 125, 470 MHz, $CDCl_3$)

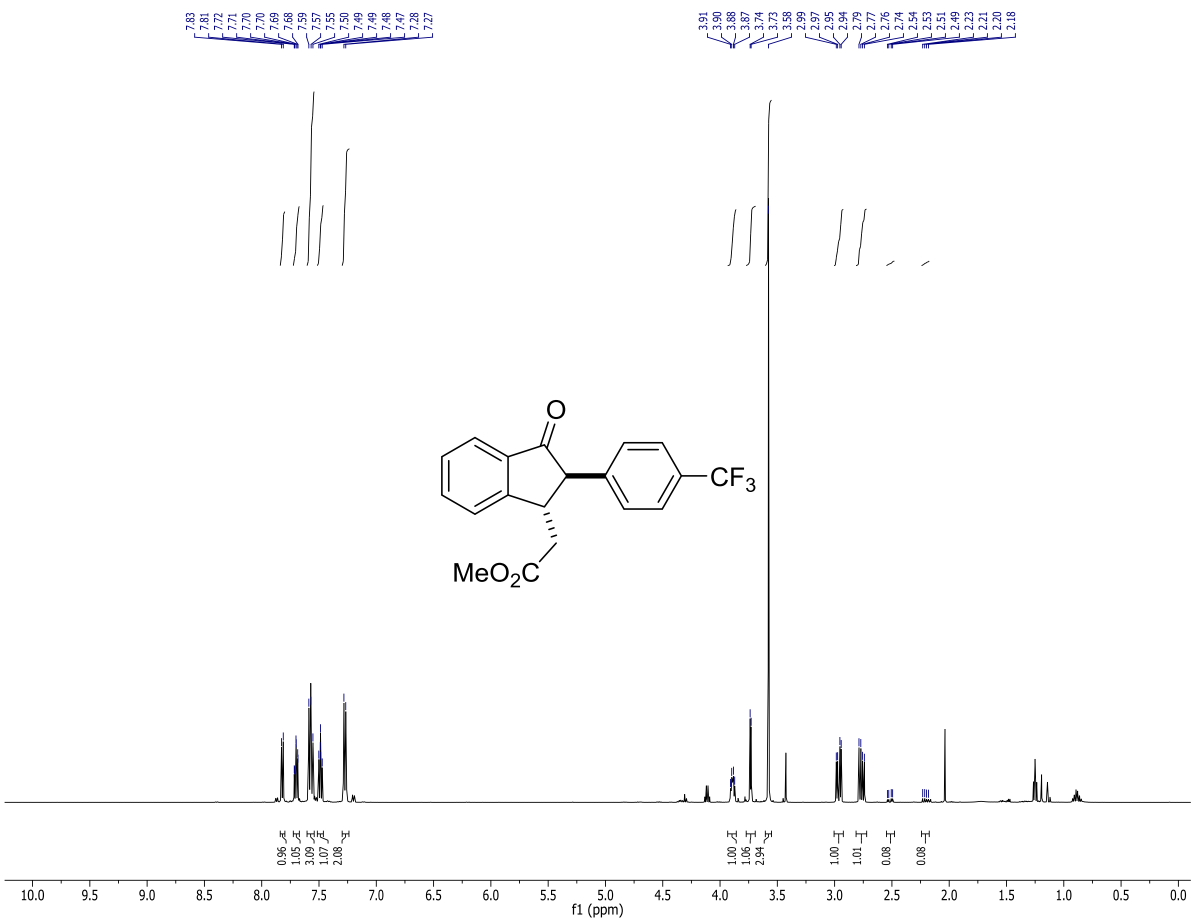
7.83
7.81
7.72
7.71
7.70
7.70
7.69
7.68
7.59
7.57
7.55
7.50
7.49
7.49
7.48
7.47
7.28
7.27
3.91
3.90
3.88
3.87
3.74
3.73
3.58
2.99
2.97
2.95
2.94
2.79
2.77
2.76
2.74
2.54
2.53
2.51
2.49
2.23
2.21
2.20
2.18
O
CF3
MeO2C
0.96
1.05
3.09
1.07
2.08
1.00
1.06
2.94
1.00
1.01
0.08
0.08
10.0 9.5 9.0 8.5 8.0 7.5 7.0 6.5 6.0 5.5 5.0 4.5 4.0 3.5 3.0 2.5 2.0 1.5 1.0 0.5 0.0
f1 (ppm)

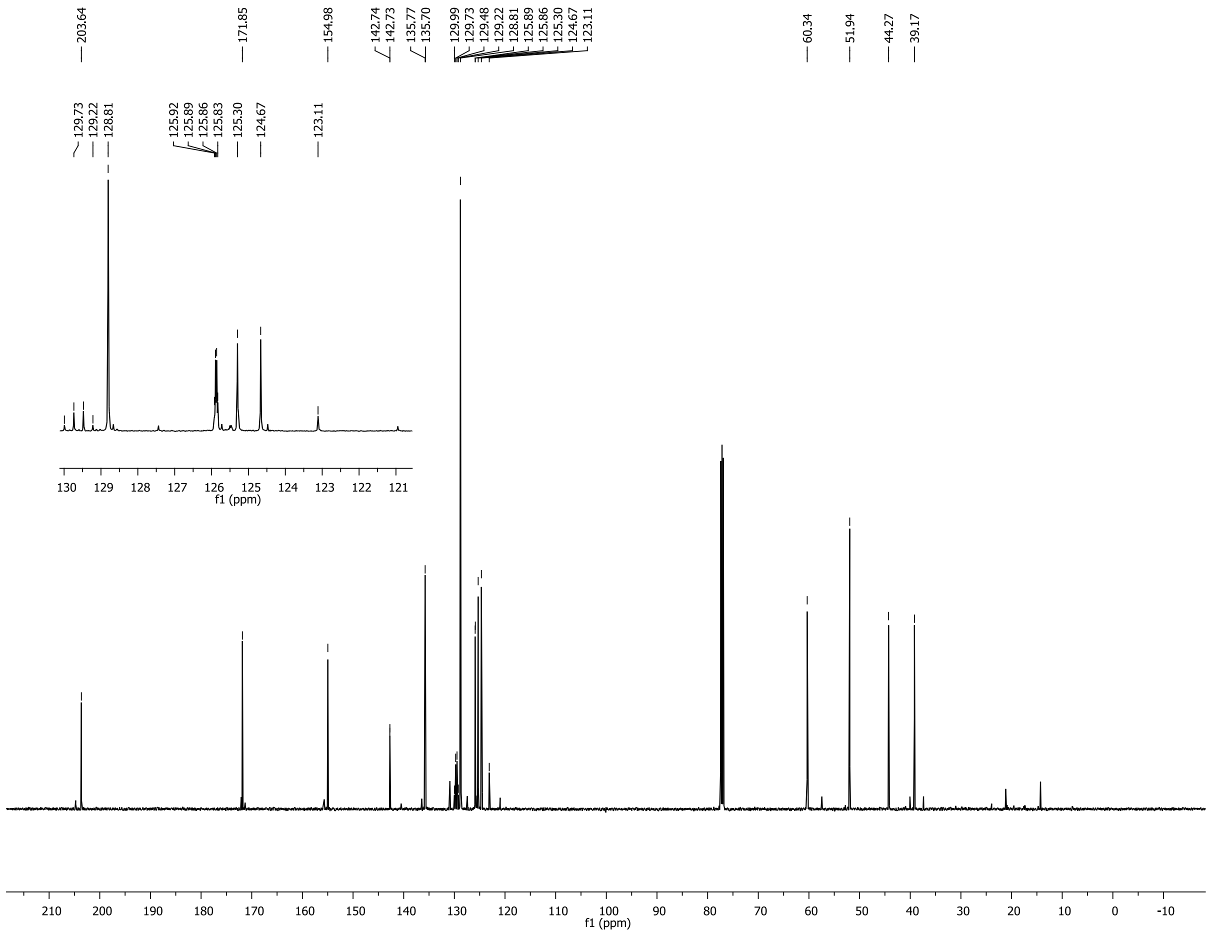
203.64
171.85
154.98
142.74
142.73
135.77
135.70
129.99
129.73
129.48
129.22
128.81
125.89
125.86
125.30
124.67
123.11
60.34
51.94
44.27
39.17
129.73
129.22
128.81
125.92
125.89
125.86
125.83
125.30
124.67
123.11
130 129 128 127 126 125 124 123 122 121
f1 (ppm)
210 200 190 180 170 160 150 140 130 120 110 100 90 80 70 60 50 40 30 20 10 0 -10
f1 (ppm)

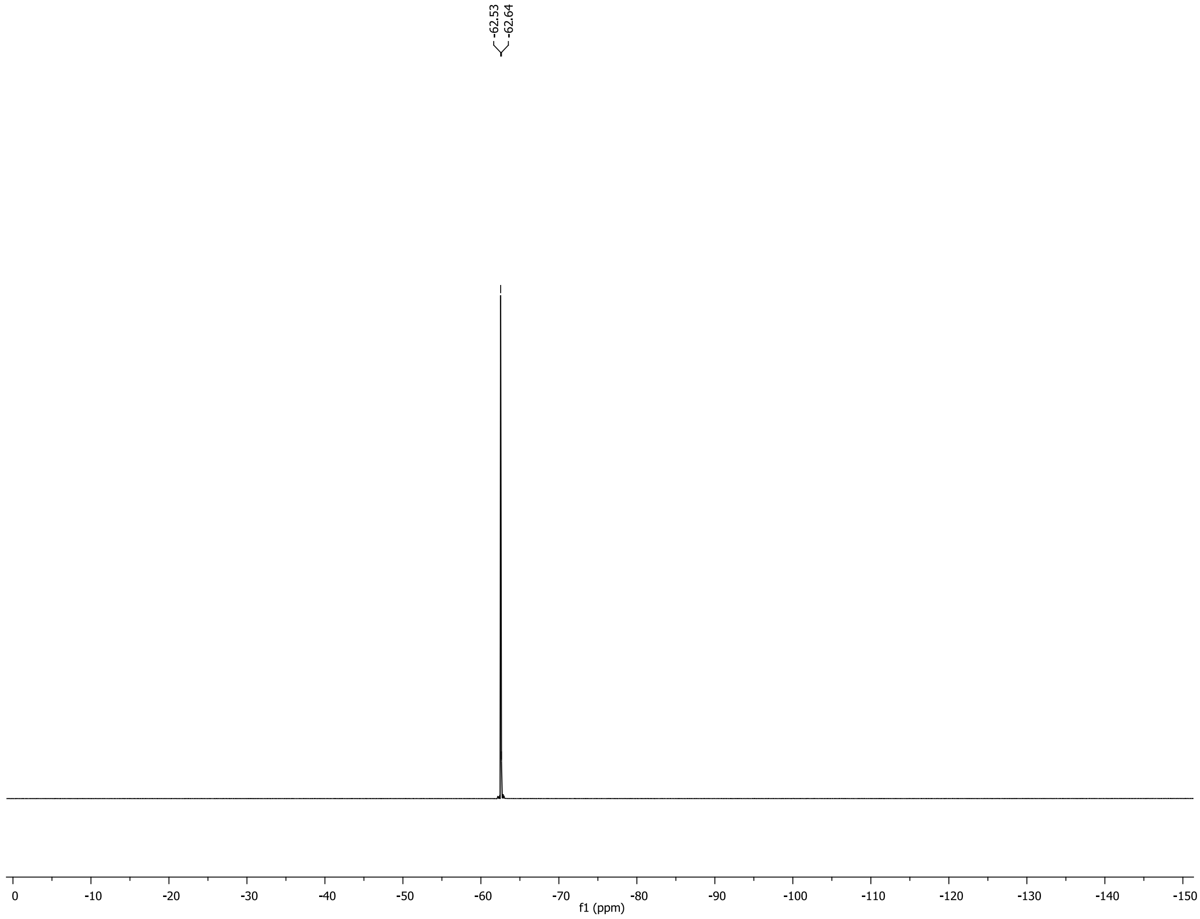

^{1}H and ^{13}C NMR data of compound **6x** (400, 101 MHz, $CDCl_3$)

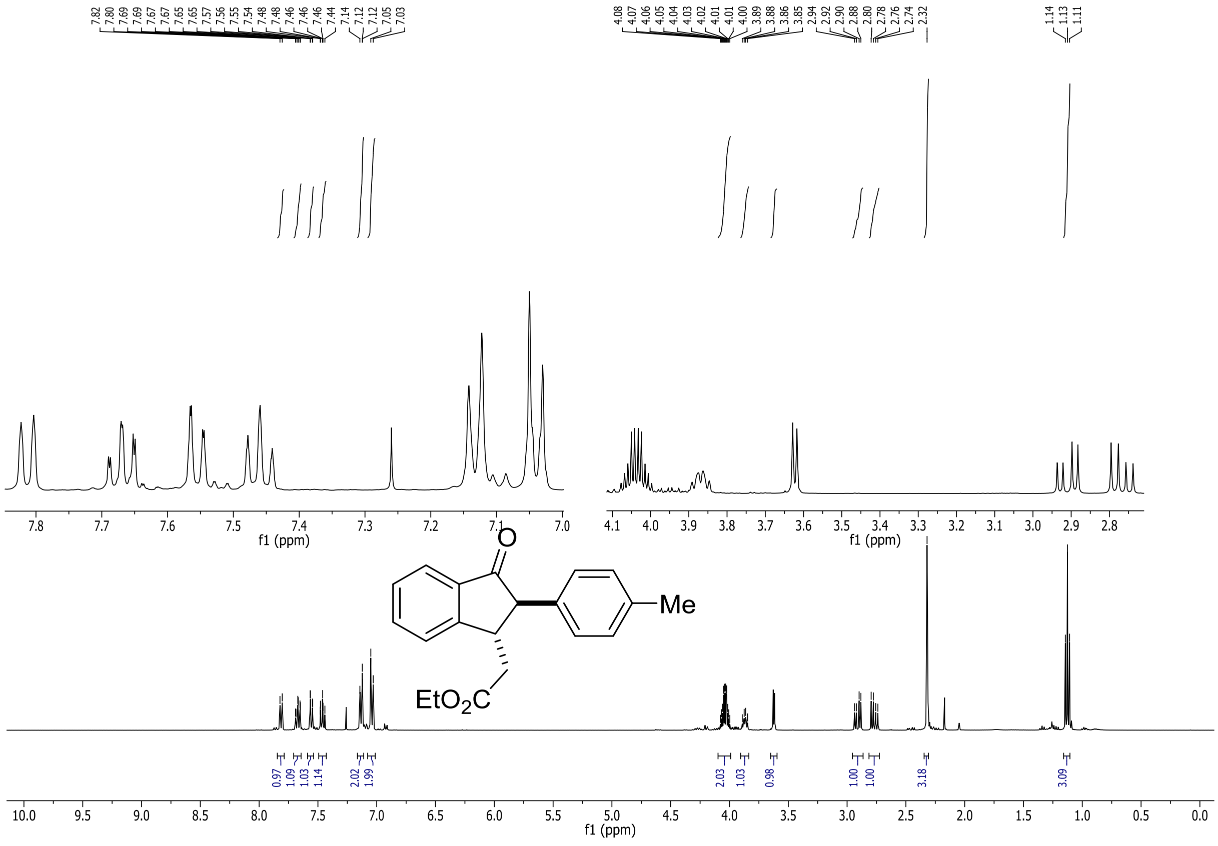

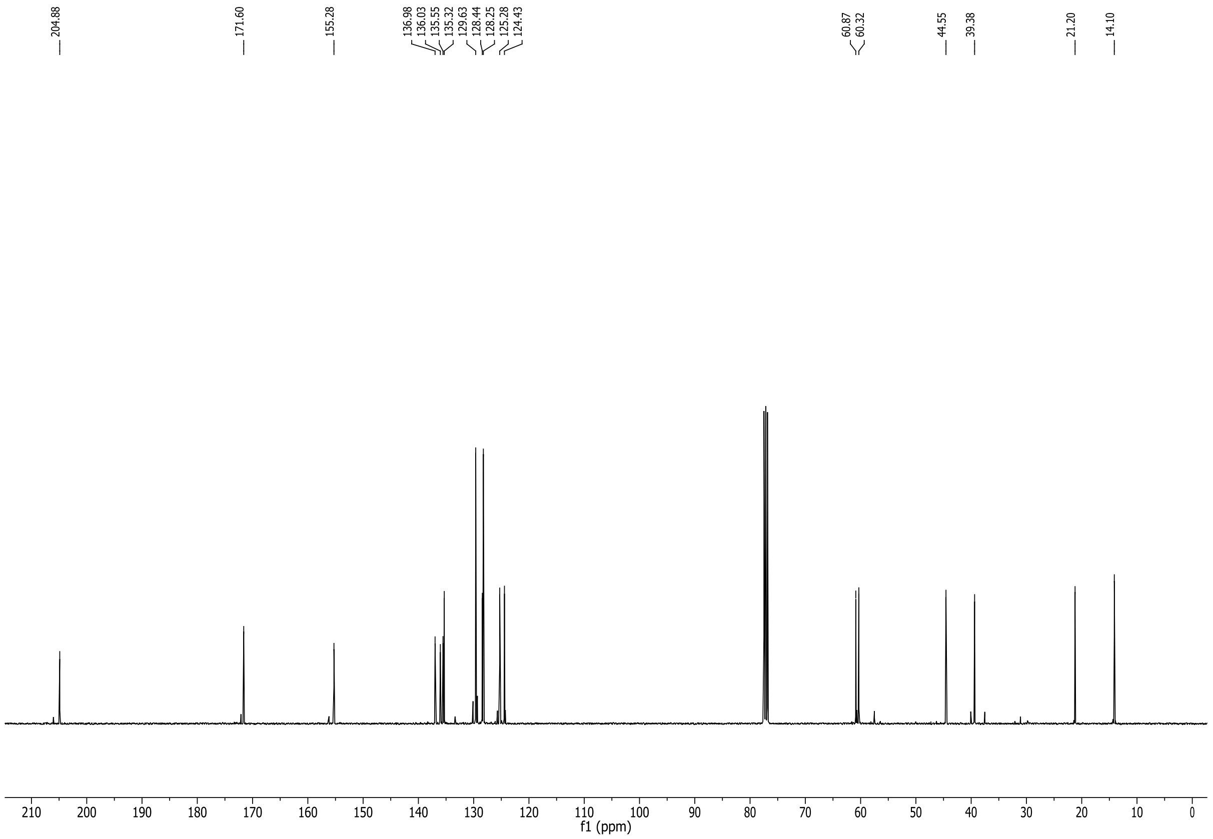
204.88
171.60
155.28
136.98
136.03
135.55
135.32
129.63
128.44
128.25
125.28
124.43
60.87
60.32
44.55
39.38
21.20
14.10
210 200 190 180 170 160 150 140 130 120 110 100 90 80 70 60 50 40 30 20 10 0
f1 (ppm)

^{1}H and ^{13}C NMR data of compound **6y** (500, 125 MHz, $CDCl_3$)

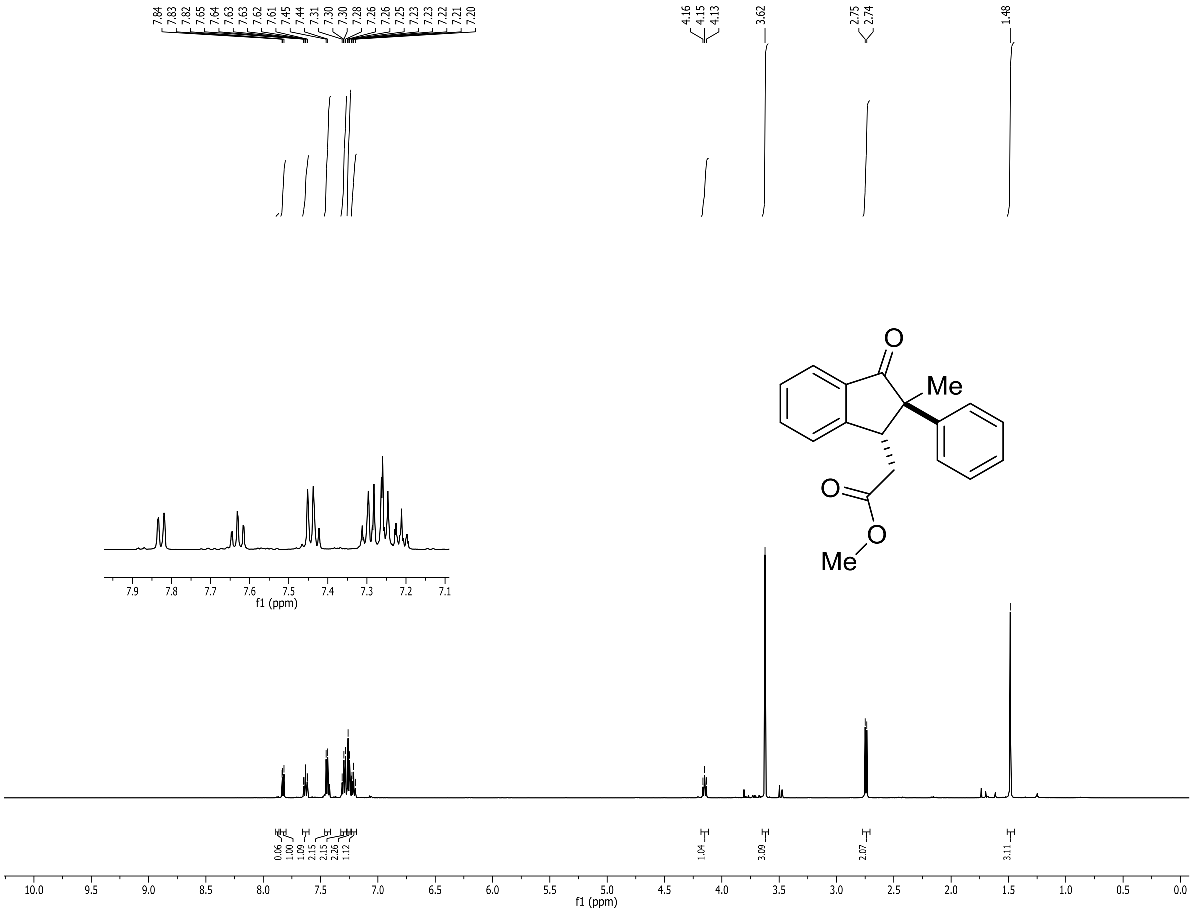

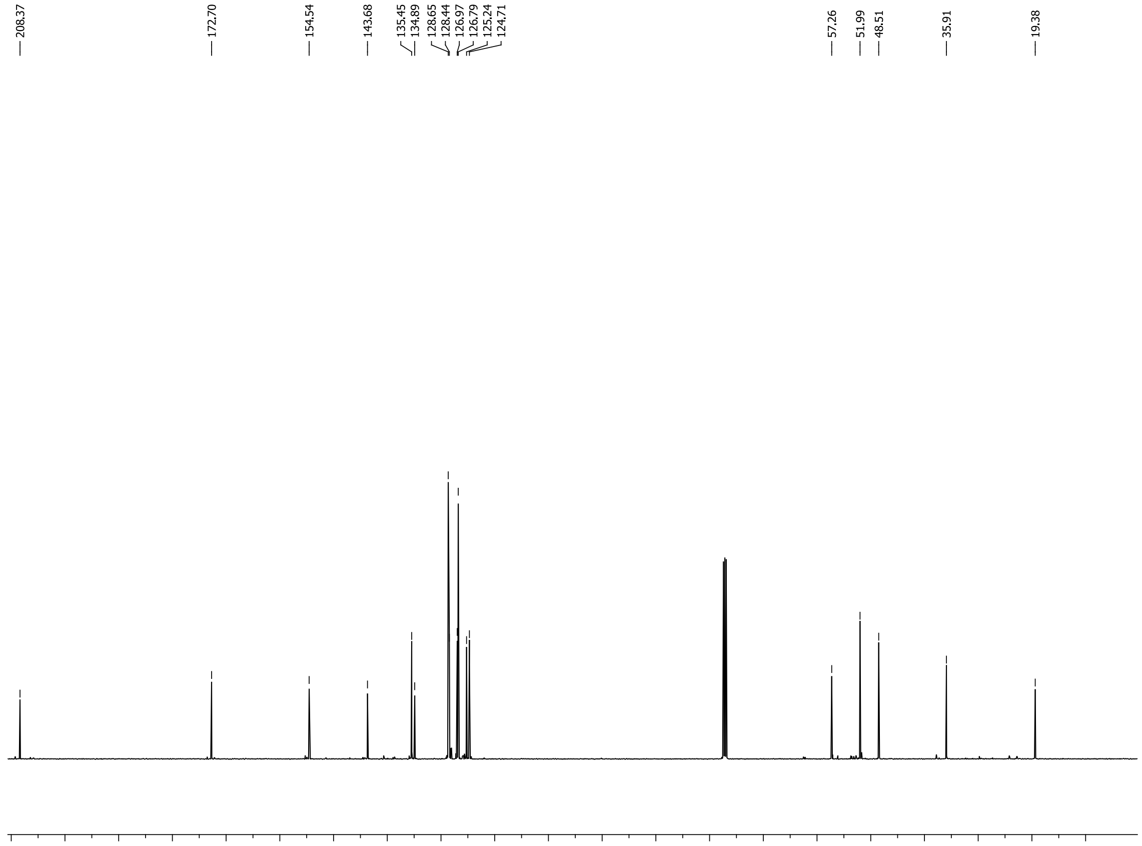

^{1}H and ^{13}C NMR data of compound **7y** (400, 101 MHz, $CDCl_3$)

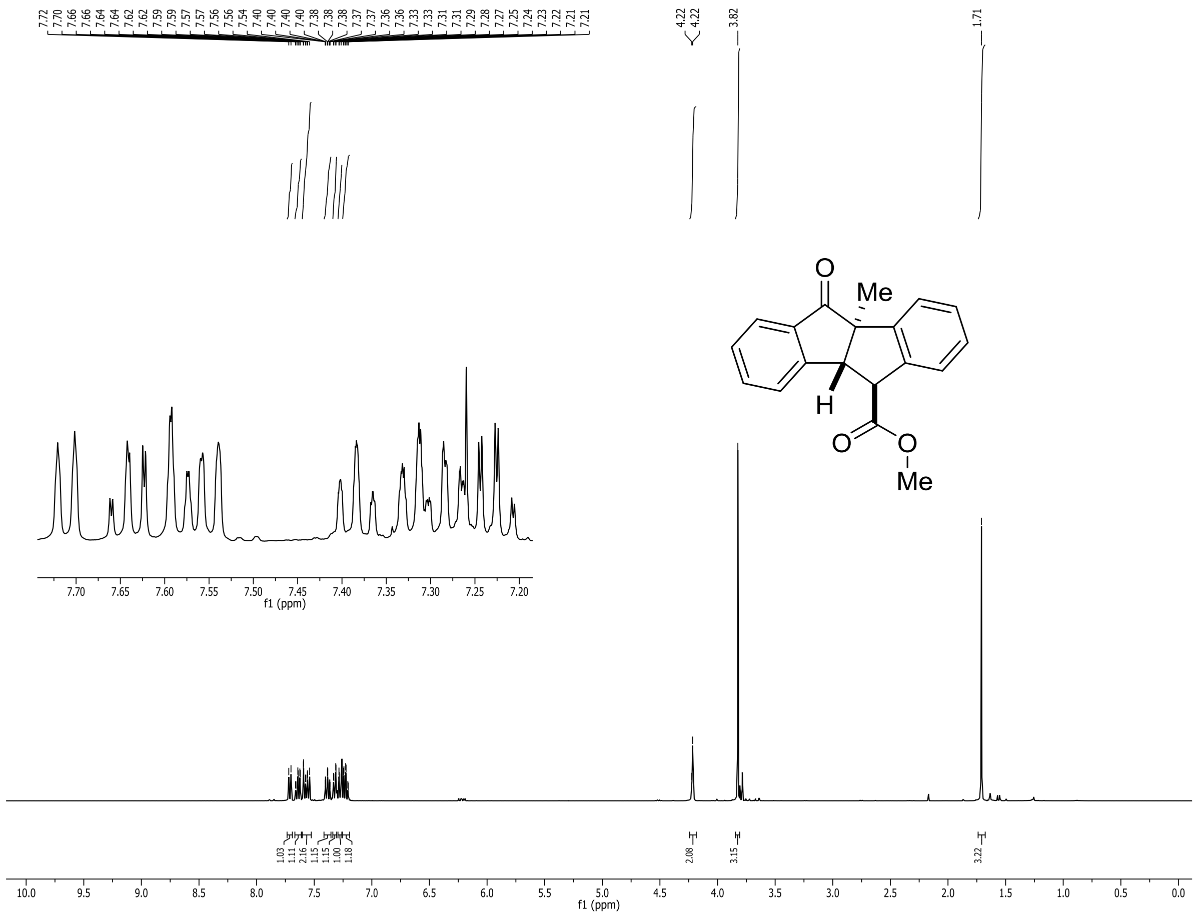

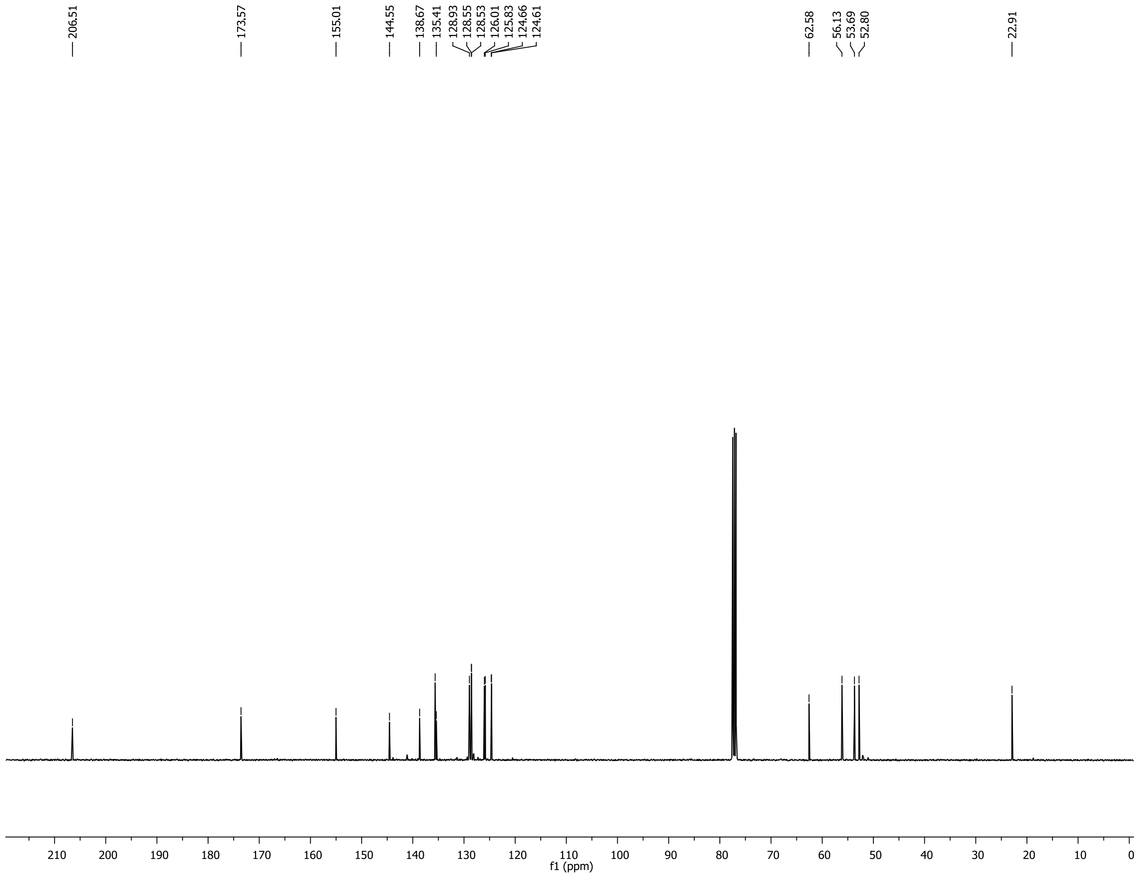

^{1}H and ^{13}C NMR data of compound **6z** (500, 125 MHz, $CDCl_3$)

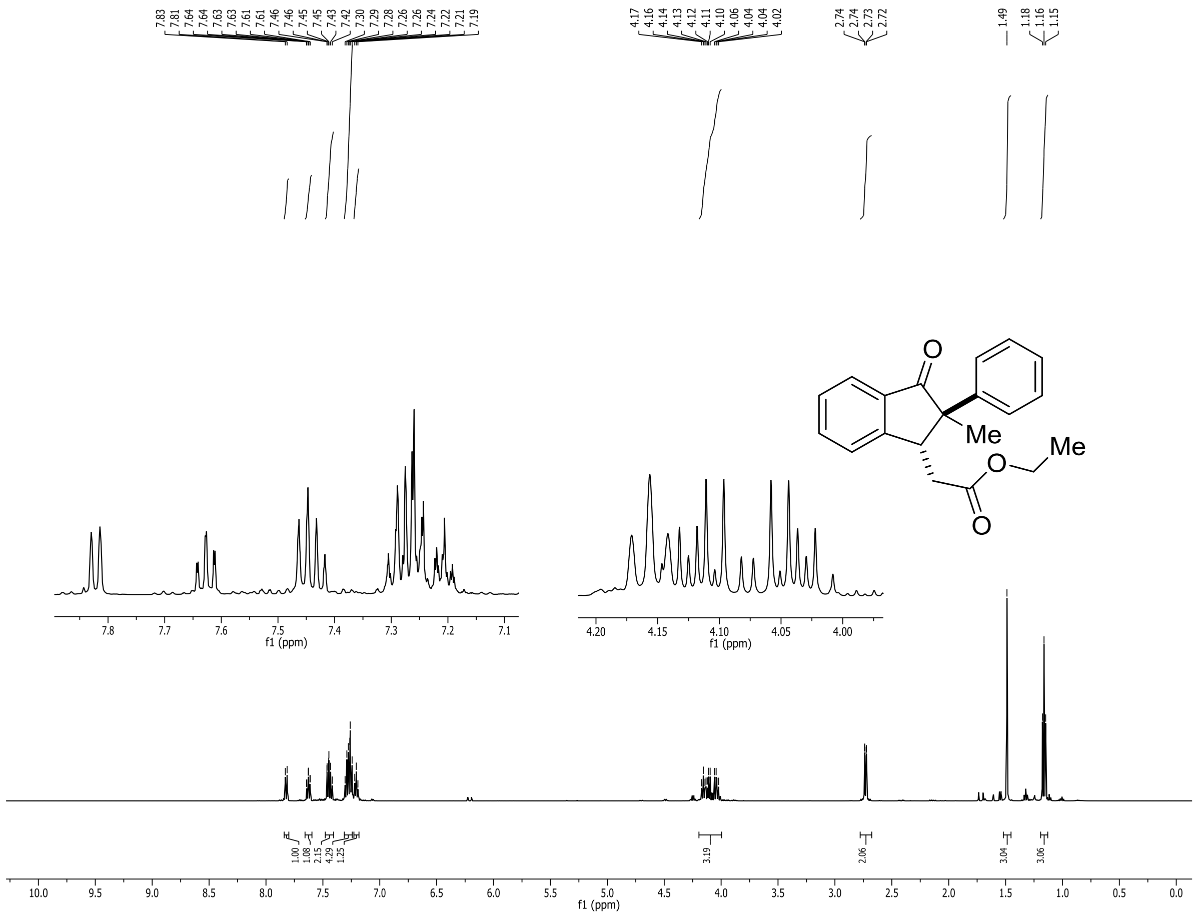

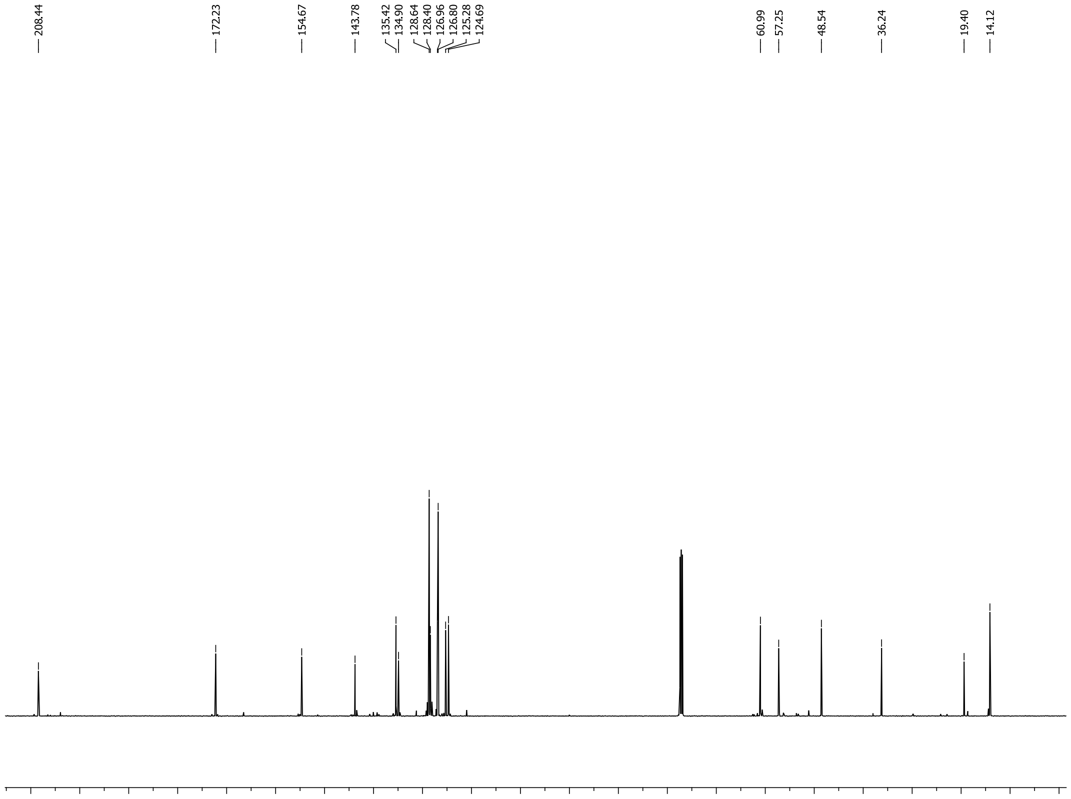

^{1}H and ^{13}C NMR data of compound **7z** (500, 125 MHz, $CDCl_3$)

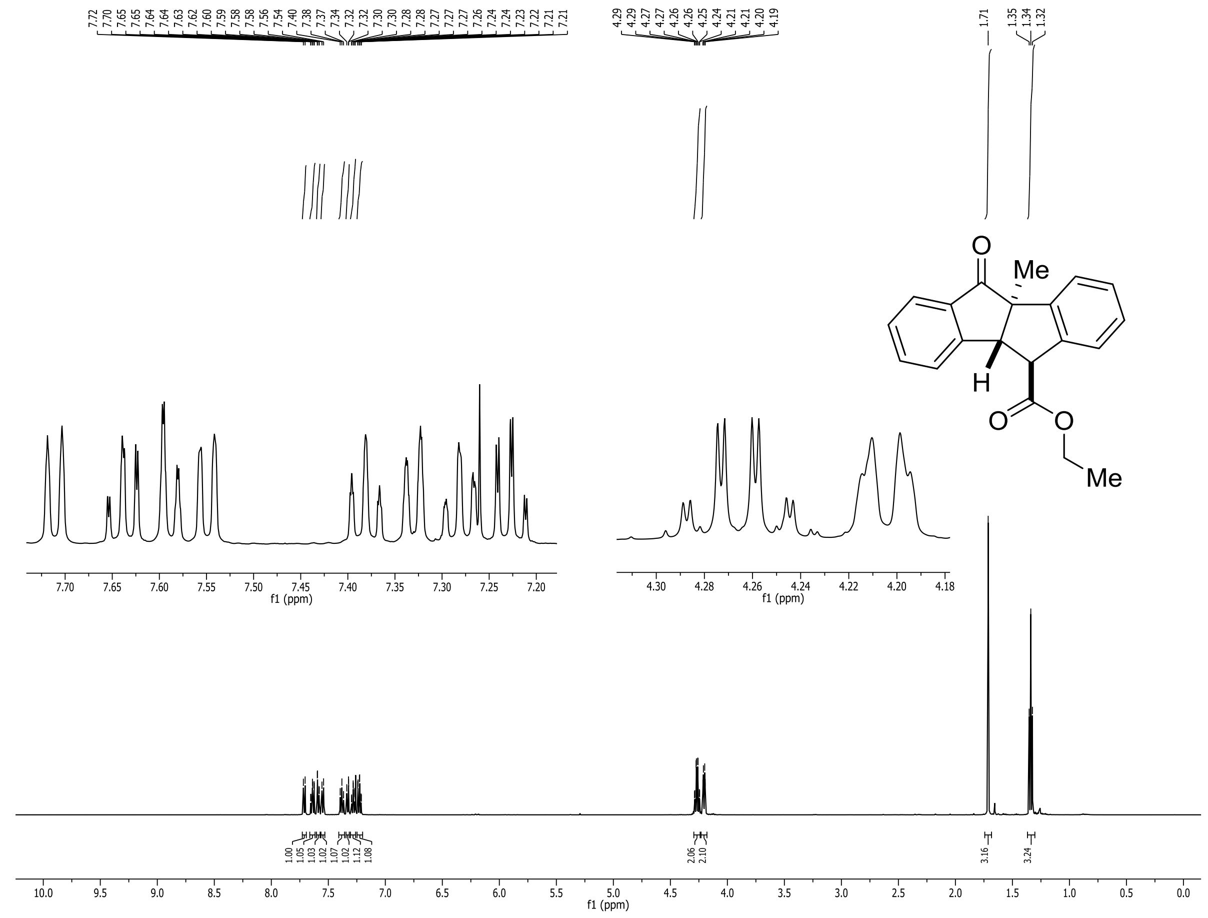

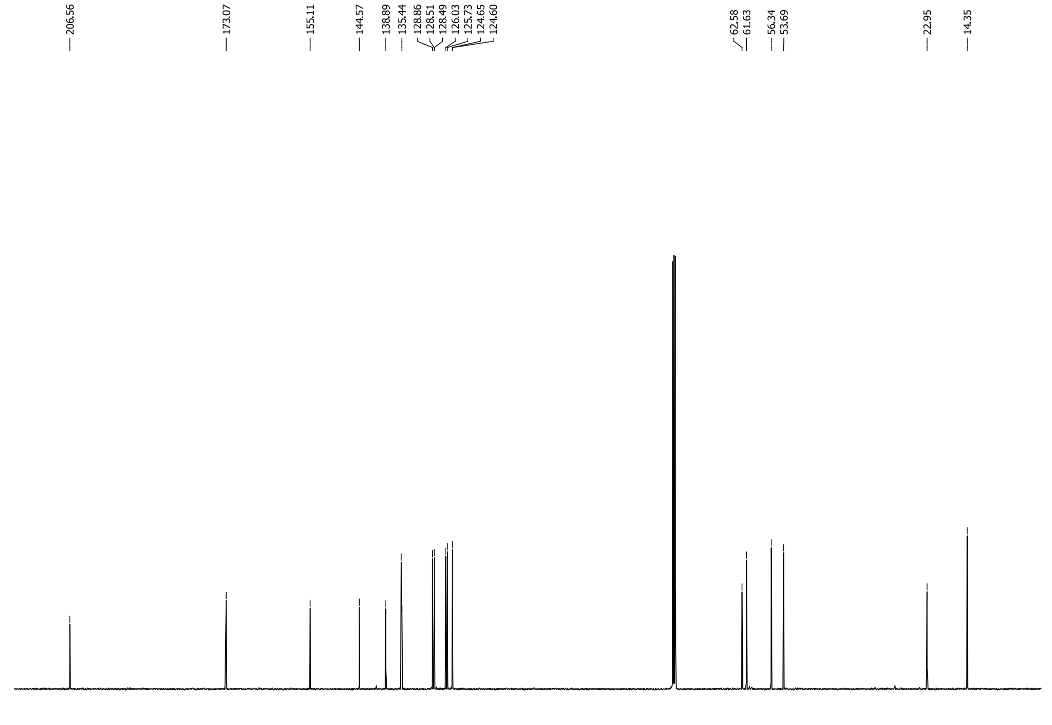

^{1}H and ^{13}C NMR data of compound **6ab** (400, 101 MHz, $CDCl_3$)

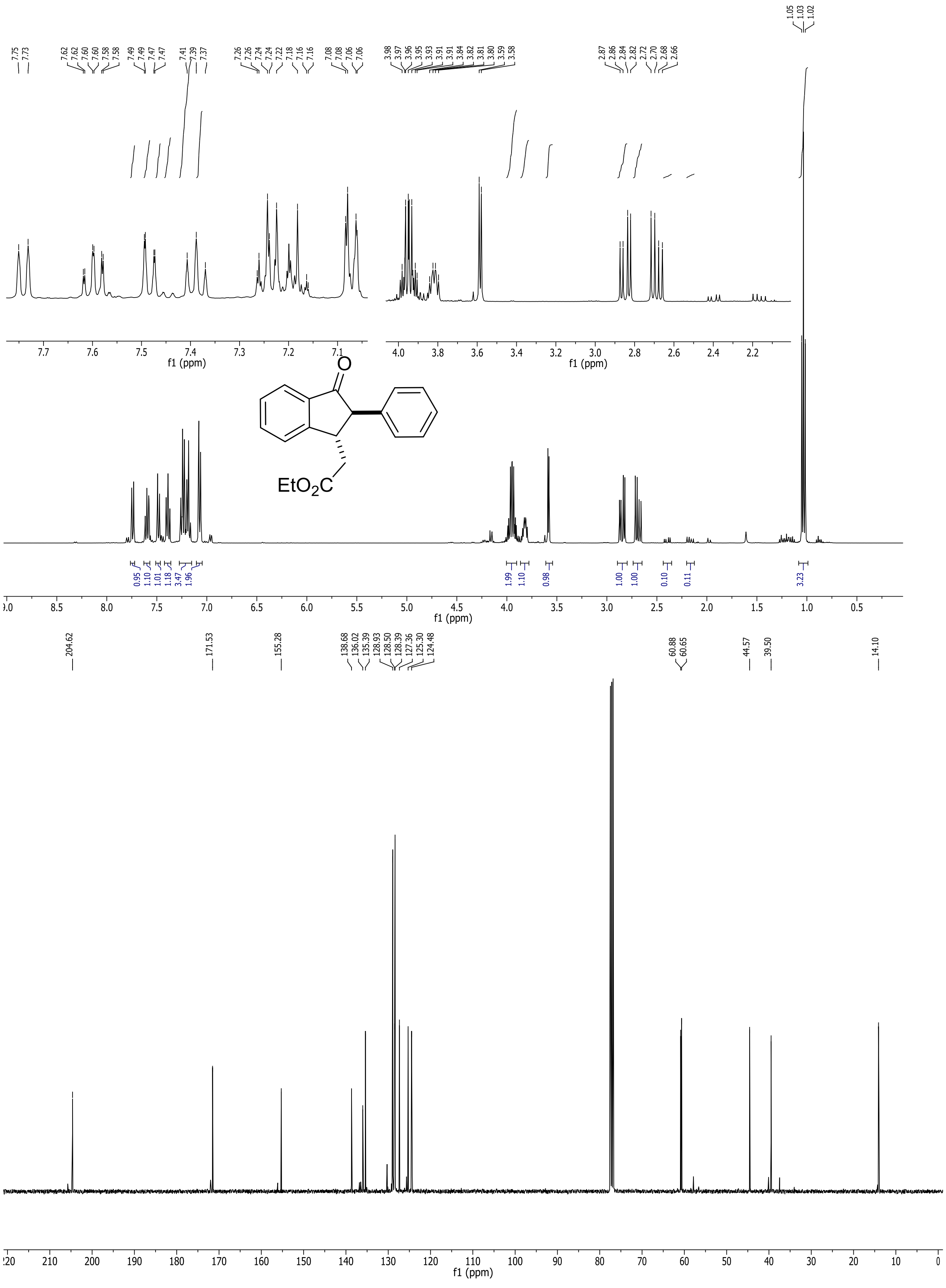

^{1}H and ^{13}C NMR data of compound **7ab** (300, 75 MHz, $CDCl_3$)

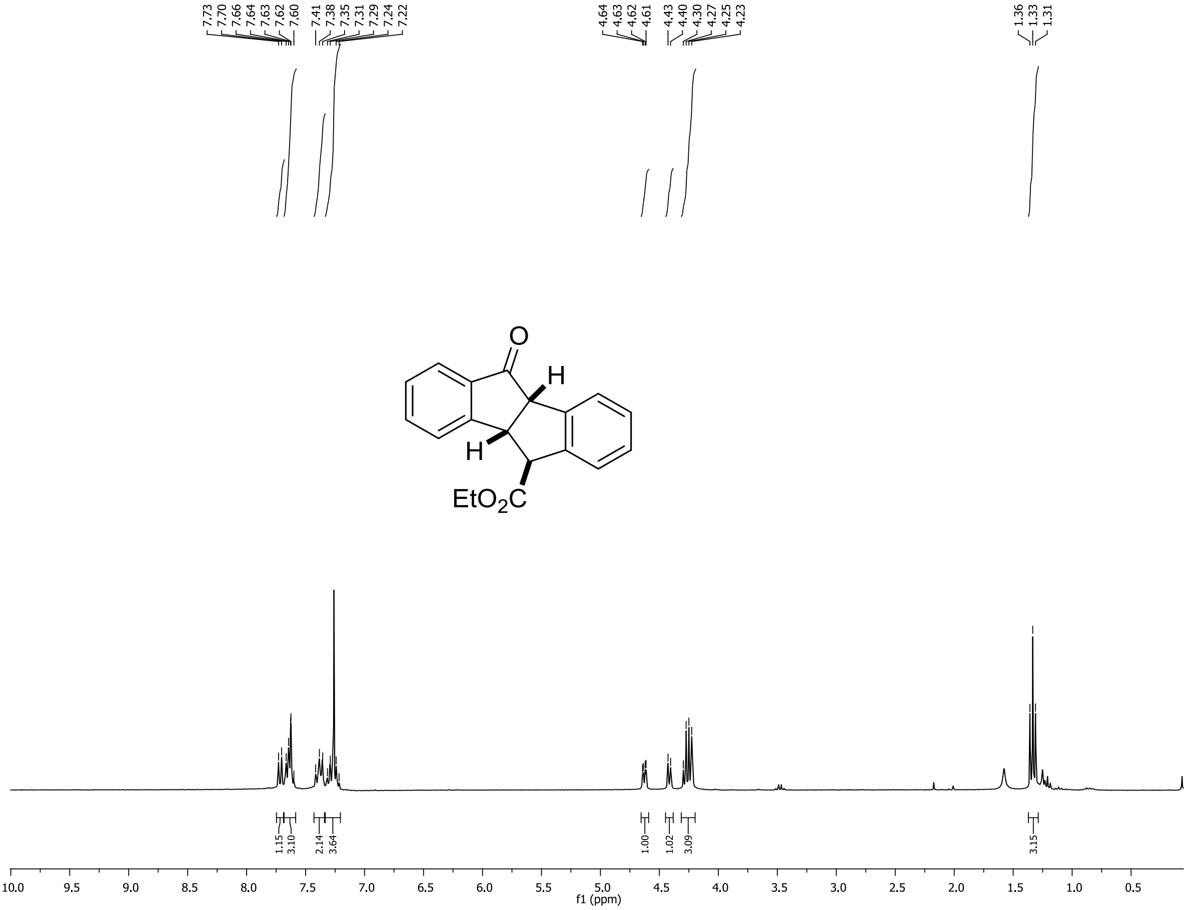

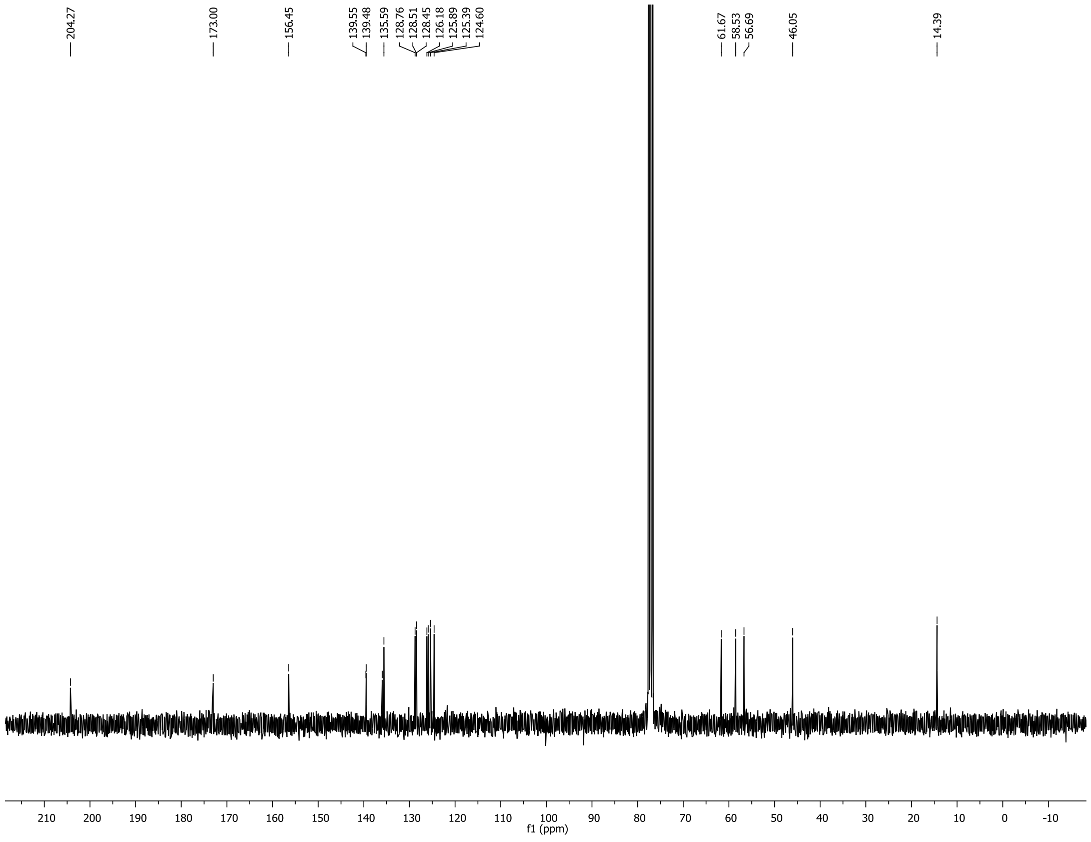

^{1}H and ^{13}C NMR data of compound **6ac** (400, 101 MHz, $CDCl_3$)

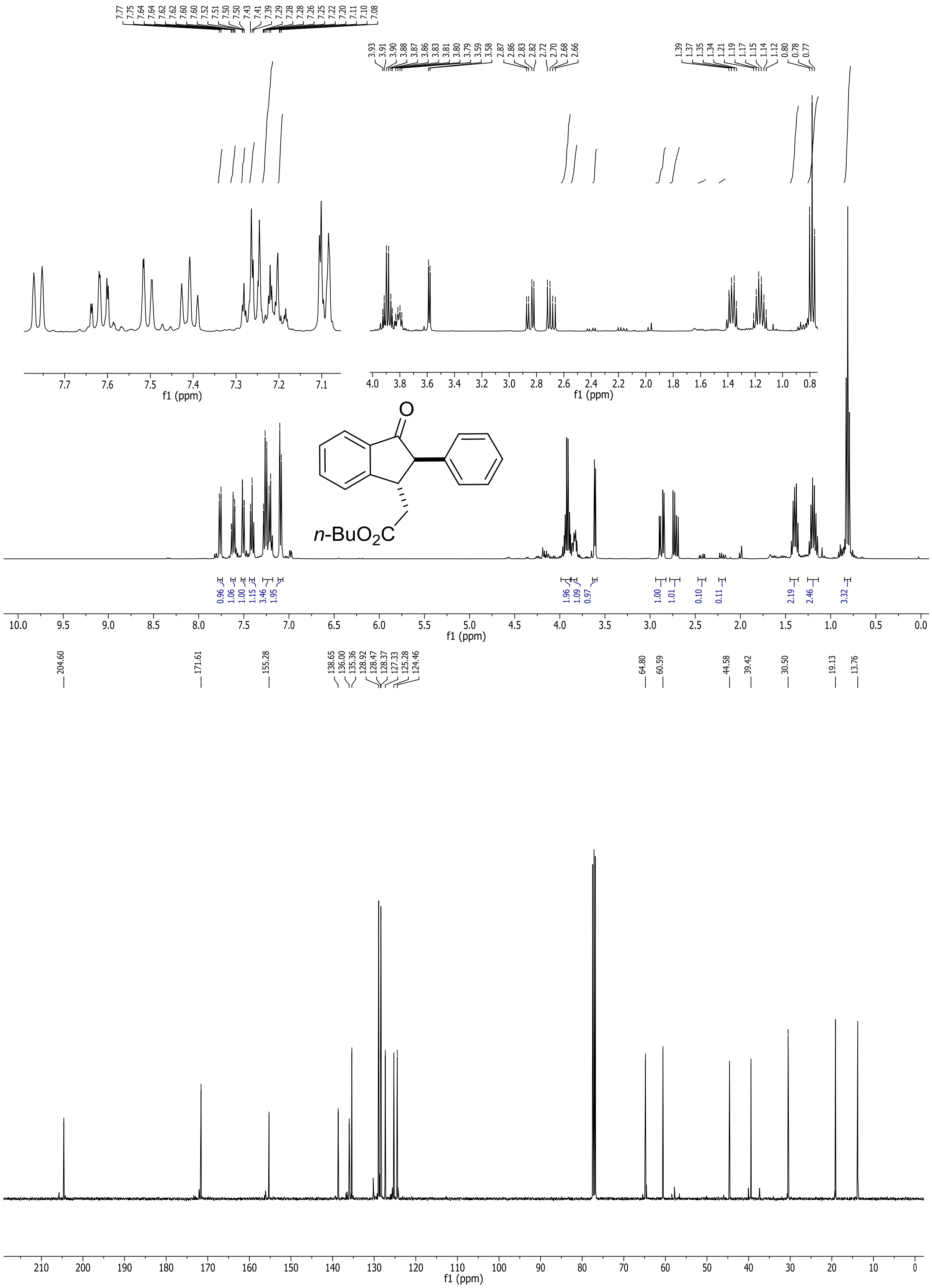

^{1}H and ^{13}C NMR data of compound **6ad** (500, 125 MHz, $CDCl_3$)

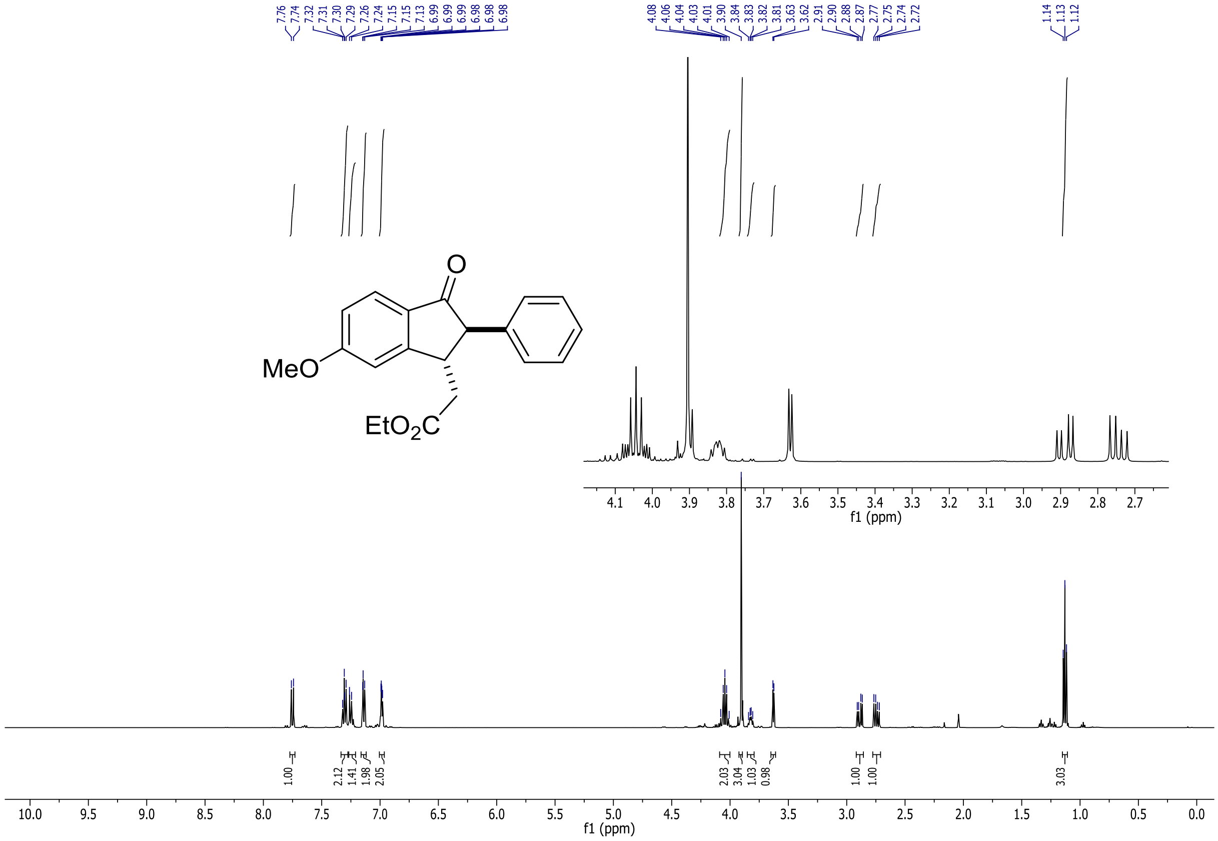

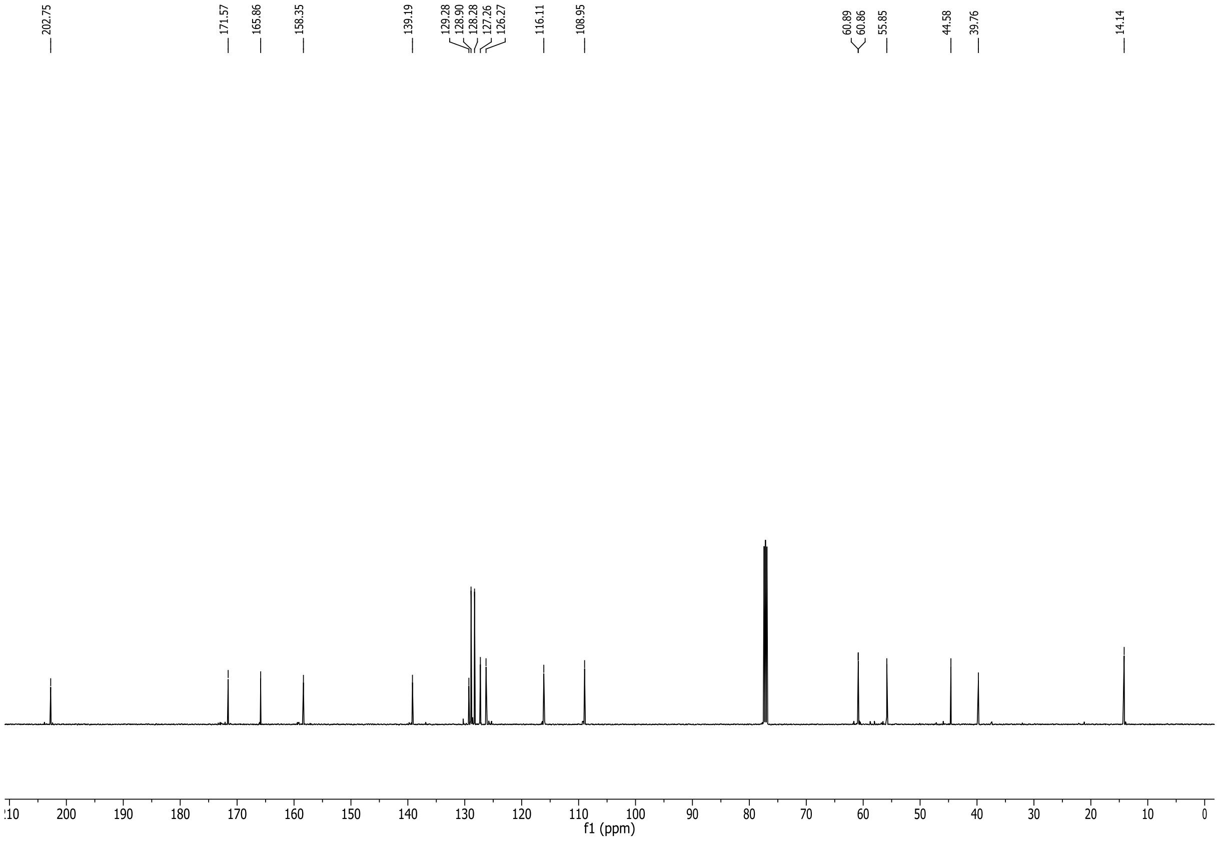

^{1}H and ^{13}C NMR data of compound **6ae** (400, 101 MHz, $CDCl_3$)

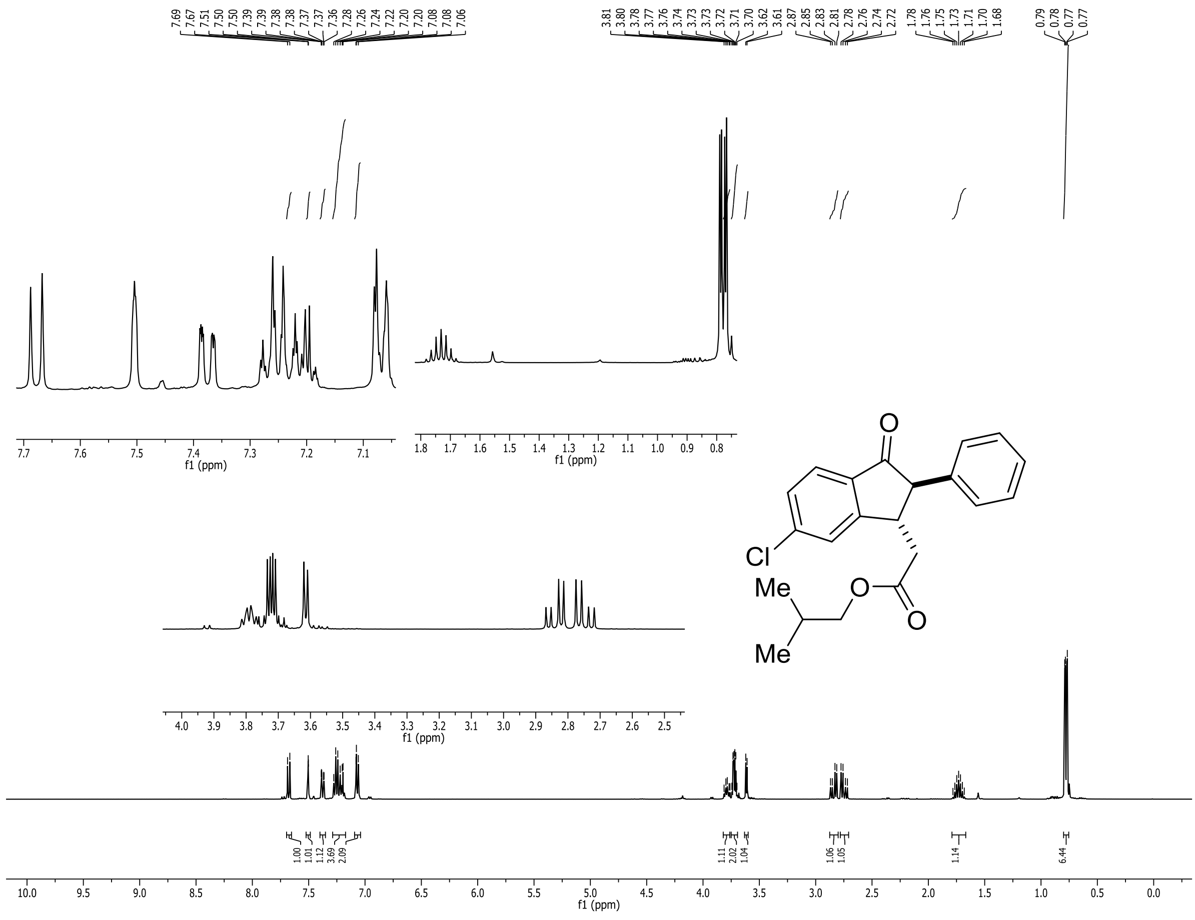

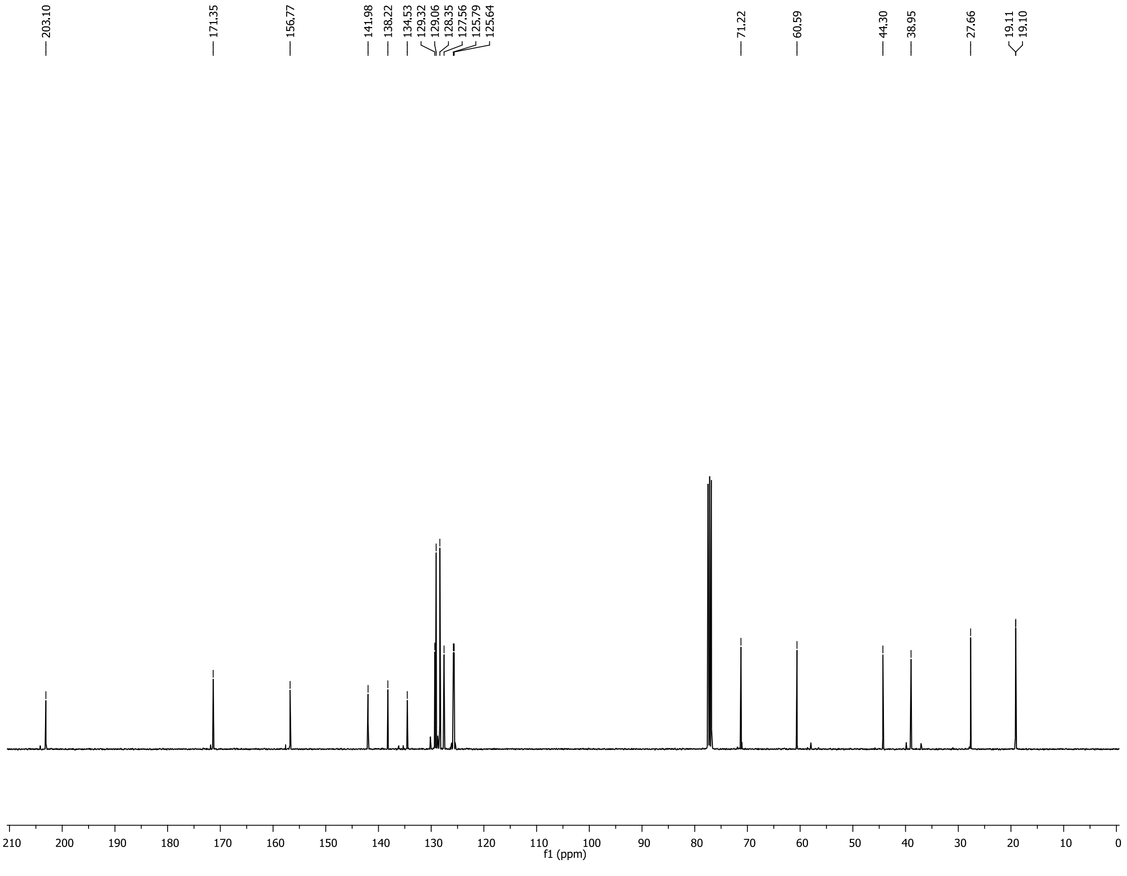

^{1}H, ^{13}C and ^{19}F NMR data of compound **6af** (400, 101, 376 MHz, $CDCl_3$)

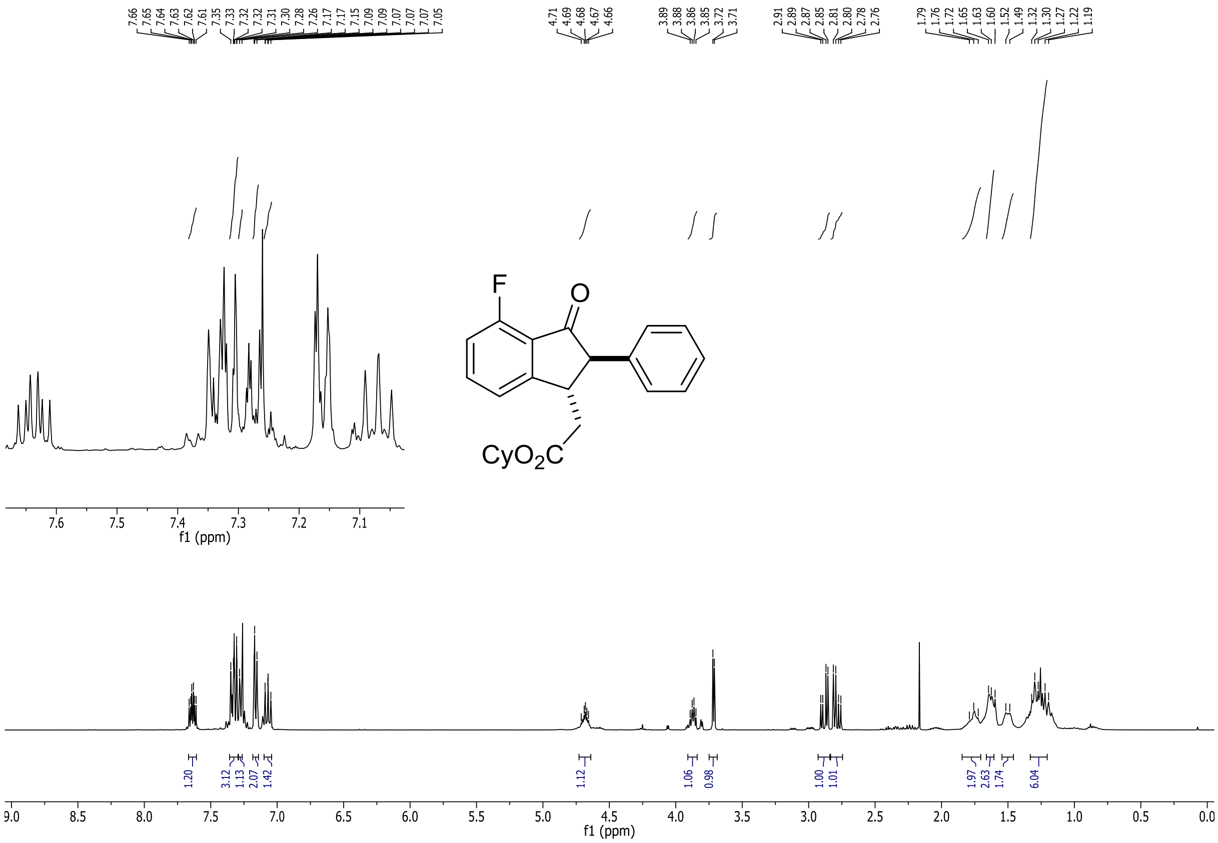

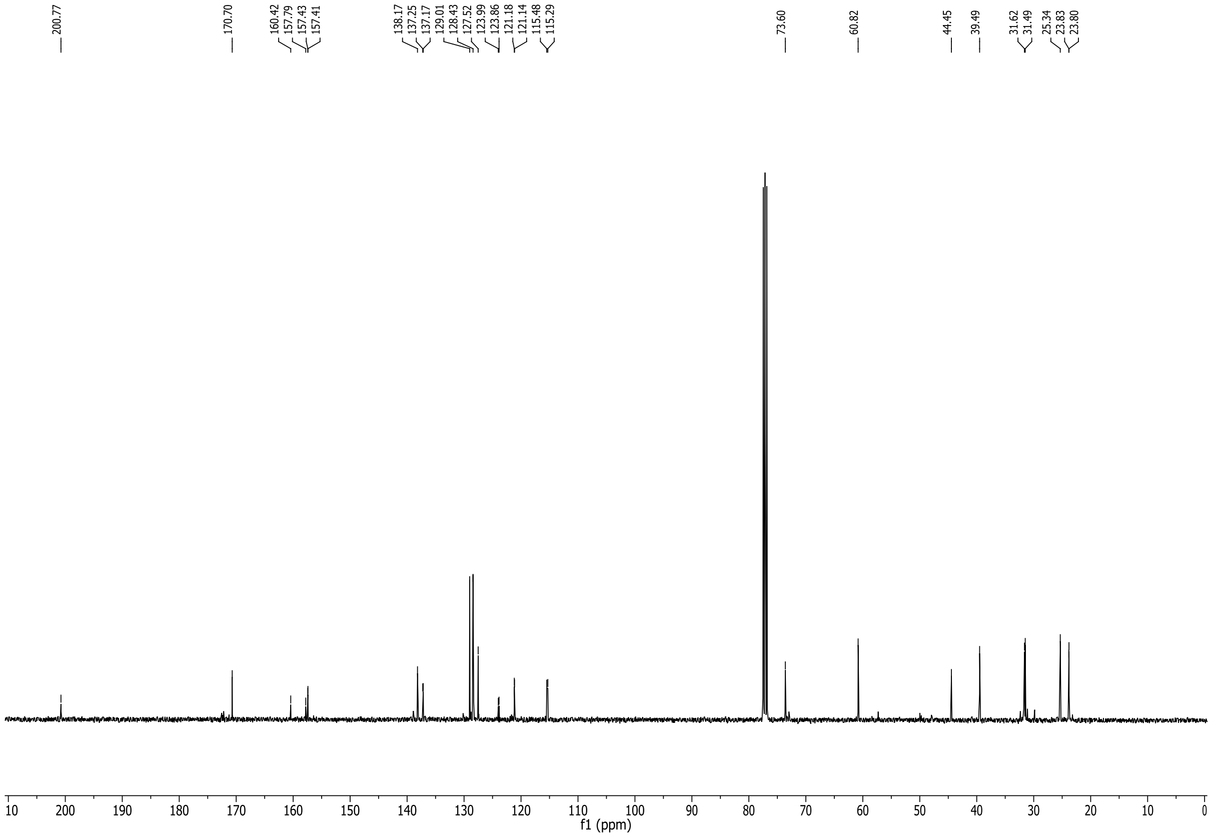

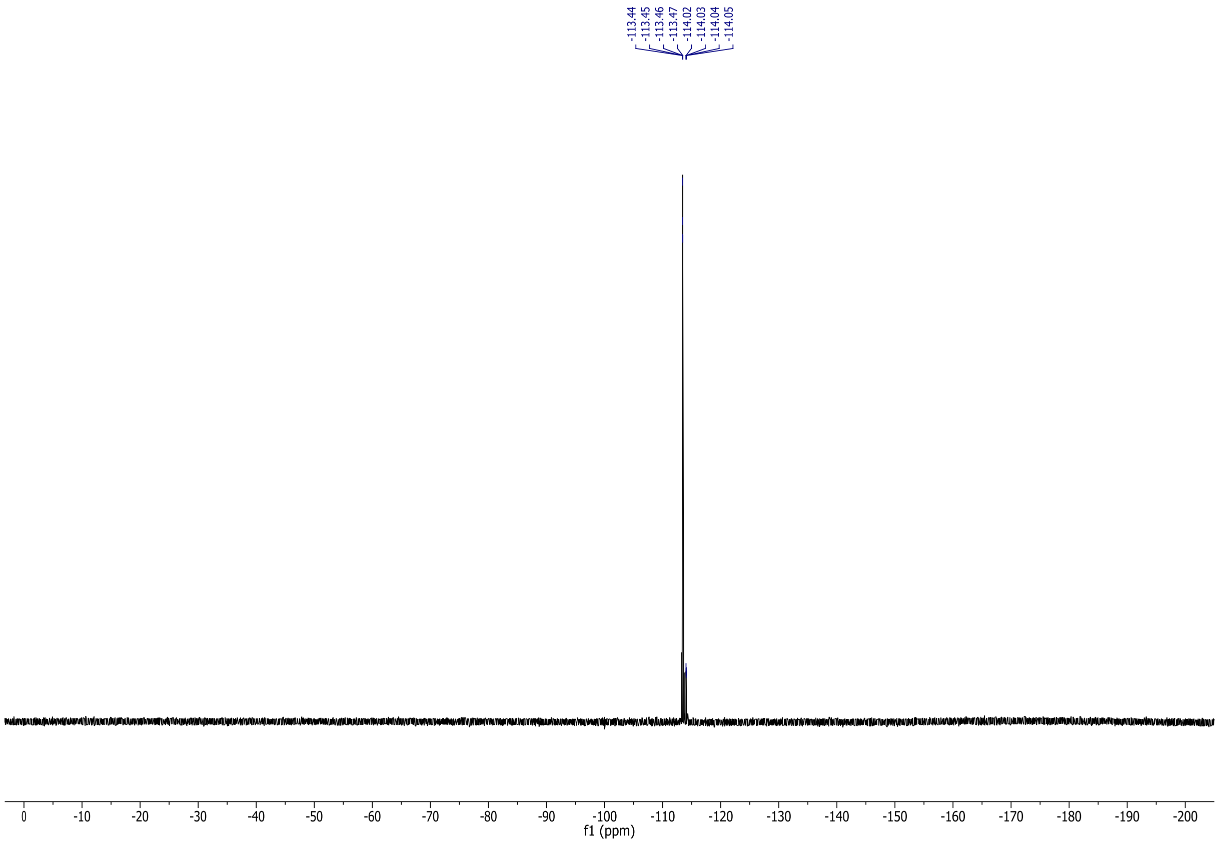
-113.44
-113.45
-113.46
-113.47
-114.02
-114.03
-114.04
-114.05
0
-10
-20
-30
-40
-50
-60
-70
-80
-90
-100
-110
-120
-130
-140
-150
-160
-170
-180
-190
-200
f1 (ppm)

^{1}H, ^{13}C and ^{19}F NMR data of compound **6ag** (500, 125, 470 MHz, $CDCl_3$)

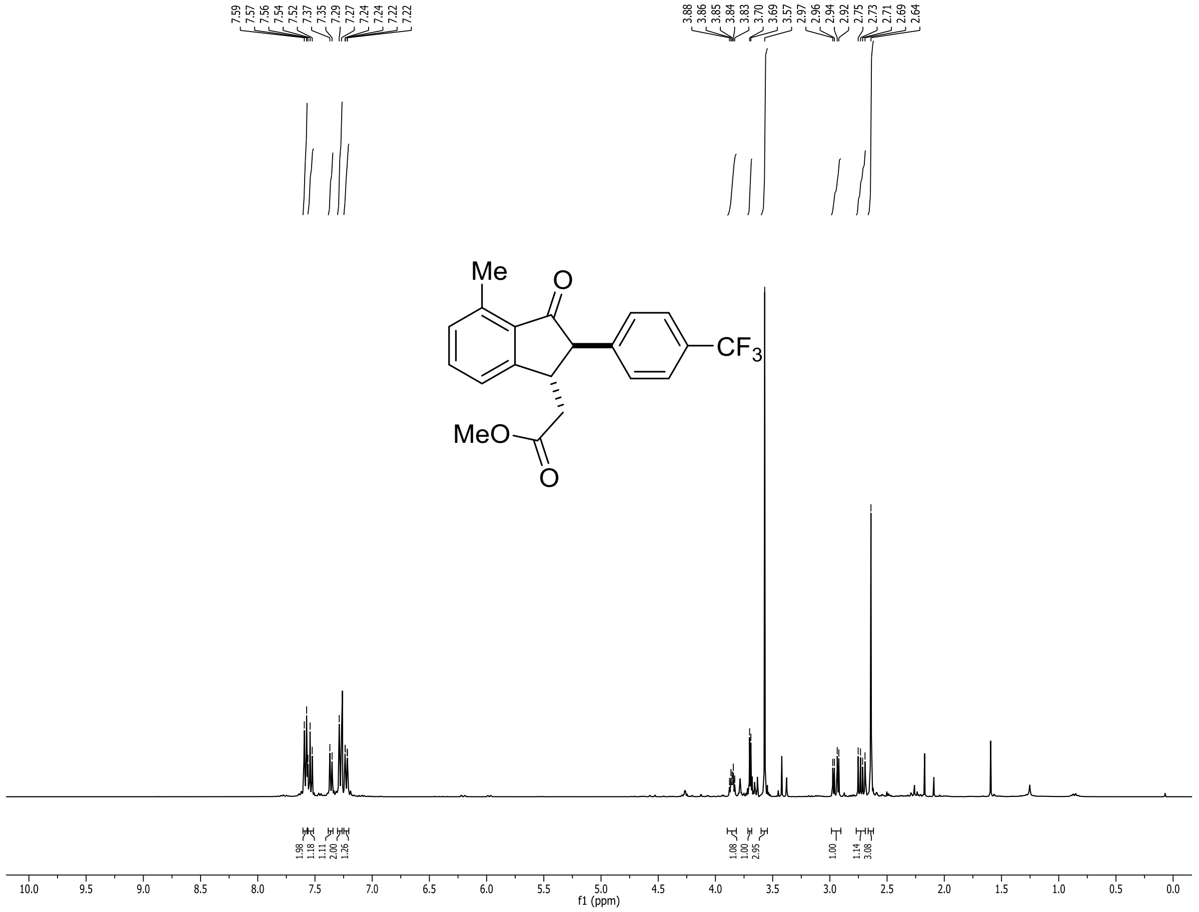

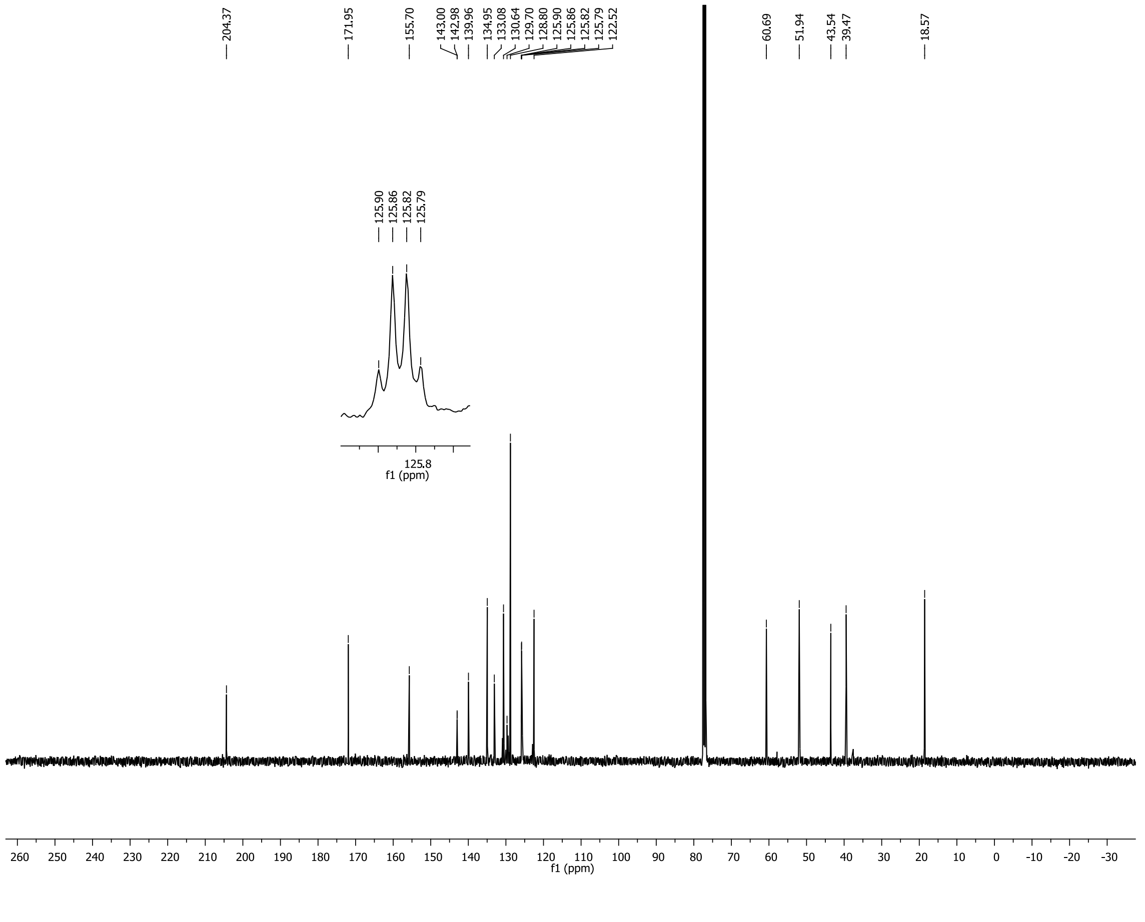

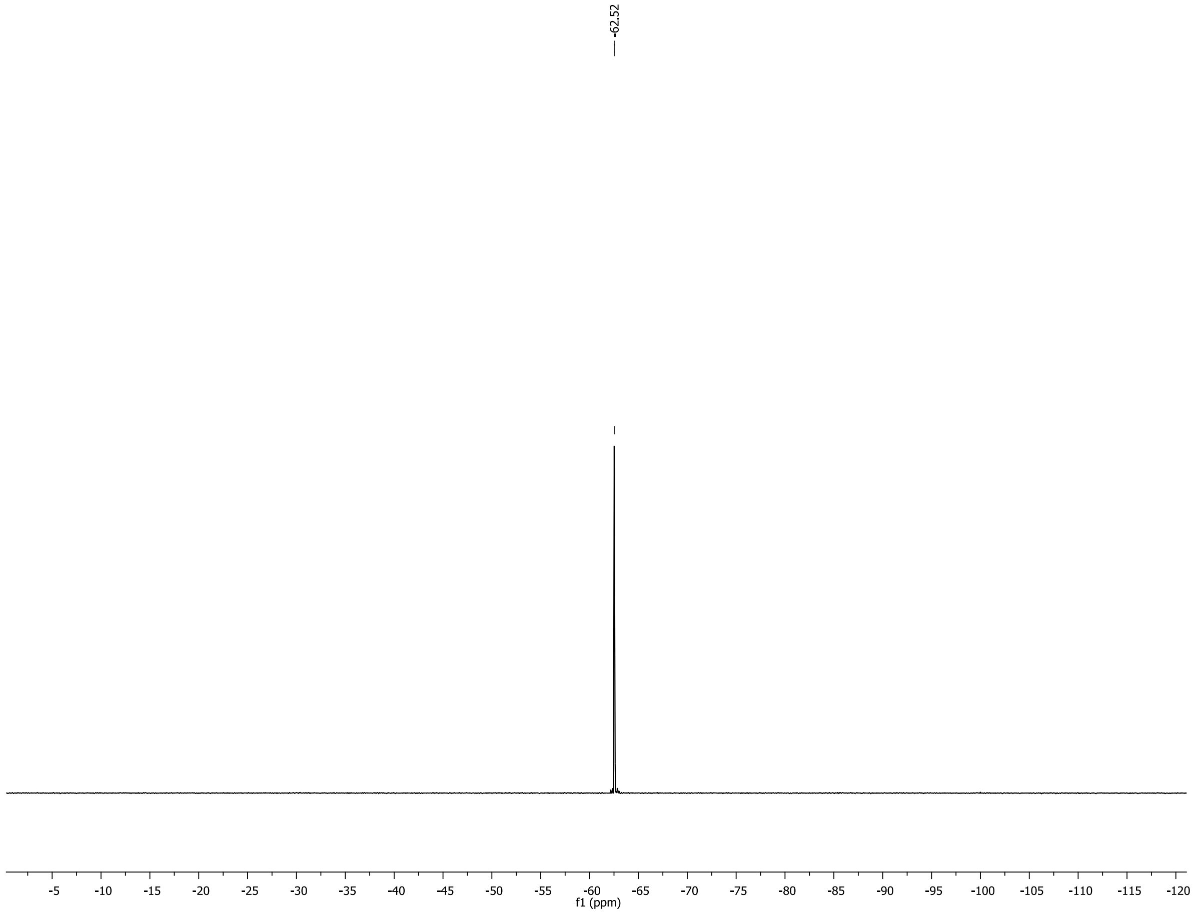

-62.52
-5
-10
-15
-20
-25
-30
-35
-40
-45
-50
-55
-60
-65
-70
-75
-80
-85
-90
-95
-100
-105
-110
-115
-120
f1 (ppm)

^{1}H and ^{13}C NMR data of compound **8** (500, 125 MHz, $CDCl_3$)

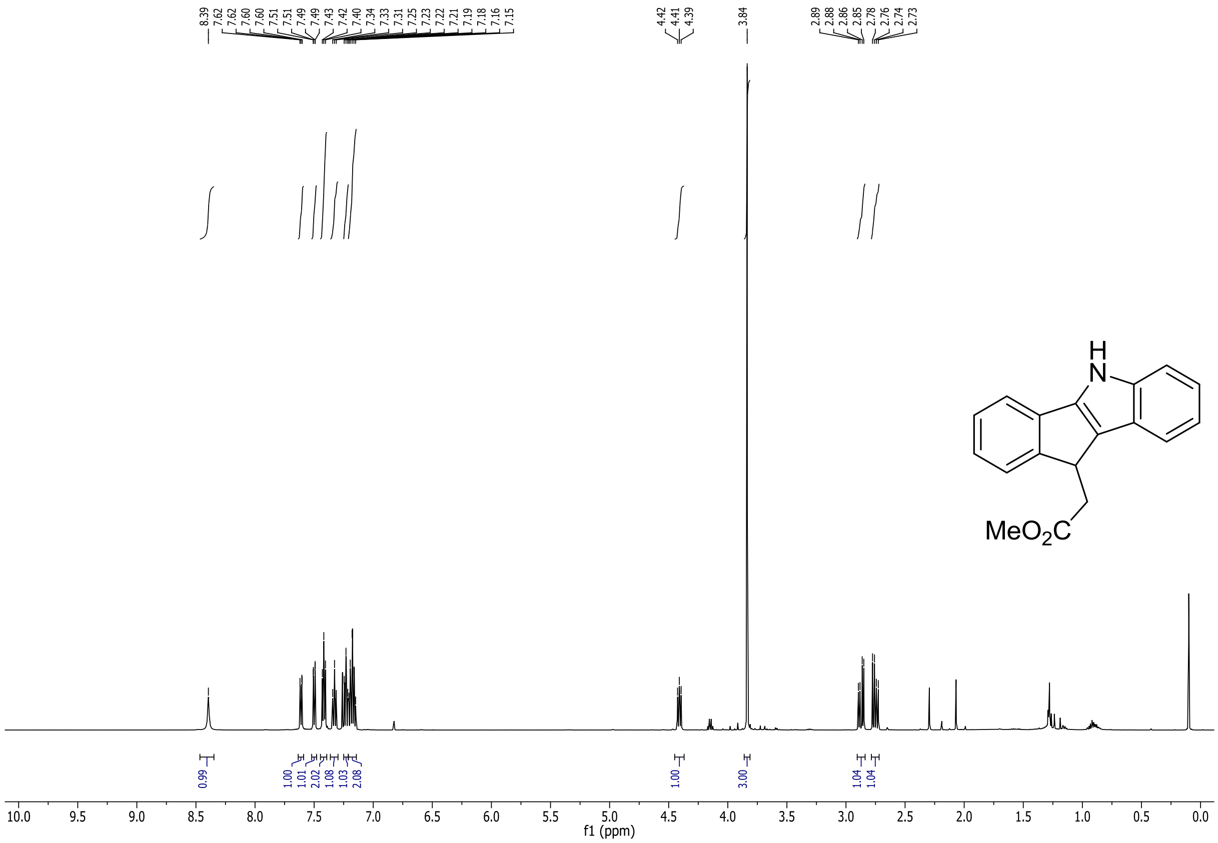

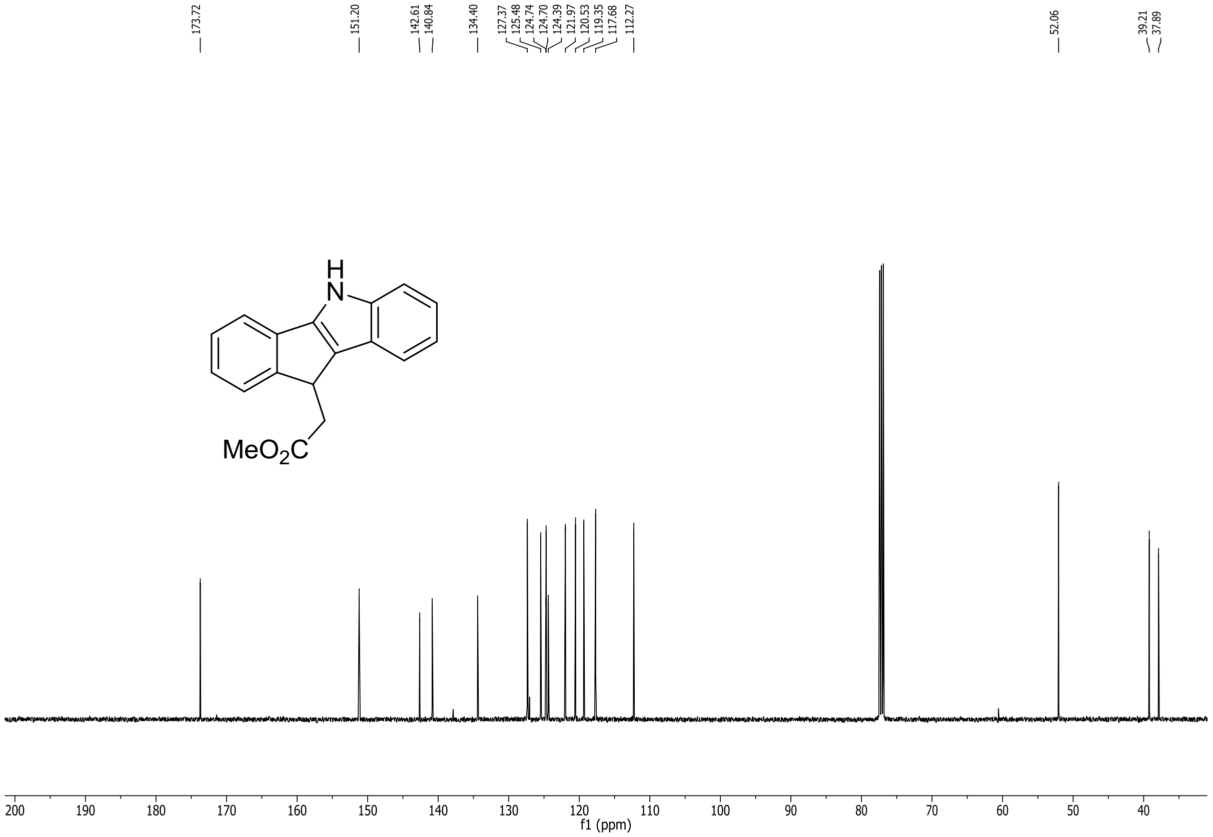

^{1}H and ^{13}C NMR data of compound **9** (500, 125 MHz, $CDCl_3$)

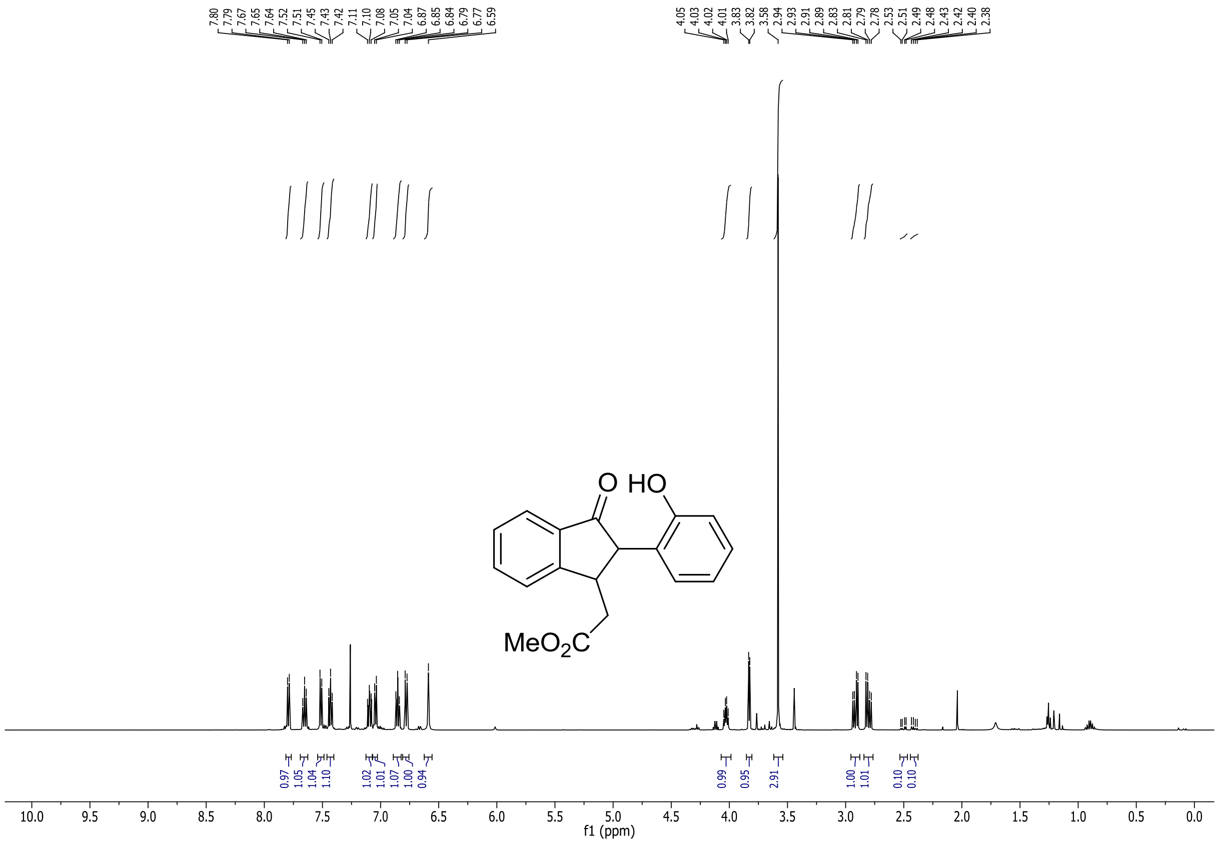

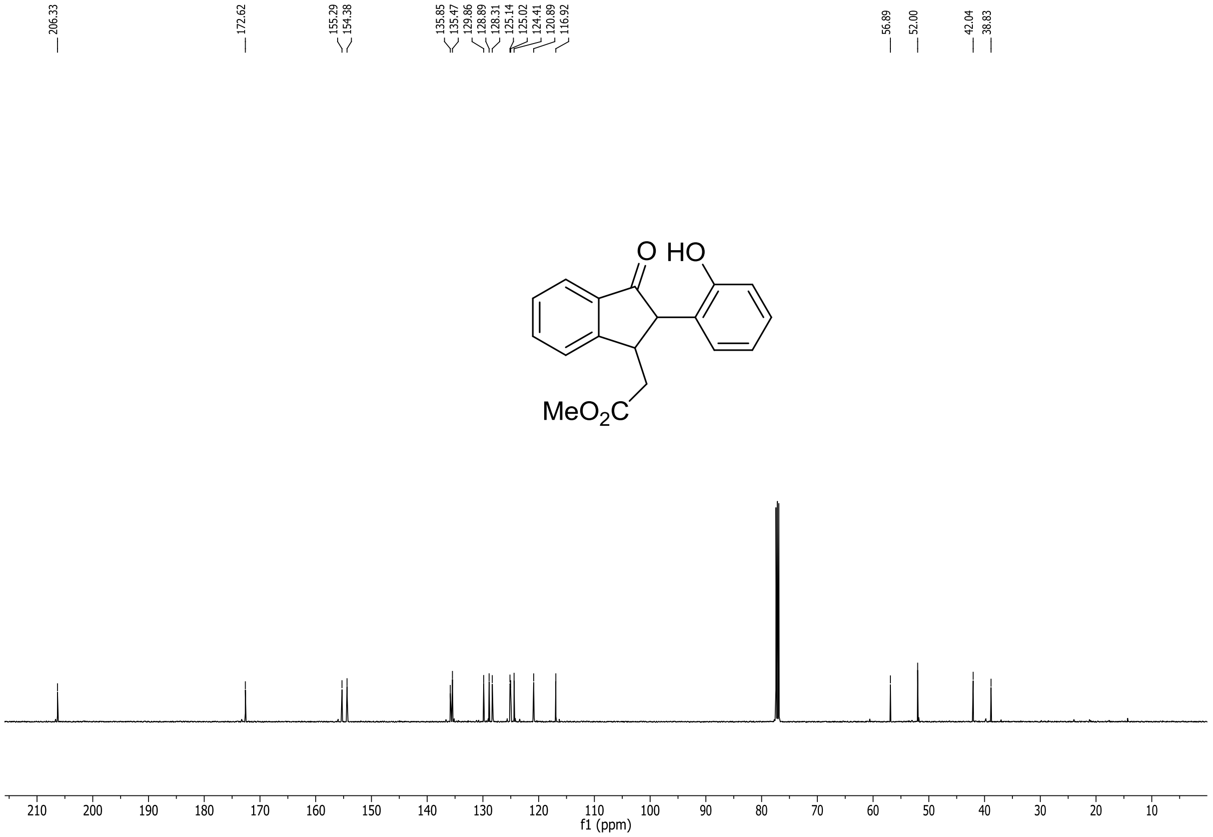

^{1}H and ^{13}C NMR data of compound **10** (500, 125 MHz, $CDCl_3$)

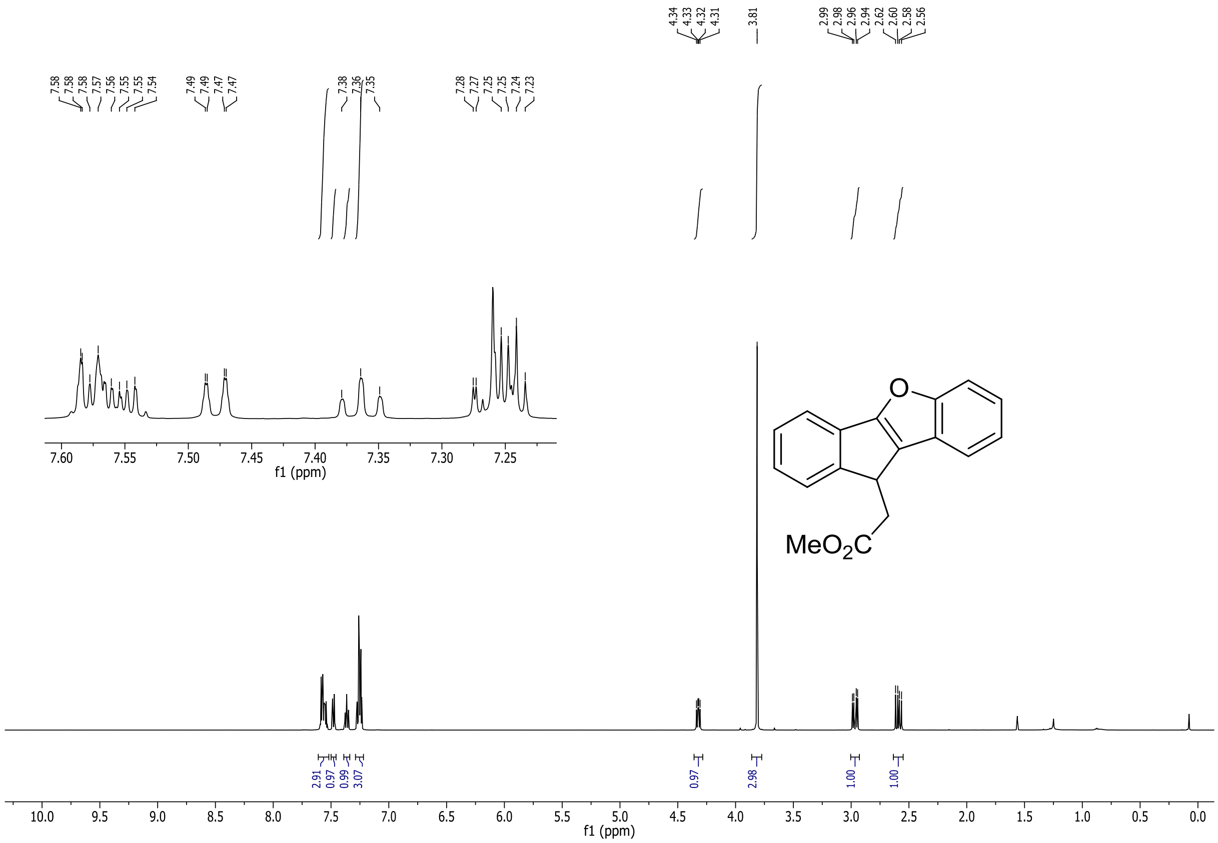

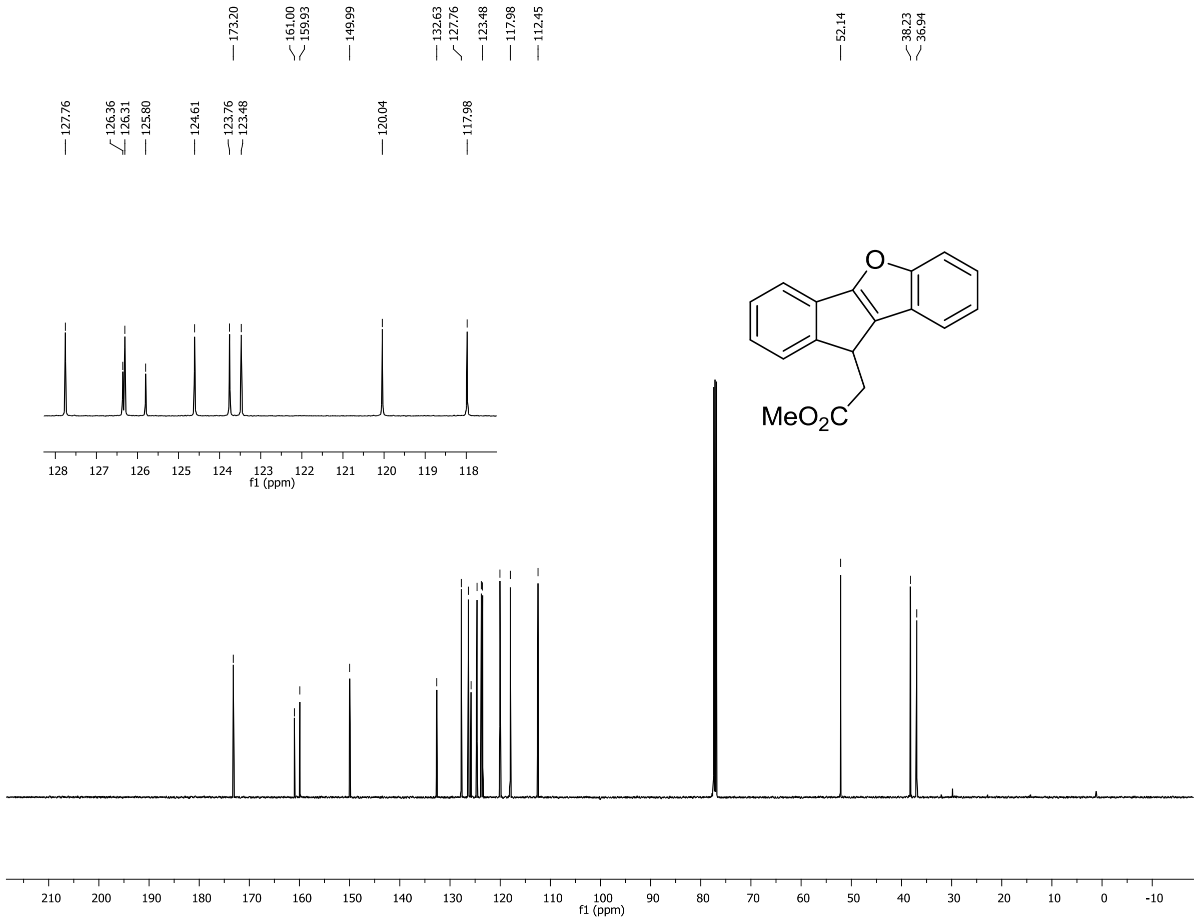

^{1}H and ^{13}C NMR data of compound **12a** (400, 101 MHz, $CDCl_3$)

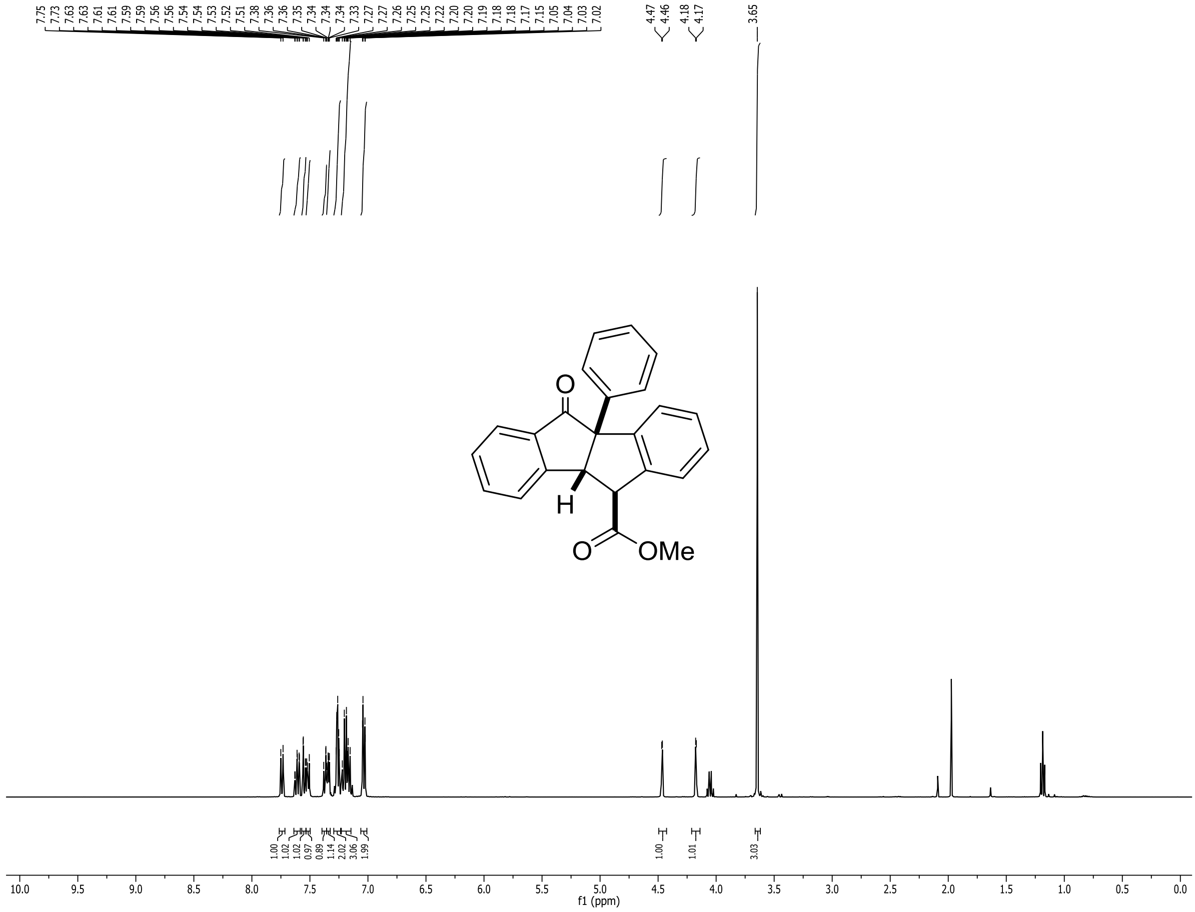
7.75 7.73 7.63 7.63 7.61 7.61 7.59 7.59 7.56 7.56 7.54 7.54 7.53 7.52 7.51 7.38 7.36 7.35 7.34 7.34 7.34 7.33 7.27 7.27 7.26 7.25 7.25 7.22 7.20 7.20 7.19 7.18 7.18 7.17 7.15 7.05 7.04 7.03 7.02
4.47 4.46 4.18 4.17
3.65
O
H
O
OMe
1.00 1.02 1.02 0.97 0.89 1.14 2.02 3.06 1.99
1.00
1.01
3.03
10.0 9.5 9.0 8.5 8.0 7.5 7.0 6.5 6.0 5.5 5.0 4.5 4.0 3.5 3.0 2.5 2.0 1.5 1.0 0.5 0.0
f1 (ppm)

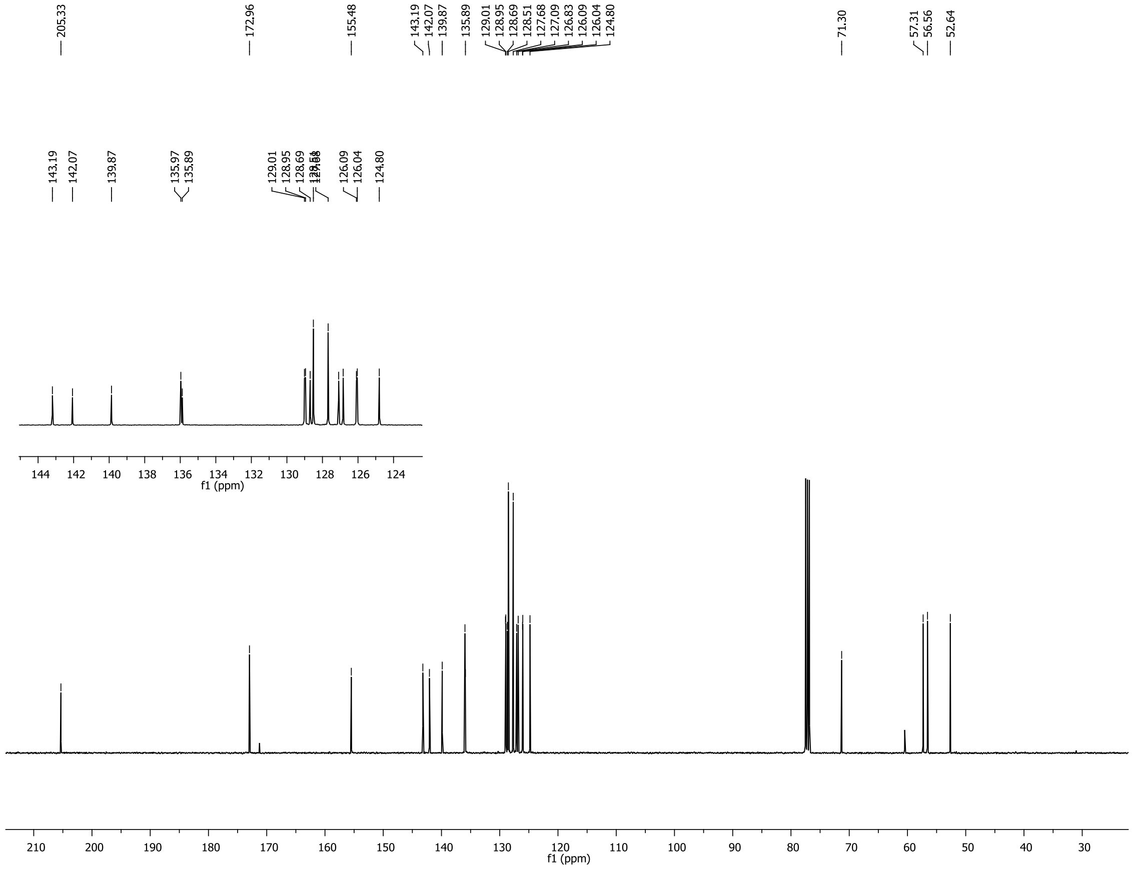
205.33
172.96
155.48
143.19 142.07 139.87 135.89 129.01 128.95 128.69 128.51 127.68 127.09 126.83 126.09 126.04 124.80
71.30
57.31 56.56 52.64
143.19 142.07 139.87 135.97 135.89 129.01 128.95 128.69 126.09 126.04 124.80
144 142 140 138 136 134 132 130 128 126 124
f1 (ppm)
210 200 190 180 170 160 150 140 130 120 110 100 90 80 70 60 50 40 30
f1 (ppm)

^{1}H and ^{13}C NMR data of compound **12b** (400, 101 MHz, $CDCl_3$)

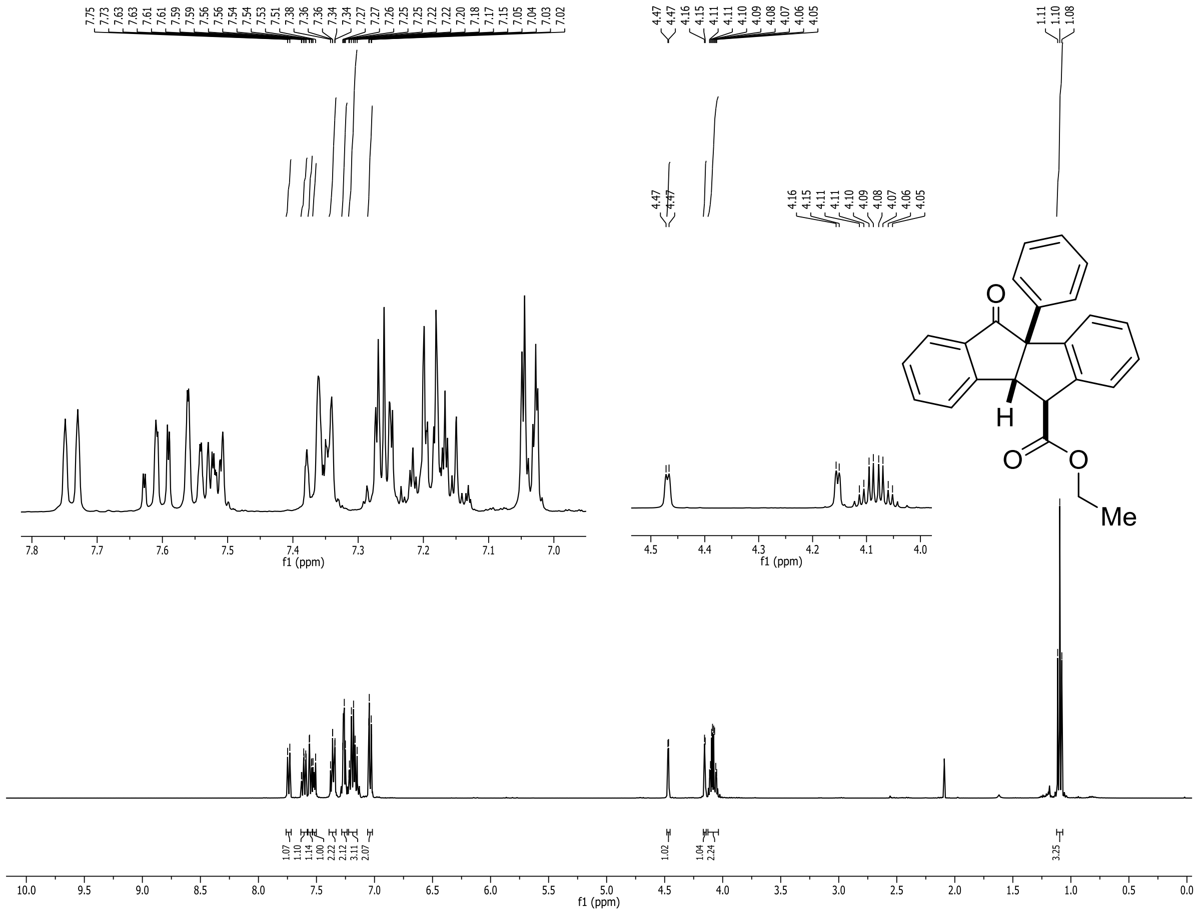

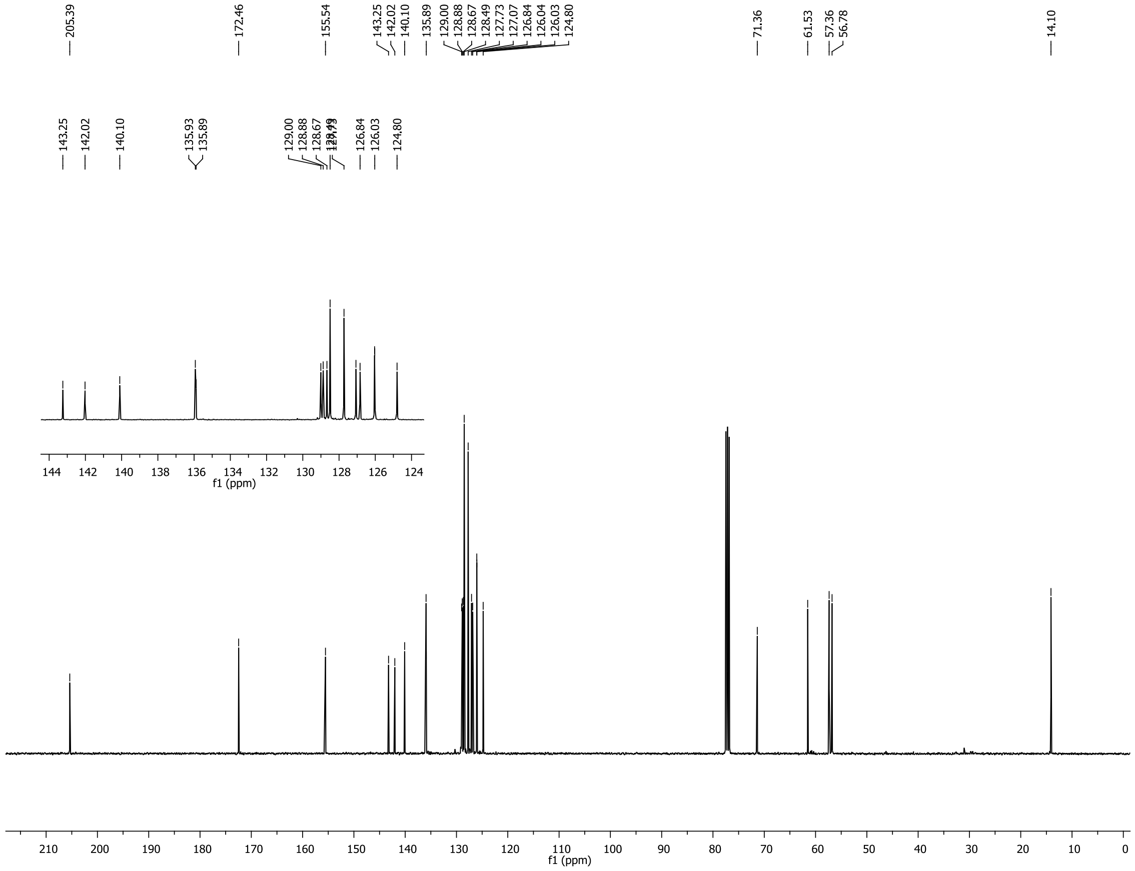

^{1}H and ^{13}C NMR data of compound **12c** (500, 125 MHz, $CDCl_3$)

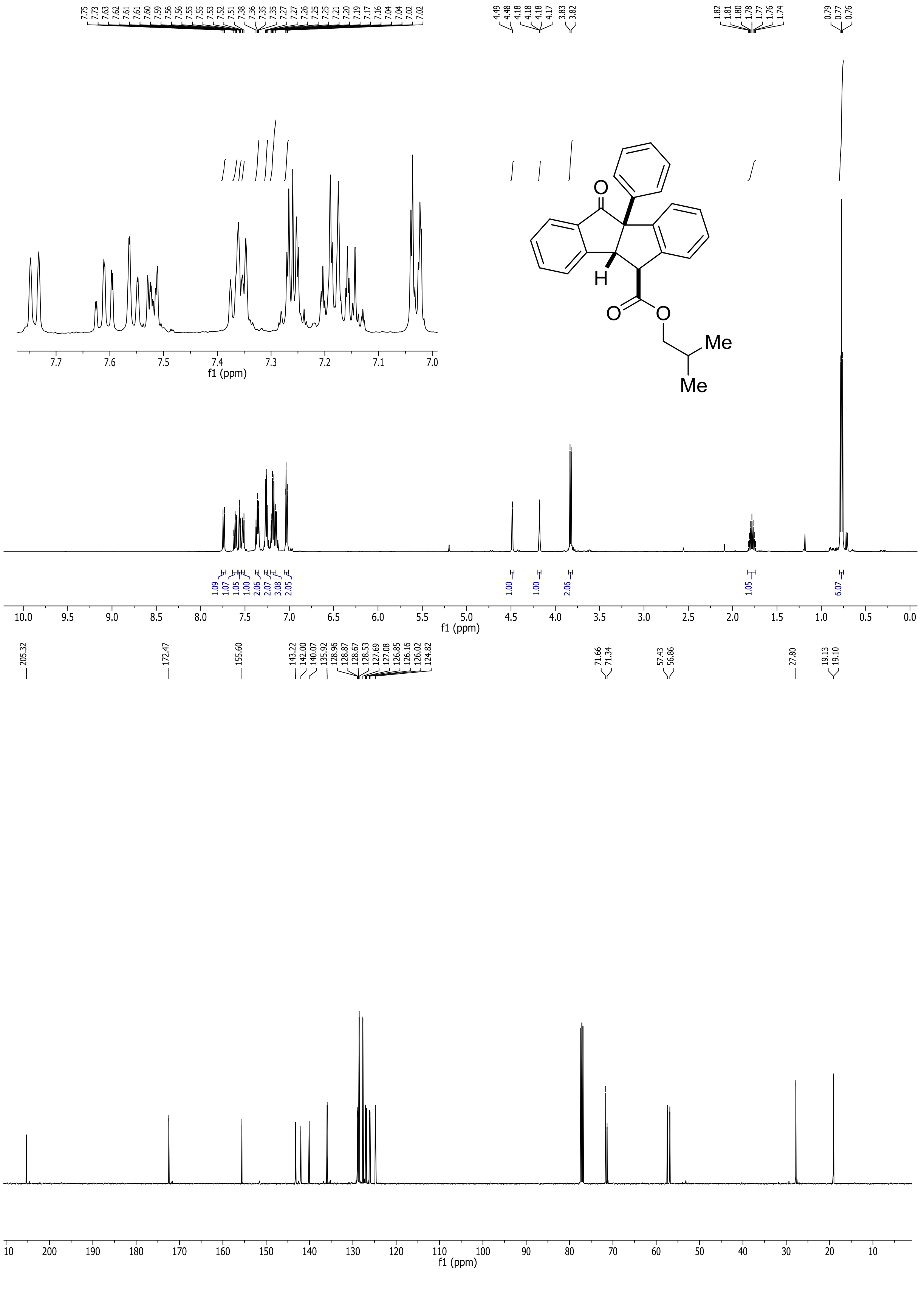

^{1}H and ^{13}C NMR data of compound **12d** (400, 101 MHz, $CDCl_3$)

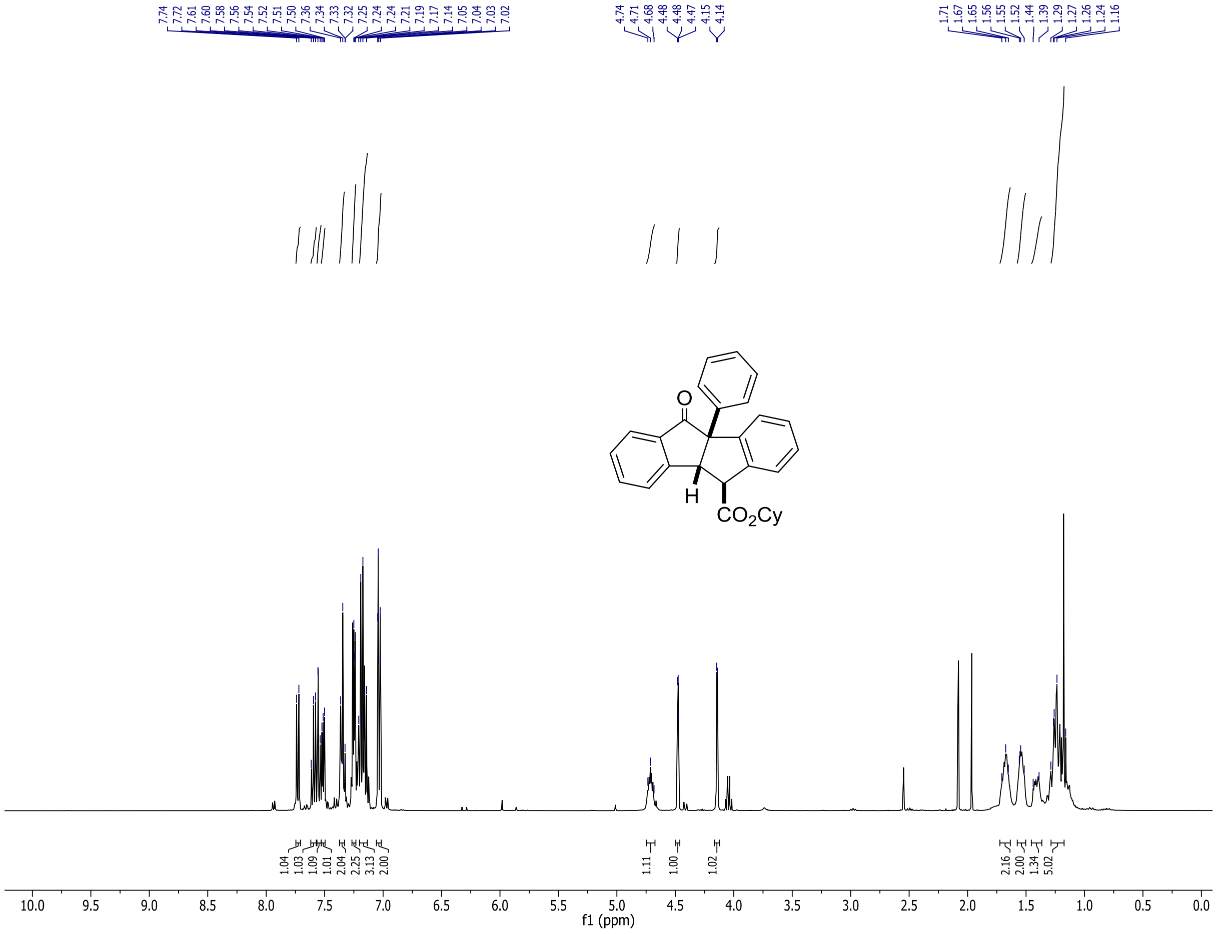

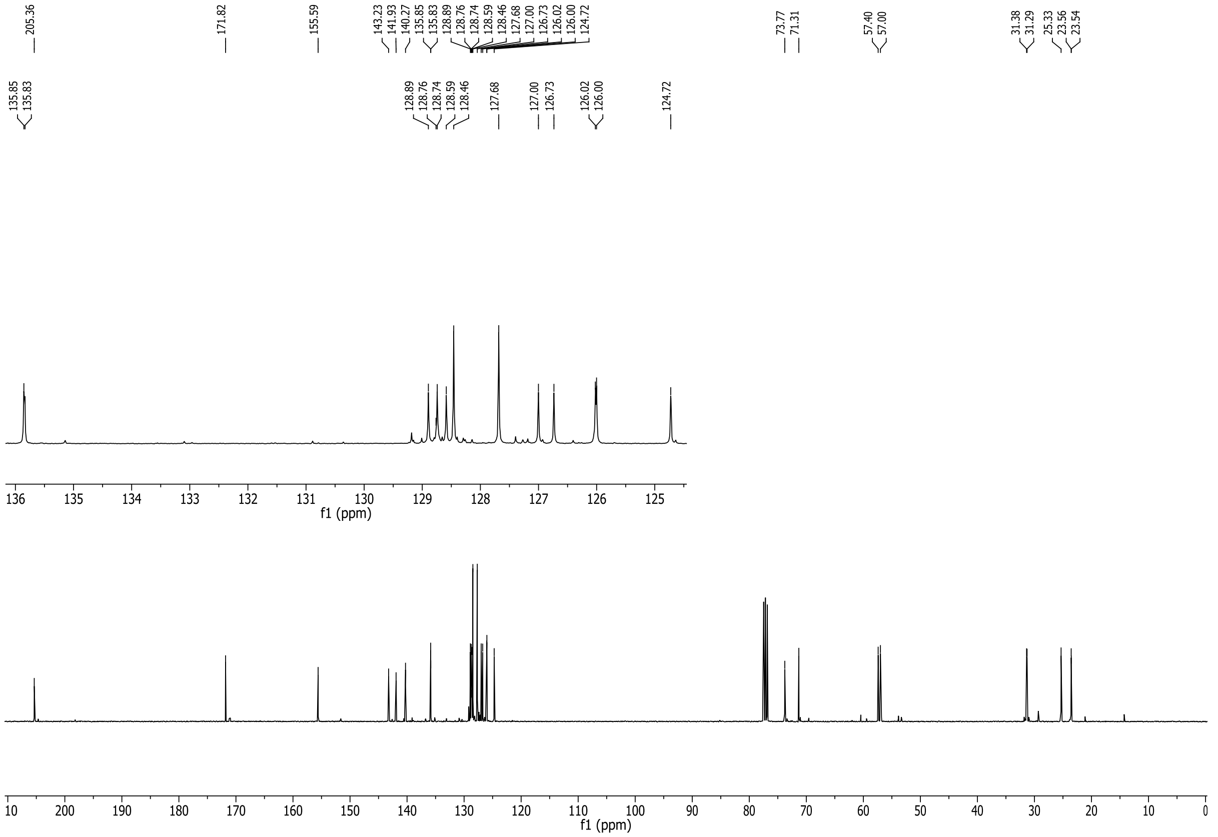

^{1}H and ^{13}C NMR data of compound **12e** (300, 75 MHz, $CDCl_3$)

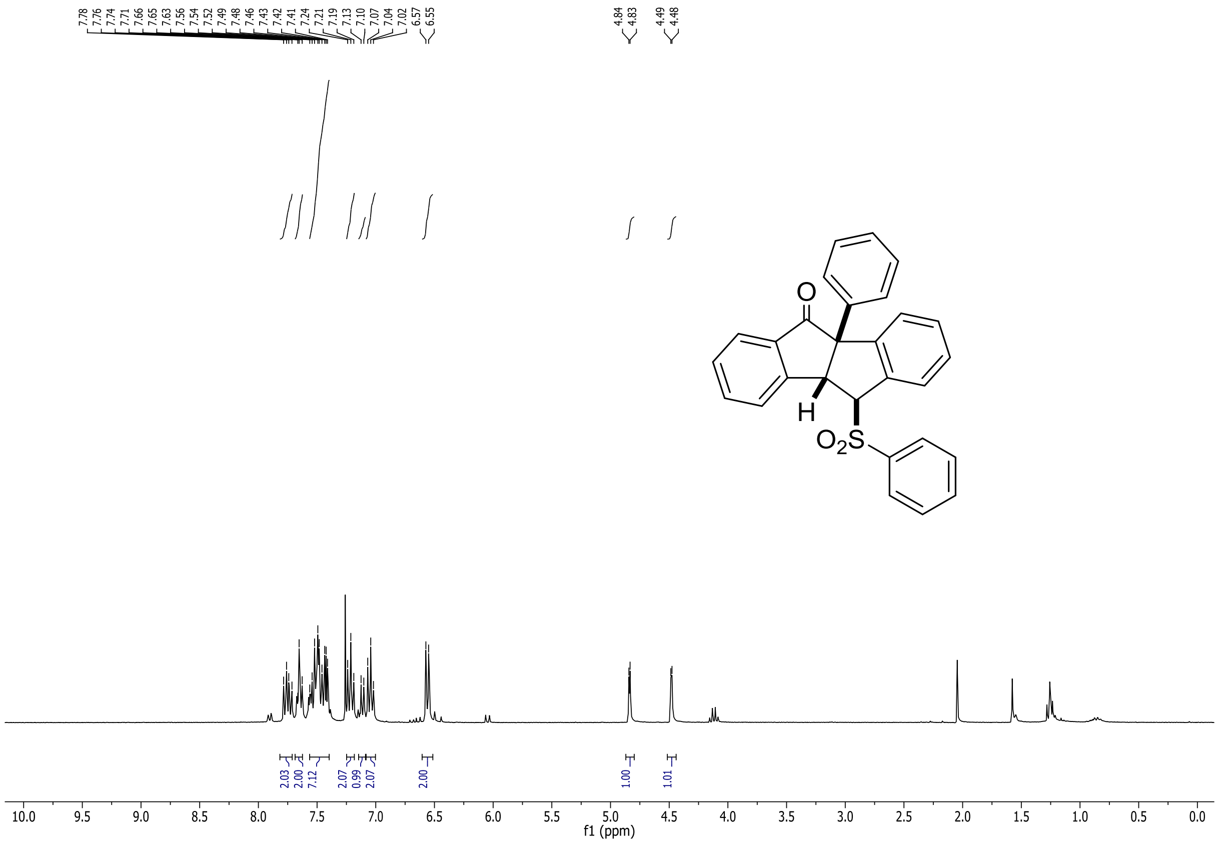

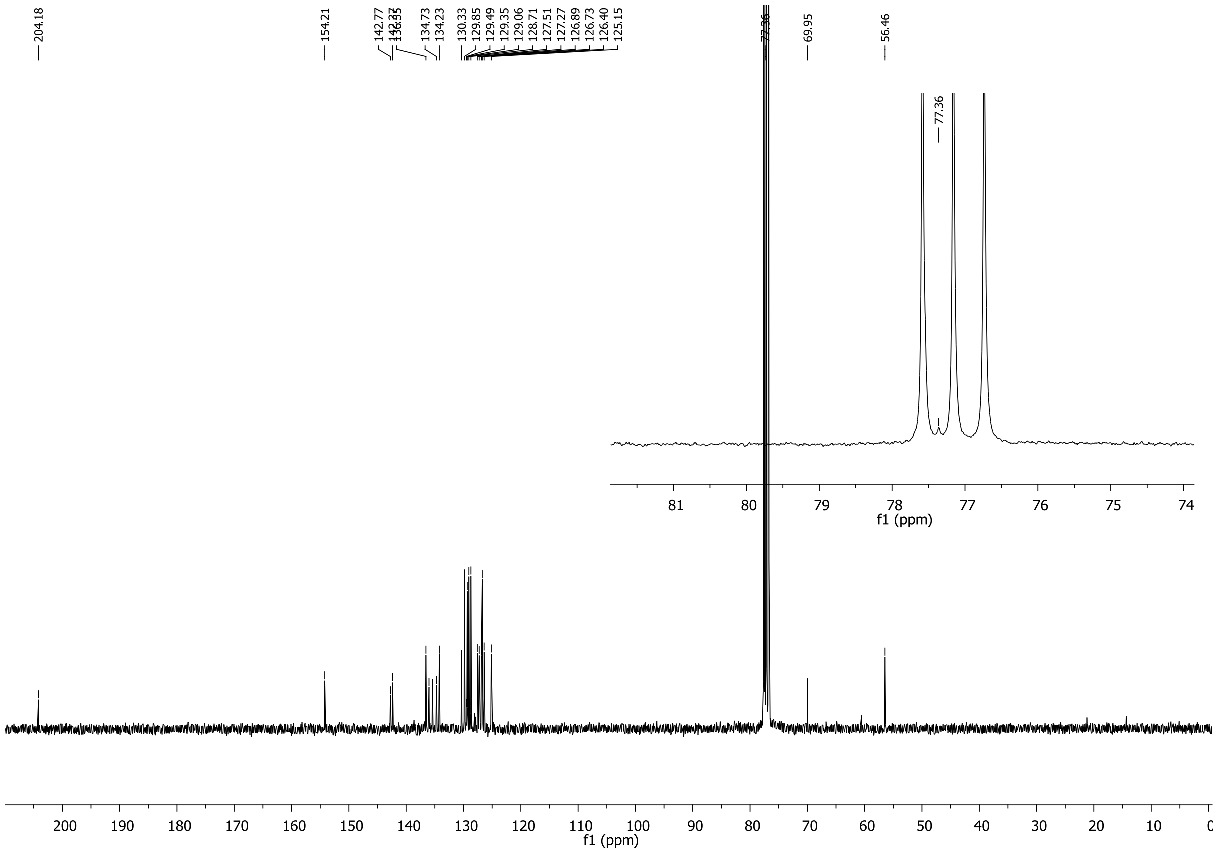

^{1}H, ^{13}C and ^{19}F NMR data of compound **12f** (500, 125, 470 MHz, $CDCl_3$)

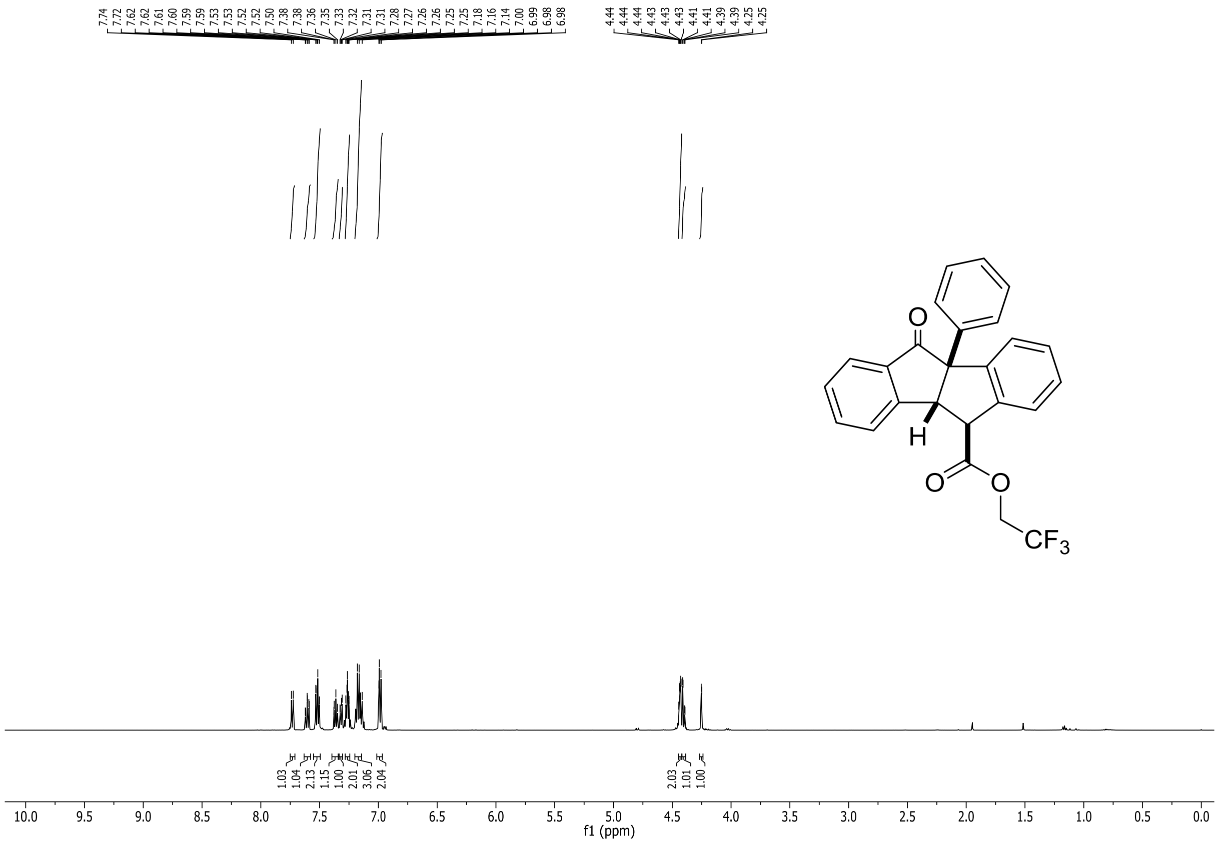

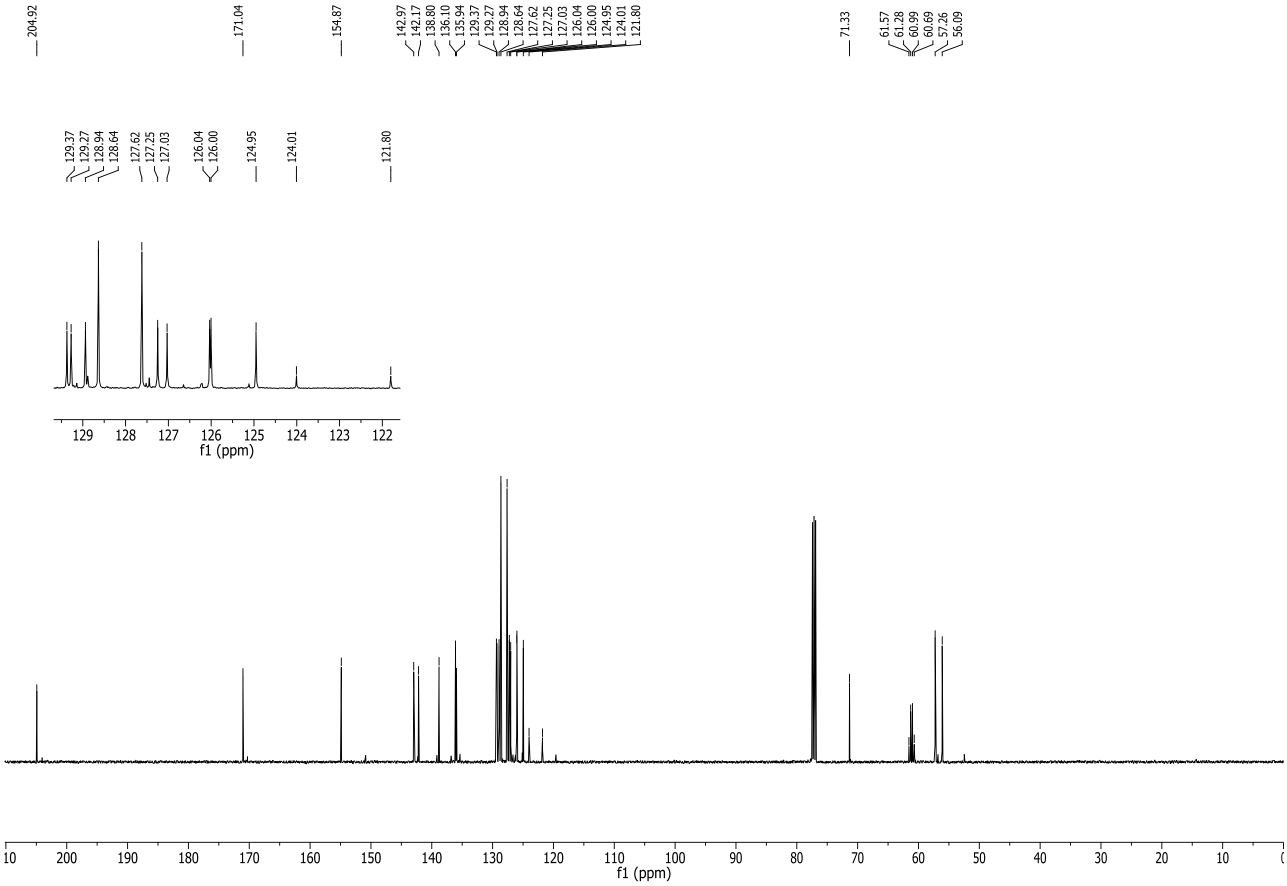

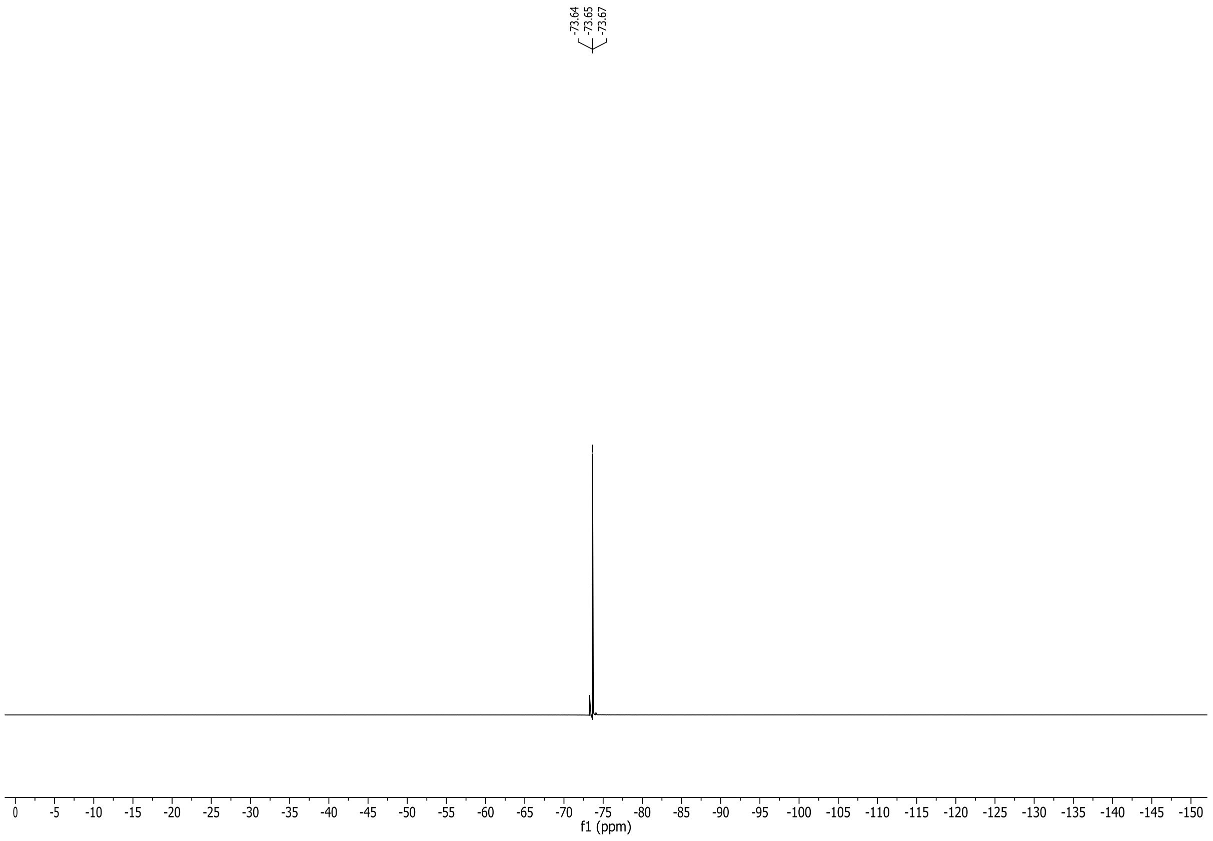
-73.64
-73.65
-73.67
0 -5 -10 -15 -20 -25 -30 -35 -40 -45 -50 -55 -60 -65 -70 -75 -80 -85 -90 -95 -100 -105 -110 -115 -120 -125 -130 -135 -140 -145 -150
f1 (ppm)

^1H and ^{13}C NMR data of compound **12g** (400, 101 MHz, $CDCl_3$)

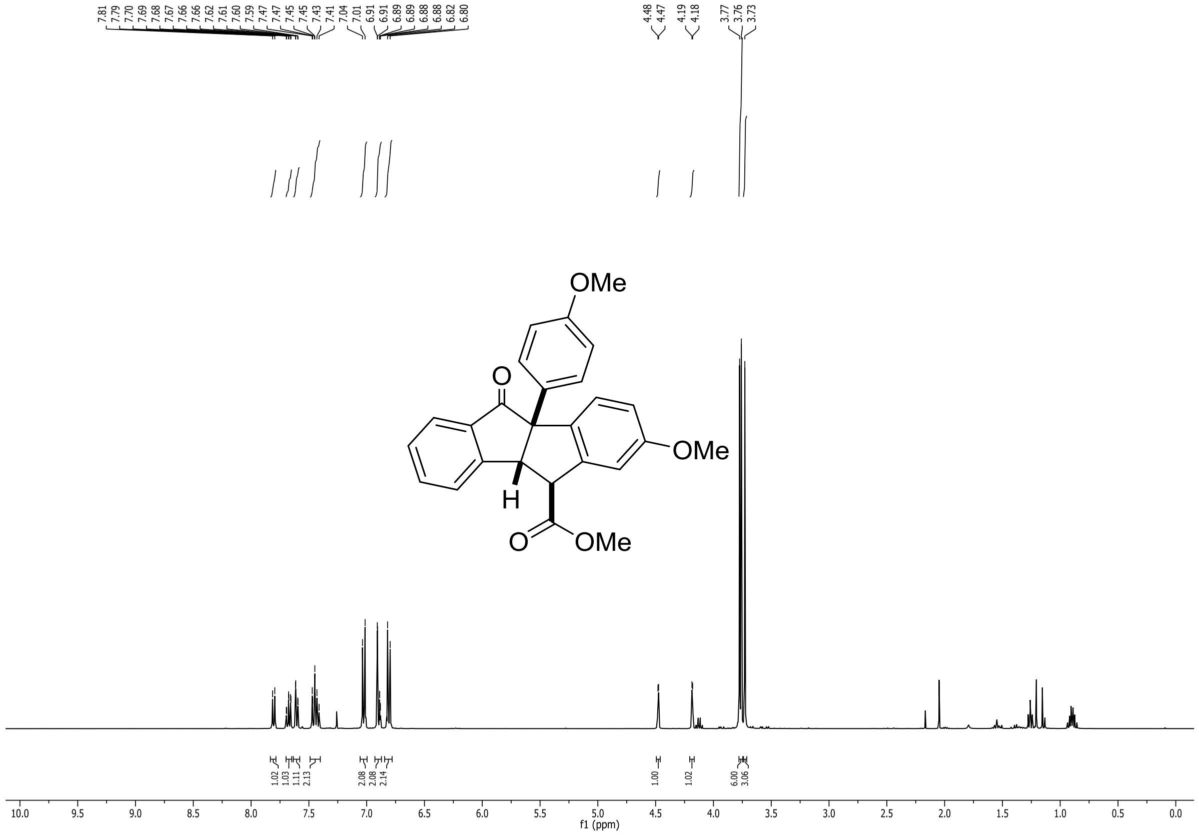

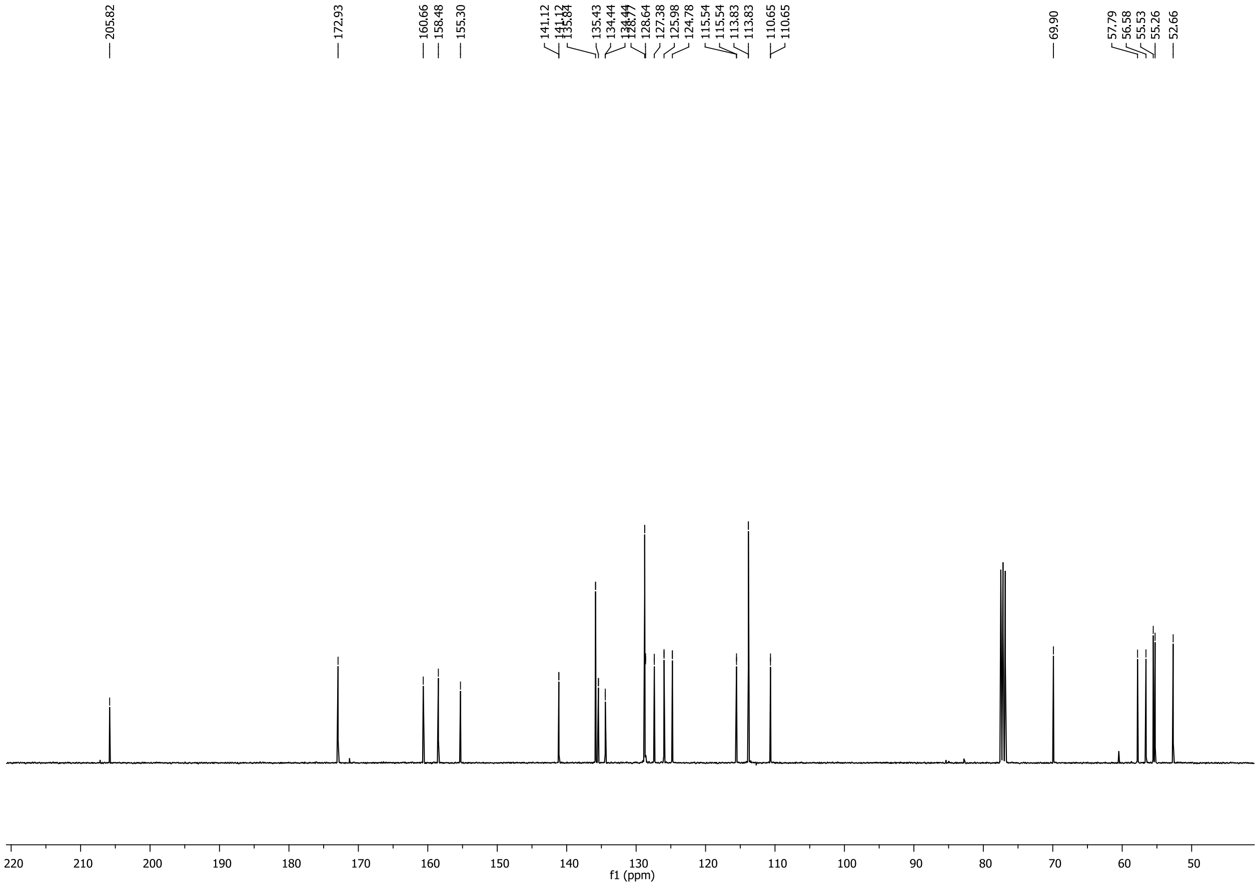

^{1}H and ^{13}C NMR data of compound **12h** (400, 101 MHz, $CDCl_3$)

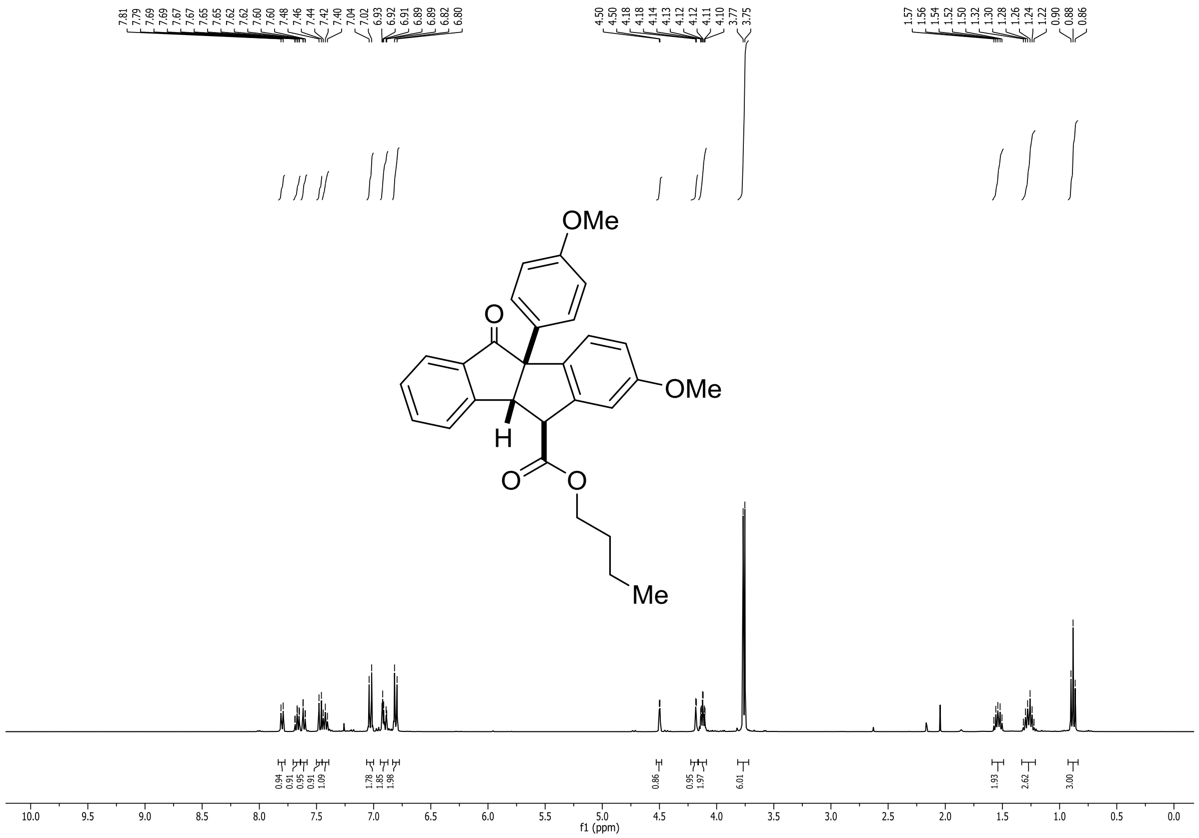

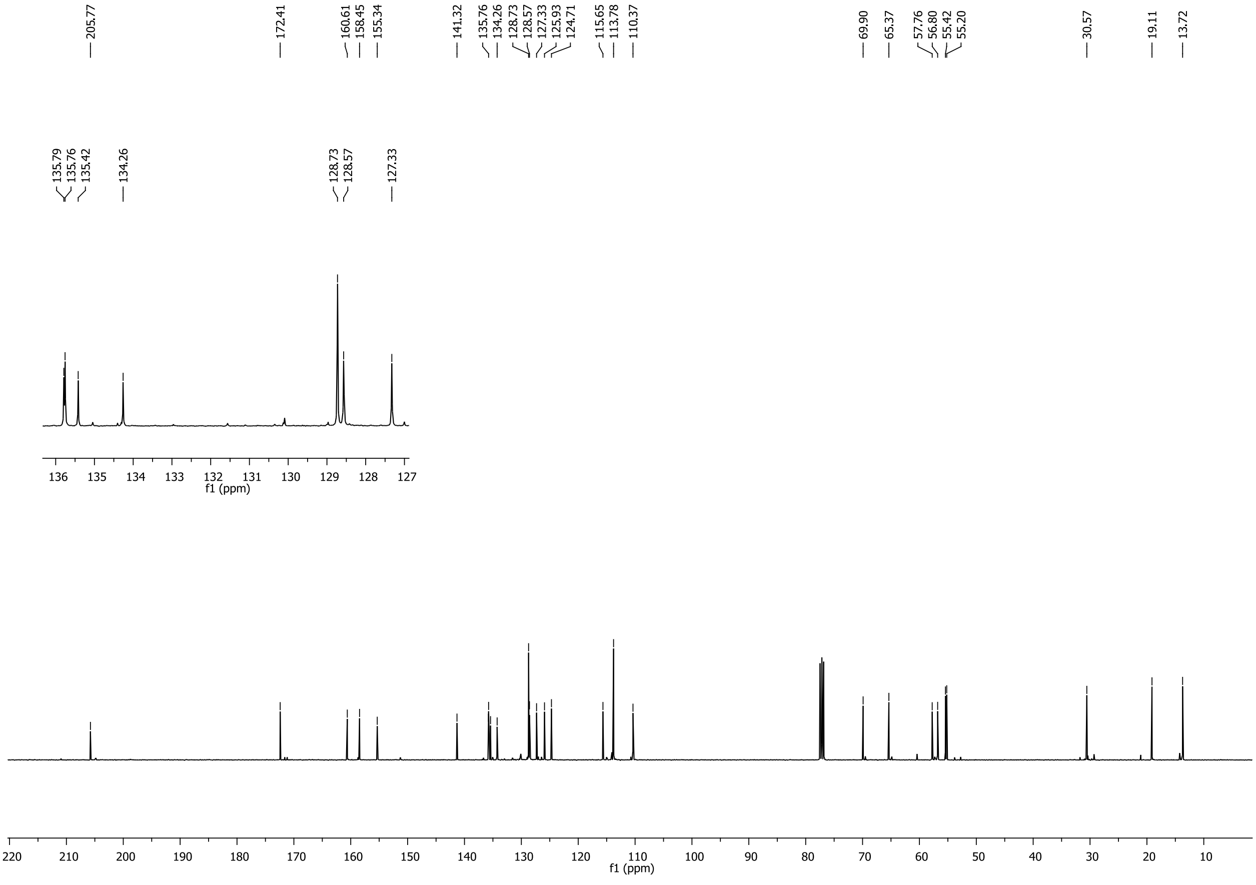

^{1}H and ^{13}C NMR data of compound **12i** (400, 101 MHz, $CDCl_3$)

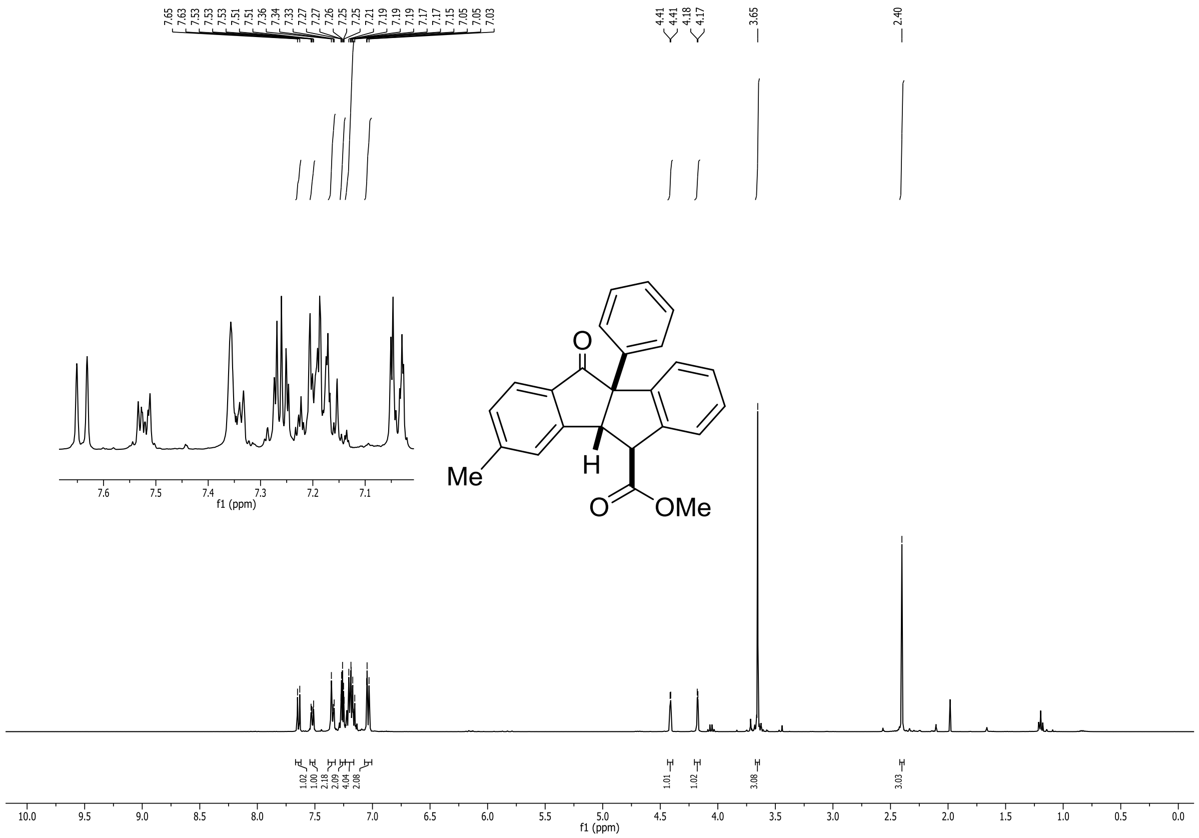

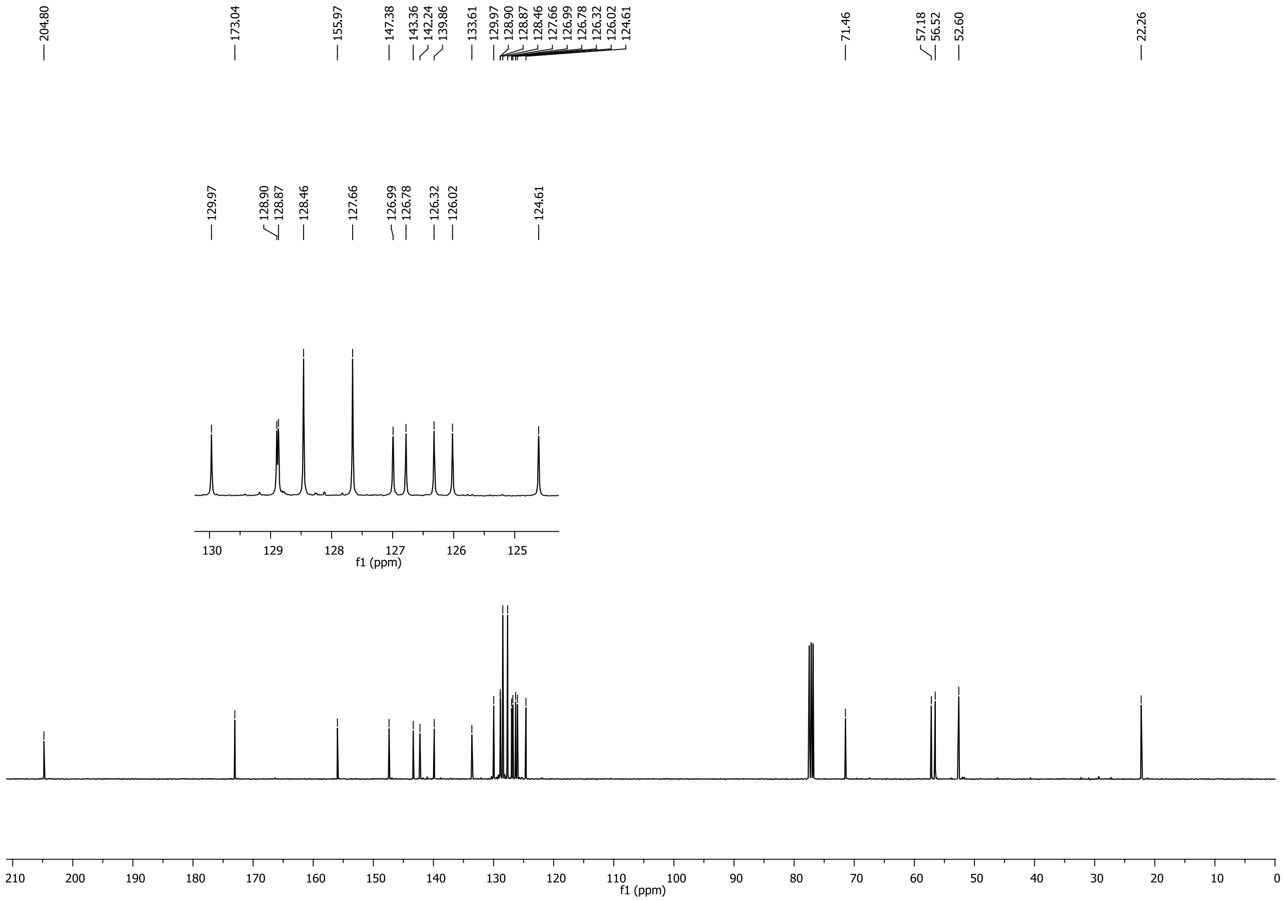

^{1}H and ^{13}C NMR data of compound **12j** (400, 101 MHz, $CDCl_3$)

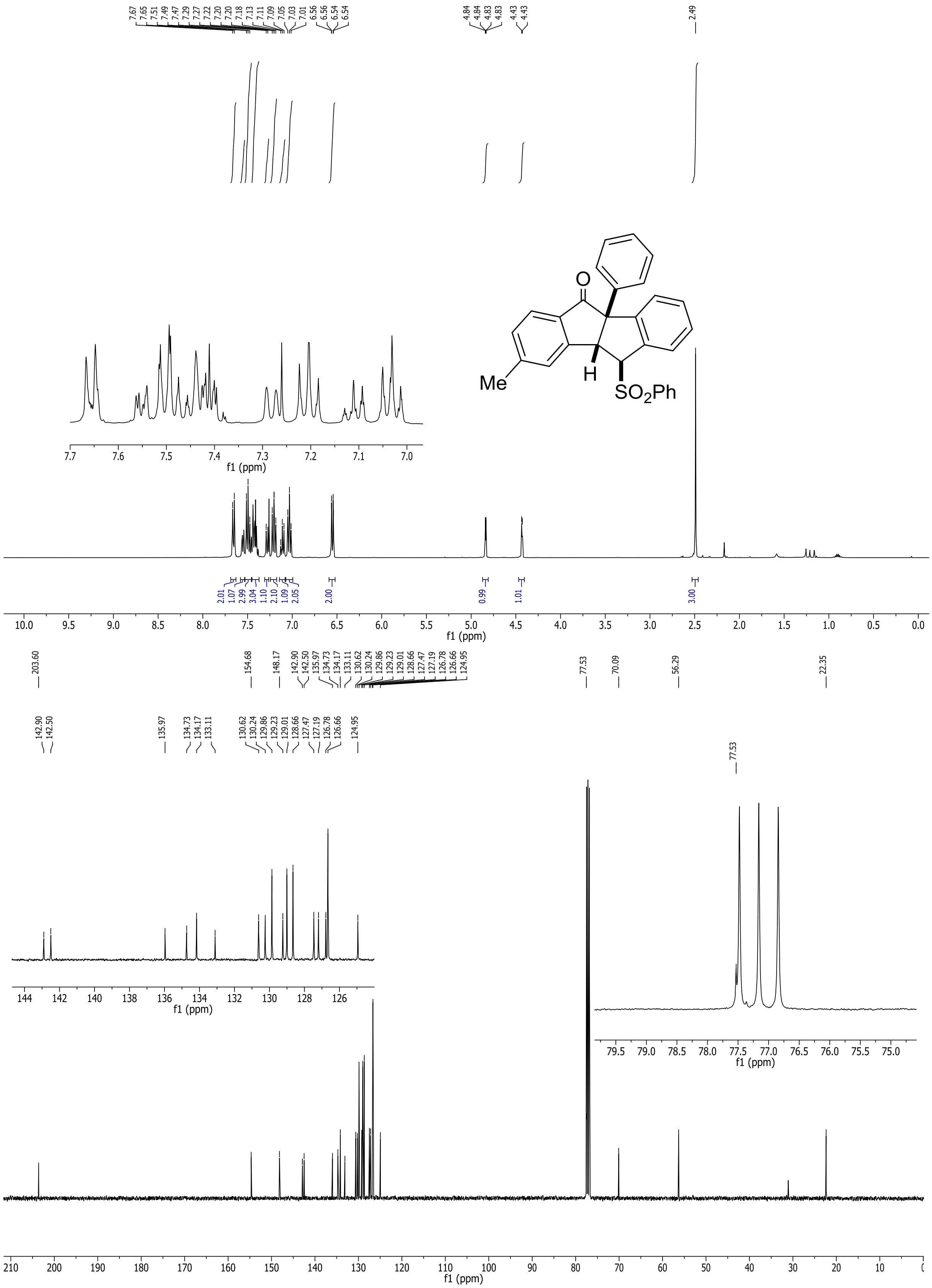

^{1}H, ^{13}C and ^{19}F NMR data of compound **12k** (400, 101, 376 MHz, $CDCl_3$)

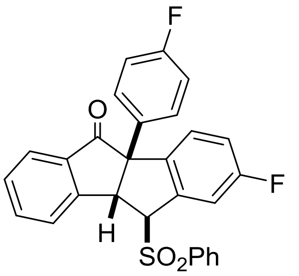

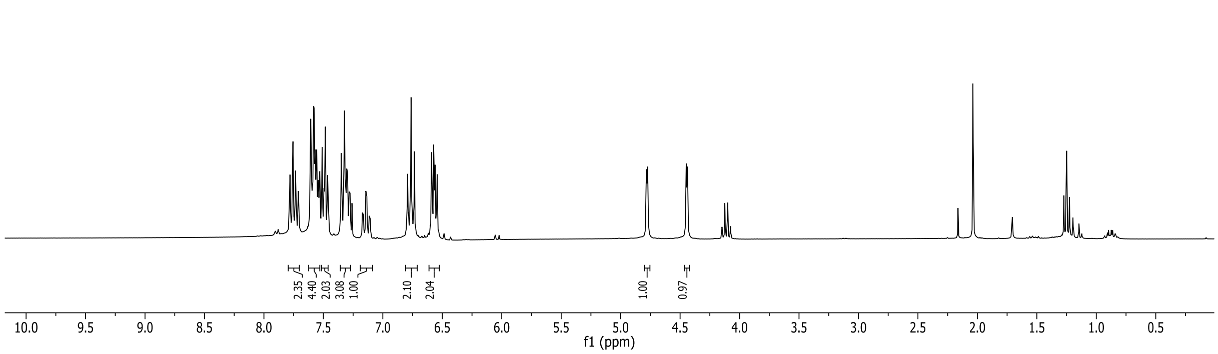

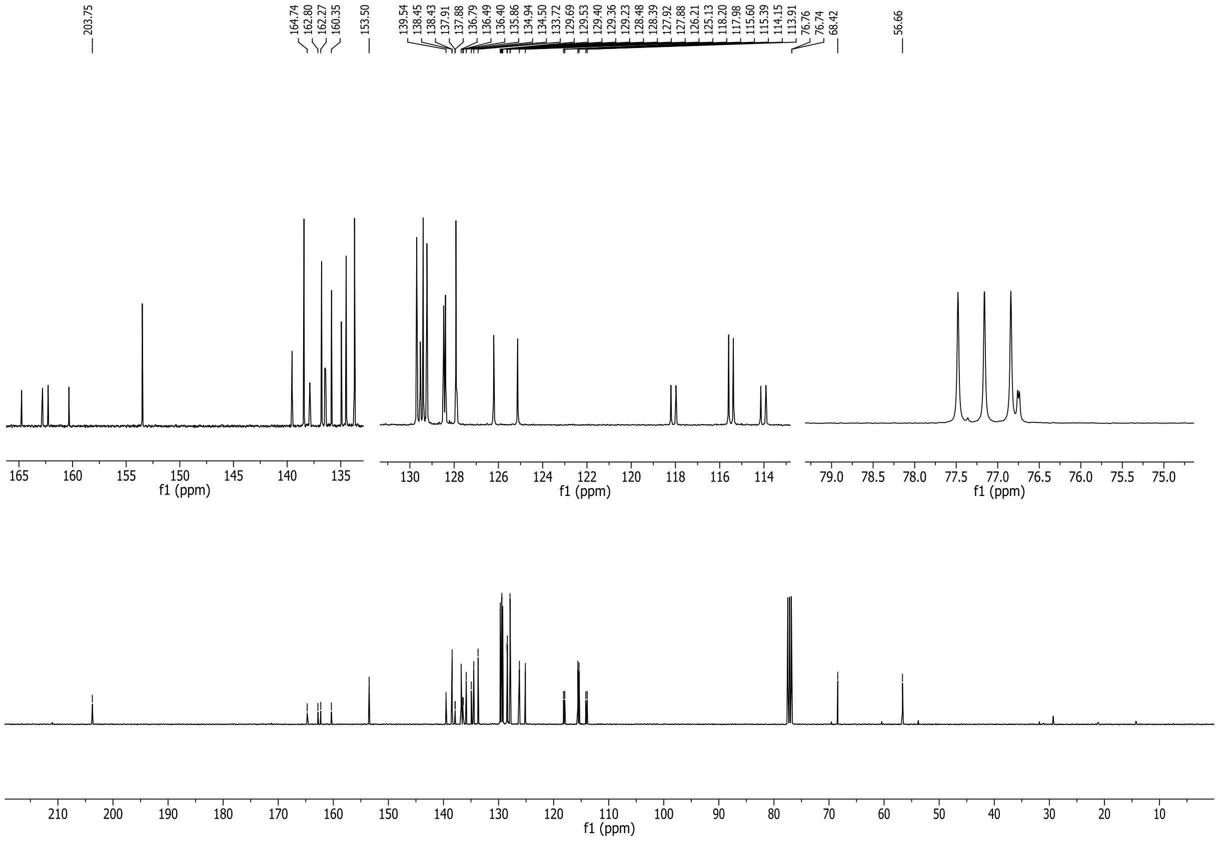

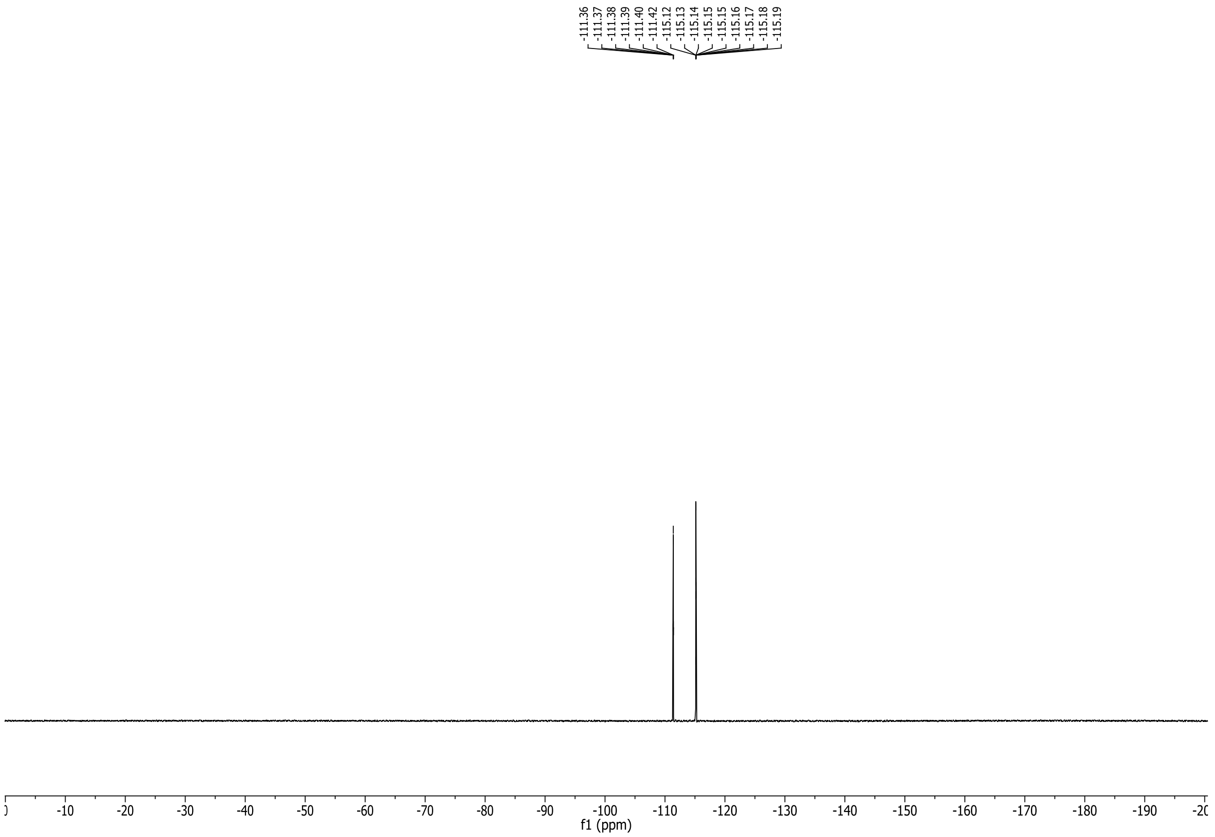

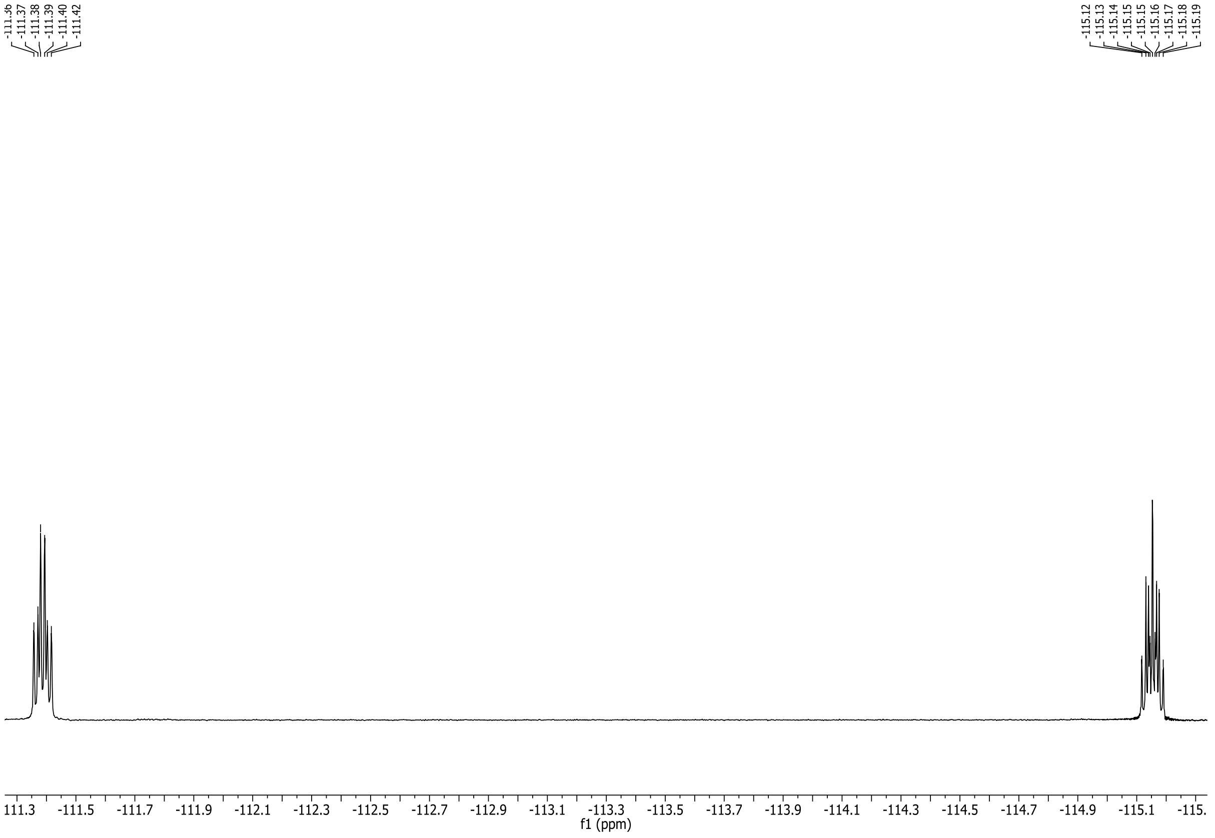

^{1}H and ^{13}C NMR data of compound **12l** (400, 125 MHz, $CDCl_3$)

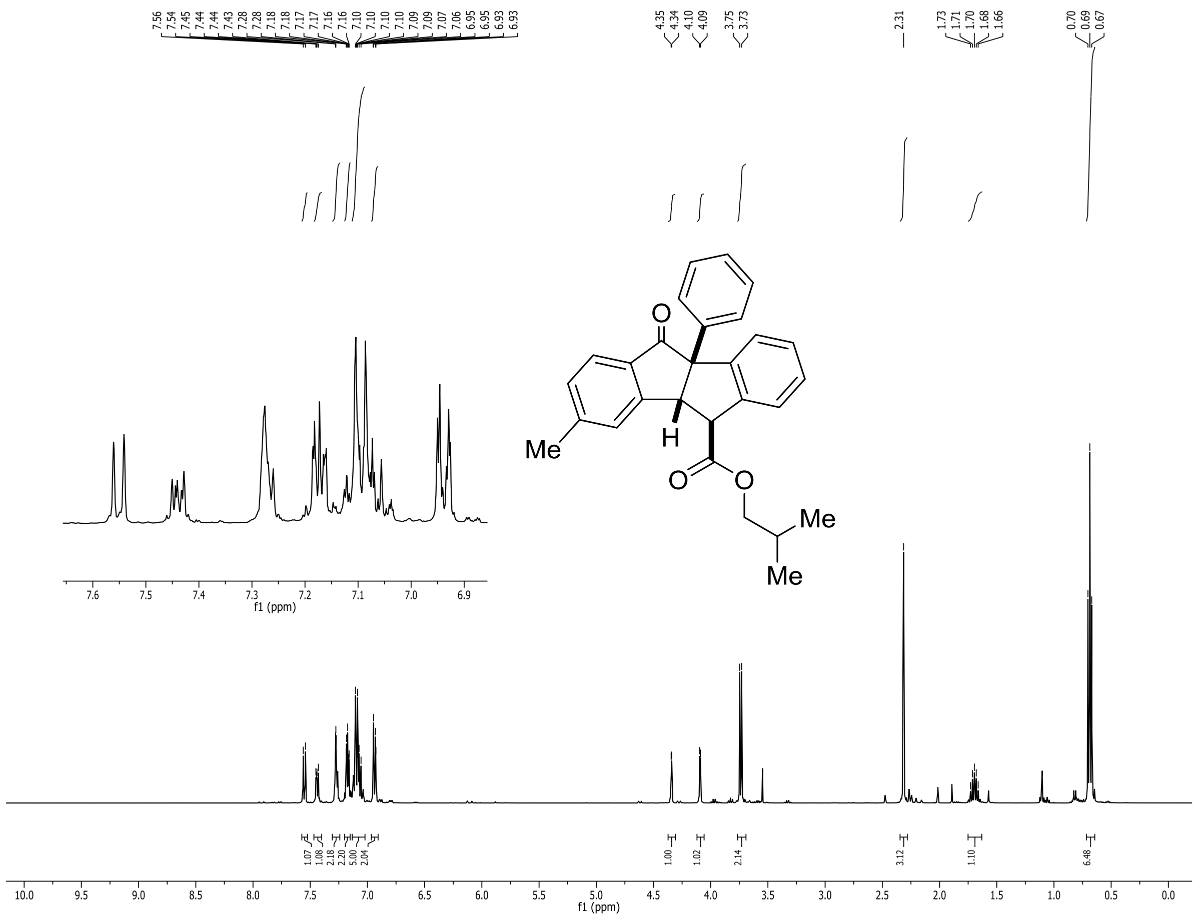
7.56 7.54 7.45 7.44 7.44 7.43 7.28 7.28 7.18 7.18 7.17 7.17 7.16 7.16 7.10 7.10 7.10 7.10 7.10 7.09 7.09 7.07 7.06 6.95 6.95 6.93 6.93
4.35 4.34 4.10 4.09 3.75 3.73
2.31
1.73 1.71 1.70 1.68 1.66
0.70 0.69 0.67
O
H
Me
O
O
Me
Me
1.07 1.08 2.18 2.20 5.00 2.04 1.00 1.02 2.14 3.12 1.10 6.48
f1 (ppm)

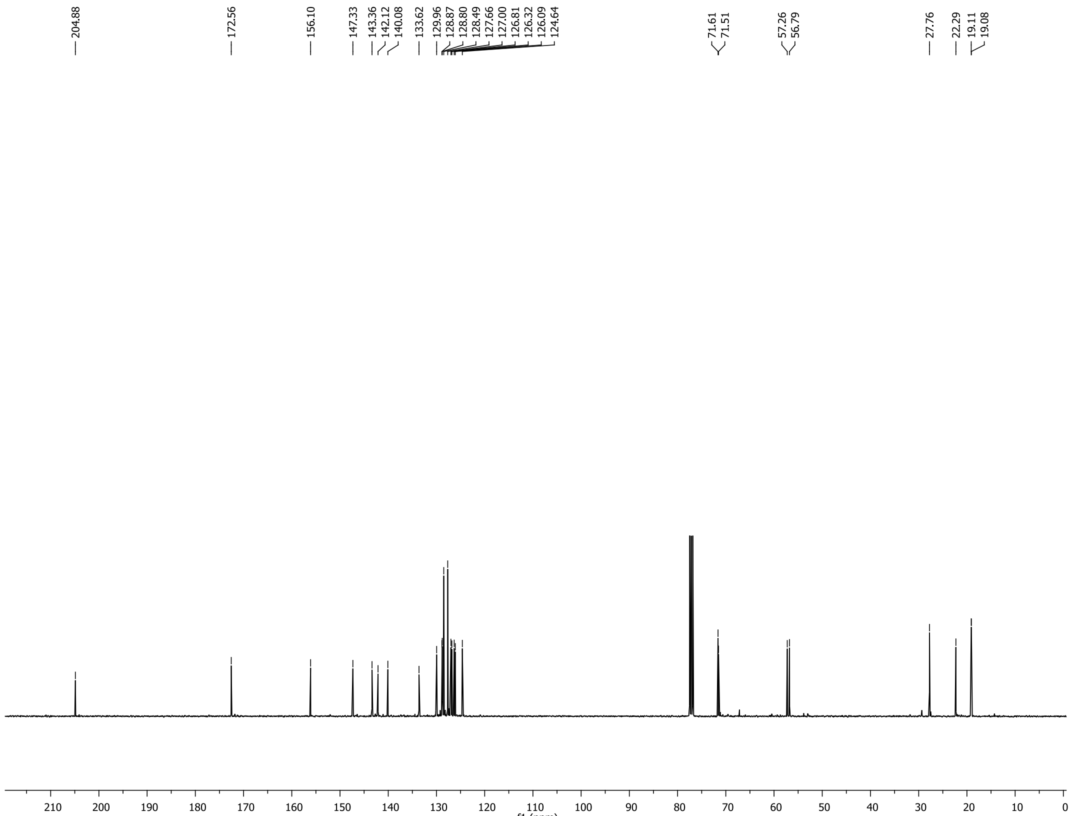
204.88
172.56
156.10
147.33 143.36 142.12 140.08 133.62 129.96 128.87 128.80 128.49 127.66 127.00 126.81 126.32 126.09 124.64
71.61 71.51
57.26 56.79
27.76 22.29 19.11 19.08
f1 (ppm)

^{1}H and ^{13}C NMR data of compound **13a** (400, 101 MHz, $CDCl_3$)

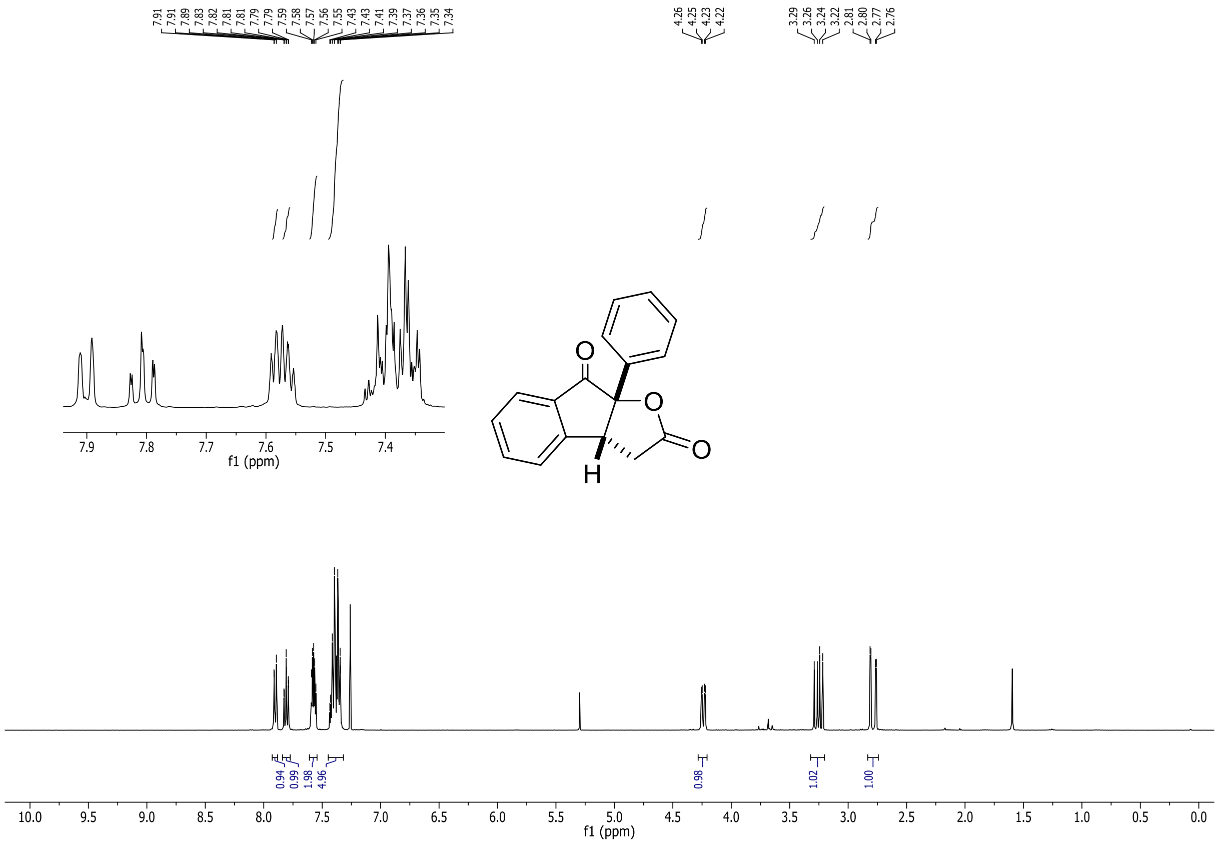

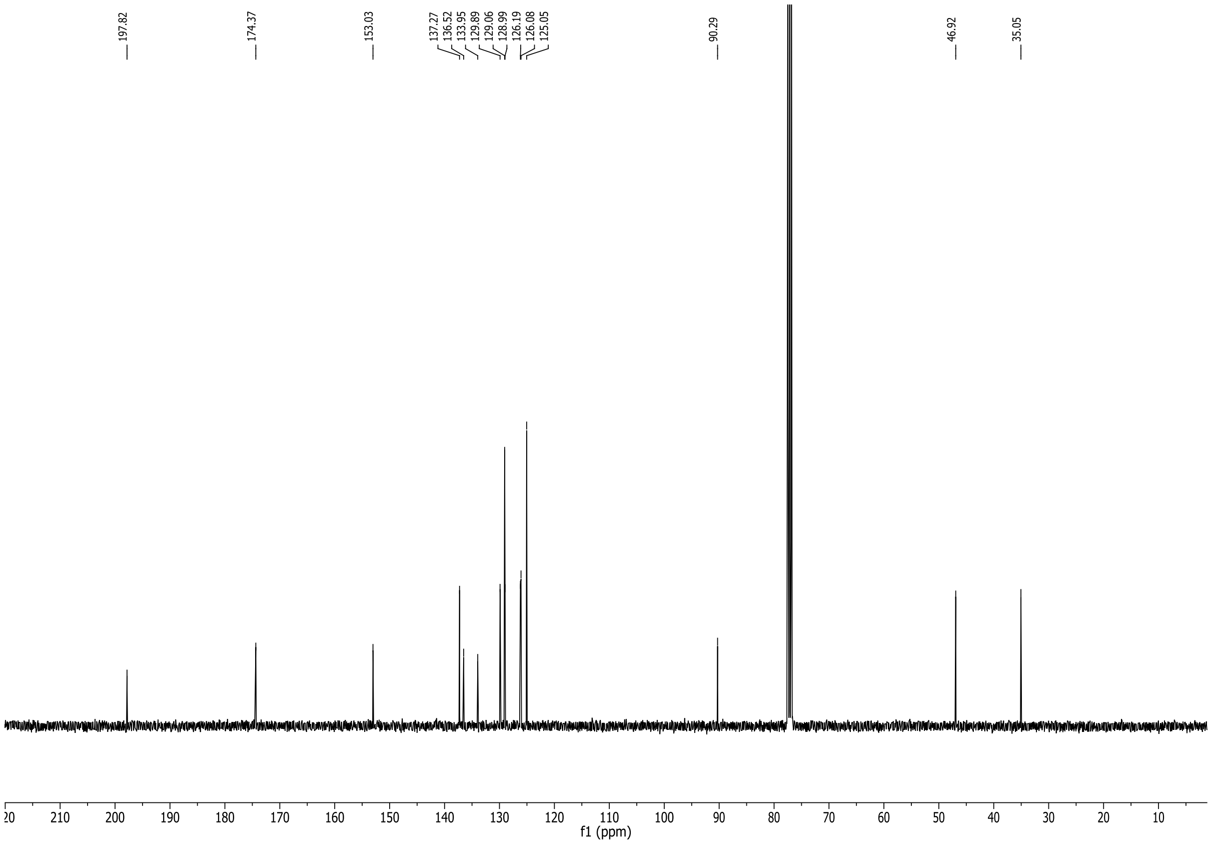

^{1}H and ^{13}C NMR data of compound **13b** (400, 101 MHz, $CDCl_3$)

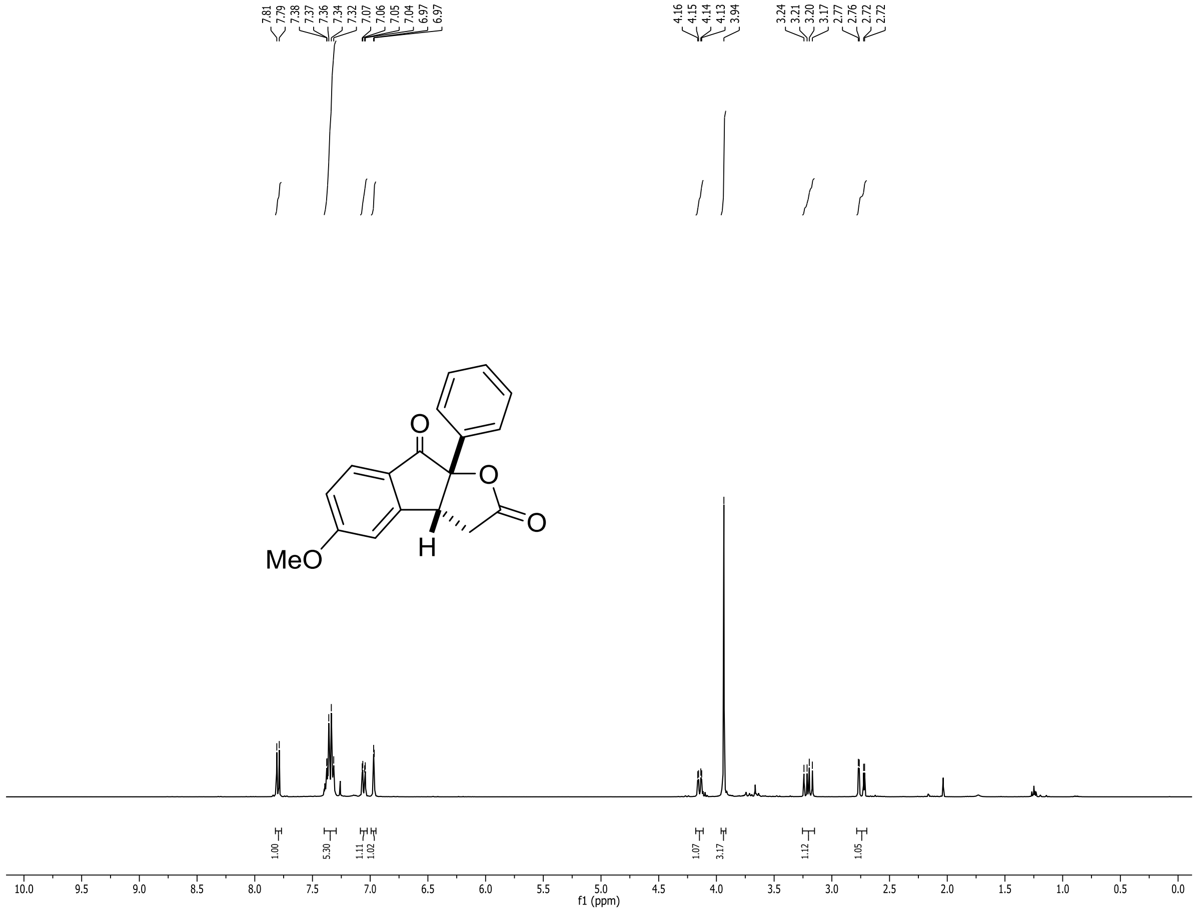

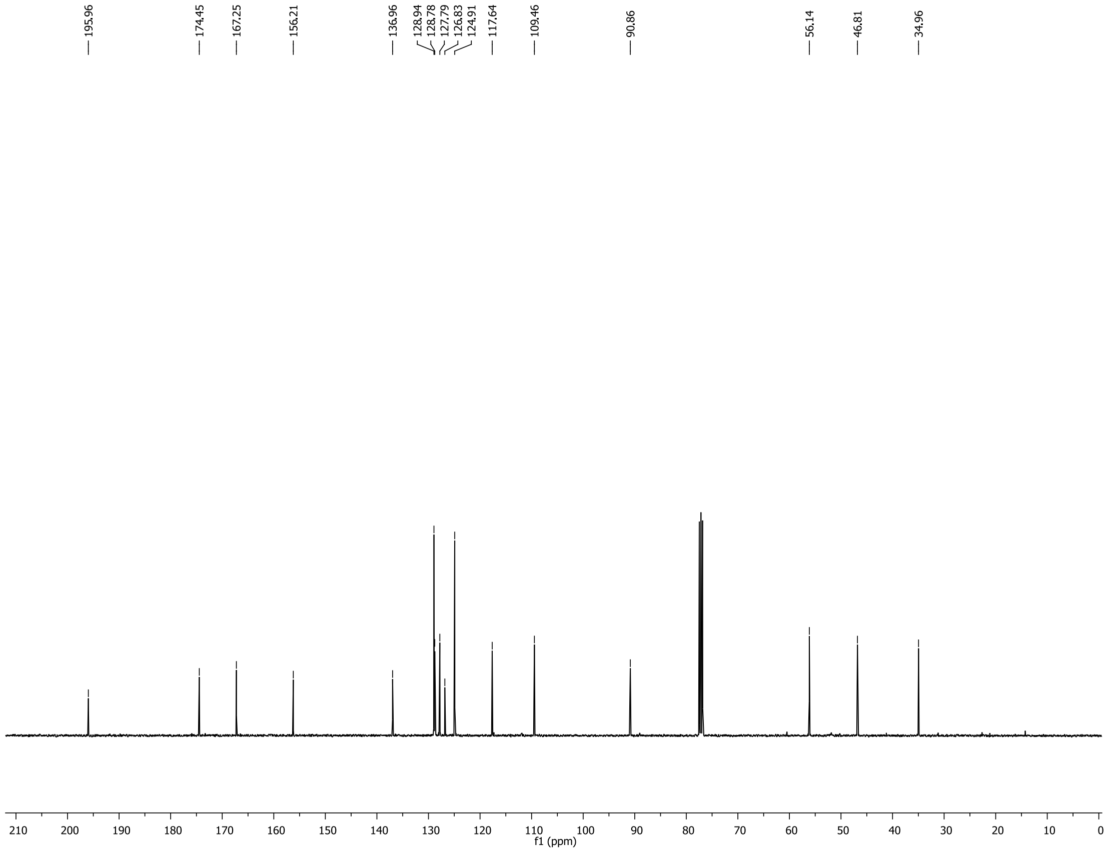

[1] H and [13] C NMR data of compound **13c** (400, 101 MHz, $CDCl_3$)

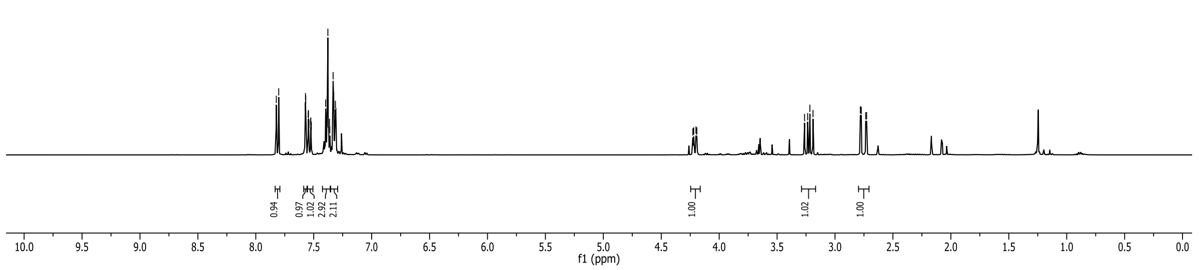

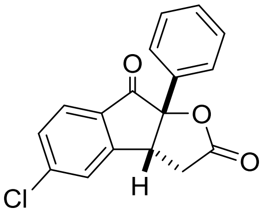

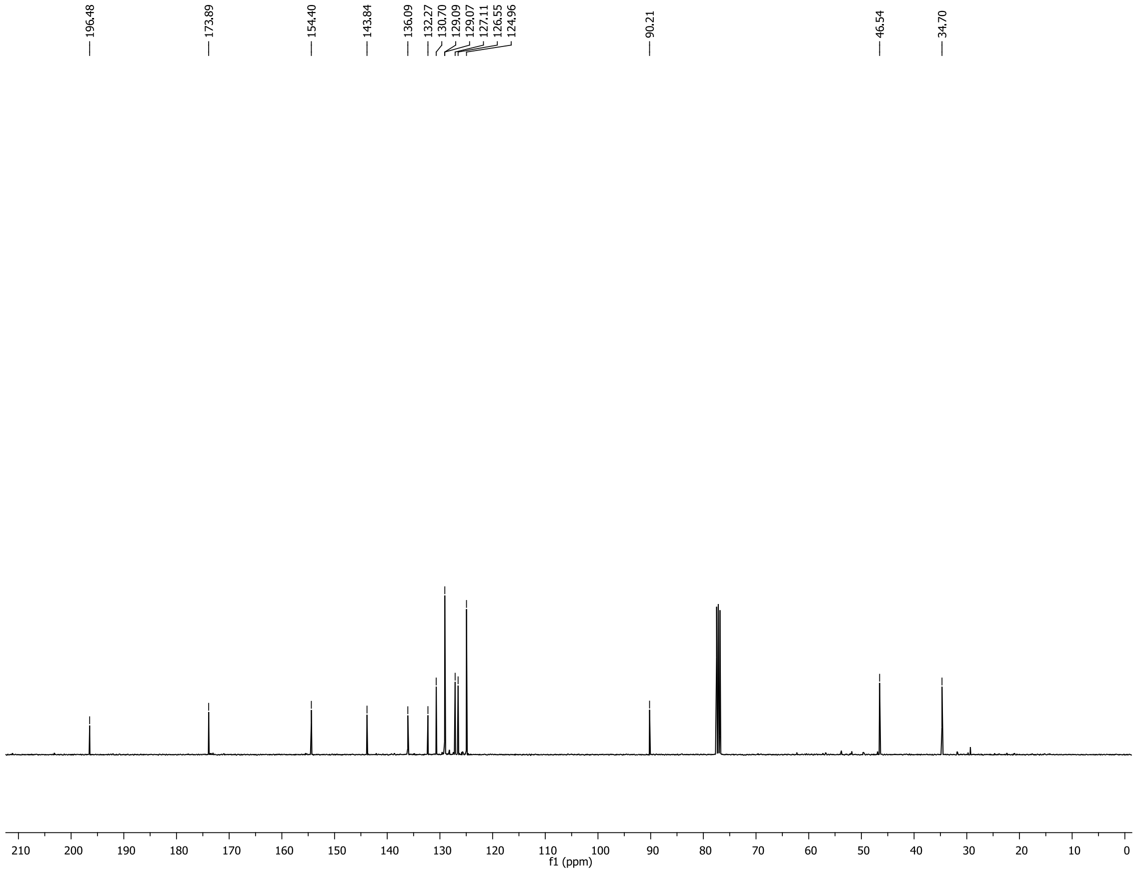

^{1}H and ^{13}C NMR data of compound **13d** (400, 101 MHz, $CDCl_3$)

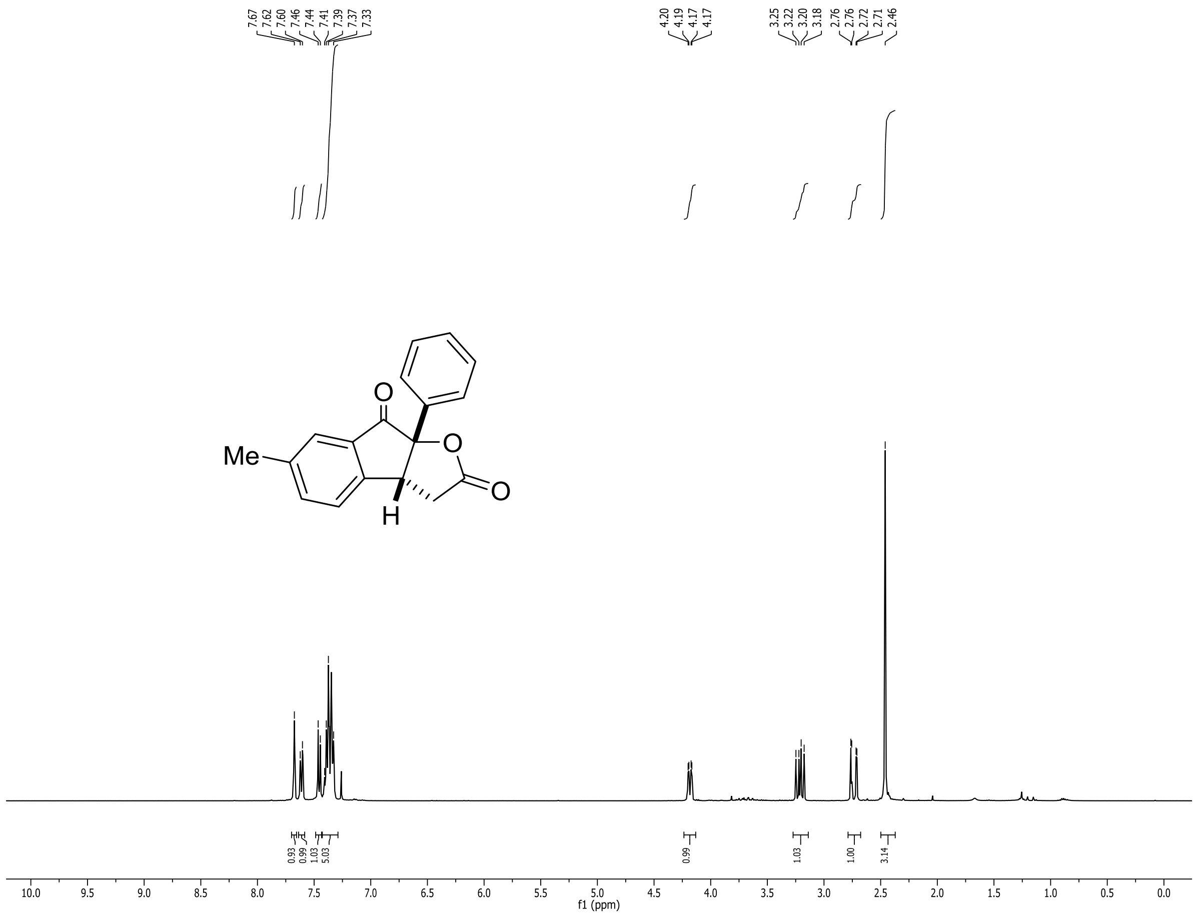

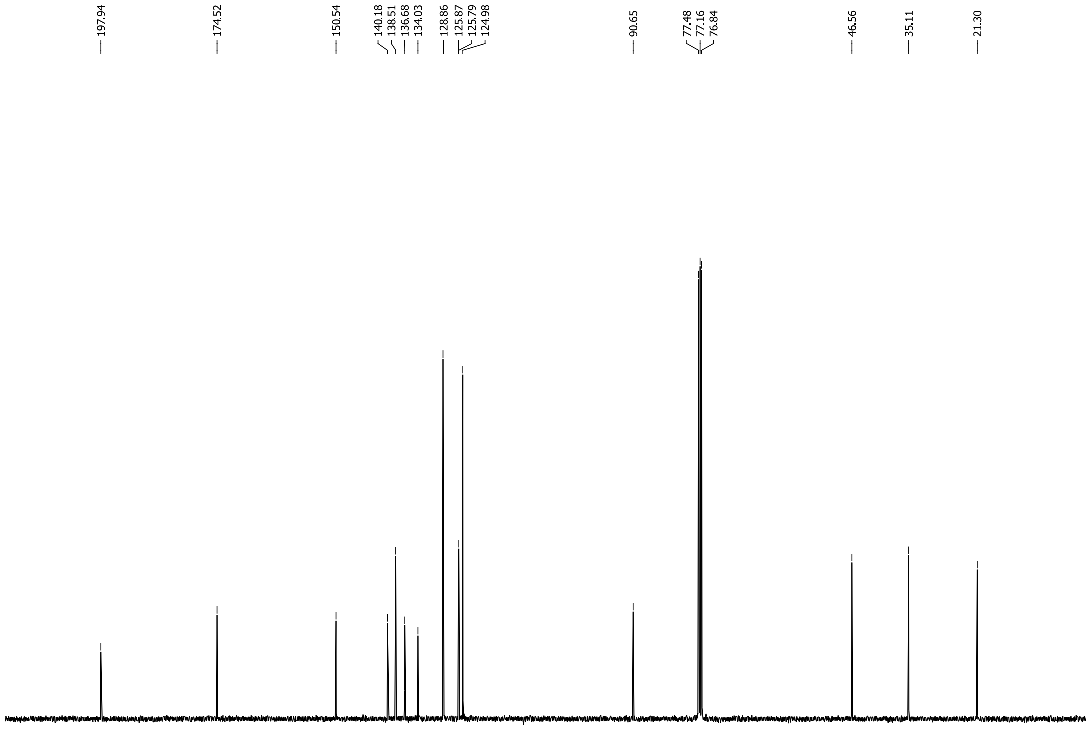

^{1}H, ^{13}C and ^{19}F NMR data of compound **13e** (400, 101, 470 MHz, $CDCl_3$)

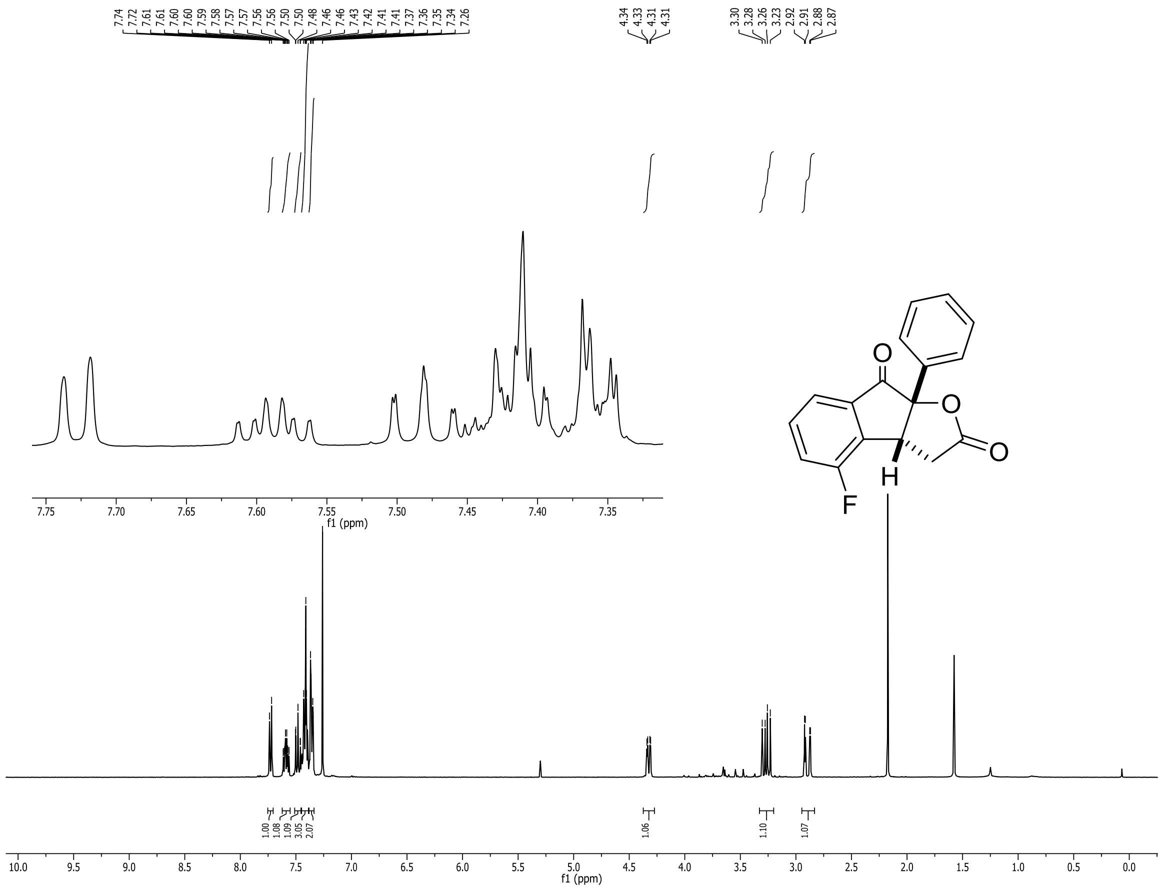

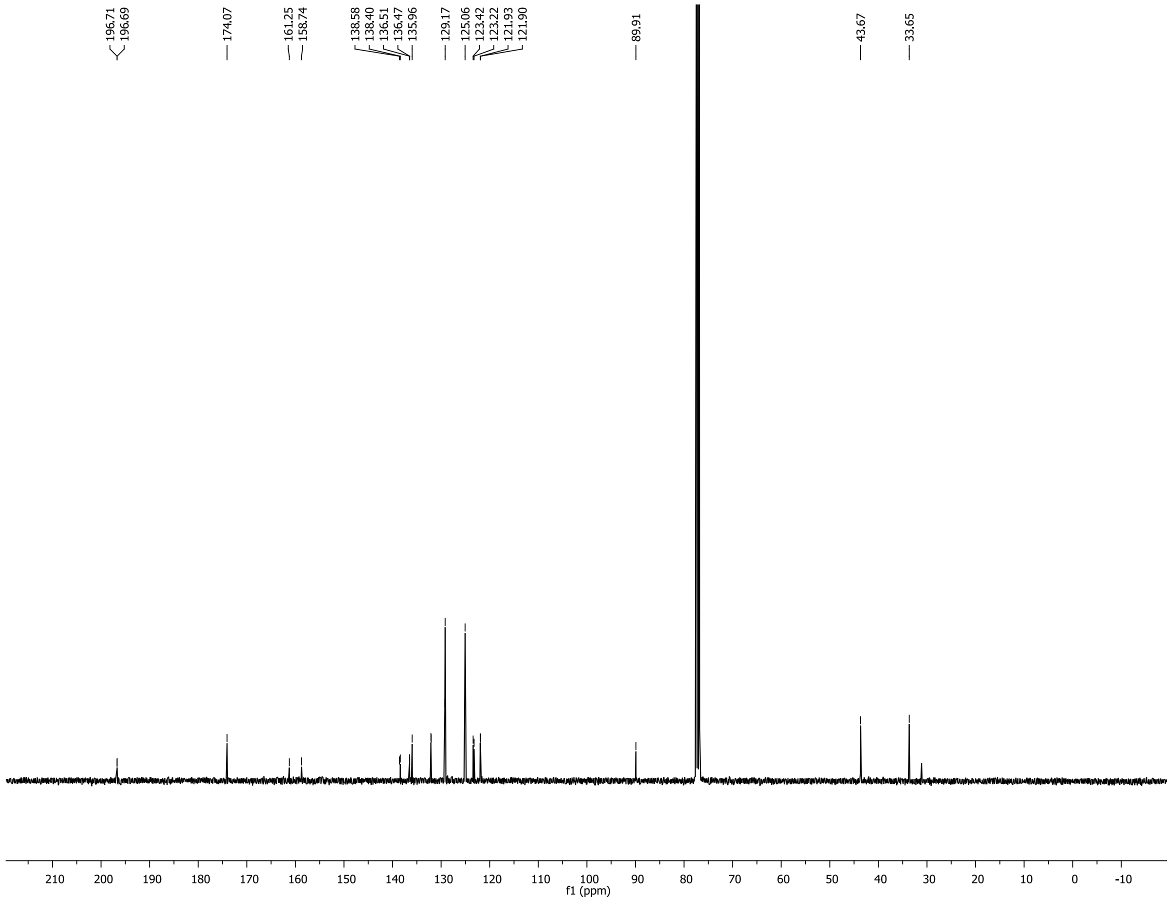

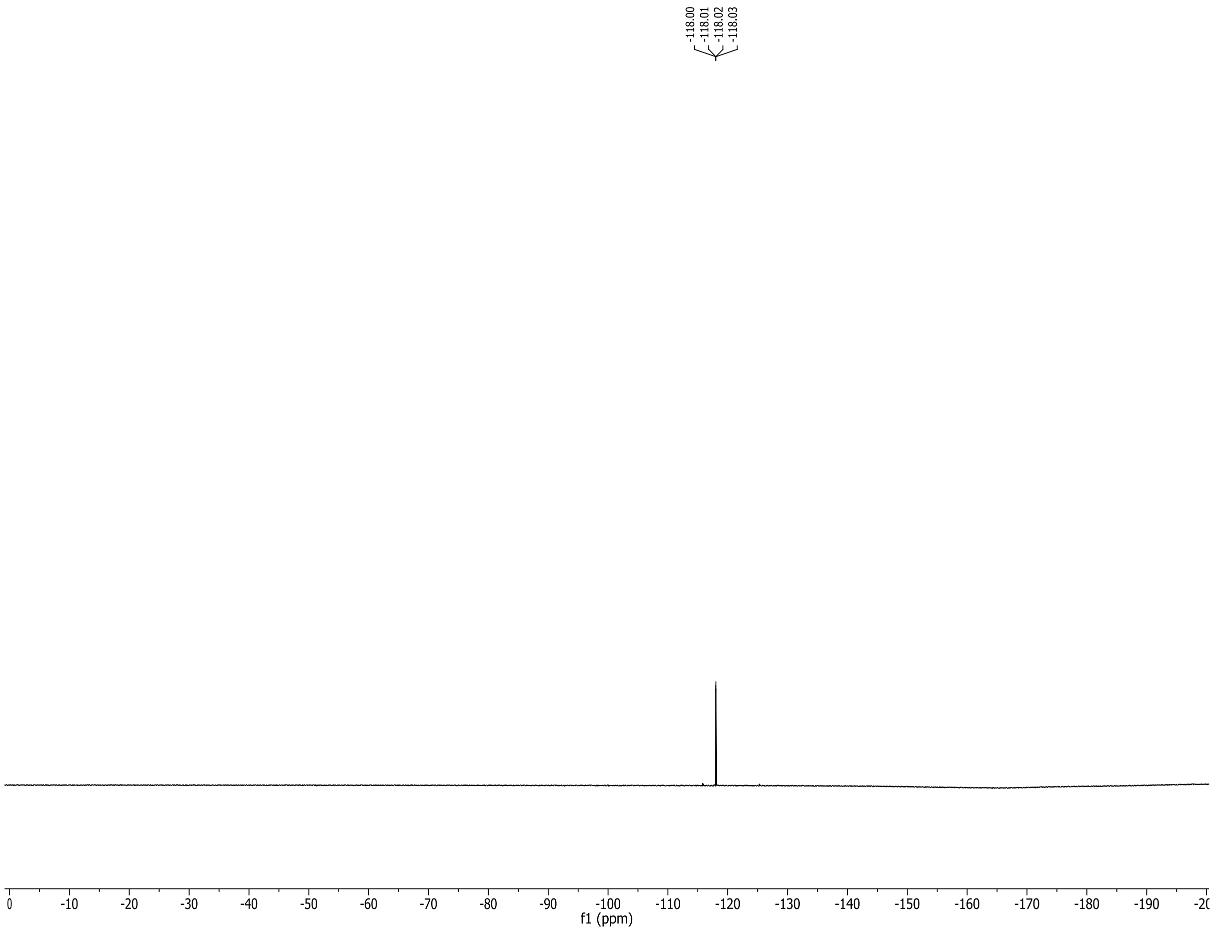
-118.00
-118.01
-118.02
-118.03
0
-10
-20
-30
-40
-50
-60
-70
-80
-90
-100
-110
-120
-130
-140
-150
-160
-170
-180
-190
f1 (ppm)

^{1}H and ^{13}C NMR data of compound **13f** (400, 101 MHz, $CDCl_3$)

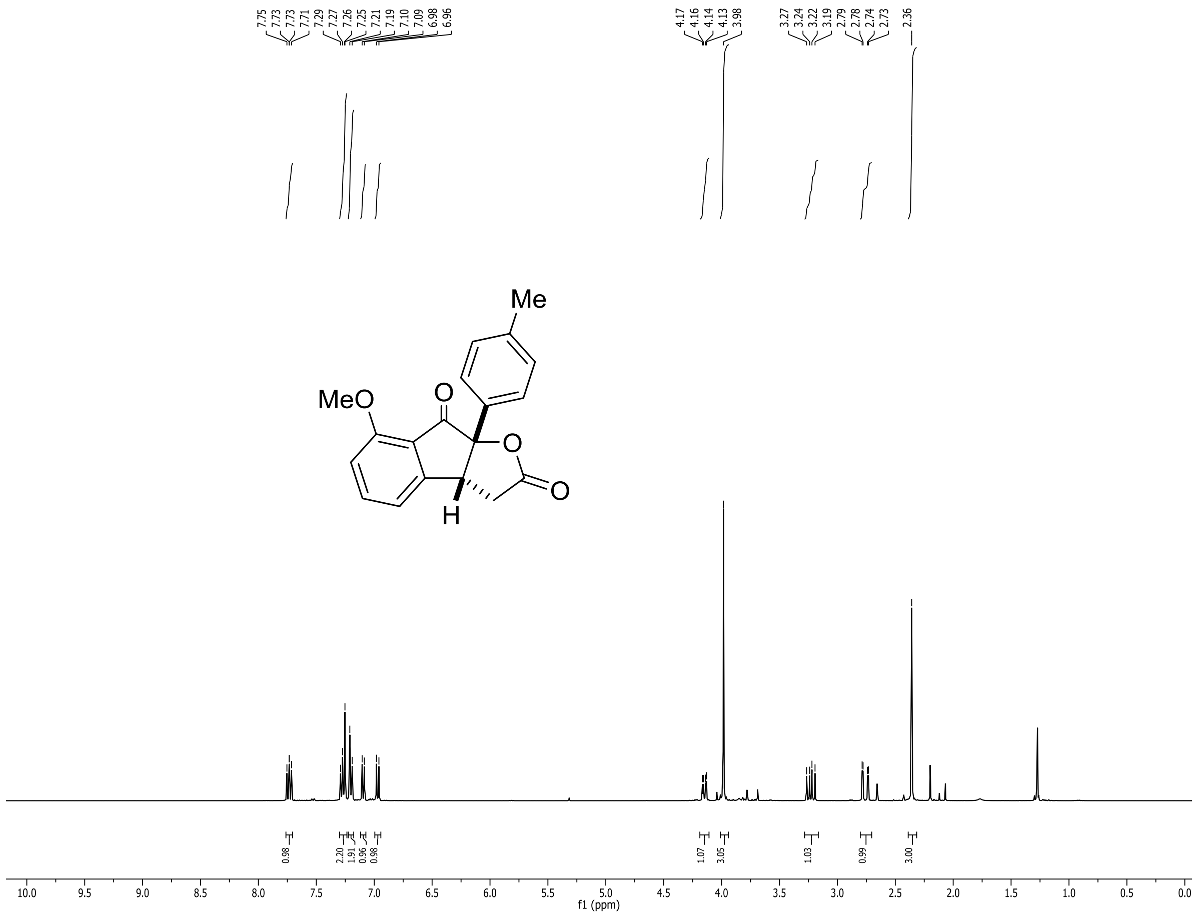

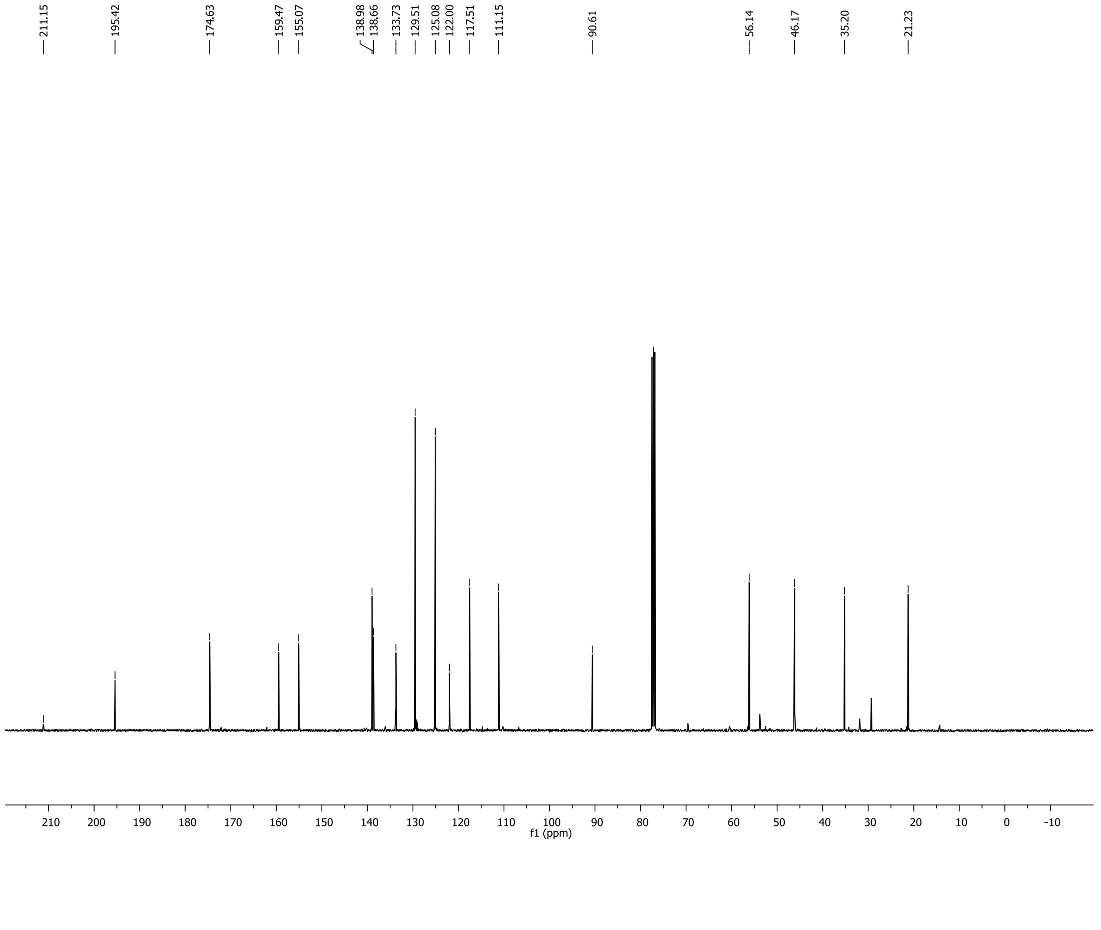

^{1}H, ^{13}C and ^{19}F NMR data of compound **13g** (500, 125, 470 MHz, $CDCl_3$)

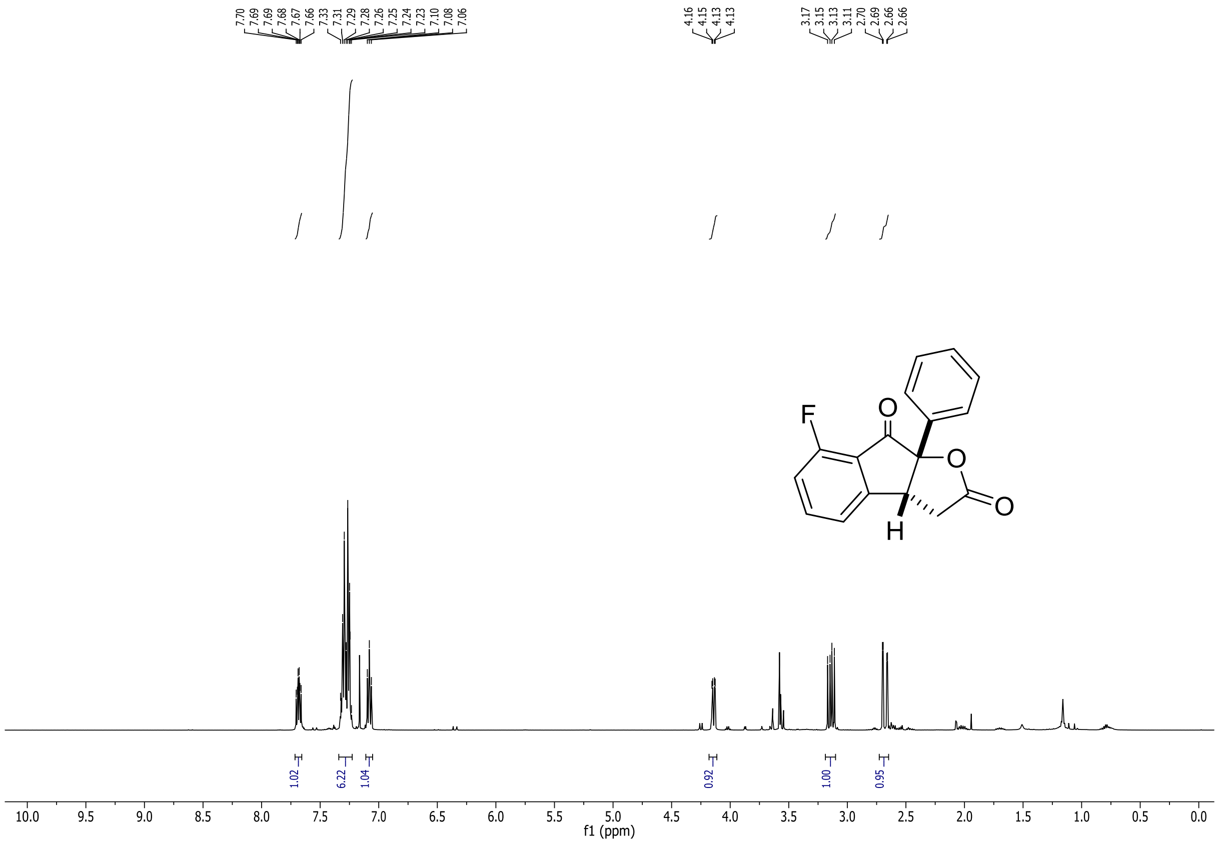

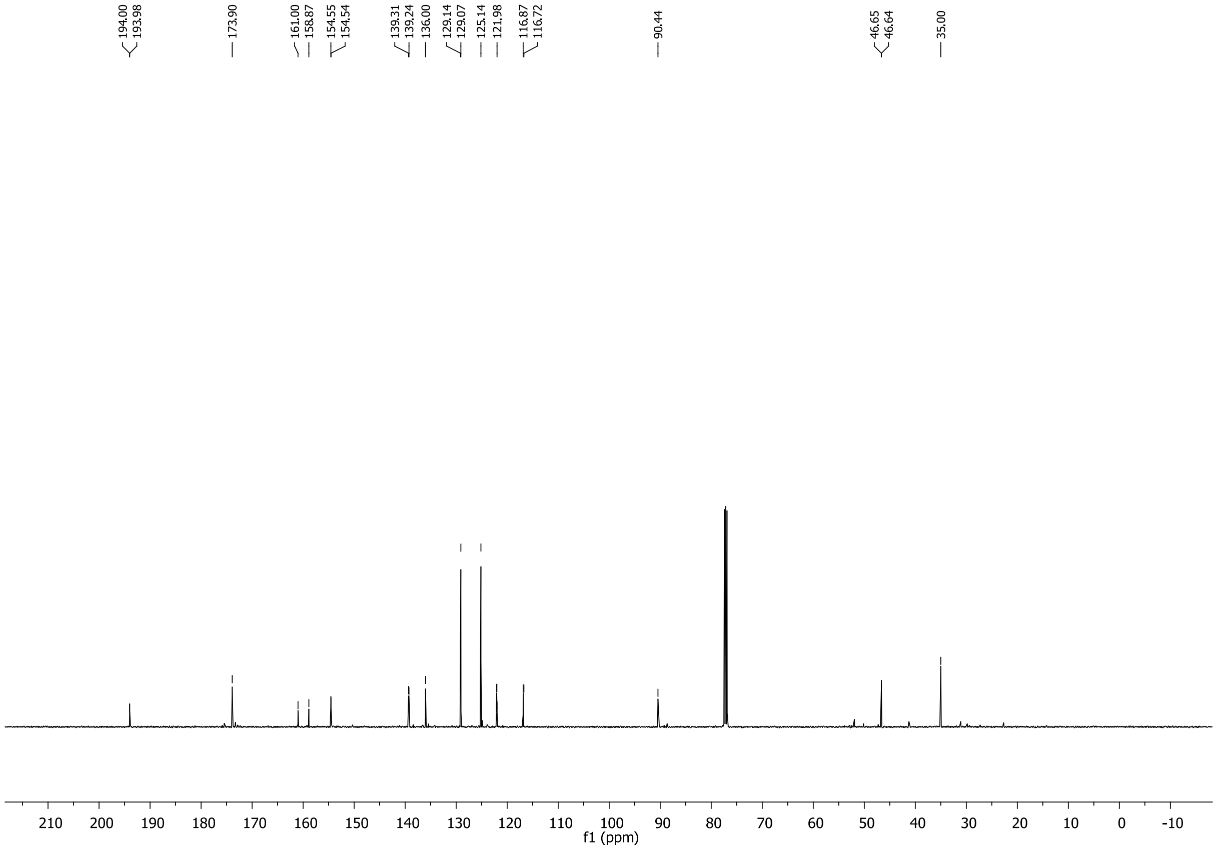

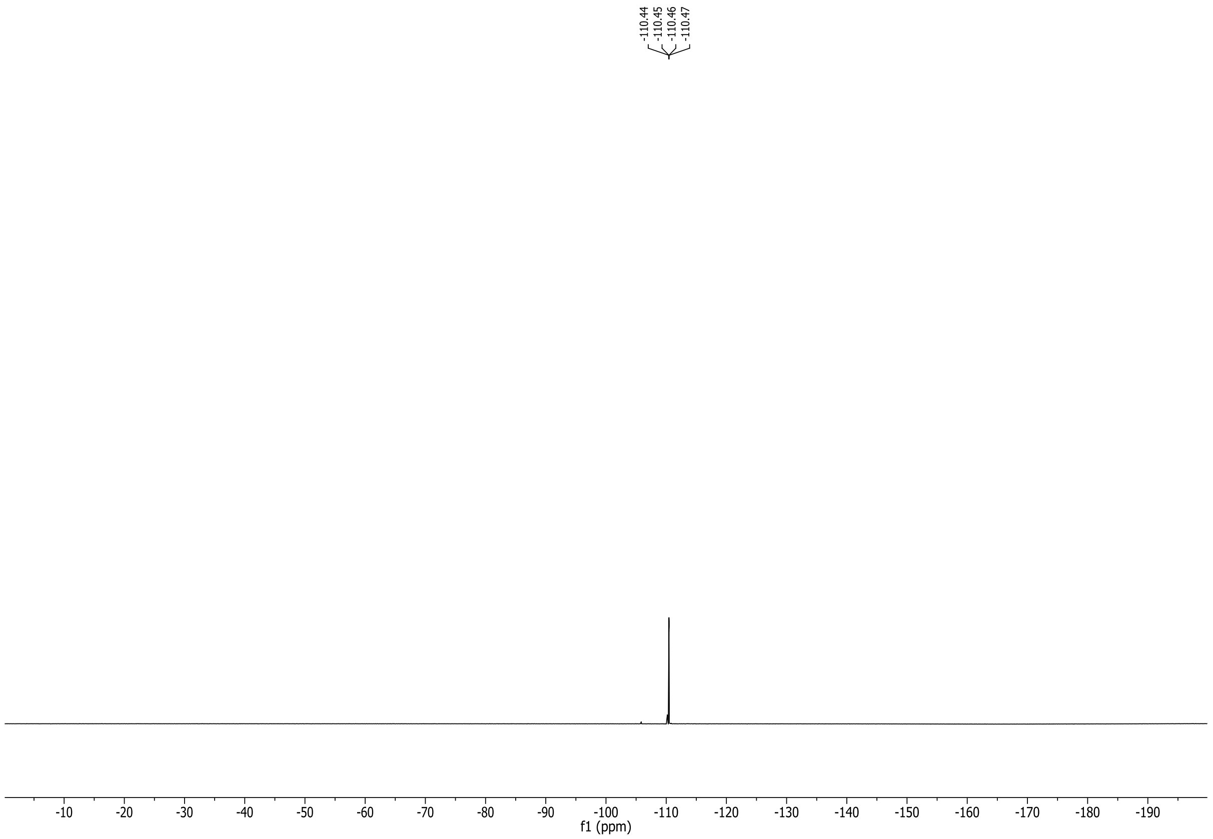
-110.44
-110.45
-110.46
-110.47
-10
-20
-30
-40
-50
-60
-70
-80
-90
-100
-110
-120
-130
-140
-150
-160
-170
-180
-190
f1 (ppm)

^{1}H, ^{13}C and ^{19}F NMR data of compound **13h** (400, 101, 376 MHz, $CDCl_3$)

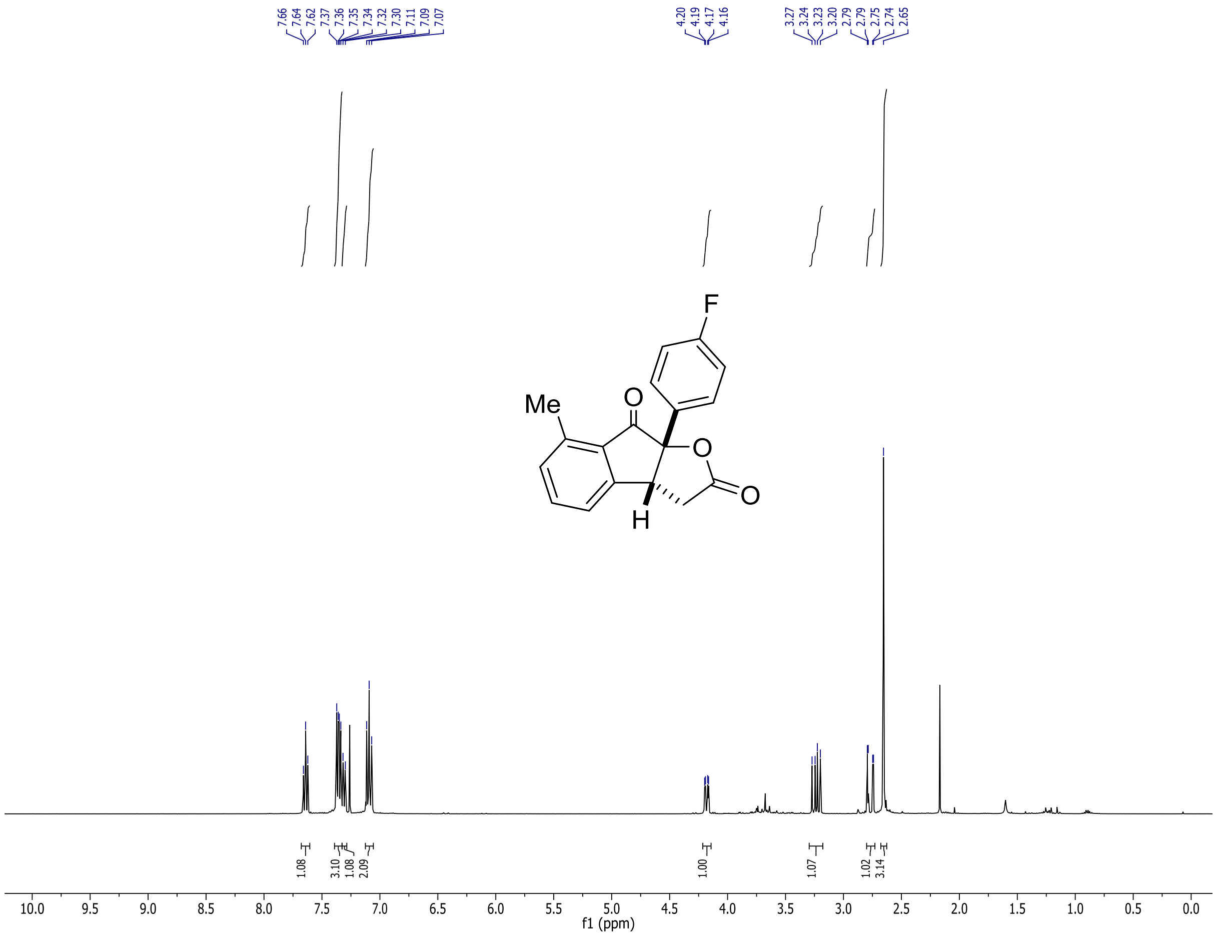

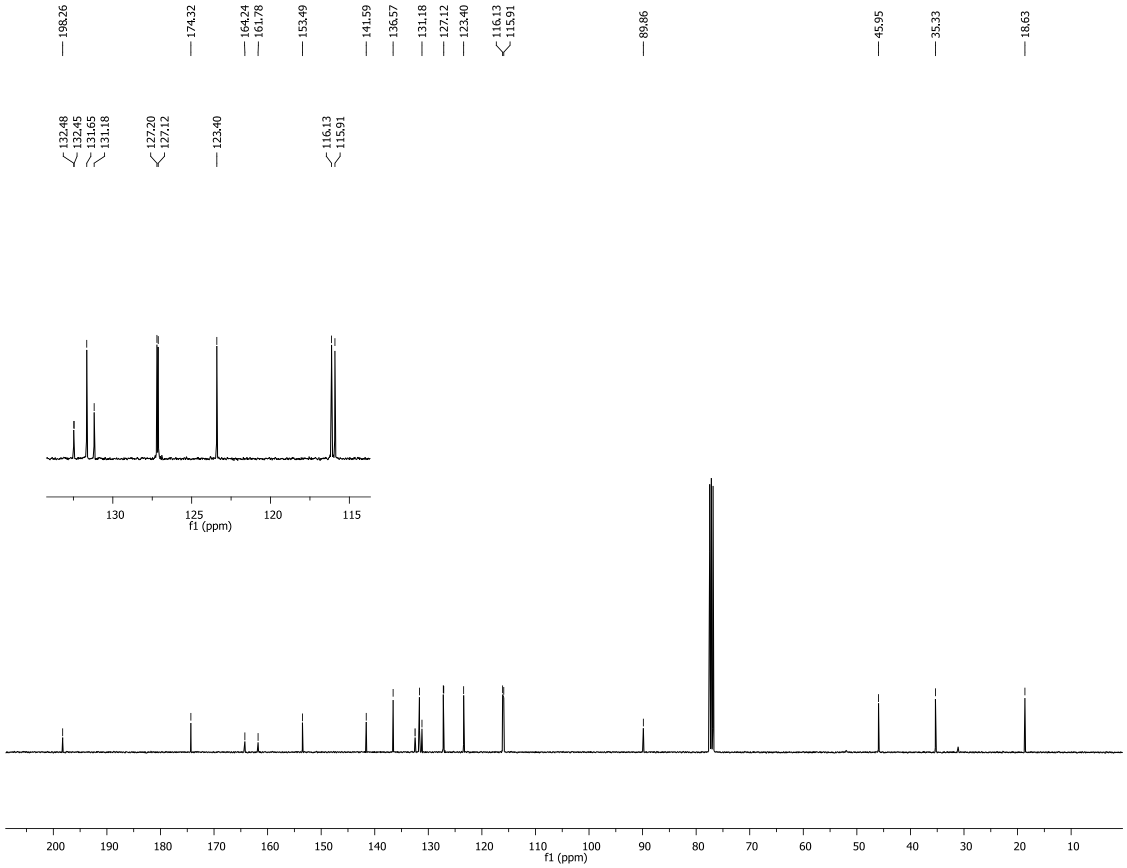

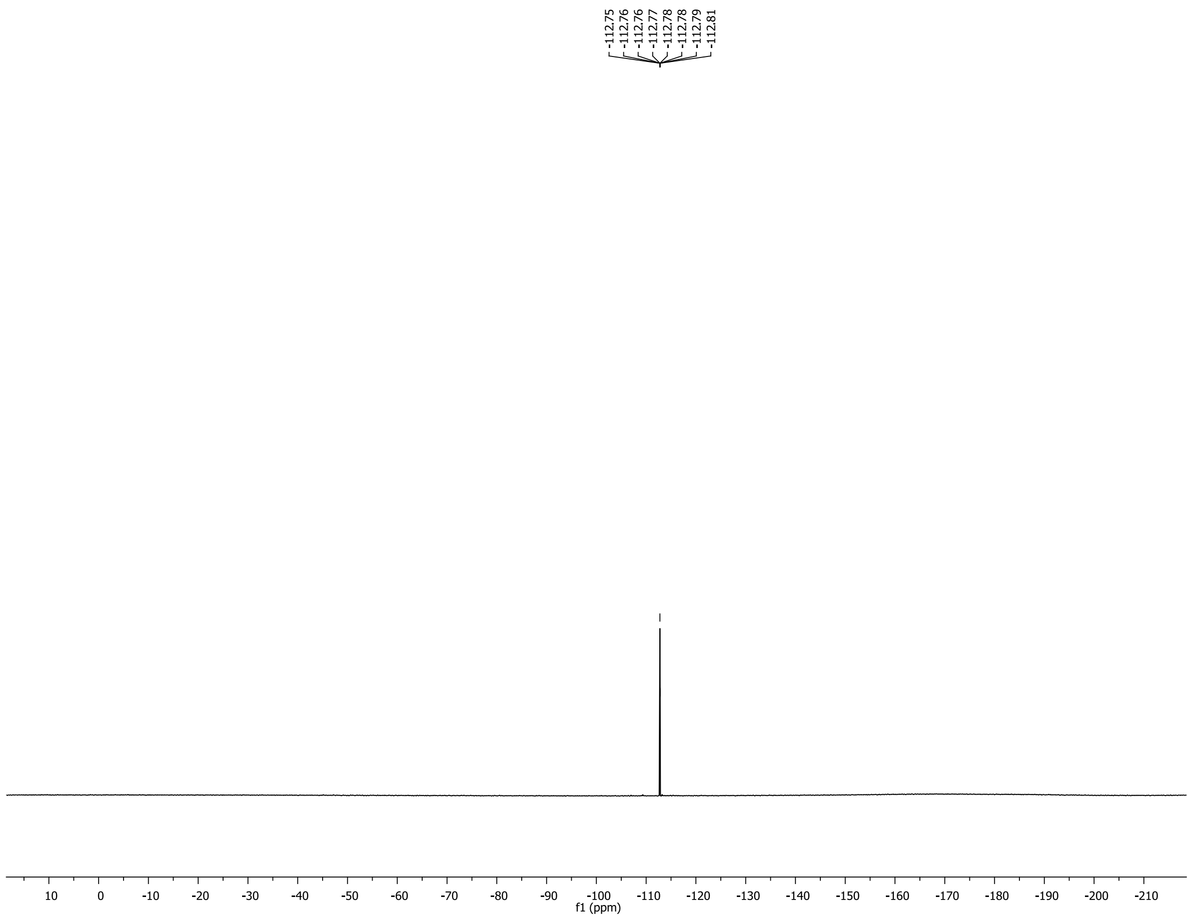
-112.75
-112.76
-112.76
-112.77
-112.78
-112.78
-112.79
-112.81
10
0
-10
-20
-30
-40
-50
-60
-70
-80
-90
-100
-110
-120
-130
-140
-150
-160
-170
-180
-190
-200
-210
f1 (ppm)

^{1}H and ^{13}C NMR data of compound **13i** (500, 125 MHz, $CDCl_3$)

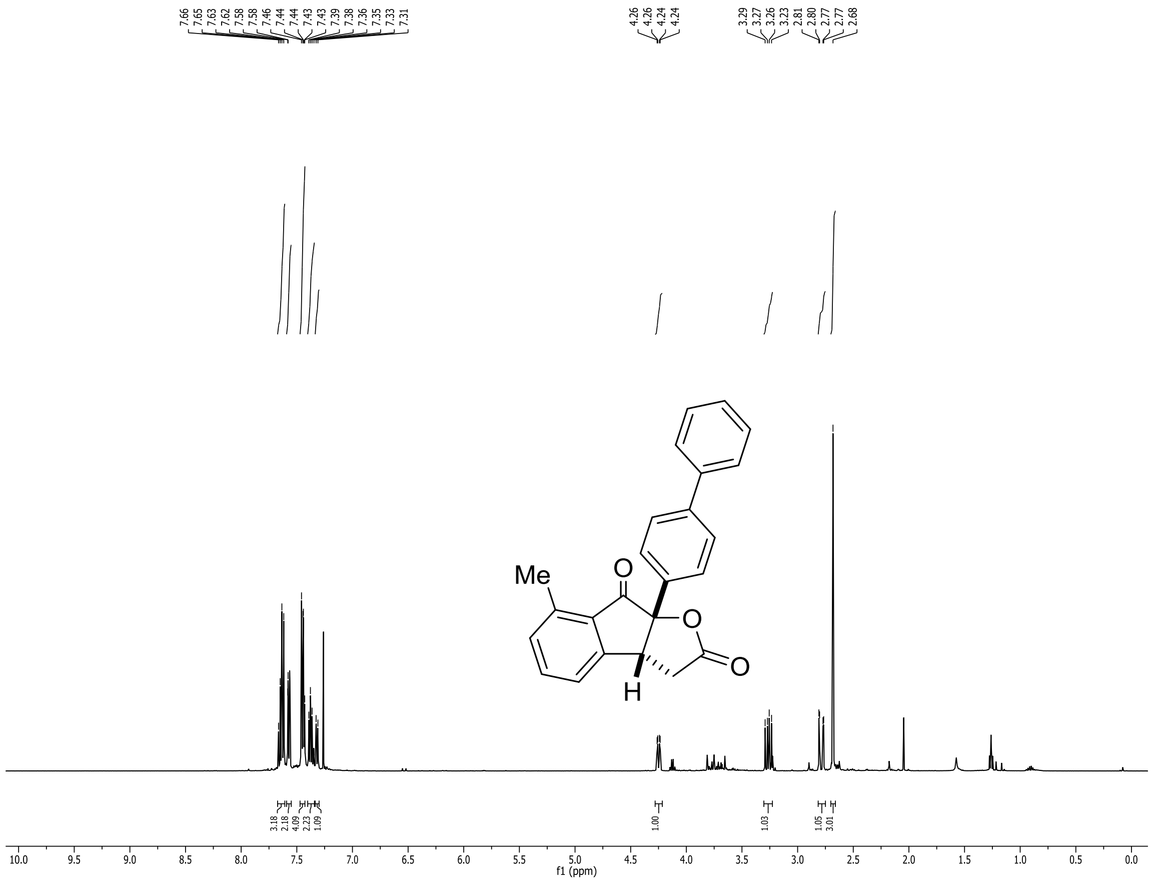

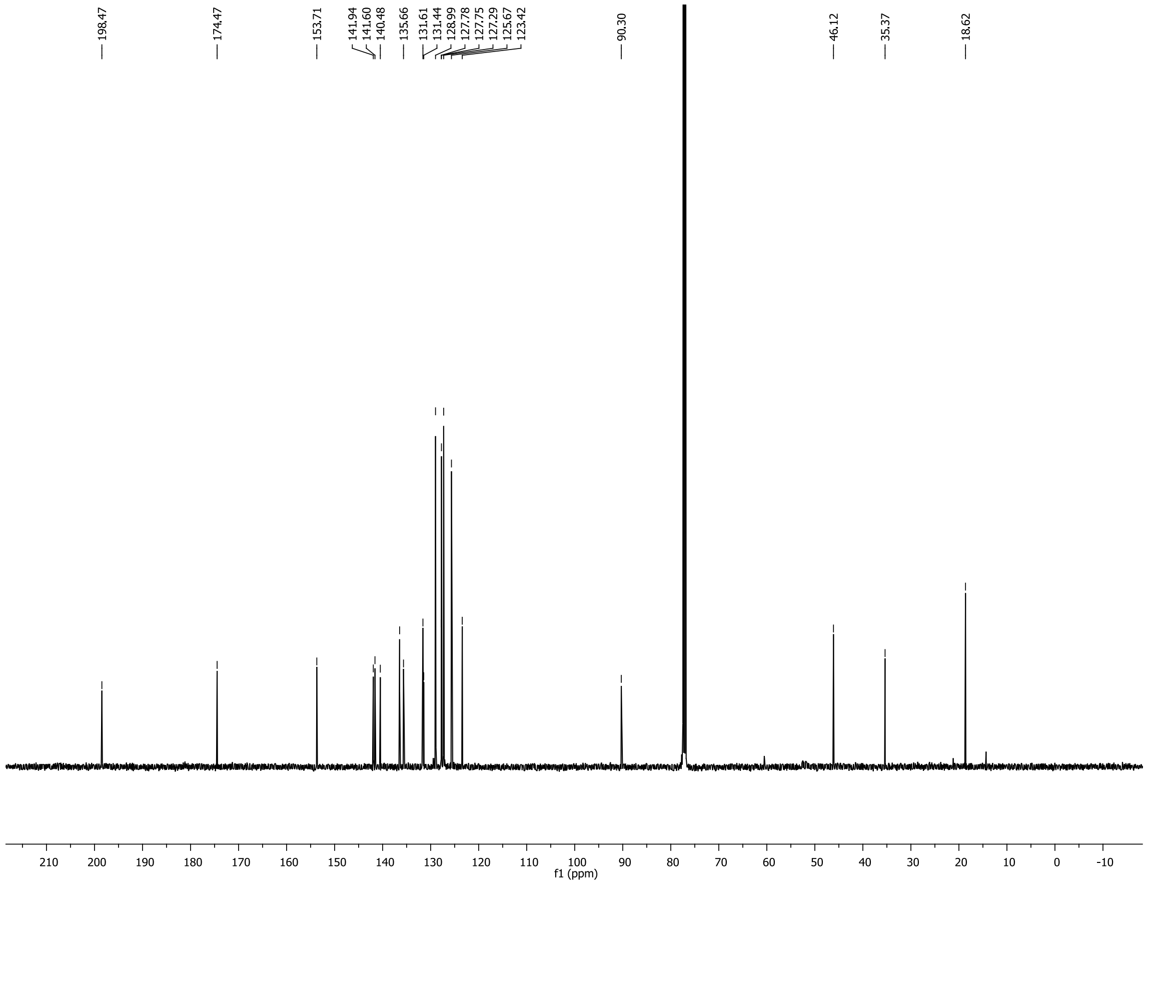

^{1}H and ^{13}C NMR data of compound **13j** (500, 125 MHz, $CDCl_3$)

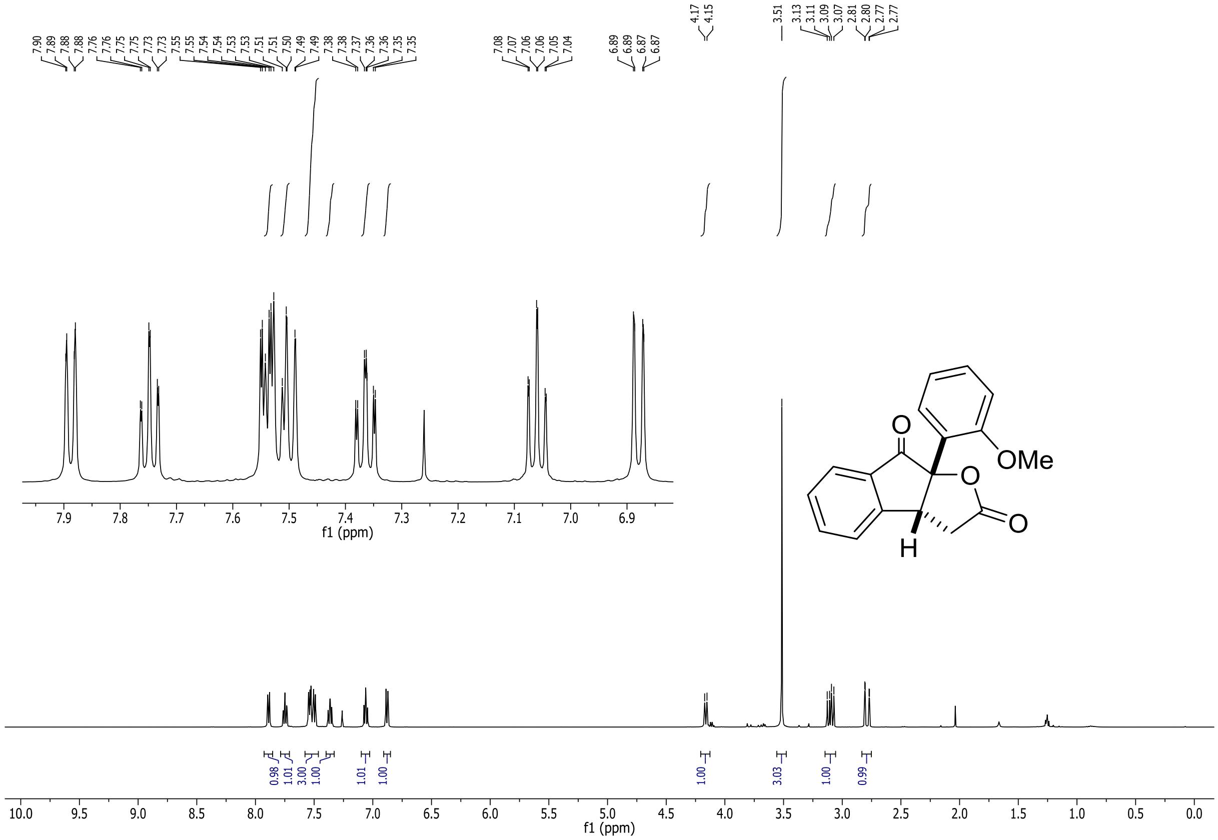

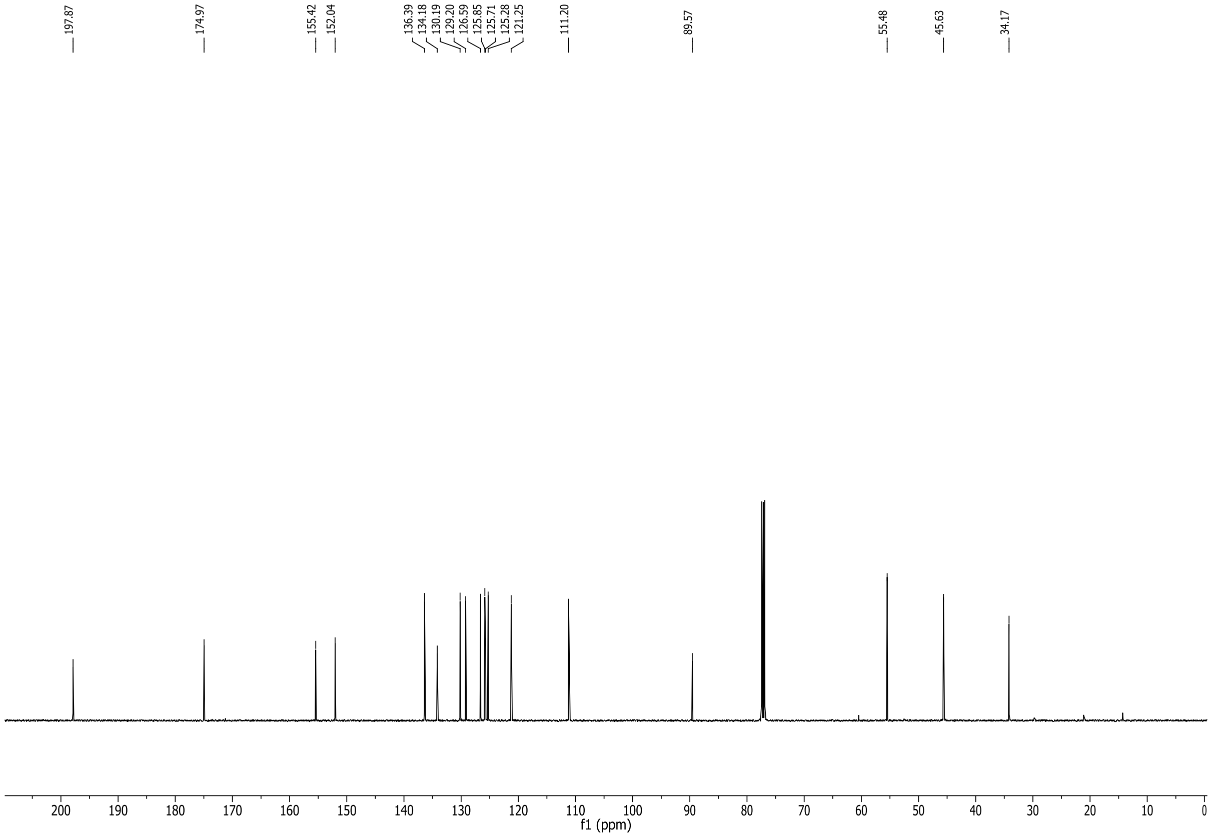

^{1}H, ^{13}C and ^{19}F NMR data of compound **13k** (400, 101, 376 MHz, $CDCl_3$)

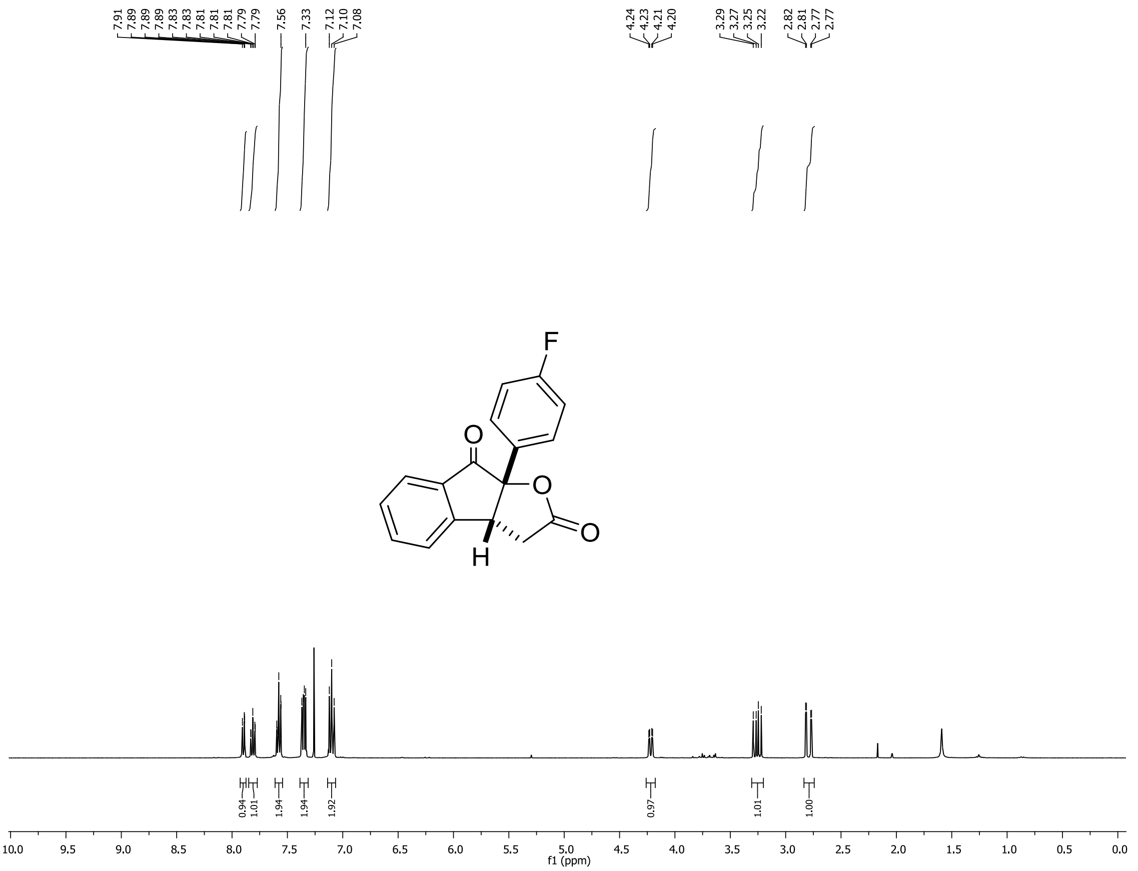

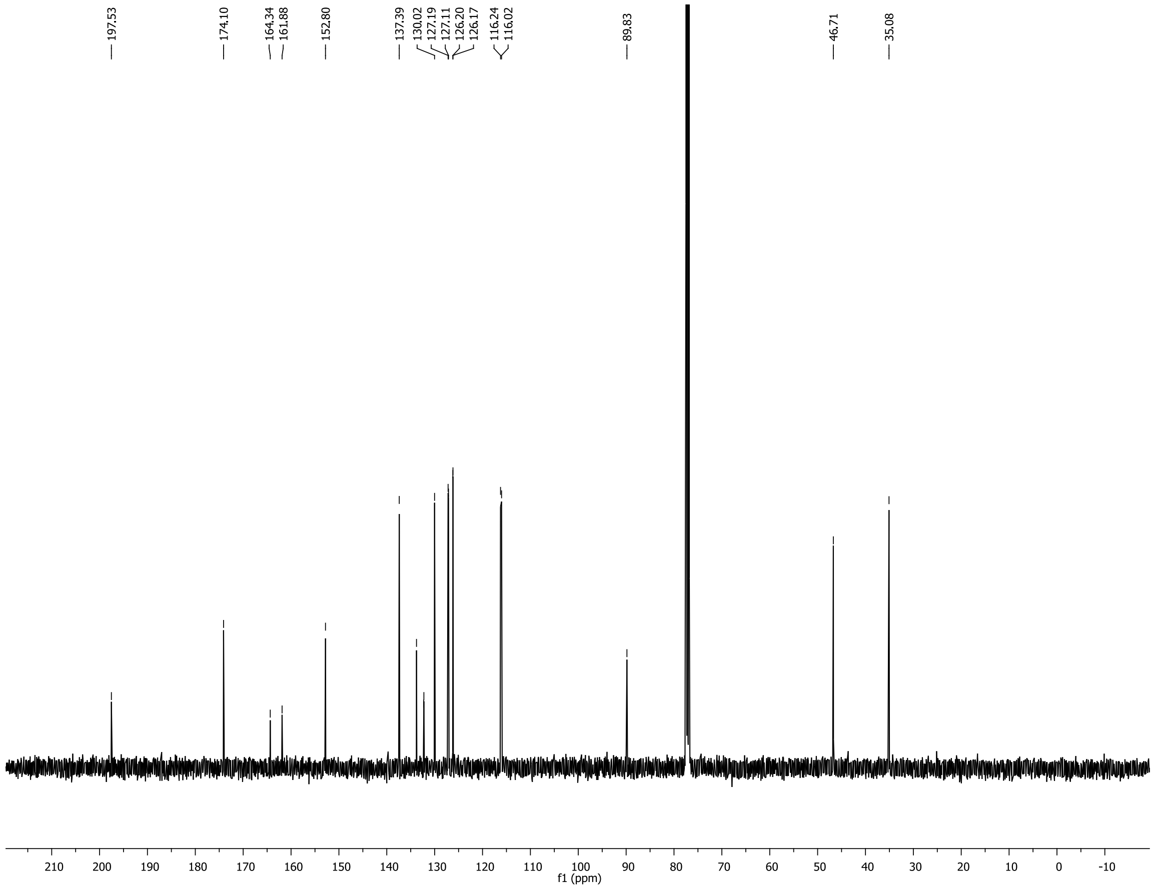

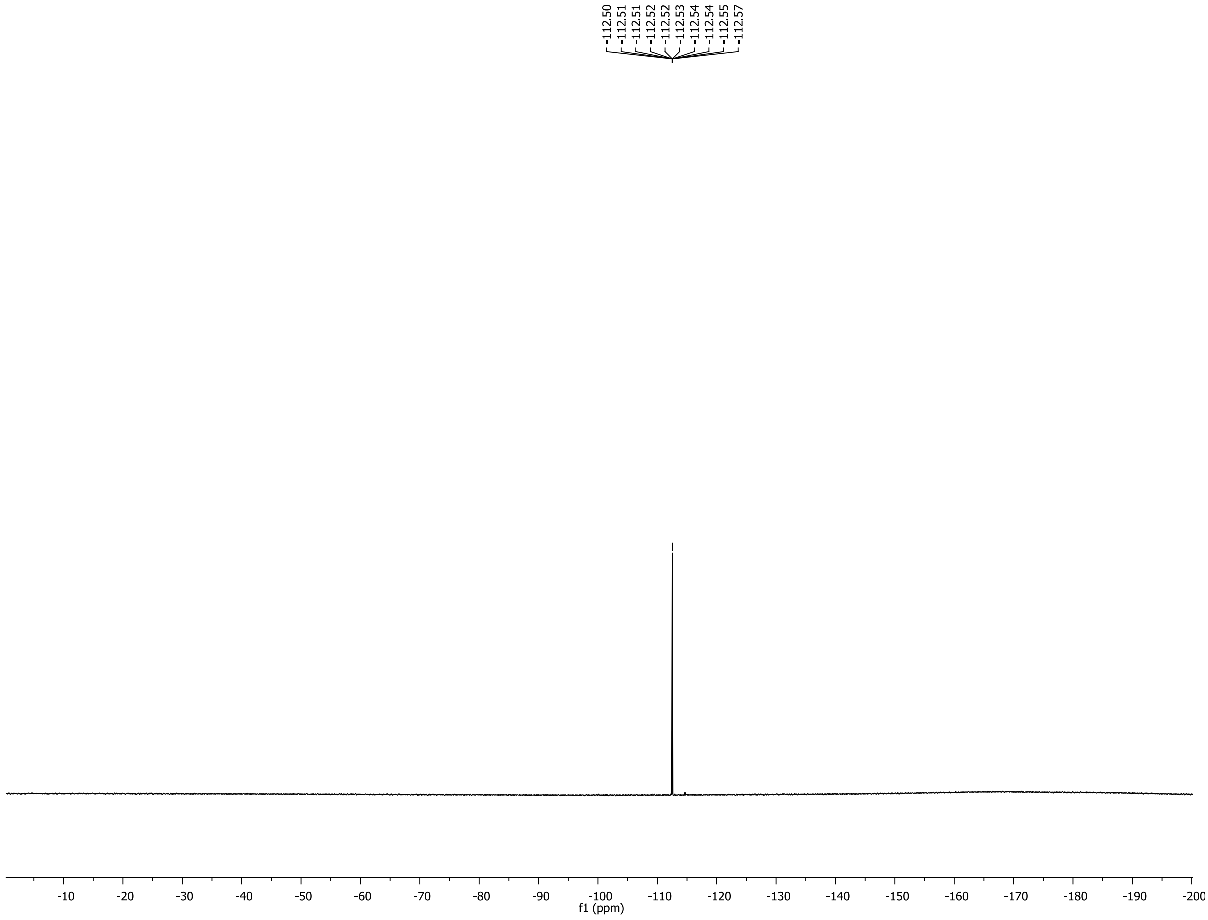
-112.50
-112.51
-112.51
-112.52
-112.52
-112.53
-112.54
-112.54
-112.55
-112.57
-10
-20
-30
-40
-50
-60
-70
-80
-90
-100
-110
-120
-130
-140
-150
-160
-170
-180
-190
-200
f1 (ppm)

^{1}H and ^{13}C NMR data of compound **13l** (400, 101 MHz, $CDCl_3$)

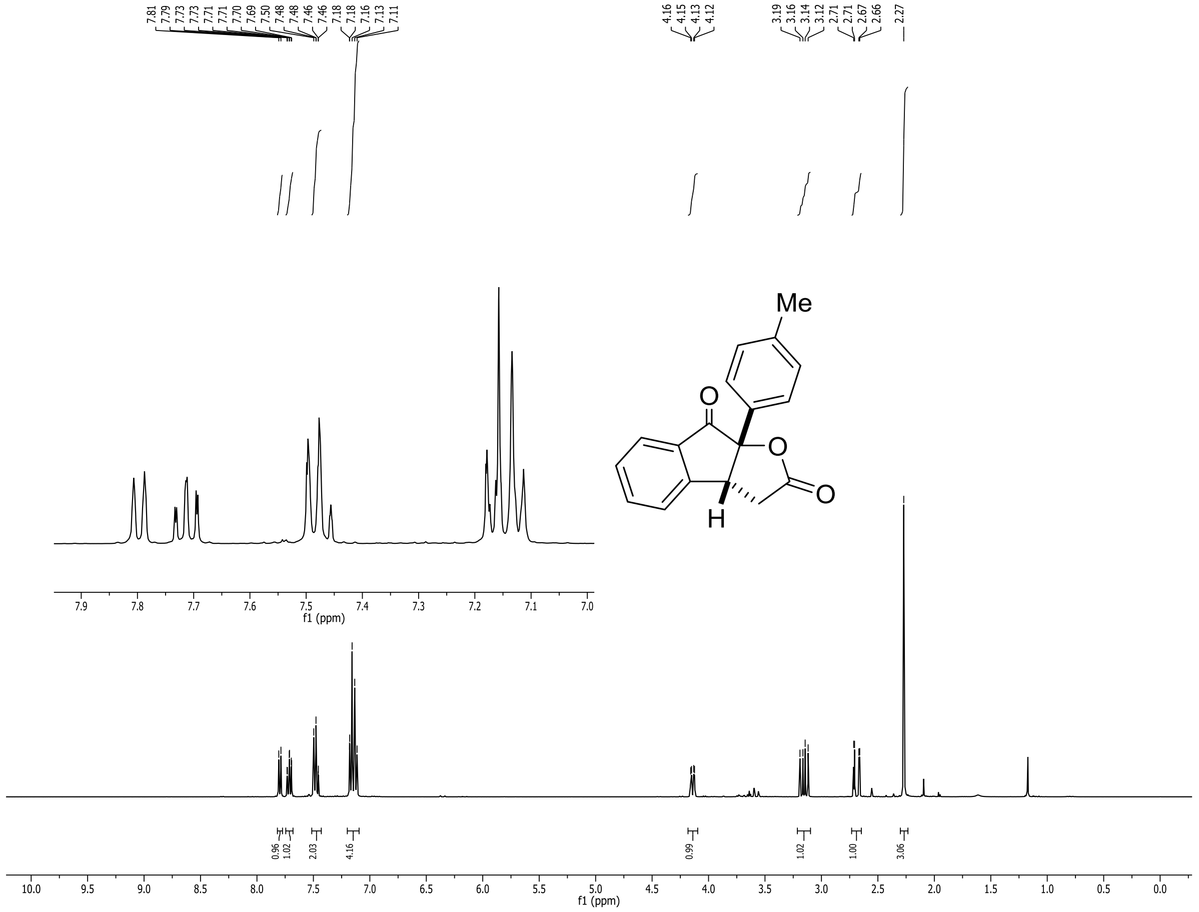

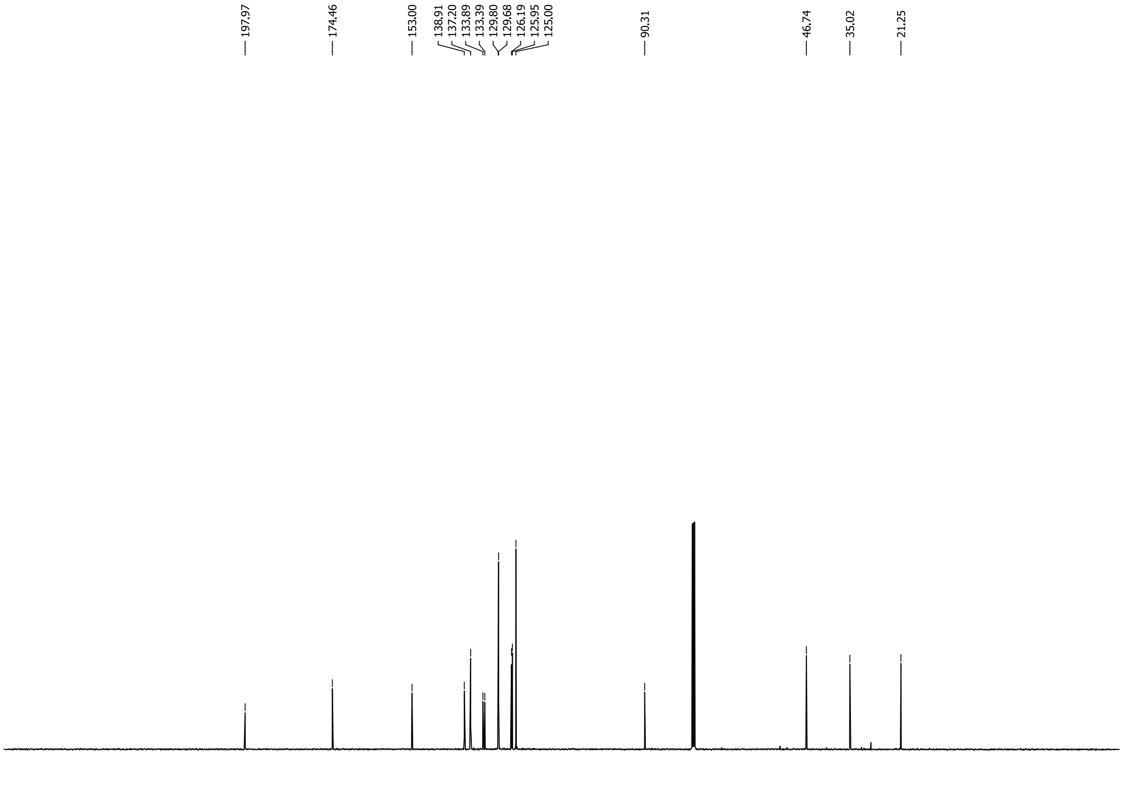

^{1}H and ^{13}C NMR data of compound **13n** (400, 101 MHz, $CDCl_3$)

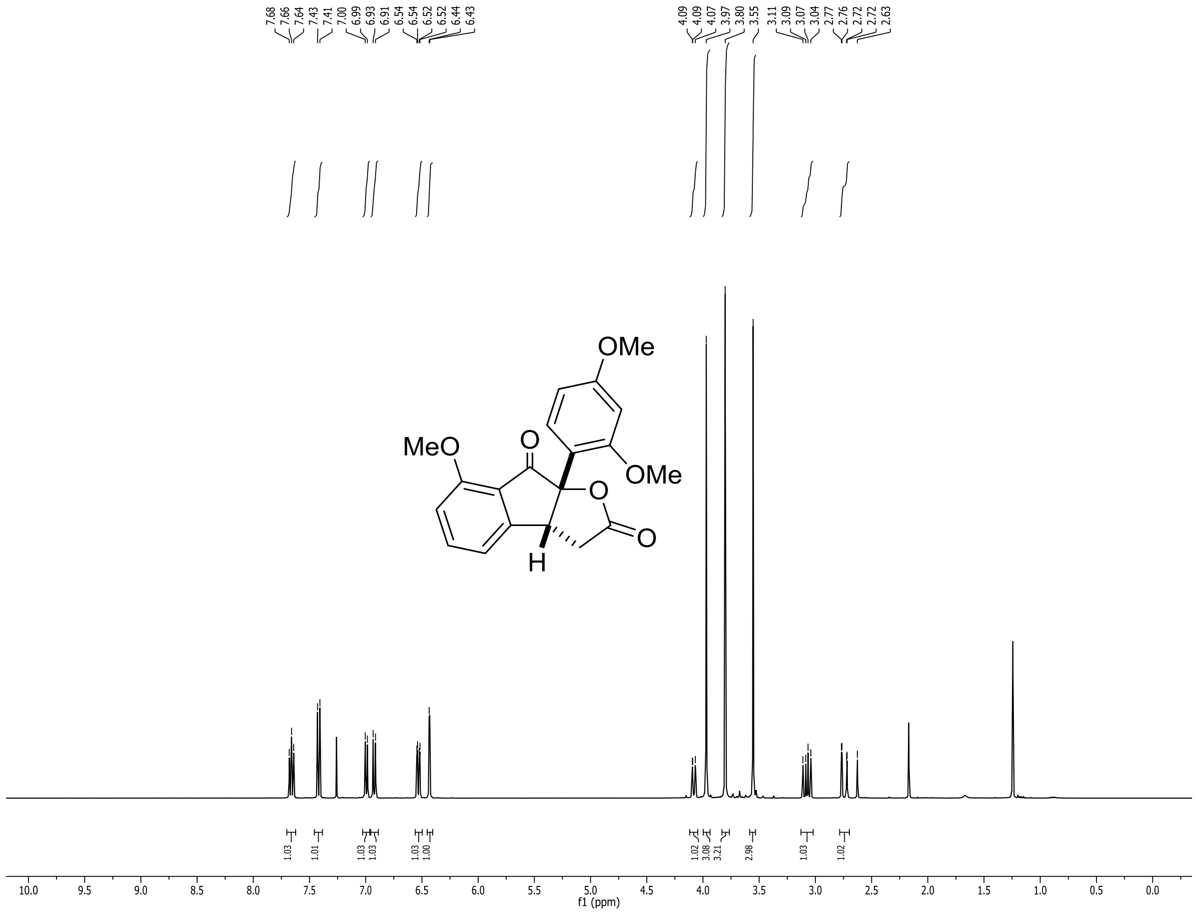

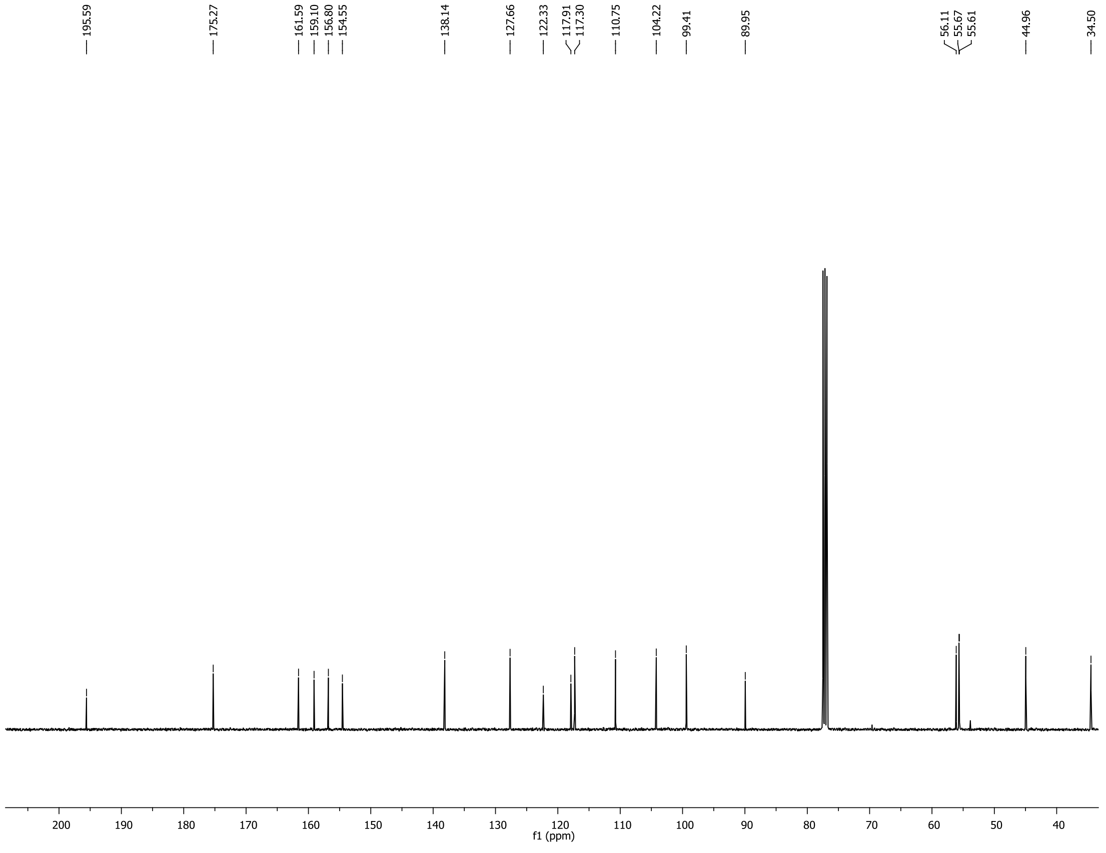

^{1}H and ^{13}C NMR data of compound **14** (400, 101 MHz, $CDCl_3$)

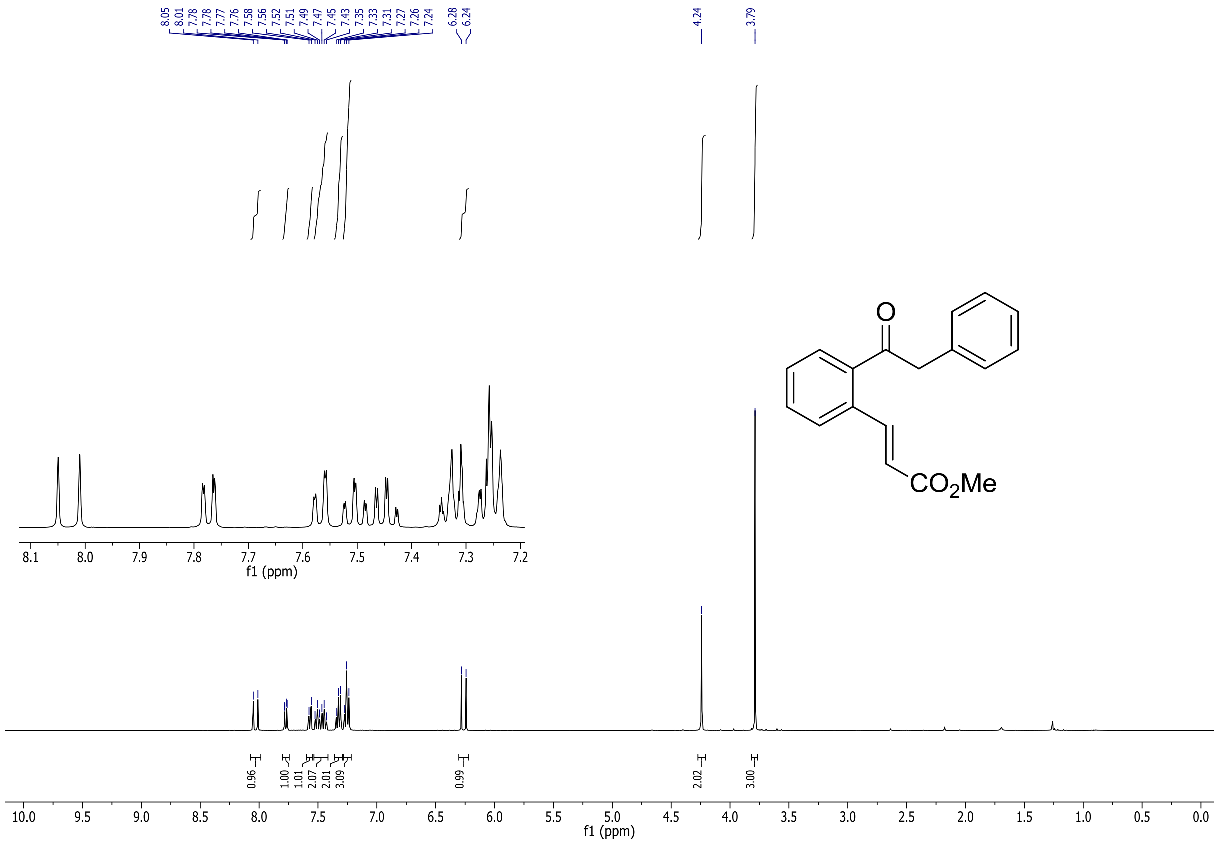

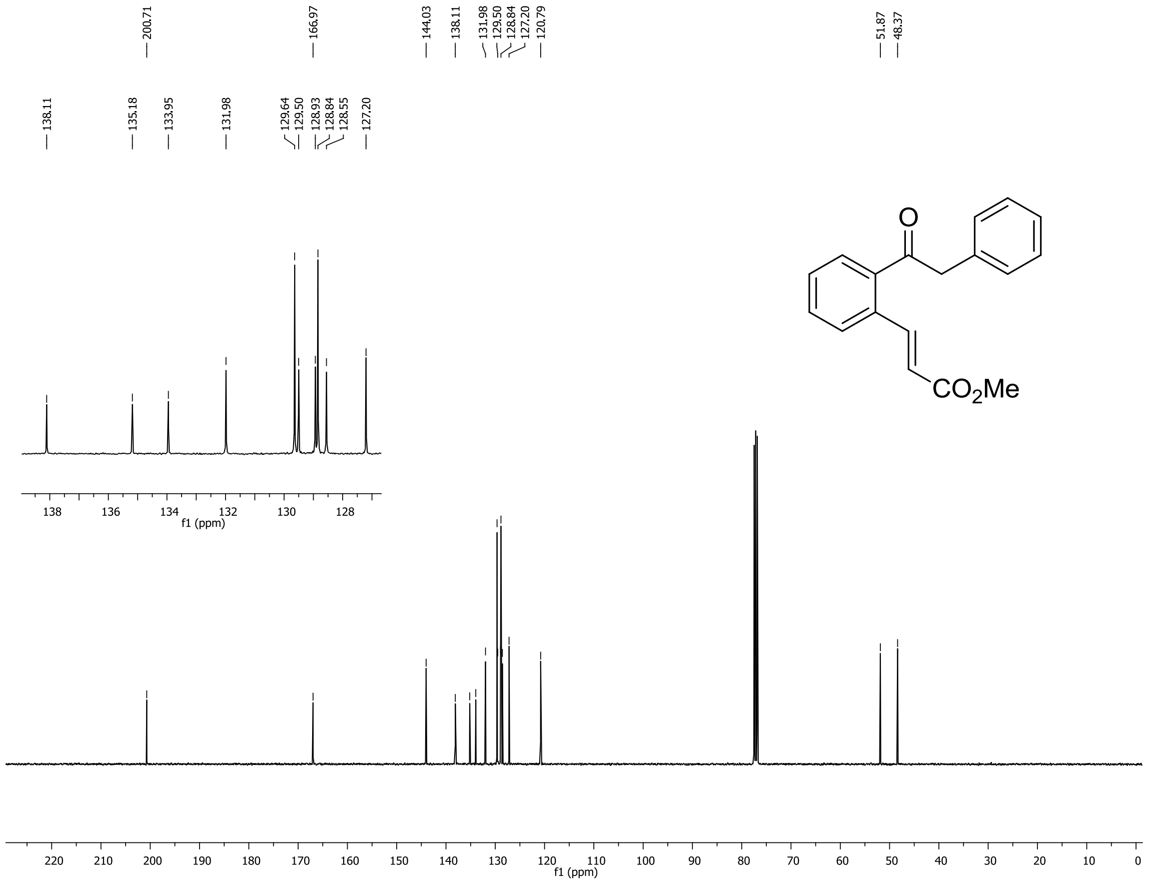

Supplement: Supplementary file 1 — miscellaneous_information [file anie0053-1529-sd1.pdf]
